# Regioselective synthesis of C3 alkylated and arylated benzothiophenes

**DOI:** 10.1038/ncomms14801

**Published:** 2017-03-20

**Authors:** Harry J. Shrives, José A. Fernández-Salas, Christin Hedtke, Alexander P. Pulis, David J. Procter

**Affiliations:** 1 School of Chemistry, University of Manchester, Oxford Road, Manchester M13 9PL, UK

## Abstract

Benzothiophenes are heterocyclic constituents of important molecules relevant to society, including those with the potential to meet modern medical challenges. The construction of molecules would be vastly more efficient if carbon–hydrogen bonds, found in all organic molecules, can be directly converted into carbon–carbon bonds. In the case of elaborating benzothiophenes, functionalization of carbon–hydrogen bonds at carbon-number 3 (C3) is markedly more demanding than at C2 due to issues of regioselectivity (C3 versus C2), and the requirement of high temperatures, precious metals and the installation of superfluous directing groups. Herein, we demonstrate that synthetically unexplored but readily accessible benzothiophene *S*-oxides serve as novel precursors for C3-functionalized benzothiophenes. Employing an interrupted Pummerer reaction to capture and then deliver phenol and silane coupling partners, we have discovered a directing group-free method that delivers C3-arylated and -alkylated benzothiophenes with complete regioselectivity, under metal-free and mild conditions.

## Abstract (web summary)

Benzothiophenes are common motifs in bioactive compounds, but selective functionalization at C3 is challenging. Here the authors report a method starting from benzothiophene S-oxides via an interrupted Pummerer reaction, giving access to a range of C3-alkylated and -arylated products.

Benzothiophenes are sulfur containing heterocyclic molecules that when functionalized, are often incorporated into important molecular scaffolds which have found utility in materials science^1^ and in particular in biology and medicine^2^,^3^,^4^,^5^,^6^,^7^,^8^. For example, sertaconazole^2^ is an antifungal medicine and raloxifene^3^ is used in the prevention of osteoporosis, and other benzothiophenes have promising biological activity in the areas of diabetes^4^, antibacterials^5^ and water regulation^6^ among others^7^,^8^ (Fig. 1a).

The synthesis of these important molecules can be achieved by construction of one or both rings, or via direct functionalization of the benzothiophene core^9^,^10^,^11^. Due to the ubiquity of C–H bonds, the most efficient methods of C–C bond construction are based on C–H functionalization^12^,^13^. Functionalization at C2 of readily available benzothiophenes^14^ is generally well established due to the increased acidity of the C–H bond^15^. However, C3 C–H functionalization is underdeveloped. In the absence of a directing group, direct C–H arylation at C3 of benzothiophenes is traditionally accomplished with palladium catalysts and coupling partners such as aryl-halides^16^,^17^,^18^,^19^, -borons^20^,^21^, -silanes^22^, -sulfonyl chlorides^23^ and-iodoniums^24^ (Fig. 1bi). While recent disclosures address the problems associated with harsh conditions, expensive ligands and regioselectivity issues, the risk of metal contamination remains, particularly when the products are intended for human consumption^25^ or to be used in organic materials where performance can be adversely affected by trace metals^26^.

In contrast to C3 C–H arylation of benzothiophenes, C3 C–H alkylation is significantly more challenging and methods are in drastically short supply as reported procedures are severely limited and often described in isolation: Friedel–Crafts alkylation at C3 is restricted to benzylation and suffers from poor regioselectivity^27^, and C–H metallation at C3, whether stoichiometric^28^ or catalytic with palladium^29^ (at 100 °C with primary alkyl boronic acids) or iridium^30^ (at 90 °C with a diazomalonate), requires an ancillary directing group at C2 to selectively activate the C–H bond at C3 (Fig. 1bii).

Herein, we report a method for the completely regioselective, metal-free C3 C–H functionalization of benzothiophenes that utilizes synthetically unexplored benzothiophene *S*-oxides **1**, readily available from straightforward oxidation of benzothiophenes, and phenol, propargyl silane and allyl silane coupling partners, that does not require a conventional directing group (Fig. 1c). This umpolung strategy couples two carbon sites that are inherently nucleophilic and delivers C3-arylated and the more challenging C3-alkylated benzothiophenes under mild conditions, with broad scope. The reaction operates via activation of the S–O bond in benzothiophene *S*-oxides **1** for an interrupted Pummerer reaction^31^ with phenol, or allyl- or propargyl-silanes to form intermediates **I** and **II**, which are predisposed for charge accelerated [3,3]-sigmatropic rearrangement^32^, resulting in C–C bond formation, therefore delivering the coupling partner in a perfectly site-selective manner.

## Results

### Metal-free C3 C–H arylation

Metal-free methods complement synthetic procedures traditionally based upon the use of transition metals^33^. Recently, we^34^,^35^,^36^,^37^,^38^,^39^,^40^ and others^41^,^42^,^43^,^44^,^45^,^46^,^47^,^48^,^49^,^50^,^51^,^52^,^53^,^54^,^55^,^56^ reported the metal-free, sulfoxide directed^57^ C–H functionalization of a variety of molecular scaffolds, which was enabled by an interrupted Pummerer reaction^28^. Spurred on by these recent achievements, we considered the use of benzothiophene *S*-oxides **1** for the synthesis of important C3-functionalized benzothiophenes, as the corresponding sulfonium salts **I** and **II**, formed after reaction with the coupling partners, lack aromaticity about the five-membered ring and should therefore undergo facile C–C bond formation via [3,3]-sigmatropic rearrangement^58^, thus delivering unexplored reactivity not accessible in benzothiophenes. Surprisingly, benzothiophene *S*-oxides have found limited application in synthesis and their chemistry is relatively unexplored^59^,^60^,^61^. In fact, interrupted Pummerer-type reactivity of benzothiophene *S*-oxides has not been reported until now^62^,^63^. In contrast to previous studies exploiting sulfoxide groups to direct metal-free functionalization, our approach avoids the installation and use of a formal directing group, instead we recruit the sulfur intrinsic to benzothiophenes to capture and deliver the coupling partner.

**Figure 1:**
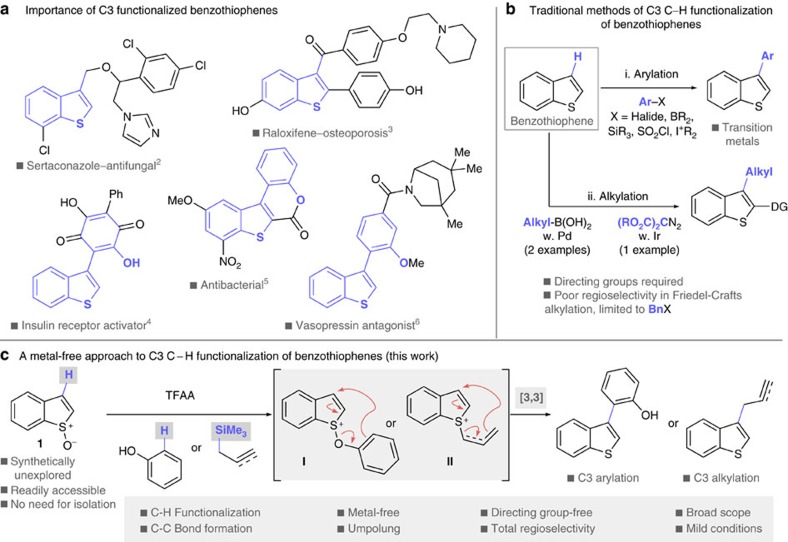
C3-functionalized benzothiophenes. (**a**) Prevalence of C3 functionalized benzothiophene motifs in biologically active molecules including commercial drugs and others with potential in diverse target areas. (**b**) Traditional methods of C3 C–H arylation of benzothiophenes rely on transition metals and alkylation at C3 is limited in scope and either requires a directing group or exhibits poor regioselectivity. (**c**) A metal-free approach to benzothiophene C3 C–H arylation and alkylation employing synthetically unexplored benzothiophene *S*-oxides does not require a directing group and is completely regioselective by virtue of the interrupted Pummerer reaction mechanism which allows the coupling partner to be delivered in a site-selective manner (this work). DG, directing group.

We began our investigation by attempting to couple isolable 5-bromobenzothiophene *S*-oxide **1a** with 4-methylphenol (Table 1). Thus, **1a**, readily prepared from the corresponding benzothiophene by straightforward oxidation with *m*CPBA/BF_3_·OEt_2,_ was treated with trifluoroacetic acid anhydride (TFAA), and reacted with the phenol coupling partner^42^,^43^,^46^. The interrupted Pummerer reaction and, surprisingly, the resulting [3,3]-sigmatropic rearrangement occurred at or below ambient temperature (*cf*. Fig. 1c, **I**). Although the phenol ring is dearomatized during the [3,3]-sigmatropic rearrangement, C–C bond formation is facile due to the lack of aromaticity in the five-membered ring of **I**^58^. After rearomatization of the phenol ring, thioacetal **3a** was formed (in 67% yield when isolated) and its structure elucidated by X-ray crystallography (Table 1). Upon warming the reaction mixture to 45 °C in the presence of *para*-toluene sulfonic acid (*p*TsOH), **3a** opened to form the desired C3-arylated benzothiophene **4a** in high isolated yield (77%) and complete control over regiochemistry with respect to both C3 of the benzothiophene and the *ortho* position of the phenol.

In exploring the scope of the phenol coupling partner, we found that various functionality, including bromo (**4b**,**i**), iodo (**4c**), ester (**4d**), trifluoromethyl (**4e**), nitro (**4g**,**j**), keto (**4h**), amido (**4k**) and chloro (**4l**) were well tolerated, regardless of their position on the phenol ring. 3-Chlorophenol (*cf*. **4l**) and estrone (*cf*. **4m**), containing two inequivalent *ortho* positions, underwent completely regioselective cross-coupling. In the case of **4m** (and later with **4r** and **4t**), opening of the intermediary thioacetal (*cf*. **3a**) proceeded in higher yield when iodine was used in place of *p*TsOH (69% versus 30%).

Although many of the employed benzothiophene *S*-oxides **1** were readily prepared and isolated in 50-85% yield from the corresponding benzothiophenes (for example, those used in the preparation of **4a**, **4q**, **4t** and **4u**, and later for **8a**, **8b**, **8d**, **8e** and **8j**), the chemistry of benzothiophene *S*-oxides **1** is significantly different to that of aryl sulfoxides and, as previously noted^59^, certain substitution patterns in **1** favour a formal [4+2] cycloaddition dimerization upon concentration (for example, benzothiophene *S*-oxides required for the formation of **4f**, **4n**, **4p**, **4r** and **4s**). Using conditions previously reported for the oxidation of thiophenes^64^, we were pleased to find that benzothiophenes could be oxidized with *m*CPBA/BF_3_·OEt_2_, worked-up via a simple filtration and directly applied in the metal-free cross-coupling reaction. This protocol enabled access to C3-arylated benzothiophenes **4** derived from unsubstituted benzothiophene *S*-oxides (*cf*. **4i**-**k**), and those with versatile bromo substitution at all positions of the benzene ring (*cf*. **4n**, **o**, **r** and **s**), as well as chloro substitution at C5 (**4p**). Notably, and in contrast to metal-mediated couplings, the process is broadly compatible with the presence of halide substituents allowing further, downstream functionalization of products.

In addition, the nitro group at C5 (*cf*. **4q**), as well as CO_2_Me (*cf*. **4t**) and phenyl (*cf*. **4u**) at C2, where the corresponding benzothiophene *S*-oxides were isolable, were also well tolerated. The presence of bromo or cyano substituents at C2 of **1** resulted in a complex mixture, presumably due to the instability of the corresponding thioacetals **3** bearing a leaving group at C2. In the case of C2-CO_2_Me substituted benzothiophene, spontaneous lactonization between the phenol and ester moieties formed isothiacoumestan **4t** which constitutes the core structure of the known antibacterial^5^ shown in Fig. 1a. Indeed, the 2-(benzothiophen-3-yl)phenol core strcuture is found in molecules that bear a variety of biological activity (*cf*. Fig. 1a)^4^,^5^,^6^,^7^,^8^.

### Metal-free C3 C–H alkylation

Moving onto the more challenging C3 C–H alkylated benzothiophenes using the metal-free protocol, we established that both allyl silanes **5** and propargyl silanes **7** successfully couple with benzothiophene *S*-oxides **1** to deliver a range of C3-allylated (**6**) and -propargylated benzothiophenes (**8**) with total regiocontrol (Table 2). The allylation proceeded smoothly with allyl silanes that were unsubstituted (**6a**) and that contained a β-methyl (**6b**) substituent, as well as with those bearing reactive functional groups including β-chloromethyl (**6c**), β-methylacetate (**6d**), β-ester (**6e**), β-bromo (**6f**), γ-keto (**6g**) and γ-ester (**6h**) substituents.

The scope of the C3 C–H propargylation with respect to the benzothiophene *S*-oxide **1** was found to be broad and enveloped versatile substituents at various positions. For example, substituted benzothiophene *S*-oxides bearing C2 bromo (**8a**), cyano (**8b**), ester (**8c**), alkyl (**8d**,**e**) and phenyl (**8f**), as well as C5 carrying nitro (**8h**), bromo (**8i**) and aryl (**8j**), and bromo at C6 (**8v**) furnished C3-alkylated benzothiophenes. Again, a procedure involving *in situ* oxidation to the benzothiophene *S*-oxide followed by functionalization proved highly effective (for example, in the formation of **8g**).

The propargylic silane coupling partner **7** was also amenable to variation, with various primary silanes containing primary alkyl (**8a**–**j**,**k**), alkyl halide (**8l**), secondary alkyl (**8m**,**n**), phenyl (**8o**) and silyl (**8p,q**) substituents at the terminal position, as well as unsubstituted propargyl silanes (**8r**), all undergoing efficient metal-free cross coupling. In addition, more challenging hindered secondary propargyl silanes delivered branched products **8s**–**v** that would be inaccessible via conventional C3-alkylation techniques that rely on bromination followed by metalation/electrophilic trapping^14^. Alkylation product **8q** is a key intermediate in the synthesis of an inducer of bacterial biofilms^7^.

### Mechanistic discussion

**Figure 2:**
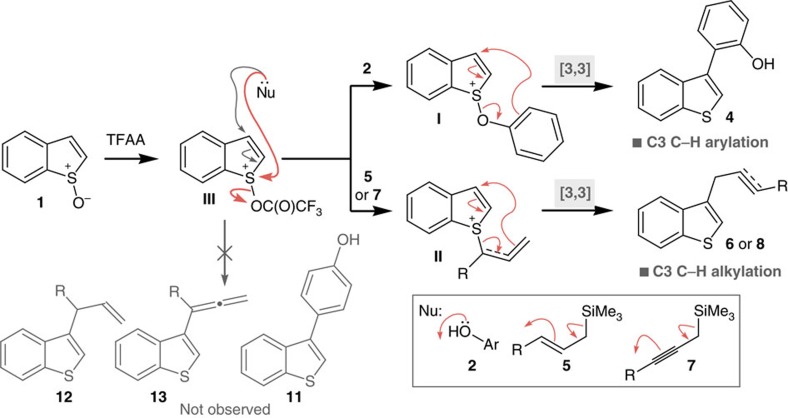
Mechanism of regioselective metal-free C3 C–H functionalization of benzothiophene *S*-oxides. Activated benzothiophene *S*-oxides **III** capture nucleophilic coupling partners prior to regioselective delivery to C3 via a charge accelerated [3,3]-sigmatropic rearrangement of intermediates **I** and **II**. The expected products of direct addition of nucleophiles to **III**, the *para*-substituted phenols **11**, alkenes **12** and allenes **13**, were not observed. Nu, nucleophile.

The mechanisms of the metal-free C3 C–H arylation and alkylation processes are intrinsically related. Based upon our previous and present experimental observations, we propose that an interrupted Pummerer reaction to form **I** or **II**, via addition of oxygen of phenols **2** (refs 42, 43, 46) or S_E_2′ addition of the silanes **5** (refs 34, 35, 45) and **7** (refs 36, 37, 38) to the sulfur of activated benzothiophene *S*-oxide **III**, precedes a charge accelerated [3,3]-sigmatropic rearrangement, resulting in complete regioselective C–C bond formation at the expense of a C–H bond (Fig. 2). Due to the rapid [3,3]-sigmatropic rearrangement resulting from the lack of aromaticity in **I** and **II**, we were unable to isolate or observe these sulfonium salts. Evidence for these mechanisms, rather than a direct addition to C3 of **III**, comes by way of the exclusive formation of *ortho* substituted phenols **4**, allylated products **6g** and **6h** and propargylated products **8**; *para*-substituted phenols **11**, C3-allylated benzothiophenes **12** and C3-allenyl benzothiophenes **13** would be expected from direct addition but were not observed.

## Discussion

In summary, we have described a metal-free approach that harnesses the synthetic potential of benzothiophene *S*-oxides, readily accessible from benzothiophenes, to generate C3-functionalized benzothiophenes at the expense of C–H bonds. The absolute regiocontrol observed stems from the ability of the activated benzothiophene *S*-oxide to first capture the nucleophilic coupling partner and then deliver it to C3. The method utilizes readily available coupling partners, has broad scope and by virtue of the facile interrupted Pummerer reaction and charge accelerated [3,3]-sigmatropic rearrangement sequence, the latter facilitated by the non-aromatic benzothiophenium salt intermediates (*cf*. **I** and **II**), operates under mild conditions. This directing group-free method delivers C3-arylated products that map directly onto medicinally relevant scaffolds, and unlike previously reported methods for C3 C–H alkylation of benzothiophenes, the process does not require a directing group at C2, thus making available greater diversity in important benzothiophene scaffolds.

## Methods

### General

Supplementary Figures 1–61 for the NMR spectra, Supplementary Fig. 62 for the X-ray crystallographic analysis of **3a**, Supplementary Tables 1–7 for X-ray crystallographic data, and Supplementary Methods giving full experimental details and the characterization of compounds are given in the Supplementary Information.

### General procedure for C3 C–H arylation of benzothiophene *S*-oxides

To an N_2_ flushed, oven dried reaction vessel equipped with a magnetic stir bar, benzothiophene *S*-oxide **1** (0.2 mmol) and CH_2_Cl_2_ (1 ml) were added. The mixture was stirred at −40 °C and TFAA (0.3 mmol) was added. After 5 min, phenol **2** (0.3 mmol) dissolved in CH_2_Cl_2_ (1 ml) was added and the mixture stirred for 15 min, before removing the cooling bath and stirring the mixture at ambient temperature overnight (∼16 h). *p*TsOH (0.4 mmol) was added, and the mixture heated at 45 °C for 5 h. Water (3 ml) was added and the aqueous phase was extracted with CH_2_Cl_2_ (3 × 5 ml). The combined organic phases were dried over MgSO_4_ and concentrated *in vacuo*. The crude mixture was purified by column chromatography on silica gel to give pure C3-arylated benzothiophenes **4**.

### General procedure for C3 C–H alkylation of benzothiophene *S*-oxides

To an N_2_ flushed, oven dried reaction vessel equipped with a magnetic stir bar, benzothiophene *S*-oxide **1** (0.2 mmol), silane **5** or **7** (0.3 mmol) and MeCN (1 ml) were added. The mixture was stirred at 0 °C and TFAA (0.3 mmol) was added. The cooling bath was removed and the mixture stirred at ambient temperature overnight (∼16 h). Saturated NaHCO_3_(aq) (3 ml) was added and the aqueous phase was extracted with EtOAc (3 × 5 ml). The combined organic phases were dried over MgSO_4_ and concentrated *in vacuo*. The crude mixture was purified by column chromatography on silica gel to give pure C3-allylated (**6**) or -propargylated (**8**) benzothiophenes.

### Data availability

The X-ray crystallographic coordinates for **3a** have been deposited at the Cambridge Crystallographic Data Centre (CCDC) under deposition number CCDC 1511568. This data can be obtained free of charge from the CCDC via www.ccdc.cam.ac.uk/data_request/cif. The authors declare that all other data supporting the findings of this study are available within the article and its Supplementary Information file.

## Additional information

**How to cite this article:** Shrives, H. J. *et al*. Regioselective synthesis of C3 alkylated and arylated benzothiophenes. *Nat. Commun.* **8,** 14801 doi: 10.1038/ncomms14801 (2017).

**Publisher's note**: Springer Nature remains neutral with regard to jurisdictional claims in published maps and institutional affiliations.

## Supplementary Material

Supplementary Information Supplementary figures, supplementary tables, supplementary methods and supplementary references.

## Figures and Tables

**Table 1:**
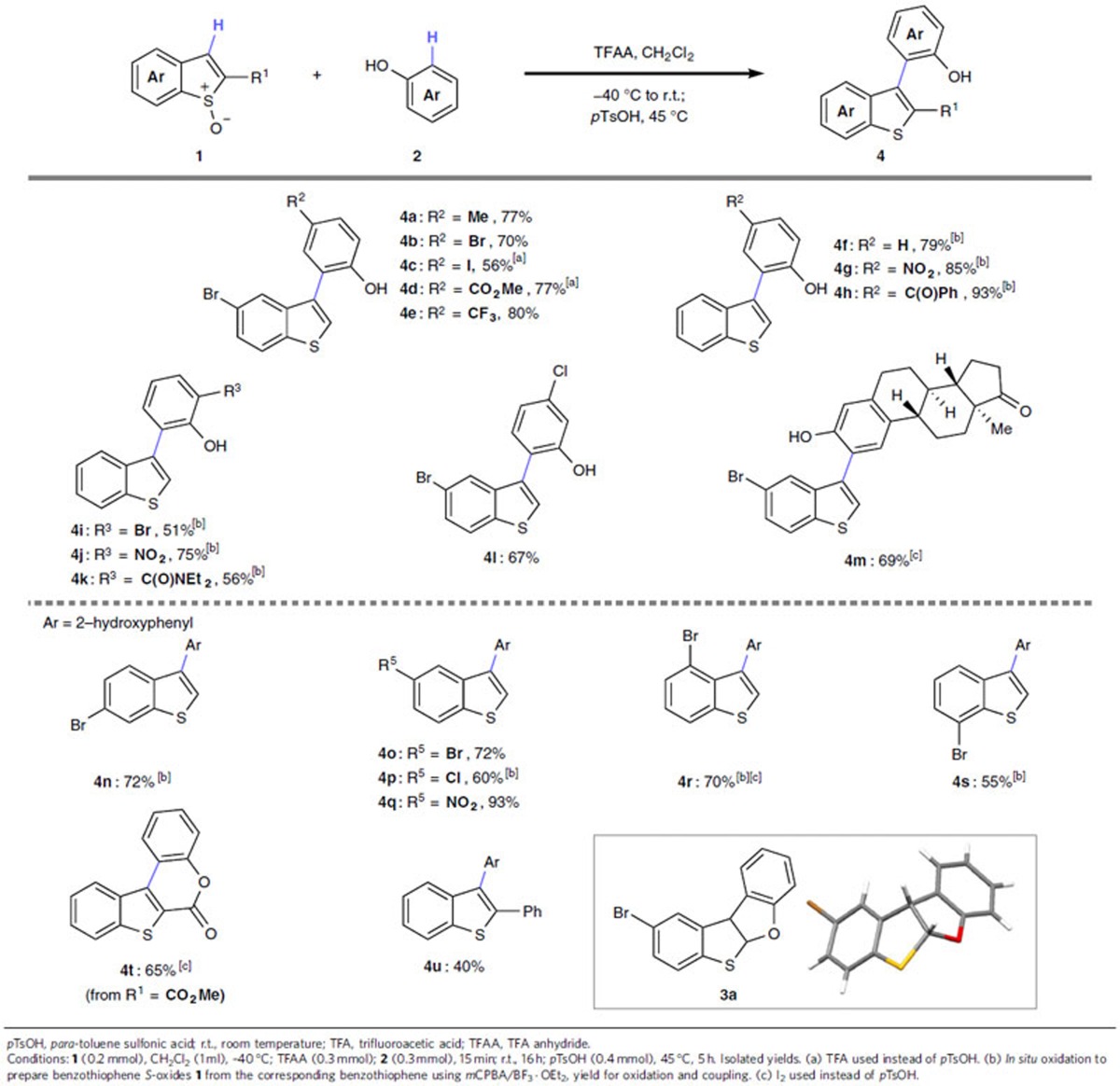
Metal-free C3 C–H arylation of benzothiophene *S*-oxides.

**Table 2:**
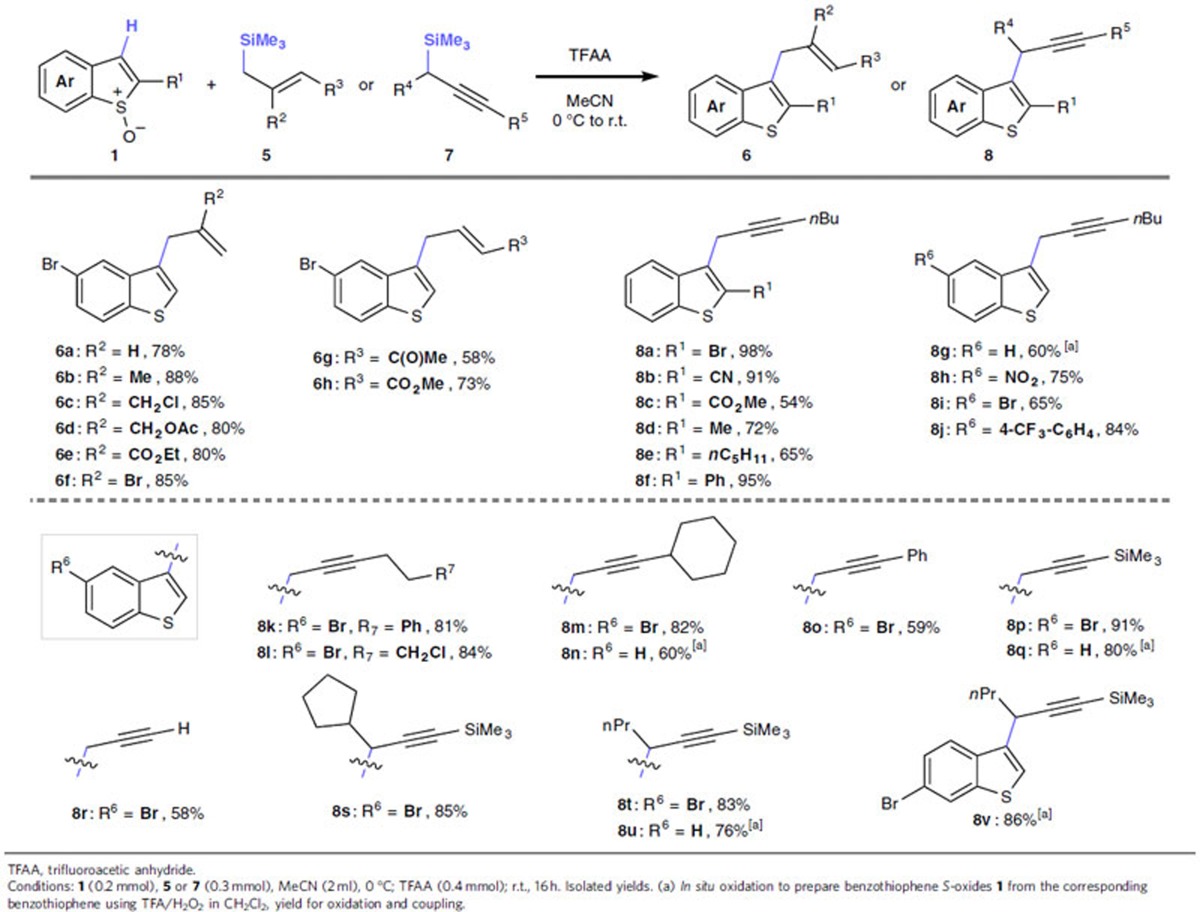
Metal-free C3 C–H alkylation of benzothiophene *S*-oxides.
