# Supplementary material for: Regioselective synthesis of C3 alkylated and arylated benzothiophenes
Source: Nat Commun. 2017 Mar 20;8:14801. doi: 10.1038/ncomms14801 (PMC5364387; doi:10.1038/ncomms14801)
Supplement: Supplementary Information — Supplementary figures, supplementary tables, supplementary methods and supplementary references. [file ncomms14801-s1.pdf]

Supplementary methods

General Experimental

All experiments were performed under an atmosphere of nitrogen, using anhydrous solvents, unless stated otherwise. THF was distilled from sodium/benzophenone and CH_2Cl_2 was distilled from CaH_2. All other solvents and reagents were purchased from commercial sources and used as supplied.

^{1}H NMR spectra were recorded on a 400 or 500 MHz spectrometer. ^{13}C NMR spectra were recorded on a 101 or 125 MHz spectrometer. All chemical shift values are reported in ppm, with coupling constants in Hz. Mass spectra were obtained using positive or negative electrospray (ESI), electron ionization (EI), atmospheric sample analysis probe (ASAP), atmospheric pressure chemical ionization (APCI) or gas chromatography-mass spectrometry methodology. Infrared spectra were recorded as evaporated films or neat using FT/IR spectrometers.

Melting points were measured on solids as obtained after chromatography. Column chromatography was carried out using 35 – 70 μ, 60Å silica gel. Routine TLC analysis was carried out on silica gel 60 F254 coated aluminium sheets of 0.2 mm thickness. Plates were viewed using a 254 nm ultraviolet lamp and developed by dipping in aqueous potassium permanganate solution.

General procedure A. Oxidation of Benzothiophenes

To an oven dried vial under nitrogen was added the benzo[b]thiophene, CH_2Cl_2 and TFA. H_2O_2 (30% aq.) was added to the solution at room temperature and the reaction monitored by TLC (5% EtOAc in $CHCl_3$). More H_2O_2 was added until complete consumption of the starting material was observed. The reaction was then quenched with $NaHCO_3$ at 0 $^{\circ}$C and the aqueous phase extracted with CH_2Cl_2. The combined organic layers were dried with $MgSO_4$ and concentrated *in vacuo*. The crude mixture was purified by column chromatography (5% EtOAc in $CHCl_3$).

Benzothiophene *S*-oxides: Characterization data

5-Bromobenzo[b]thiophene *S*-oxide 1a

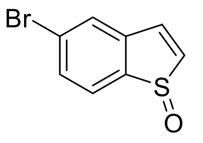

As described in general procedure **A**, 5-bromobenzo[b]thiophene (213 mg, 1.0 mmol) and H_2O_2 (0.1 mL, 1.0 mmol) in CH_2Cl_2 and trifluoroacetic acid (1:1), gave **1a** (171 mg, 0.74 mmol, 74%) as an white solid; $\boldsymbol{\delta_H}$ (400 MHz, $CDCl_3$) 7.12 - 7.20 (2 H, m, ArC*H*), 7.60 - 7.64 (1 H, m, ArC*H*), 7.67 (1 H, d, *J* = 1.8 Hz, ArC*H*), 7.79 (1 H, d, *J* = 8.0 Hz, ArC*H*); $\boldsymbol{\delta_C}$ (101 MHz, $CDCl_3$) 126.6 (Ar*C*Br), 127.4 (Ar*C*H), 128.1 (Ar*C*H), 131.8 (Ar*C*H), 133.6 (Ar*C*H), 138.9 (Ar*C*), 139.4 (Ar*C*H), 144.0 (Ar*C*); $\boldsymbol{\nu_{max}}$ (neat)/cm^{-1} 705, 772, 810, 894, 940, 1023, 1095, 1189, 1290, 1313, 1442, 1528, 1573, 3002, 3053, 3074; **HRMS** (APCI): Calcd. for C_8H_6SO $(M+H)^+$, 228.9317; found 228.9317.

5-Nitrobenzo[b]thiophene *S*-oxide 1b

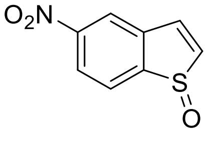

As described in general procedure **A**, 5-nitrobenzo[b]thiophene (179 mg, 1.0 mmol) and H_2O_2 (0.1 mL, 1.0 mmol) in CH_2Cl_2 and trifluoroacetic acid (1:1), gave **1b** (146 mg, 0.73 mmol, 73%) as a yellow solid; $\boldsymbol{\delta_H}$ (400 MHz, $CDCl_3$) 7.34 (2 H, s, ArC*H*), 8.09 - 8.15 (1 H, m, ArC*H*), 8.37 (2 H, dq, *J* = 4.4, 2.1 Hz, ArC*H*); $\boldsymbol{\delta_C}$ (101 MHz, $CDCl_3$) 120.2 (Ar*C*H), 124.2 (Ar*C*H), 127.4 (Ar*C*H), 133.5 (Ar*C*), 138.8 (Ar*C*), 141.2 (Ar*C*H), 150.8 (Ar*C*), 151.4 (Ar*C*H); $\boldsymbol{\nu_{max}}$ (neat)/cm^{-1} 736, 843, 953, 1021, 1045, 1094, 1200, 1290, 1347, 1526, 3060, 3077; **HRMS** (APCI): Calcd. for $C_8H_6SO_3N$ $(M+H)^+$, 196.0063; found 196.0063.

Methyl benzo[*b*]thiophene-2-carboxylate 1-oxide[1] 1c

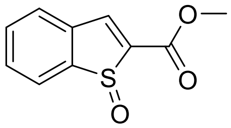

As described in general procedure **A**, methyl benzo[*b*]thiophene-2-carboxylate (96.1 mg, 0.5 mmol) and H_2O_2 (0.05 mL, 0.5 mmol) in CH_2Cl_2 and trifluoroacetic acid (1:1), gave **1c** (76.4 mg, 0.36 mmol, 72%) as a yellow solid; $\boldsymbol{\delta_H}$ (400 MHz, $CDCl_3$) 3.98 (3 H, s, C*H*$_3$), 7.57 - 7.66 (3 H, m, ArC*H*), 7.94 - 7.98 (2 H, m, ArC*H*); $\boldsymbol{\delta_C}$ (101 MHz, $CDCl_3$) 53.3 (*C*H$_3$), 127.2 (Ar*C*H), 127.3 (Ar*C*H), 131.9 (Ar*C*H), 132.7 (Ar*C*H), 135.4 (Ar*C*), 142.8 (Ar*C*H), 143.5 (Ar*C*), 147.0 (Ar*C*), 161.8 (*C*=O); $\boldsymbol{\nu_{max}}$ (neat)/cm^{-1} 756, 850, 947, 1032, 1068, 1175, 1234, 1341, 1434, 1561, 1714, 2959, 3046; **HRMS** (APCI): Calcd. for $C_{10}H_9SO_3$ $(M+H)^+$, 209.0267; found 209.0260.

2-Phenylbenzo[b]thiophene *S*-oxide 1d

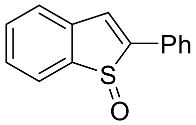

As described in general procedure **A**, 2-phenylbenzo[b]thiophene (186 mg, 1.0 mmol) and H_2O_2 (0.1 mL, 1.0 mmol) in CH_2Cl_2 and trifluoroacetic acid (1:1), gave **1d** (153 mg, 0.76 mmol, 76%) as a yellow solid; $\boldsymbol{\delta_H}$ (400 MHz, $CDCl_3$) 7.28 (1 H, s, ArC*H*), 7.39 - 7.56 (6 H, m, ArC*H*), 7.78 - 7.84 (2 H, m, ArC*H*), 7.94 (1 H, dd, *J* = 7.5, 0.8 Hz, ArC*H*); $\boldsymbol{\delta_C}$ (101 MHz, $CDCl_3$) 124.5 (Ar*C*H), 126.4 (Ar*C*H), 126.6 (Ar*C*H), 127.0 (Ar*C*H), 128.4 (Ar*C*H), 129.2 (Ar*C*H), 129.5 (Ar*C*H), 130.8 (Ar*C*), 132.3 (Ar*C*H), 137.7 (Ar*C*), 144.2 (Ar*C*), 152.4 (Ar*C*); $\boldsymbol{\nu_{max}}$ (neat)/cm^{-1} 682, 731, 894, 996, 1061, 1447, 1585, 3050; **HRMS** (APCI): Calcd. for $C_{14}H_{11}SO$ $(M)^+$, 227.0525; found 227.0524.

2-Bromobenzo[b]thiophene *S*-oxide 1e

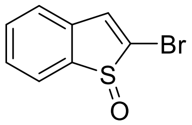

As described in general procedure **A**, 2-bromobenzo[b]thiophene (0.21 g, 1.0 mmol) and H_2O_2 (0.1 mL, 1.0 mmol) in CH_2Cl_2 and trifluoroacetic acid (1:1), gave **1e** (0.13 g, 0.57 mmol, 57%) as an orange oil; $\boldsymbol{\delta_H}$ (400 MHz, $CDCl_3$) 7.23 (1 H, d, *J* = 0.5 Hz, ArC*H*), 7.36 - 7.49 (3 H, m, ArC*H*), 7.82 (1 H, dt, *J* = 7.4, 0.6 Hz, ArC*H*); $\boldsymbol{\delta_C}$ (101 MHz, $CDCl_3$) 123.9 (Ar*C*H), 126.3 (Ar*C*H), 128.5 (Ar*C*H), 131.6 (Ar*C*), 132.4 (Ar*C*H), 134.3 (Ar*C*H), 137.3 (Ar*C*), 145.2 (Ar*C*); $\boldsymbol{\nu_{max}}$ (neat)/cm^{-1} 700, 772, 819, 888, 942, 1023, 1078, 1190, 1278, 1308, 1441, 1519, 1599, 3017; **HRMS** (EI): Calcd. for C_8H_5SBrO $(M)^+$, 227.9244; found 227.9240.

2-Cyanobenzo[b]thiophene *S*-oxide 1f

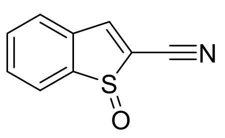

As described in general procedure **A**, 2-cyanobenzo[b]thiophene (159 mg, 1.0 mmol) and H_2O_2 (0.1 mL, 1.0 mmol) in CH_2Cl_2 and trifluoroacetic acid (1:1), gave **1f** (87.4 mg, 0.5 mmol, 50%) as an orange oil; **δ_H** (400 MHz, $CDCl_3$) 7.64 - 7.74 (3 H, m, ArC*H*), 7.83 (1 H, d, *J* = 0.5 Hz, ArC*H*), 7.97 (1 H, d, *J* = 7.1 Hz, ArC*H*); **δ_C** (101 MHz, $CDCl_3$) 112.3 (Ar*C*), 125.0 (*C*N), 126.9 (Ar*C*H), 127.1 (Ar*C*H), 132.2 (Ar*C*H), 133.1 (Ar*C*H), 134.7 (Ar*C*), 145.7 (Ar*C*), 145.9 (Ar*C*H); **ν_{max}** (neat)/cm^{-1} 773, 919, 1040, 1065, 1078, 1198, 1442, 1548, 2220, 3050, 3058, 3082; **HRMS** (APCI): Calcd. for C_9H_6SON $(M+H)^+$, 176.0165; found 176.0160.

2-Methylbenzo[b]thiophene *S*-oxide 1g

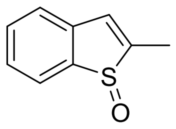

As described in general procedure **A**, 2-methylbenzo[b]thiophene (296 mg, 2.0 mmol) and H_2O_2 (0.2 mg, 2.0 mmol) in CH_2Cl_2 and trifluoroacetic acid (1:1), gave **1g** (283 mg, 0.17 mmol, 86%) as a yellow solid; **δ_H** (400 MHz, $CDCl_3$) 2.39 (3 H, d, *J* = 1.5 Hz, C*H*$_3$), 6.78 (1 H, s, ArC*H*), 7.33 - 7.40 (2 H, m, ArC*H*), 7.42 - 7.49 (1 H, m, ArC*H*), 7.85 (1 H, d, *J* = 7.5 Hz, ArC*H*); **δ_C** (101 MHz, $CDCl_3$) 13.0 (*C*H$_3$), 123.6 (Ar*C*H), 126.2 (ArC*H*), 127.6 (ArC*H*), 128.6 (ArC*H*), 132.0 (ArC*H*), 138.1 (ArC), 144.5 (ArC), 150.4 (ArC); **ν_{max}** (neat)/cm^{-1} 762, 901, 1021, 1057, 1447, 1459, 1587, 1711, 2911, 3047; **HRMS** (APCI): Calcd. for C_9H_9SO $(M+H)^+$, 165.0369; found 165.0370.

2-Pentylbenzo[b]thiophene *S*-oxide 1h

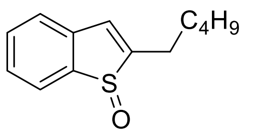

As described in general procedure **A**, 2-pentylbenzo[b]thiophene (0.37 g, 1.79 mmol) and H_2O_2 (0.18 mL, 1.79 mmol) in CH_2Cl_2 and trifluoroacetic acid (1:1), gave **1h** (0.30 g, 1.37 mmol, 76%) as an orange oil; **δ_H** (400 MHz, $CDCl_3$) 0.93 (3 H, t, *J* = 7.1 Hz, C*H*$_3$), 1.34 - 1.47 (4 H, m, 2 × C*H*$_2$), 1.67 - 1.85 (2 H, m, C*H*$_2$), 2.74 (2 H, td, *J* = 7.6, 1.5 Hz, C*H*$_2$), 6.77 (1 H, d, *J* = 0.6 Hz, ArC*H*), 7.34 - 7.41 (2 H, m, ArC*H*), 7.44 - 7.51 (1 H, m, ArC*H*), 7.84 - 7.90 (1 H, m, ArC*H*); **δ_C** (101 MHz, $CDCl_3$) 14.0 (*C*H$_3$), 22.3 (*C*H$_2$), 27.3 (*C*H$_2$), 28.4 (*C*H$_2$), 31.3 (*C*H$_2$), 123.7 (Ar*C*H), 126.2 (Ar*C*H), 127.5 (Ar*C*H), 127.6 (Ar*C*H), 132.0 (Ar*C*H), 138.1 (Ar*C*), 144.3 (Ar*C*), 155.5 (Ar*C*); **ν_{max}** (neat)/cm^{-1} 692, 734, 878, 976, 1069, 1444, 1582, 2846, 2968, 3050; **HRMS** (APCI): Calcd. for $C_{13}H_{16}SO$ $(M)^+$, 220.0922; found 220.0920.

5-[4-(Trifluoromethyl)phenyl]benzo[b]thiophene *S*-oxide 1i

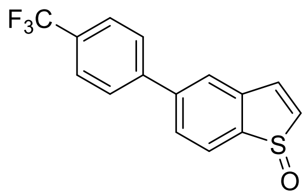

As described in general procedure **A**, to 5-(4-(trifluoromethyl)phenyl)benzo[b]thiophene (59.2 mg, 0.21 mmol) and H_2O_2 (0.02 mL, 0.21 mmol) in CH_2Cl_2 and trifluoroacetic acid (1:1), gave **1i** (50.1 mg, 0.17 mmol, 85%) as a brown solid; $\boldsymbol{\delta_H}$ (400 MHz, $CDCl_3$) 7.18 (1 H, d, *J* = 6.0 Hz, ArC*H*), 7.30 (1 H, dd, *J* = 6.1, 0.6 Hz, ArC*H*), 7.67 - 7.77 (6 H, m, ArC*H*), 8.03 (1 H, d, *J* = 7.8 Hz, ArC*H*); $\boldsymbol{\delta_C}$ (101 MHz, $CDCl_3$) 124.0 (q, *J* = 271.9 Hz, *C*F_3), 123.8 (Ar*C*H), 126.0 (q, *J* = 3.6 Hz, Ar*C*H), 126.7 (Ar*C*H), 127.7 (Ar*C*H), 127.9 (Ar*C*H), 130.5 (q, *J* = 32.8 Hz, Ar*C*CF_3), 134.5 (Ar*C*H), 138.0 (Ar*C*), 138.7 (Ar*C*H), 143.0 (Ar*C*), 143.9 (Ar*C*), 144.8 (Ar*C*); $\boldsymbol{\nu_{max}}$ (neat)/cm^{-1} 770, 841, 942, 1013, 1069, 1108, 1177, 1323, 1596, 1617, 3049; **HRMS** (APCI): Calcd. for $C_{15}H_{10}SOF_3$ $(M+H)^+$, 295.0399; found 295.0389.

General procedure B. Coupling of benzo[b]thiophene *S*-oxides with phenols

The thiophene *S*-oxide (**1**) (0.2 mmol) was dissolved in CH_2Cl_2 (1 mL, 0.2 M) in an oven-dried tube flushed with N_2. TFAA (0.3 mmol, 1.5 equiv.) was then added at −40 ºC. After 5 min, the corresponding phenol (**2**) (1.5 equiv.) dissolved in CH_2Cl_2 (1 mL) was added and the mixture stirred for 15 min at −40ºC before removing the cooling bath and stirring the mixtures at ambient temperature overnight (16 h). *p*TsOH (2 equiv.) or I_2 (2 equiv.) or TFA (4 equiv.) (indicated in each case) was then added, and the mixture was heated at 45 ºC for 5 h. The solution was quenched with H_2O (3 mL), and the aqueous layer was extracted with CH_2Cl_2 (3 x 5 mL). The combined organic layers were dried ($MgSO_4$) and concentrated *in vacuo*. The crude product was purified by column chromatography on silica gel eluting with toluene (indicated if different eluent was used).

General procedure C. Coupling of benzo[b]thiophene with phenols-*in situ* oxidation and coupling.

The benzothiophene (**1**) (0.5 mmol) was dissolved in CH_2Cl_2 (2 mL, 0.25 M) in an oven-dried tube flushed with N_2 at −20 ºC and $BF_3 \cdot OEt_2$ (8 equiv.) was added. *m*CPBA (1.2 equiv.) in CH_2Cl_2 (1 mL) was then added in portions over 1.5 h at the same temperature. The reaction was monitored by TLC, and after the disappearance of the starting material, saturated Na_2CO_3 (0.2 mL) was added to the mixture, followed by K_2CO_3 (100 mg) at −20 ºC. The mixture was then filtered through a plug loaded with $MgSO_4$ and K_2CO_3, washing with CH_2Cl_2 (5 mL). The resulting solution was cooled to −40ºC and TFAA (0.3 mmol, 1.5

equiv.) was added. After 5 min, the corresponding phenol (**2**) (1.5 equiv.) dissolved in CH_2Cl_2 (1 mL) was added, and the mixture stirred for 15 min at −40ºC before removing the cooling bath and stirring the mixtures at ambient temperature overnight (16 h). *p*TsOH (2 equiv.) or I_2 (2 equiv.) was then added, and the mixture was heated at 45 ºC for 5h. The solution was quenched with H_2O (8 mL) and the aqueous layer was extracted with CH_2Cl_2 (3 × 5 mL). The combined organic layers were dried ($MgSO_4$) and concentrated *in vacuo*. The crude product was purified by column chromatography on silica gel eluting with toluene (indicated if different eluent was used).

C3 arylation. Characterization data.

9-Bromo-5a,10b-dihydrobenzo[4,5]thieno[2,3-b]benzofuran 3a

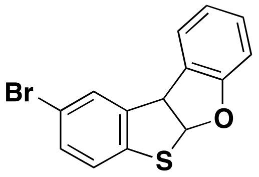

5-Bromo-benzo[b]thiophene *S*-oxide (**1a)** (0.2 mmol) was dissolved in CH_2Cl_2 (1 mL, 0.2 M) in an oven-dried tube flushed with N_2. TFAA (41 µl, 0.3 mmol) was then added at -40 ºC. After 5 min, phenol (**2f**) (29 mg, 0.3 mmol), dissolved in CH_2Cl_2 (1 mL), was added at the same temperature. The mixture was then allowed to warm to room temperature and stirred overnight. The solution was quenched with H_2O (3 mL) and the aqueous layer was extracted with CH_2Cl_2 (3 x 5 mL). The combined organic layers were dried ($MgSO_4$) and concentrated in vacuo. Purification by column chromatography on silica gel eluting with 5% AcOEt in *n*-hexanes, gave **3a** (41 mg, 0.134 mmol, 67%) as white solid; δ_H (400 MHz, $CDCl_3$) 5.23 (1 H, d, *J* = 8.0 Hz, C*H*), 6.88 (2 H, d, *J* = 8.0 Hz, C*H* + ArC*H*), 6.98 (1 H, td, *J* = 7.5, 0.9 Hz, ArC*H*), 7.05 (1 H, d, *J* = 8.5 Hz, ArC*H*), 7.17 - 7.24 (1 H, m, ArC*H*), 7.29 (1 H, ddd, *J* = 8.3, 1.9, 0.8 Hz, ArC*H*), 7.41 (1 H, d, *J* = 7.5 Hz, ArC*H*), 7.51 (1 H, dd, *J* = 1.9, 0.9 Hz, ArC*H*); δ_C (101 MHz, $CDCl_3$) 56.5 (*C*H), 95.2 (*C*H), 110.7 (Ar*C*H), 118.6 (Ar*C*Br), 122.1 (Ar*C*H), 123.5 (Ar*C*H), 124.2 (Ar*C*H), 127.0 (Ar*C*), 127.7 (Ar*C*H), 129.6 (Ar*C*H), 131.7 (Ar*C*H), 138.4 (Ar*C*), 141.8 (Ar*C*), 158.4 (Ar*C*). **HRMS** (ESI): Calcd. for $C_{14}H_9OSBr$ $(M-H)^+$, 303.9550; found 303.9952.

2-(5-Bromobenzo[*b*]thiophen-3-yl)-4-methylphenol 4a

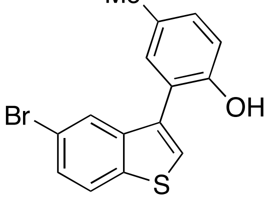

As described in general procedure **B**, 5-bromo-benzo[b]thiophene *S*-oxide (**1a)** (46 mg, 0.2 mmol), *p*-cresol (**2a)** (32 mg, 0.3 mmol), trifluoroacetic anhydride (41 µl, 0.3 mmol) and *p*TsOH (69 mg, 0.4 mmol), gave **4a** (49 mg, 0.154 mmol, 77%) as a colourless oil; δ_H

(500 MHz, $CDCl_3$) 2.35 (3H, s, C*H*$_3$) , 4.85 (1 H, s, O*H*), 6.95 (1 H, d, *J* = 8.2 Hz, ArC*H*), 7.09 (1 H, d, *J* = 2.1 Hz, ArC*H*), 7.17 - 7.14 (1 H, m, ArC*H*), 7.51 (2 H, d, *J* = 8.9 Hz, ArC*H*), 7.80 - 7.76 (2 H, m, ArC*H*); **δ_C** (126 MHz, $CDCl_3$) 20.6 (*C*H_3), 116.0 (Ar*C*H), 119.0 (Ar*C*), 120.9 (Ar*C*), 124.3 (Ar*C*H), 126.0 (Ar*C*H), 127.1 (Ar*C*H), 128.1 (Ar*C*H), 130.2 (Ar*C*), 130.7 (Ar*C*H), 131.3 (Ar*C*H), 132.2 (Ar*C*), 139.2 (Ar*C*), 140.1 (Ar*C*), 151.1 (Ar*C*); **ν_{max}** (neat)/cm^{-1} 729, 779, 820, 872, 1063, 1181, 1275, 1494, 1579, 2919, 3090, 3520. **HRMS** (ESI): Calcd. for $C_{15}H_{10}OBrS$ $(M-H)^+$, 316.9641; found 316.9633.

4-Bromo-2-(5-bromobenzo[*b*]thiophen-3-yl)phenol 4b

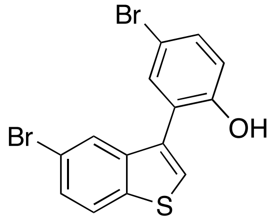

As described in general procedure **B**, 5-bromo-benzo[b]thiophene *S*-oxide **(1a)** (46 mg, 0.2 mmol), 4-bromophenol **(2b)** (52 mg, 0.3 mmol), trifluoroacetic anhydride (41 µl, 0.3 mmol) and *p*TsOH (69 mg, 0.4 mmol), gave **4b** (54 mg, 0.14 mmol, 70%) as a white solid; **m.p**: 115-116 ºC; δ_H (400 MHz, $CDCl_3$) 6.94 (1 H, d, *J* = 8.6 Hz, ArC*H*), 7.41 (1 H, d, *J* = 2.4 Hz, ArC*H*), 5.02 (1 H, s, O*H*), 7.45 (1 H, dd, *J* = 8.6, 2.5 Hz, ArC*H*), 7.49 - 7.55 (2 H, m, ArC*H*), 7.73 (1 H, d, *J* = 1.8 Hz, ArC*H*), 7.79 (1 H, d, *J* = 8.6 Hz, ArC*H*); δ_C (101 MHz, $CDCl_3$) 112.9 (Ar*C*), 118.0 (Ar*C*H), 119.3 (Ar*C*), 123.2 (Ar*C*), 124.4 (Ar*C*H), 125.7 (Ar*C*H), 128.0 (Ar*C*H), 128.4 (Ar*C*H), 130.5 (Ar*C*), 132.9 (Ar*C*H), 133.3 (Ar*C*H), 139.2 (Ar*C*), 139.5 (Ar*C*), 152.6 (Ar*C*); ν_{max} (neat)/cm^{-1} 665, 753, 1063, 1209, 1268, 1392, 1426, 1472, 2923, 3089, 3512; **HRMS** (ESI): Calcd. for $C_{14}H_7OBr_2S$ $(M-H)^+$, 380.8579; found 380.8584.

2-(5-Bromobenzo[*b*]thiophen-3-yl)-4-iodophenol 4c

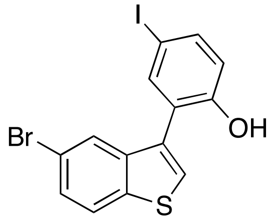

As described in general procedure **B**, 5-bromo-benzo[b]thiophene *S*-oxide **(1a)** (46 mg, 0.2 mmol), 4-iodophenol **(2c)** (66 mg, 0.3 mmol), trifluoroacetic anhydride (41 µl, 0.3 mmol) and TFA (61 µl, 0.8 mmol), gave **4c** (49 mg, 0.112 mmol, 56%) as a white solid; **m.p**: 112-114 ºC; δ_H (500 MHz, $CDCl_3$) 4.97 (s, 1H, O*H*), 6.83 (1 H, d, *J* = 8.6 Hz, ArC*H*), 7.51 - 7.54 (2 H, m, ArC*H*), 7.59 (1 H, d, *J* = 2.2 Hz, ArC*H*), 7.63 (1 H, dd, *J* = 8.6, 2.2 Hz, ArC*H*), 7.72 (1 H, dd, *J* = 1.9, 0.6 Hz, ArC*H*), 7.79 (1 H, dd, *J* = 8.6, 0.6 Hz, ArC*H*); δ_C (126 MHz, $CDCl_3$) 82.7 (Ar*C*), 118.5 (Ar*C*H), 119.3 (Ar*C*), 123.8 (Ar*C*), 124.4 (Ar*C*H), 125.8 (Ar*C*H), 128.0 (Ar*C*H), 128.5 (Ar*C*H), 130.4 (Ar*C*), 138.9 (Ar*C*H), 139.2 (Ar*C*H), 139.6 (Ar*C*), 153.5 (Ar*C*); ν_{max} (neat)/cm^{-1} 753, 791, 1063, 1168, 1209, 1268,

1425, 1471, 1579, 3088, 3509; **HRMS** (ESI): Calcd. for $C_{14}H_7OBrIS$ $(M-H)^+$, 428.8451; found 428.8442.

Methyl 3-(5-bromobenzo[*b*]thiophen-3-yl)-4-hydroxybenzoate 4d

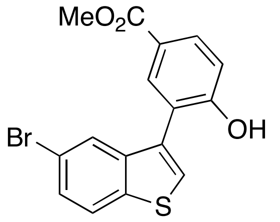

As described in general procedure **B**, 5-bromo-benzo[b]thiophene *S*-oxide (**1a**) (46 mg, 0.2 mmol), methyl 4-hydroxybenzoate (**2d**) (46 mg, 0.3 mmol), trifluoroacetic anhydride (41 µl, 0.3 mmol) and TFA (61 µl, 0.8 mmol), gave **4d** (56 mg, 0.154 mmol, 77%) as a white solid; **m.p**: 177-179ºC; δ_H (500 MHz, $CDCl_3$) 3.89 (3 H, d, *J* = 1.0 Hz, OCH_3), 5.72 (1 H, s, O*H*), 7.09 (1 H, d, *J* = 8.5 Hz, ArC*H*), 7.51 (1 H, dd, *J* = 8.6, 1.7 Hz, ArC*H*), 7.54 (1 H, s, ArC*H*), 7.70 (1 H, d, *J* = 1.7 Hz, ArC*H*), 7.79 (1 H, d, *J* = 8.6 Hz, ArC*H*), 8.00 (1 H, d, *J* = 2.1 Hz, ArC*H*), 8.04 (1 H, dd, *J* = 8.5, 2.0 Hz, ArC*H*); δ_C (126 MHz, $CDCl_3$) 52.2 (OCH_3), 116.2 (Ar*C*H), 119.2 (Ar*C*), 121.3 (Ar*C*), 123.0 (Ar*C*), 124.4 (Ar*C*H), 125.8 (Ar*C*H), 128.0 (Ar*C*H), 128.3 (Ar*C*H), 130.9 (Ar*C*), 132.0 (Ar*C*H), 133.0 (Ar*C*H), 139.2 (Ar*C*), 139.8 (Ar*C*), 157.6 (Ar*C*), 166.8 (C=O); ν_{max} (neat)/cm^{-1} 769, 831, 1067, 1123, 1277, 1403, 1428, 1597, 1687, 3278; **HRMS** (APCI): Calcd. for $C_{16}H_{12}O_3BrS$ $(M+H)^+$, 362.9685; found 362.9682.

Methyl 3-(5-bromobenzo[*b*]thiophen-3-yl)-4-hydroxybenzoate 4e

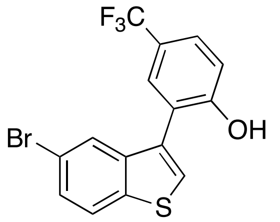

As described in general procedure **B**, 5-bromo-benzo[b]thiophene *S*-oxide (**1a**) (46 mg, 0.2 mmol), 4-(trifluoromethyl)phenol (**2e**) (49 mg, 0.3 mmol), trifluoroacetic anhydride (41 µl, 0.3 mmol) and *p*TsOH (69 mg, 0.4 mmol), gave **4e** (60 mg, 0.16 mmol, 80%) as a white solid; **m.p**: 113-114 ºC; δ_H (400 MHz, $CDCl_3$) 5.31 (1 H, d, *J* = 3.4 Hz, O*H*), 7.15 (1 H, d, *J* = 8.6 Hz, ArC*H*), 7.54 (1 H, dd, *J* = 8.6, 1.8 Hz, ArC*H*), 7.58 (2 H, d, *J* = 3.4 Hz, ArC*H*), 7.63 (1 H, dd, *J* = 8.6, 2.3 Hz, ArC*H*), 7.69 (1 H, dd, *J* = 1.9, 0.5 Hz, ArC*H*), 7.82 (1 H, d, *J* = 8.5 Hz, ArC*H*); δ_C (101 MHz, $CDCl_3$) 116.5 (Ar*C*H), 119.4 (Ar*C*), 121.6 (Ar*C*), 123.5 (q, *J* = 33.0 Hz, Ar*C*), 124.2 (q, *J* = 273 Hz, CF_3), 124.5 (Ar*C*H), 125.6 (Ar*C*H), 127.5 (q, *J* = 3.7 Hz, Ar*C*H), 128.3 (q, *J* = 3.7 Hz, Ar*C*H), 128.4 (Ar*C*H), 128.6 (Ar*C*H), 130.4 (Ar*C*), 139.3 (Ar*C*), 139.5 (Ar*C*), 156.1 (Ar*C*); δ_F (376 MHz, $CDCl_3$) -61.5; ν_{max} (neat)/cm^{-1} 753, 832, 1066, 1108, 1206, 1277, 1316, 1431, 1499, 1587, 1625, 3094, 3446; **HRMS** (ESI): Calcd. for $C_{15}H_7OBrF_3S$ $(M-H)^+$, 370.9359; found 370.9351.

2-(Benzo[*b*]thiophen-3-yl)phenol 4f

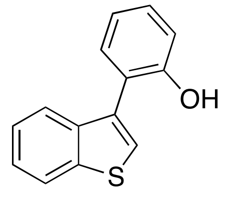

As described in general procedure **C**, benzo[b]thiophene (67 mg, 0.5 mmol), phenol (**2f**) (90 mg, 0.75 mmol), trifluoroacetic anhydride (105 μl, 0.75 mmol) and *p*TsOH (172 mg, 1 mmol), gave **4f** (58 mg, 0.395 mmol, 79 %) as a white solid; **m.p**: 82-83 ºC; δ_H (400 MHz, $CDCl_3$) 5.12 (s, 1H, O*H*), 7.02 - 7.10 (2 H, m, ArC*H*), 7.32 - 7.39 (2 H, m, ArC*H*), 7.46 - 7.39 (2 H, m, ArC*H*), 7.51 (1 H, s, ArC*H*), 7.64 - 7.69 (1 H, m, ArC*H*), 7.93 - 7.98 (1 H, m, ArC*H*); δ_C (101 MHz, $CDCl_3$) 116.0 (Ar*C*H), 120.8 (Ar*C*H), 121.7 (Ar*C*), 123.0 (Ar*C*H), 123.2 (Ar*C*H), 124.8 (Ar*C*H), 125.1 (Ar*C*H), 125.7 (Ar*C*H), 129.9 (Ar*C*H), 130.9 (Ar*C*H), 132.4 (Ar*C*), 138.2 (Ar*C*), 140.7 (Ar*C*), 153.5 (Ar*C*); ν_{max} (neat)/cm^{-1} 729, 752, 839, 1176, 1212, 1345, 1447, 1476, 1576, 3058, 3521; **HRMS** (ESI): Calcd. for $C_{14}H_9OS$ $(M-H)^+$, 225.0369; found 225.0366.

2-(Benzo[*b*]thiophen-3-yl)-4-nitrophenol 4g

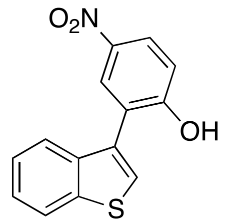

As described in general procedure **C**, benzo[b]thiophene (67 mg, 0.5 mmol), 4-nitrophenol (**2g**) (104 mg, 0.75 mmol), trifluoroacetic anhydride (105 μl, 0.75 mmol) and *p*TsOH (172 mg, 1 mmol), gave **4g** (115 mg, 0.375 mmol, 85 %) as a yellow solid; **m.p**: 122-123 ºC; δ_H (500 MHz, $CDCl_3$) 5.84 (1 H, s, O*H*), 7.16 (1 H, d, *J* = 8.9 Hz, ArC*H*), 7.42 - 7.51 (2 H, m, ArC*H*), 7.57 - 7.62 (2 H, m, ArC*H*), 7.95 - 8.01 (1 H, m, ArC*H*), 8.23 - 8.30 (2 H, m, ArC*H*); δ_C (126 MHz, $CDCl_3$) 116.53 (Ar*C*H), 122.43 (Ar*C*), 122.61 (Ar*C*H), 123.33 (Ar*C*H), 125.35 (Ar*C*H), 125.64 (Ar*C*H), 125.94 (Ar*C*H), 127.02 (Ar*C*H), 127.34 (Ar*C*H), 129.61 (Ar*C*), 137.35 (Ar*C*H), 140.84 (Ar*C*), 141.71 (Ar*C*), 159.04 (Ar*C*); ν_{max} (neat)/cm^{-1} 752, 763, 1082, 1274, 1328, 1480, 1534, 1581, 3095, 3336; **HRMS** (ESI): Calcd. for $C_{14}H_8O_3NS$ $(M-H)^+$, 270.0230; found 270.0220.

[3-(Benzo[*b*]thiophen-3-yl)-4-hydroxyphenyl](phenyl)methanone 4h

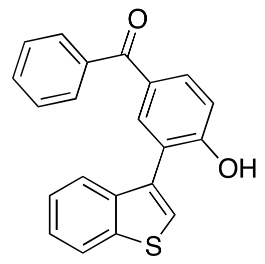

As described in general procedure **C**, benzo[b]thiophene (67 mg, 0.5 mmol), 4-hydroxybenzophenone (**2h**) (148 mg, 0.75 mmol), trifluoroacetic anhydride (105 μl, 0.75 mmol) and *p*TsOH (172 mg, 1 mmol), gave **4h** (152 mg, 0.46 mmol, 92 %) as a white solid; **m.p**: 180-183 ºC; δ_H (400 MHz, $CDCl_3$) 5.68 - 5.75 (1 H, m, O*H*), 7.14 - 7.17 (1 H, m, ArC*H*), 7.42 - 7.44 (2 H, m, ArC*H*), 7.47 (2 H, t, *J* = 7.6 Hz, ArC*H*), 7.56 (2 H, d, *J* =

5.9 Hz, ArC*H*), 7.64 (1 H, dd, *J* = 6.9, 2.1 Hz, ArC*H*), 7.79 - 7.82 (2 H, m, ArC*H*), 7.86 - 7.89 (2 H, m, ArC*H*), 7.95 (1 H, dd, *J* = 6.9, 2.0 Hz, ArC*H*); δ_C (101 MHz, $CDCl_3$) 115.9 (Ar*C*H), 121.8 (Ar*C*), 123.0 (Ar*C*H), 123.2 (Ar*C*H), 125.0 (Ar*C*H), 125.3 (Ar*C*H), 126.5 (Ar*C*H), 128.4 (Ar*C*H), 129.9 (Ar*C*H), 130.5 (Ar*C*), 131.1 (Ar*C*), 132.2 (Ar*C*H), 132.7 (Ar*C*H), 133.8 (Ar*C*H), 137.8 (Ar*C*), 138.2 (Ar*C*), 140.8 (Ar*C*), 157.5 (Ar*C*), 195.6 (C=O); ν_{max} (neat)/cm^{-1} 701, 718, 835, 922, 1112, 1232, 1273, 1428, 1552, 1626, 3068; **HRMS** (APCI): Calcd. for $C_{21}H_{15}O_2S$ $(M+H)^+$, 331.0787; found 331.0785.

2-(Benzo[*b*]thiophen-3-yl)-6-bromophenol 4i

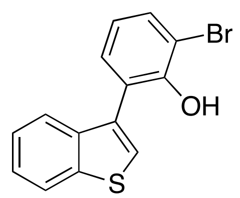

As described in general procedure **C**, benzo[b]thiophene (67 mg, 0.5 mmol), 2-bromophenol (**2i**) (130 mg, 0.75 mmol), trifluoroacetic anhydride (105 µl, 0.75 mmol) and *p*TsOH (172 mg, 1 mmol), gave **4i** (78 mg, 0.255 mmol, 51 %) as a colourless oil; $\boldsymbol{\delta_H}$ (400 MHz, $CDCl_3$) 5.66 (s, 1H, O*H*), 6.93 (1 H, t, *J* = 7.7 Hz, ArC*H*), 7.34 (1 H, dd, *J* = 7.7, 1.6 Hz, ArC*H*), 7.34 - 7.46 (2 H, m, ArC*H*), 7.52 (1 H, s, ArC*H*), 7.56 (1 H, dd, *J* = 8.0, 1.6 Hz, ArC*H*), 7.64 - 7.69 (1 H, m, ArC*H*), 7.91 - 7.97 (1 H, m, ArC*H*); $\boldsymbol{\delta_C}$ (101 MHz, $CDCl_3$) 110.9 (Ar*C*), 121.7 (Ar*C*H), 123.0 (Ar*C*H), 123.3 (Ar*C*H), 123.6 (Ar*C*), 124.6 (Ar*C*H), 124.8 (Ar*C*H), 125.8 (Ar*C*H), 130.8 (Ar*C*H), 132.3 (Ar*C*H), 132.5 (Ar*C*), 138.2 (Ar*C*), 140.3 (Ar*C*), 150.2 (Ar*C*); ν_{max} (neat)/cm^{-1} 731, 840, 953, 1112, 1162, 1215, 1234, 1318, 1438, 3055, 3447; **HRMS** (ESI): Calcd. for $C_{14}H_8OBrS$ $(M-H)^+$, 302.9485; found 302.9477.

2-(Benzo[*b*]thiophen-3-yl)-6-nitrophenol 4j

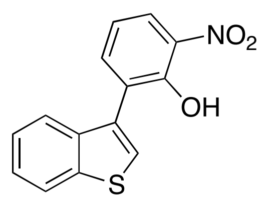

As described in general procedure **C**, benzo[b]thiophene (67 mg, 0.5 mmol), 2-nitrophenol (**2j**) (104 mg, 0.75 mmol), trifluoroacetic anhydride (105 µl, 0.75 mmol) and *p*TsOH (172 mg, 1 mmol), gave **4j** (102 mg, 0.375 mmol, 75 %) as a yellow solid; **m.p**: 122-123 ºC; $\boldsymbol{\delta_H}$ (400 MHz, $CDCl_3$) 7.11 - 7.44 (1 H, dd, *J* = 8.5, 7.4 Hz, ArC*H*), 7.36 (2 H, m, ArC*H*), 7.57 (1H, s, ArC*H*), 7.62 - 7.65 (1 H, m, ArC*H*), 7.75 (1 H, dd, *J* = 7.4, 1.6 Hz, ArC*H*), 7.91 - 7.97 (1 H, m, ArC*H*), 8.21 (1 H, dd, *J* = 8.5, 1.6 Hz, ArC*H*), 11.07 (1 H, s, O*H*); $\boldsymbol{\delta_C}$ (101 MHz, $CDCl_3$) 119.9 (Ar*C*H), 123.0 (Ar*C*H), 123.1 (Ar*C*H), 124.5 (Ar*C*H), 124.7 (Ar*C*H), 124.8 (Ar*C*H), 126.5 (Ar*C*H), 127.5 (Ar*C*), 131.2 (Ar*C*), 134.4 (Ar*C*), 138.1 (Ar*C*), 139.1 (Ar*C*H), 140.0 (Ar*C*), 153.4 (Ar*C*); $\boldsymbol{\nu_{max}}$ (neat)/cm^{-1} 733, 755, 1217, 1276, 1442, 1540, 1603, 3125; **HRMS** (ESI): Calcd. for $C_{14}H_8O_3NS$ $(M-H)^+$, 270.0230; found 270.0221.

3-(Benzo[*b*]thiophen-3-yl)-*N*,*N*-diethyl-2-hydroxybenzamide 4k

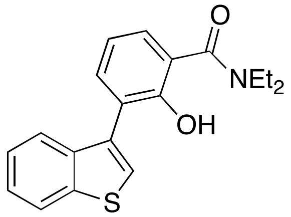

As described in general procedure **C**, benzo[b]thiophene (67 mg, 0.5 mmol), *N*,*N*-diethyl-2-hydroxybenzamide (**2k**) (145 mg, 0.75 mmol), trifluoroacetic anhydride (105 μl, 0.75 mmol) and *p*TsOH (172 mg, 1 mmol), gave **4k** (90 mg, 0.275 mmol, 55 %) as a yellow oil; δ_H (400 MHz, $CDCl_3$) 1.31 (6 H, t, *J* = 7.1 Hz, CH_3), 3.57 (4 H, q, *J* = 7.1 Hz, CH_2), 6.98 (1 H, t, *J* = 7.6 Hz, ArC*H*), 7.33 - 7.39 (3H, m, ArC*H*), 7.46 (1 H, dd, *J* = 7.6, 1.6 Hz, ArC*H*), 7.52 (1 H, s, ArC*H*), 7.70 - 7.74 (1 H, m, ArC*H*), 7.89 - 7.94 (1 H, m, ArC*H*), 9.60 (1 H, s, O*H*); δ_C (101 MHz, $CDCl_3$) 13.6 (CH_3), 42.3 (CH_2), 118.6 (Ar*C*H), 119.4 (Ar*C*), 122.8 (Ar*C*H), 123.5 (Ar*C*H), 124.2 (Ar*C*H), 124.4 (Ar*C*), 124.9 (Ar*C*H), 125.4 (Ar*C*H), 127.2 (Ar*C*H), 133.2 (Ar*C*), 133.7 (Ar*C*H), 138.5 (Ar*C*), 140.2 (Ar*C*), 155.9 (Ar*C*), 171.4 (C=O); ν_{max} (neat)/cm^{-1} 729, 760, 908, 1117, 1250, 1345, 1431, 1573, 1599, 2973; **HRMS** (ESI+): Calcd. for $C_{19}H_{20}O_2NS$ $(M+H)^+$, 326.1209; found 326.1201.

2-(5-Bromobenzo[*b*]thiophen-3-yl)-5-chlorophenol 4l

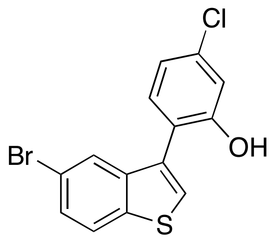

As described in general procedure **B**, 5-bromo-benzo[b]thiophene *S*-oxide (**1a**) (46 mg, 0.2 mmol), 3-chlorophenol (**2l**) (38 mg, 0.3 mmol), trifluoroacetic anhydride (41 μl, 0.3 mmol) and I_2 (102 mg, 0.4 mmol), gave **4l** (45 mg, 0.134 mmol, 67%) as a white solid; **m.p**: 94-96 ºC; δ_H (500 MHz, $CDCl_3$) 5.13 (1 H, s, O*H*), 7.04 (1 H, dd, *J* = 8.1, 2.1 Hz, ArC*H*), 7.08 (1 H, d, *J* = 2.1 Hz, ArC*H*), 7.22 (1 H, d, *J* = 8.1 Hz, ArC*H*), 7.51 - 7.54 (2 H, m, ArC*H*), 7.73 (1 H, d, *J* = 1.8 Hz, ArC*H*), 7.79 (1 H, d, *J* = 8.7 Hz, ArC*H*); δ_C (126 MHz, $CDCl_3$) 116.6 (Ar*C*H), 119.3 (Ar*C*), 119.7 (Ar*C*), 121.3 (Ar*C*H), 124.4 (Ar*C*H), 125.8 (Ar*C*H), 127.7 (Ar*C*H), 128.4 (Ar*C*H), 130.8 (Ar*C*), 131.7 (Ar*C*H), 135.4 (Ar*C*), 139.3 (Ar*C*), 139.8 (Ar*C*), 154.1 (Ar*C*); ν_{max} (neat)/cm^{-1} 752, 785, 892, 1065, 1173, 1211, 1298, 1477, 1567, 3089, 3513; **HRMS** (ESI): Calcd. for $C_{14}H_7OBrClS$ $(M-H)^+$, 336.9084; found 336.9091.

(8*R*,9*S*,13*S*,14*S*)-2-(5-Bromobenzo[*b*]thiophen-3-yl)-3-hydroxy-13-methyl-7,8,9,11,12,13,15,16-octahydro-6*H*-cyclopenta[*a*]phenanthren-17(14*H*)-one 4m

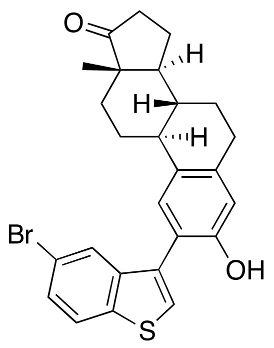

As described in general procedure **B**, 5-bromo-benzo[b]thiophene *S*-oxide (**1a**) (23 mg, 0.1 mmol), estrone (**2m**) (60 mg, 0.125 mmol), trifluoroacetic anhydride (20 µl, 0.15 mmol) and I_2 (51 mg, 0.2 mmol), gave **4m** (33 mg, 0.069 mmol, 69%) as a white solid; **m.p**: 136-138 ºC; δ_H (400 MHz, $CDCl_3$) 0.93 (3 H, s, C*H*$_3$), 1.38 - 1.73 (6 H, m), 1.90 - 1.96 (1 H , m), 2.02 - 2.21 (3 H, m), 2.30 - 2.38 (2 H, m), 2.52 (1 H, dd, *J* = 18.6, 8.7 Hz), 2.96 (2 H, dd, *J* = 9.1, 4.2 Hz), 4.94 (1 H, s, O*H*), 6.80 (1 H, s, ArC*H*), 7.19 (1 H, d, *J* = 1.0 Hz, ArC*H*), 7.47 (1 H, s, ArC*H*), 7.50 (1 H, dd, *J* = 8.6, 1.9 Hz, ArC*H*), 7.80 - 7.76 (2H, m, ArC*H*); δ_C (101 MHz, $CDCl_3$) 14.0 (CH_3), 21.7 (CH_2), 26.1 (CH_2), 26.6 (CH_2), 29.5 (CH_2), 31.6 (CH_2), 36.0 (CH_2), 38.4 (CH), 44.1 (CH), 48.1 (*C*), 50.5 (CH), 116.0 (Ar*C*H), 118.8 (Ar*C*), 119.1 (Ar*C*), 124.3 (Ar*C*H), 126.1 (Ar*C*H), 127.1 (Ar*C*H), 128.0 (Ar*C*H), 128.1 (Ar*C*H), 132.5 (Ar*C*), 138.9 (Ar*C*), 139.2 (Ar*C*), 140.2 (Ar*C*), 151.3 (Ar*C*), 221.2 (Ar*C*); ν_{max} (neat)/cm^{-1} 749, 871, 1063, 1214, 1404, 1721, 2927, 3346; **HRMS** (ESI): Calcd. for $C_{26}H_{24}O_2BrS$ $(M-H)^+$, 479.0686; found 579.0675.

2-(6-Bromobenzo[*b*]thiophen-3-yl)phenol 4n

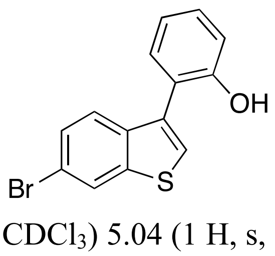

As described in general procedure **C**, 6-bromo-benzo[b]thiophene (106 mg, 0.5 mmol), phenol (**2f**) (71 mg, 0.75 mmol), trifluoroacetic anhydride (105 µl, 0.75 mmol) and *p*TsOH (172 mg, 1 mmol), gave **4n** (109 mg, 0.36 mmol, 72 %) as a colourless oil; δ_H (400 MHz, $CDCl_3$) 5.04 (1 H, s, O*H*), 7.01 - 7.08 (2 H, m, ArC*H*), 7.30 (1 H, dd, *J* = 7.5, 1.7 Hz, ArC*H*), 7.34 - 7.37 (1 H, m, ArC*H*), 7.46 - 7.51 (3 H, m, ArC*H*), 8.06 - 8.10 (1H, m, ArC*H*); δ_C (101 MHz, $CDCl_3$) 116.1 (Ar*C*H), 119.2 (Ar*C*), 120.9 (Ar*C*H), 121.2 (Ar*C*), 124.5 (Ar*C*H), 125.5 (Ar*C*H), 126.0 (Ar*C*H), 128.2 (Ar*C*H), 130.1 (Ar*C*H), 131.0 (Ar*C*H), 132.3 (Ar*C*), 137.1 (Ar*C*), 142.1 (Ar*C*), 153.4 (Ar*C*); ν_{max} (neat)/cm^{-1} 746, 887, 1069, 1206, 1286, 1470, 1560, 3080, 3520; **HRMS** (ESI): Calcd. for $C_{14}H_8OBrS$ $(M-H)^+$, 302.9474; found 302.9477.

2-(5-Bromobenzo[*b*]thiophen-3-yl)phenol 4o

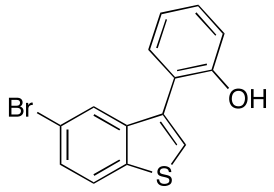

As described in general procedure **B**, 5-bromo-benzo[b]thiophene *S*-oxide (46 mg, 0.2 mmol), phenol (**2f**) (29 mg, 0.3 mmol), trifluoroacetic anhydride (41 µl, 0.3 mmol) and *p*TsOH (69 mg, 0.4 mmol), gave **4o** (44 mg, 0.145 mmol, 72 %) as a colourless oil; $\boldsymbol{\delta_H}$ (400 MHz, $CDCl_3$) 5.03 (1 H, s, O*H*), 7.04 - 7.10 (2 H, m, ArC*H*), 7.29 - 7.33 (1 H, m, ArC*H*), 7.35 - 7.40 (1 H, m, ArC*H*), 7.50 - 7.56 (2 H, m, ArC*H*), 7.78 (1 H, d, *J* = 1.5 Hz, ArC*H*), 7.80 (1 H, d, *J* = 8.5 Hz, ArC*H*); $\boldsymbol{\delta_C}$ (101 MHz, $CDCl_3$) 116.1 (Ar*C*H), 119.0 (Ar*C*Br), 120.9 (Ar*C*H), 121.0 (Ar*C*), 124.2 (Ar*C*H), 125.9 (Ar*C*H), 127.1 (Ar*C*H), 128.1 (Ar*C*H), 130.0 (Ar*C*H), 130.8 (Ar*C*H), 131.8 (Ar*C*), 139.1 (Ar*C*), 140.0 (Ar*C*), 153.3 (Ar*C*OH); $\boldsymbol{\nu_{max}}$ (neat)/cm^{-1} 753, 789, 906, 1064, 1177, 1211, 1420, 1478, 1578, 2849, 2919, 3087, 3520; **HRMS** (ESI): Calcd. for $C_{14}H_8OSBr$ $(M-H)^+$, 302.9484; found 302.9484.

2-(5-Chlorobenzo[b]thiophen-3-yl)phenol 4p

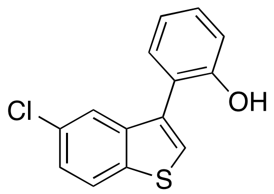

As described in general procedure **C**, 5-chloro-benzo[b]thiophene *S*-oxide (85 mg, 0.5 mmol), phenol (**2f**) (73 mg, 0.75 mmol), trifluoroacetic anhydride (105 µl, 0.75 mmol) and *p*TsOH (172 mg, 1 mmol), gave **4p** (77 mg, 0.3 mmol, 60 %) as a colourless oil; $\boldsymbol{\delta_H}$ (400 MHz, $CDCl_3$) 5.07 (1 H, s, O*H*), 7.04 - 7.10 (2 H, m, ArC*H*), 7.29 - 7.42 (3 H, m, ArC*H*), 7.56 (1 H, s, ArC*H*), 7.63 (1 H, d, *J* = 2.0 Hz, ArC*H*), 7.85 (1 H, d, *J* = 8.5 Hz, ArC*H*); δ_C (101 MHz, $CDCl_3$) 115.7 (Ar*C*H), 120.5 (Ar*C*H), 120.7 (Ar*C*), 122.5 (Ar*C*H), 123.5 (Ar*C*H), 125.1 (Ar*C*H), 126.9 (Ar*C*H), 129.6 (Ar*C*H), 130.5 (Ar*C*H), 130.8 (Ar*C*), 131.5 (Ar*C*Cl), 138.3 (Ar*C*), 139.2 (Ar*C*), 152.9 (Ar*C*OH); $\boldsymbol{\nu_{max}}$ (neat)/cm^{-1} 753, 832,906, 1075, 1172, 1212, 1277, 1423, 1479, 1579, 3087, 3525; **HRMS** (ESI): Calcd. for $C_{14}H_{10}OSCl$ $(M+H)^+$, 261.0135; found 261.0134.

2-(5-Nitrobenzo[*b*]thiophen-3-yl)phenol 4q

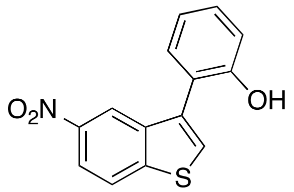

As described in general procedure **B**, 5-nitro-benzo[b]thiophene *S*-oxide (**1b**) (39 mg, 0.2 mmol), phenol (**2f**) (28 mg, 0.3 mmol), trifluoroacetic anhydride (41 µl, 0.3 mmol) and *p*TsOH (69 mg, 0.4 mmol), gave **4q** (50 mg, 0.186 mmol, 93%) as a yellow solid; **m.p**: 202-204 ºC; $\boldsymbol{\delta_H}$ (500 MHz, $CDCl_3$) 5.00 (s, 1H, O*H*), 7.03 - 7.14 (2 H, m, ArC*H*), 7.31 - 7.42 (2 H, m, ArC*H*), 7.69 (1 H, s, ArC*H*), 8.04 (1 H, d, *J* = 8.8 Hz, ArC*H*), 8.23 - 8.29 (1 H, m, ArC*H*), 8.54 (1 H, d, *J* = 2.3 Hz, ArC*H*); $\boldsymbol{\delta_C}$ (126 MHz, $CDCl_3$) 116.5 (Ar*C*H), 119.3 (Ar*C*H),

119.5 (Ar*C*H), 120.6 (Ar*C*), 121.4 (Ar*C*H), 123.6 (Ar*C*H), 128.6 (Ar*C*H), 130.5 (Ar*C*H), 131.2 (Ar*C*H), 134.3 (Ar*C*), 138.6 (Ar*C*), 145.9 (Ar*C*), 146.3 (Ar*C*), 153.2 (Ar*C*); ν_{max} (neat)/cm^{-1} 738, 754, 1052, 1097, 1184, 1284, 1325, 1451, 1484, 1529, 3093, 3427; **HRMS** (APCI): Calcd. for $C_{14}H_{10}O_3NS$ $(M+H)^+$, 272.0376; found 272.0373.

2-(4-Bromobenzo[*b*]thiophen-3-yl)phenol 4r

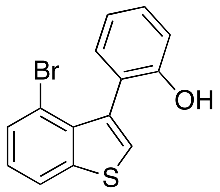

As described in general procedure **C**, 4-bromo-benzo[b]thiophene (106 mg, 0.5 mmol), phenol (**2f**) (71 mg, 0.75 mmol), trifluoroacetic anhydride (105 µl, 0.75 mmol) and I_2 (500 mg, 1 mmol), gave **4r** (104 mg, 0.35 mmol, 70 %) as a white solid; **m.p**: 120-122 ºC; δ_H (400 MHz, $CDCl_3$) 4.75 (s, 1H, O*H*), 6.95 - 7.01 (2 H, m, ArC*H*), 7.21 - 7.26 (2 H, m, ArC*H*), 7.35 (1 H, td, *J* = 8.0, 1.7 Hz, ArC*H*), 7.51 (1 H, s, ArC*H*), 7.58 (1 H, dd, *J* = 7.6, 1.0 Hz, ArC*H*), 7.90 (1 H, dd, *J* = 8.0, 1.0 Hz, ArC*H*); δ_C (101 MHz, $CDCl_3$) 115.2 (Ar*C*H), 117.5 (Ar*C*), 120.2 (Ar*C*H), 122.5 (Ar*C*H), 123.7 (Ar*C*), 125.8 (Ar*C*H), 129.1 (Ar*C*H), 130.1 (Ar*C*H), 130.3 (Ar*C*H), 131.9 (Ar*C*H), 132.8 (Ar*C*), 135.53 (Ar*C*), 142.6 (Ar*C*), 154.5 (Ar*C*); ν_{max} (neat)/cm^{-1} 753, 792, 844, 1074, 1100, 1199, 1227, 1322, 1438, 1579, 3260; **HRMS** (ESI): Calcd. for $C_{14}H_8OBrS$ $(M-H)^+$, 302.9485; found 302.9478.

2-(7-Bromobenzo[*b*]thiophen-3-yl)phenol 4s

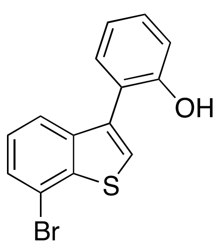

As described in general procedure **C**, 7-bromo-benzo[b]thiophene *S*-oxide (106 mg, 0.5 mmol), phenol (**2f**) (71 mg, 0.75 mmol), trifluoroacetic anhydride (105 µl, 0.75 mmol) and *p*TsOH (172 mg, 1 mmol), gave **4s** (84 mg, 0.275 mmol, 55%) as a colourless oil; δ_H (500 MHz, $CDCl_3$) 5.04 (1 H, s, O*H*), 7.01 - 7.08 (2 H, m, ArC*H*), 7.26 - 7.33 (2 H, m, ArC*H*), 7.35 - 7.37 (1 H, m, ArC*H*), 7.56 - 7.63 (3 H, m, ArC*H*); δ_C (126 MHz, $CDCl_3$) 116.1 (Ar*C*H), 116.5 (Ar*C*), 120.9 (Ar*C*H), 121.7 (Ar*C*), 122.3 (Ar*C*H), 126.2 (Ar*C*H), 126.6 (Ar*C*H), 127.9 (Ar*C*H), 130.1 (Ar*C*H), 131.0 (Ar*C*H), 133.5 (Ar*C*), 139.4 (Ar*C*), 142.4 (Ar*C*), 153.3 (Ar*C*); ν_{max} (neat)/cm^{-1} 722, 752, 782, 1080, 1176, 1213, 1343, 1383, 1475, 1575, 2923, 3064, 3525; **HRMS** (APCI): Calcd. for $C_{14}H_{10}OBrS$ $(M+H)^+$, 304.9630; found 304.9629.

6H-Benzo[4,5]thieno[2,3-c]chromen-6-one[2] 4t

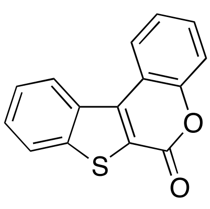

As described in general procedure **B**, methyl benzo[*b*]thiophene-2-carboxylate 1-oxide (24.8 mg, 0.12 mmol), phenol (**2f**) (16.9 mg, 0.18 mmol), trifluoroacetic anhydride (105 µl, 0.75 mmol) and I_2 (61 mg, 0.24 mmol), gave **4t** (22.1 mg, 8.2 mmol, 68%) as a white solid; **m.p:** 199-200 ºC; **δ_H** (500 MHz, $CDCl_3$) 7.44-7-47 (1 H, m, ArC*H*), 7.55 (2 H, m, ArC*H*), 8.03 (1 H, m, ArC*H*), 7.64 (2 H, m, ArC*H*), 8.50 (1 H, m, ArC*H*), 8.64 (1 H, m, ArC*H*); **δ_C** (126 MHz, $CDCl_3$) 118.1 (Ar*C*H), 118.4 (Ar*C*), 123.5 (Ar*C*H), 124.0 (Ar*C*H), 124.8 (Ar*C*H), 125.7 (Ar*C*H), 126.1 (Ar*C*H), 126.2 (Ar*C*), 128.4 (Ar*C*H), 130.0 (Ar*C*H), 135.0 (Ar*C*), 138.6 (Ar*C*), 143.7 (Ar*C*), 152.7 (Ar*C*), 158.0 (*C*=O); **ν_{max}** (neat)/cm^{-1} 674, 736, 943, 1028, 1161, 1272, 1374, 1441, 1590, 1718, 2923, 3058. **HRMS** (HESI): Calcd. for $C_{15}H_9O_2S$ $(M+H)^+$, 253.0318; found 253.0308.

2-(2-Phenylbenzo[b]thiophen-3-yl)phenol 4u

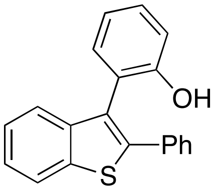

As described in general procedure **B**, 2-phenylbenzo[b]thiophene 1-oxide (25.7 mg, 0.11 mmol), phenol (**2f**) (15.5 mg, 0.17 mmol), trifluoroacetic anhydride (105 µl, 0.75 mmol) and *p*TsOH (38 mg, 0.22 mmol), gave **4u** (10.8 mg, 3.6 mmol, 40%) as a colourless oil; **δ_H** (500 MHz, $CDCl_3$) 4.91 (1 H, s, OH), 6-97-7.03 (2 H, m, ArC*H*), 7.20-7.23 (1 H, m, ArC*H*), 7.28 (3 H, m, ArC*H*), 7.37 (5 H, m, ArC*H*), 7.45 (1 H, d, *J* = 7.8 Hz, ArC*H*), 7.91 (1 H, d, *J* = 7.8 Hz, ArC*H*); **δ_C** (126 MHz, $CDCl_3$) 115.8 (Ar*C*H), 120.8 (Ar*C*H), 121.3 (Ar*C*), 122.1 (Ar*C*H), 123.2 (Ar*C*H), 124.8 (Ar*C*H), 125.0 (Ar*C*H), 126.9 (Ar*C*), 128.3 (Ar*C*H), 128.5 (Ar*C*H), 128.6 (Ar*C*H), 129.8 (Ar*C*H), 131.3 (Ar*C*H), 133.3 (Ar*C*), 139.0 (Ar*C*), 140.5 (Ar*C*), 141.9 (Ar*C*), 153.4 (Ar*C*); **ν_{max}** (neat)/cm^{-1} 3524, 3057, 693, 732, 751, 1068, 1192, 1286, 1331, 1492, 1578, 2924; **HRMS** (HESI): Calcd. for $C_{20}H_{13}OS$ $(M-H)^+$, 301.0682; found 301.0682.

General Procedure D. Propargylation/Allyation of benzo[b]thiophene *S*-oxides

To an oven dried tube under nitrogen was added the benzo[b]thiophene *S*-oxide (**1**) (0.2 mmol), propargyl (**7**) or allyl silane (**6**) (0.3 mmol) and MeCN (2 mL). The solution was cooled to 0 °C, and TFAA was added (0.4 mmol). The cooling bath was then removed and the mixture stirred at ambient temperature overnight. Saturated $NaHCO_3$ (aq) (3 mL) was added and the aqueous layer was extracted with EtOAc (3 × 5 mL). The combined organic extracts dried with $MgSO_4$. The solvent was removed *in vacuo* before purification by column chromatography (hexane) to give the product.

General Procedure E. Propargylation of Benzo[b]thiophenes -*in situ* oxidation and coupling.

To an oven dried vial under nitrogen, was added the benzo[b]thiophene (0.5 mmol) CH_2Cl_2 (1 mL) and TFA (1 mL). To this mixture was added H_2O_2 (30% aq, 0.5 mmol) at room temperature. The reaction was monitored by TLC (5% EtOAc in $CHCl_3$) and more H_2O_2 was added until complete consumption of the starting material was observed. The reaction was then quenched with $NaHCO_3$ at 0 °C and the aqueous layer extracted with CH_2Cl_2 (3 × 1 mL). The combined organic layers were dried with $MgSO_4$ before MeCN was added (10 mL). CH_2Cl_2 was removed under vacuum with no heating to give a solution of benzo[b]thiophene *S*-oxide (**1**) in MeCN to which was added the silane (**6** or **7**) (0.75 mmol) and TFAA (1 mmol) at 0 °C. The cooling bath was then removed and the mixture stirred at ambient temperature overnight. Saturated $NaHCO_3$ (aq) (3 mL) was added and the aqueous layer was extracted with EtOAc (3 × 5 mL). The combined organic extracts dried with $MgSO_4$. The solvent was removed *in vacuo* before purification by column chromatography (hexane) to give the product.

C3 allylation/propargylation. Characterization data.

3-Allyl-5-bromobenzo[b]thiophene 6a

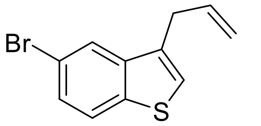

As described in general procedure **D**, 5-bromo-benzo[b]thiophene *S*-oxide (**1a**) (46 mg, 0.2 mmol), trimethyl(2-methylallyl)silane (**5a**) (47 µL, 0.3 mmol) and trifluoroacetic anhydride (56 µL, 0.4 mmol), gave **6a** (40 mg, 0.16 mmol, 79%) as a colourless oil; **δ_H** (400 MHz, $CDCl_3$) 3.57 (2 H, dq, *J* = 6.5, 1.3 Hz, C*H*$_2$), 5.15 - 5.22 (2 H, m, CH=C*H*$_2$), 6.00 - 6.11 (1 H, m, C*H*=CH$_2$), 7.17 (1 H, s, ArC*H*), 7.45 (1 H, dd, *J* = 8.5, 1.8 Hz, ArC*H*), 7.71 (1 H, d, *J* = 8.5 Hz, ArC*H*), 7.89 (1 H, d, *J* = 2.0 Hz, ArC*H*); **δ_C** (101 MHz, $CDCl_3$) 32.8 (*C*H$_2$), 117.0 (CH=*C*H$_2$), 118.1 (Ar*C*Br), 123.9 (Ar*C*H), 124.1 (Ar*C*H), 124.7 (Ar*C*H), 127.2 (Ar*C*H), 134.0 (Ar*C*), 135.0 (*C*H=CH$_2$), 139.1 (Ar*C*), 140.5 (Ar*C*); **ν_{max}** (neat)/cm^{-1} 730, 77, 815, 992, 1062, 1149, 1247, 1418, 1429, 1579, 1638; **HRMS** (EI): Calcd. for $C_{11}H_9SBr$ $(M)^+$, 251.9603; found 251.9611.

5-Bromo-3-(2-methylallyl)benzo[b]thiophene 6b

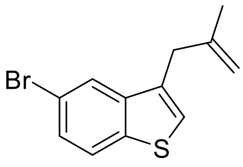

As described in general procedure **D**, 5-bromo-benzo[b]thiophene *S*-oxide (**1a**) (46 mg, 0.2 mmol), trimethyl(2-methylallyl)silane (**5b**) (52 μL, 0.3 mmol) and trifluoroacetic anhydride (56 μL, 0.4 mmol), gave **6b** (47 mg, 0.17 mmol, 88%) as a colourless oil; **δ_H** (400 MHz, $CDCl_3$) 1.77 (3 H, s, C*H*3), 3.53 (2 H, s, C*H*2), 4.78 (1 H, s, C=C*H*2), 4.91 (1 H, s, C=C*H*2), 7.18 (1 H, s, ArC*H*), 7.44 (1 H, dd, *J* = 8.5, 1.8 Hz, ArC*H*), 7.71 (1 H, d, *J* = 8.5 Hz, ArC*H*), 7.89 (1 H, d, *J* = 1.8 Hz, ArC*H*); δ_C (101 MHz, $CDCl_3$) 22.3 (*C*H_3), 37.1 (*C*H_2), 112.7 (C=*C*H_2), 118.0 (Ar*C*Br), 124.1 (Ar*C*H), 124.6 (Ar*C*H), 124.9 (Ar*C*H), 127.1 (Ar*C*H), 133.5 (Ar*C*), 139.1 (Ar*C*), 140.8 (Ar*C*), 142.9 (*C*=CH_2); ν_{max} (neat)/cm^{-1} 773, 866, 892, 1062, 1248, 1373, 1417, 1578, 1650, 2903, 2969; **HRMS** (EI): Calcd. for $C_{12}H_{11}SBr$ $(M)^+$, 265.97.59; found 265.9770.

5-Bromo-3-[2-(chloromethyl)allyl]benzo[b]thiophene 6c

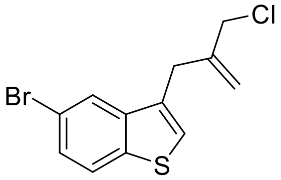

As described in general procedure **D**, 5-bromo-benzo[b]thiophene *S*-oxide (**1a**) (46 mg, 0.2 mmol), [2-(chloromethyl)allyl]trimethylsilane (**5c**) (54 μL, 0.3 mmol) and trifluoroacetic anhydride (56 μL, 0.4 mmol), gave **6c** (52 mg, 0.17 mmol, 85%) as a colourless oil: **δ_H** (400 MHz, $CDCl_3$) 3.51 (2 H, s, C*H*2Cl), 3.82 (2 H, d, *J* = 0.8 Hz, C*H*2), 4.79 (1 H, q, *J* = 1.3 Hz, C=C*H*2), 5.05 (1 H, d, *J* = 0.8 Hz, C=C*H*2), 7.04 (1 H, s, ArC*H*), 7.23 (1 H, dd, *J* = 8.5, 1.8 Hz, ArC*H*), 7.50 (1 H, d, *J* = 8.5 Hz, ArC*H*), 7.65 (1 H, d, *J* = 2.0 Hz, ArC*H*); **δ_C** (101 MHz, $CDCl_3$) 32.4 (*C*H_2), 47.7 (*C*H_2Cl), 117.0 (C=*C*H_2), 118.2 (Ar*C*Br), 124.1 (Ar*C*H), 124.8 (Ar*C*H), 125.5 (Ar*C*H), 127.4 (Ar*C*H), 132.0 (Ar*C*), 139.2 (Ar*C*), 140.4 (Ar*C*), 142.5 (*C*=CH_2); **ν_{max}** (neat)/cm^{-1} 687, 730, 778, 905, 1062, 1149, 1257, 1418, 1579, 1645; **HRMS** (EI): Calcd. for $C_{12}H_{10}SBr$ $(M)^+$, 299.9370; found 299.9374.

2-[(5-Bromobenzo[b]thiophen-3-yl)methyl]allyl acetate 6d

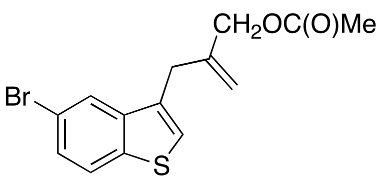

As described in general procedure **D**, 5-bromo-benzo[b]thiophene *S*-oxide (**1a**) (46 mg, 0.2 mmol), 2-((trimethylsilyl)methyl)allyl acetate (56 mg, 0.3 mmol) and trifluoroacetic anhydride (56 μL, 0.4 mmol), gave **6d** (50 mg, 0.17 mmol, 80%) as a colourless oil; **δ_H** (400 MHz, $CDCl_3$) 2.10 (3 H, s, C*H*3), 3.61 (2 H, s, C*H*2), 4.57 (2 H, s, C*H*2), 5.00 (1 H, s, C=C*H*2), 5.21 (1 H, s, C=C*H*2), 7.22 (1 H, s, ArC*H*), 7.45 (1 H, dd, *J* = 8.5, 1.8 Hz, ArC*H*), 7.71 (1 H, d, *J* = 8.5 Hz, ArC*H*), 7.86 (1 H, d, *J* = 1.8

Hz, ArC*H*); **δ**$_C$ (101 MHz, $CDCl_3$) 20.9 (CH_3), 32.6 (CH_2), 66.5 (CH_2), 115.5 (C=CH_2), 118.2 (Ar*C*Br), 124.1 (Ar*C*H), 124.8 (Ar*C*H), 125.2 (Ar*C*H), 127.3 (Ar*C*H), 132.3 (Ar*C*), 139.1 (Ar*C*), 140.5 (Ar*C*), 141.1 (*C*=CH_2), 170.6 (*C*=O); **ν$_{max}$** (neat)/cm^{-1} 723, 778, 799, 867, 959, 1025, 1040, 1061, 1223, 1371, 1418, 1655, 1734, 2931; **HRMS** (APCI): Calcd. for $C_{14}H_{14}O_2BrS$ (M+H), 324.9892; found 324.9891.

Ethyl 2-((5-bromobenzo[b]thiophen-3-yl)methyl)acrylate 6e

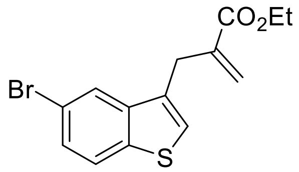

As described in general procedure **D**, 5-bromo-benzo[b]thiophene *S*-oxide (**1a**) (46 mg, 0.2 mmol), ethyl 2-((trimethylsilyl)methyl)acrylate (56 mg, 0.3 mmol) and trifluoroacetic anhydride (56 μL, 0.4 mmol), gave **6e** (50 mg, 0.16 mmol, 80%) as a colourless oil; **δ**$_H$ (400 MHz, $CDCl_3$) 1.30 (3 H, t, *J* = 7.0 Hz, C*H*$_3$), 3.83 (2 H, d, *J* = 0.5 Hz, C*H*$_2$), 4.24 (2 H, q, *J* = 7.2 Hz, C*H*$_2$), 5.44 (1 H, d, *J* = 1.2 Hz, C=C*H*$_2$), 6.28 (1 H, d, *J* = 1.2 Hz, C=C*H*$_2$), 7.20 (1 H, s, ArC*H*), 7.44 (1 H, dd, *J* = 8.5, 1.8 Hz, ArC*H*), 7.71 (1 H, d, *J* = 8.5 Hz, ArC*H*), 7.84 (1 H, d, *J* = 1.8 Hz, ArC*H*); **δ**$_C$ (101 MHz, $CDCl_3$) 14.2 (CH_3), 30.6 (CH_2), 61.0 (CH_2), 118.2 (Ar*C*Br), 124.1 (Ar*C*H), 124.7 (Ar*C*H), 125.3 (C=CH_2), 126.4 (Ar*C*H), 127.3 (Ar*C*H), 132.7 (*C*=CH_2), 138.2 (Ar*C*), 139.1 (Ar*C*), 140.3 (Ar*C*), 166.7 (*C*=O); **ν$_{max}$** (neat)/cm^{-1} 702, 749, 781, 818, 950, 1026, 1092, 1134, 1172, 1199, 1249, 1298, 1418, 1579, 1709, 2978; **HRMS** (APCI): Calcd. for $C_{14}H_{14}O_2BrS$ (M+H), 324.9892; found 324.9890.

5-Bromo-3-(2-bromoallyl)benzo[b]thiophene 6f

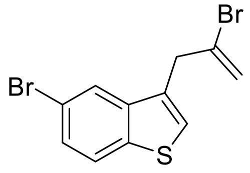

As described in general procedure **D**, 5-bromo-benzo[b]thiophene *S*-oxide (**1a**) (46 mg, 0.2 mmol), trimethyl(2-bromoallyl)silane (57 μL, 0.3 mmol) and trifluoroacetic anhydride (56 μL, 0.4 mmol), gave **6f** (53 mg, 0.16 mmol, 80%) as a colourless oil; **δ**$_H$ (400 MHz, $CDCl_3$) 3.95 (2 H, s, C*H*$_2$), 5.57 - 5.61 (2 H, m, C=C*H*$_2$), 7.33 (1 H, s, ArC*H*), 7.46 (1 H, dd, *J* = 8.6, 1.9 Hz, ArC*H*), 7.73 (1 H, d, *J* = 8.6 Hz, ArC*H*), 7.86 (1 H, d, *J* = 1.8 Hz, ArC*H*); **δ**$_C$ (101 MHz, $CDCl_3$) 40.9 (CH_2), 118.7 (Ar*C*Br), 119.1 (C=CH_2), 124.5 (Ar*C*H), 124.9 (Ar*C*H), 126.6 (Ar*C*H), 127.8 (Ar*C*H), 130.5 (*C*=CH_2), 131.3 (Ar*C*), 139.3 (Ar*C*), 140.4 (Ar*C*); **ν$_{max}$** (neat)/cm^{-1} 692, 719, 816, 917, 1032, 1063, 1172, 1212, 1247, 1341, 1413, 1580, 1634, 1837, 2913, 3084; **HRMS** (APCI): Calcd. for $C_{11}H_9Br_2S$ $(M)^+$, 329.8708; found 329.8708.

(*E*)-5-(5-Bromobenzo[*b*]thiophen-3-yl)pent-3-en-2-one 6g

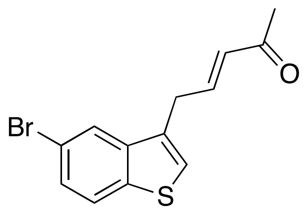

As described in general procedure **D**, 5-bromo-benzo[b]thiophene-*S*-oxide (**1a**) (46 mg, 0.2 mmol), (*E*)-5-(trimethylsilyl)pent-3-en-2-one (**5g**) (47 mg, 0.3 mmol) and trifluoroacetic anhydride (56 µL, 0.4 mmol), gave **6g** (34 mg, 0.116 mmol, 58%) as a yellow oil; **δ_H** (400 MHz, $CDCl_3$) 2.26 (3 H, s, C*H_3*), 3.72 (2 H, dd, *J* = 6.4, 1.6, Hz, C*H_2*), 6.11 (1 H, dt, *J* = 15.9, 1.6 Hz, CH=C*H*), 6.97 (1 H, dt, *J* = 15.9, 6.4 Hz, CH=C*H*), 7.21 (1 H, s, ArC*H*), 7.46 (1 H, dd, *J* = 8.5, 1.9 Hz, ArC*H*), 7.72 (1 H, dd, *J* = 8.5, 0.5 Hz, ArC*H*), 7.79 - 7.81 (1 H, m, ArC*H*); **δ_C** (101 MHz, $CDCl_3$) 27.4 (CH_3), 31.4 (CH_2), 118.5 (Ar*C*), 124.4 (Ar*C*H), 124.5 (Ar*C*H), 125.3 (Ar*C*H), 127.7 (Ar*C*H), 131.6 (Ar*C*), 132.7 (CH=*C*H), 139.2 (Ar*C*), 140.2 (Ar*C*), 143.9 (*C*H=CH), 198.3 (*C*=O); **ν_{max}** (neat)/cm^{-1} 793, 979, 1066, 1251, 1359, 1418, 1625, 1669, 2919, 3089; **HRMS** (APCI): Calcd. for $C_{13}H_{12}OSBr$ $(M+1)^+$, 294.9787; found 294.9786.

(*E*)-Methyl 4-(5-bromobenzo[*b*]thiophen-3-yl)but-2-enoate 6h

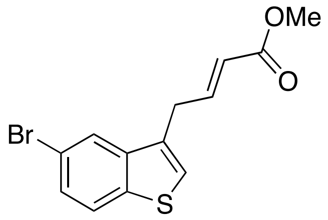

As described in general procedure **D**, 5-bromo-benzo[b]thiophene-*S*-oxide (**1a**) (46 mg, 0.2 mmol), (*E*)-methyl 4-(trimethylsilyl)but-2-enoate (**5h**) (52 mg, 0.3 mmol) and trifluoroacetic anhydride (56 µL, 0.4 mmol), gave **6h** (45 mg, 0.146 mmol, 73%) as a yellow oil; **δ_H** (400 MHz, $CDCl_3$) 3.70 (2 H, dd, *J* = 6.5, 1.7 Hz, C*H_2*), 3.73 (3 H, s, OC*H_3*), 5.86 (1 H, dt, *J* = 15.7, 1.7 Hz, CH=C*H*), 7.16 (1 H, dt, *J* = 15.7, 6.5 Hz, C*H*=CH), 7.20 (1 H, s, ArC*H*), 7.45 (1 H, dd, *J* = 8.5, 1.8 Hz, ArC*H*), 7.71 (1 H, d, *J* = 8.5 Hz, ArC*H*), 7.80 (1 H, d, *J* = 1.8 Hz, ArC*H*); **δ_C** (101 MHz, $CDCl_3$) 31.2 (CH_2), 51.7 (CH_3), 118.5 (Ar*C*), 123.0 (*C*H=CH), 124.4 (Ar*C*H), 124.5 (Ar*C*H), 125.2 (Ar*C*H), 127.7 (Ar*C*H), 131.7 (Ar*C*), 139.2 (Ar*C*), 140.2 (Ar*C*), 145.3 (CH=*C*H), 166.8 (*C*=O); **ν_{max}** (neat)/cm^{-1} 785, 982, 1067, 1161, 1208, 1271, 1432, 1655, 1715, 2948, 3092; **HRMS** (APCI): Calcd. for $C_{13}H_2OSBr$ $(M+H)^+$, 310.9736; found 310.9734.

2-Bromo-3-(hept-2-yn-1-yl)benzo[b]thiophene 8a

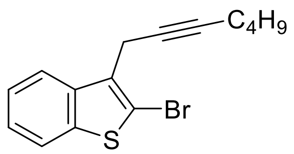

As described in general procedure **D**, 2-bromobenzo[b]thiophene *S*-oxide (**1e**) (45 mg, 0.2 mmol), hept-2-yn-1-yltrimethylsilane (**7a**) (50.0 mg, 0.3 mmol) and trifluoroacetic anhydride (56 µl, 0.4 mmol), gave **8a** (60 mg, 0.19 mmol, 98%) as a brown oil; **δ_H** (400 MHz, $CDCl_3$) 0.89 (3 H, t, *J* = 7.3 Hz, C*H_3*), 1.33 - 1.52 (4 H, m, 2 × C*H_2*), 2.14 (2 H, tt, *J* = 7.0, 2.4 Hz, C*H_2*), 3.74 (2 H, t, *J* =

2.4 Hz, CH_2), 7.32 - 7.42 (2 H, m, ArC*H*), 7.73 (1 H, ddd, *J* = 7.8, 1.4, 0.7 Hz, ArC*H*), 7.88 - 7.96 (1 H, m, ArC*H*); **δ_C** (101 MHz, $CDCl_3$) 13.6 (CH_3), 18.2 (CH_2), 18.4 (CH_2), 21.9 (CH_2), 30.9 (CH_2), 75.2 (*C*C), 81.6 (C*C*), 113.3 (Ar*C*Br), 121.7 (Ar*C*H), 122.2 (Ar*C*H), 124.5 (Ar*C*H), 124.6 (Ar*C*H), 131.2 (Ar*C*), 137.8 (Ar*C*), 139.7 (Ar*C*); **ν_{max}** (neat)/cm^{-1} 764, 824, 1025, 1067, 1149, 1247, 1263, 1347, 1418, 1498, 1662, 1706, 2858, 2928, 2952; **HRMS** (EI): Calcd. for $C_{15}H_{15}SBr$ $(M)^+$, 306.0072; found 306.0070.

3-(Hept-2-yn-1-yl)benzo[b]thiophene-2-carbonitrile 8b

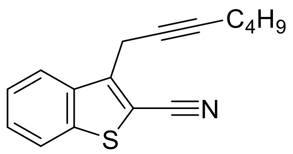

As described in general procedure **D**, 2-carbonitrile-benzo[b]thiophene *S*-oxide (**1f**) (35 mg, 0.2 mmol), hept-2-yn-1-yltrimethylsilane (**7a**) (50.0 mg, 0.3 mmol) and trifluoroacetic anhydride (56 µl, 0.4 mmol), gave **8b** (46 mg, 0.18 mmol, 91 %) as a brown oil; **δ_H** (400 MHz, $CDCl_3$) 0.89 (3 H, t, *J* = 7.3 Hz, CH_3), 1.34 - 1.49 (4 H, m, 2 x CH_2), 2.16 (2 H, tt, *J* = 7.0, 2.4 Hz, CH_2), 3.93 (2 H, t, *J* = 2.4 Hz, CH_2), 7.44 - 7.62 (2 H, m, ArC*H*), 7.84 (1 H, dt, *J* = 8.3, 0.8 Hz, ArC*H*), 8.09 (1 H, dq, *J* = 7.9, 0.7 Hz, ArC*H*); **δ_C** (101 MHz, $CDCl_3$) 12.9 (CH_3), 18.4 (CH_2), 18.8 (CH_2), 21.7 (CH_2), 30.6 (CH_2), 74.1 (*C*C), 83.4 (C*C*), 106.3 (Ar*C*CN), 113.9 (ArC*C*N), 122.6 (Ar*C*H), 124.2 (Ar*C*H), 125.3 (Ar*C*H), 127.9 (Ar*C*H), 136.5 (Ar*C*), 140.9 (Ar*C*), 143.8 (Ar*C*); **ν_{max}** (neat)/cm^{-1} 730, 755, 853, 1129, 1157, 1173, 1428, 1688, 2216, 2930, 2956; **HRMS** (EI): Calcd. for $C_{16}H_{16}NS$ $(M+H)^+$, 254.0998; found 254.0993.

Methyl 3-(hept-2-yn-1-yl)benzo[b]thiophene-2-carboxylate 8c

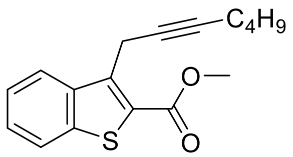

As described in general procedure **D**, 2-methylcarboxylate-benzo[b]thiophene *S*-oxide (**1c**) (41 mg, 0.2 mmol), hept-2-yn-1-yltrimethylsilane (**7a**) (50.0 mg, 0.3 mmol) and trifluoroacetic anhydride (56 µl, 0.4 mmol), gave **8c** (42 mg, 0.14 mmol, 72 %) as a yellow oil; **δ_H** (400 MHz, $CDCl_3$) 0.86 (3 H, t, *J* = 7.3 Hz, CH_3), 1.29 - 1.48 (4 H, m, 2 × CH_2), 2.12 (2 H, tt, *J* = 7.0, 2.4 Hz, CH_2), 3.95 (3 H, s, CH_3), 4.27 (2 H, t, *J* = 2.5 Hz, CH_2), 7.43 - 7.52 (2 H, m, ArC*H*), 7.85 (1 H, s, ArC*H*), 8.10 - 8.14 (1 H, m, ArC*H*); **δ_C** (101 MHz, $CDCl_3$) 13.5 (CH_3), 16.9 (CH_2), 18.4 (CH_2), 21.8 (CH_2), 30.8 (CH_2), 52.2 (CH_3), 76.1 (*C*C), 81.2 (C*C*), 122.5 (Ar*C*H), 124.4 (Ar*C*H), 124.4 (Ar*C*H), 127.0 (Ar*C*), 127.2 (Ar*C*H), 139.1 (Ar*C*), 139.7 (Ar*C*), 140.4 (Ar*C*), 163.4 (ArCCO_2Me); **ν_{max}** (neat)/cm^{-1} 732, 756, 1061, 1104, 1199, 1241, 1327, 1435, 1530, 1676, 1711, 2871, 2931, 2955; **HRMS** (EI): Calcd. for $C_{17}H_{19}O_2S$ $(M+H)^+$, 287.1098; found 287.1100.

3-(Hept-2-yn-1-yl)-2-methylbenzo[b]thiophene 8d

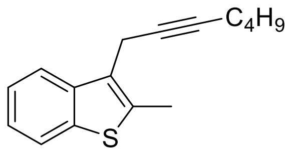

As described in general procedure **D**, 2-methylbenzo[b]thiophene *S*-oxide (**1g**) (32 mg, 0.2 mmol), hept-2-yn-1-yltrimethylsilane (**7a**) (50.0 mg, 0.3 mmol) and trifluoroacetic anhydride (56 µl, 0.4 mmol), gave **8d** (35 mg, 0.14 mmol, 72 %) as a yellow oil; δ_H (400 MHz, $CDCl_3$) 0.89 (3 H, t, *J* = 7.3 Hz, C*H*$_3$), 1.34 - 1.50 (4 H, m, 2 x C*H*$_2$), 2.14 (2 H, tt, *J* = 7.0, 2.4 Hz, C*H*$_2$), 2.56 (3 H, s, ArC*H*$_3$), 3.64 (2 H, t, *J* = 2.4 Hz, C*H*$_2$), 7.25 - 7.31 (1 H, m, ArC*H*), 7.37 (1 H, td, *J* = 7.5, 1.3 Hz, ArC*H*), 7.75 (1 H, d, *J* = 8.0 Hz, ArC*H*), 7.80 (1 H, d, *J* = 7.8 Hz, ArC*H*); δ_C (101 MHz, $CDCl_3$) 13.6 (*C*H$_3$), 13.8 (*C*H$_3$), 16.2 (*C*H$_2$), 18.5 (*C*H$_2$), 22.0 (*C*H$_2$), 31.0 (*C*H$_2$), 76.6 (C*C*), 80.9 (*C*C), 121.6 (ArC*H*), 122.0 (ArC*H*), 123.6 (ArC*H*), 123.9 (ArC*H*), 126.8 (ArC), 135.2 (ArC), 138.1 (ArC), 139.8 (ArC); ν_{max} (neat)/cm^{-1} 728, 907, 1025, 1174, 1324, 1435, 1460, 1669, 1708, 2870, 2929, 2956; **HRMS** (EI): Calcd. for $C_{16}H_{18}S$ $(M)^+$, 242.1124; found 242.1134.

3-(Hept-2-yn-1-yl)-2-pentylbenzo[b]thiophene 8e

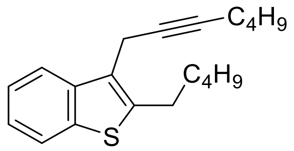

As described in general procedure **D**, 2-pentylbenzo[b]thiophene *S*-oxide (**1h**) (42 mg, 0.2 mmol), hept-2-yn-1-yltrimethylsilane (**7a**) (50.0 mg, 0.3 mmol) and trifluoroacetic anhydride (56 µl, 0.4 mmol), gave **8e** (39 mg, 0.13 mmol, 65 %) as a yellow oil; δ_H (400 MHz, $CDCl_3$) 0.82 - 0.96 (6 H, m, 2 × C*H*$_3$), 1.30 - 1.47 (8 H, m, 4 × C*H*$_2$), 1.63 - 1.81 (2 H, m, C*H*$_2$), 2.11 (2 H, tt, *J* = 7.0, 2.4 Hz, C*H*$_2$), 2.65 - 2.84 (2 H, m, C*H*$_2$), 3.41 (2 H, q, *J* = 2.3 Hz, C*H*$_2$), 7.41 (1 H, td, *J* = 7.5, 1.2 Hz, ArC*H*), 7.52 (1 H, td, *J* = 7.6, 1.1 Hz, ArC*H*), 7.57 - 7.62 (1 H, m, ArC*H*), 7.88 (1 H, dq, *J* = 7.5, 0.6 Hz, ArC*H*); δ_C (101 MHz, $CDCl_3$) 13.5 (*C*H$_3$), 14.0 (*C*H$_3$), 16.5 (*C*H$_2$), 18.3 (*C*H$_2$), 21.9 (*C*H$_2$), 22.4 (*C*H$_2$), 25.2 (*C*H$_2$), 29.5 (*C*H$_2$), 30.7 (*C*H$_2$), 31.5 (*C*H$_2$), 74.0 (C*C*), 82.6 (C*C*), 122.6 (Ar*C*H), 125.9 (Ar*C*H), 127.7 (Ar*C*H), 131.8 (Ar*C*H), 135.4 (Ar*C*), 138.6 (Ar*C*), 143.6 (Ar*C*), 149.0 (Ar*C*); ν_{max} (neat)/cm^{-1} 757, 934, 1047, 1097, 1149, 1170, 1214, 1289, 1347, 1456, 1499, 1643, 1787, 2825, 2970, 2979; **HRMS** (EI): Calcd. for $C_{20}H_{26}S$ $(M)^+$, 298.1750; found 298.1748.

3-(Hept-2-yn-1-yl)-2-phenylbenzo[b]thiophene 8f

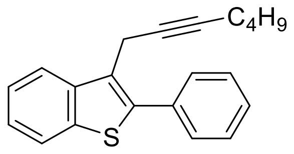

As described in general procedure **D**, 2-phenylbenzo[b]thiophene *S*-oxide (**1d**) (45 mg, 0.2 mmol), hept-2-yn-1-yltrimethylsilane (**7a**) (50.0 mg, 0.3 mmol) and trifluoroacetic anhydride (56 µl, 0.4 mmol), gave **8f** (58 mg, 0.19 mmol, 95 %) as a yellow oil; δ_H (400 MHz, $CDCl_3$) 0.88 - 0.95 (3 H, d,

J = 7.5 Hz, CH_3), 1.38 - 1.53 (4 H, m, 2 × CH_2), 2.19 (2 H, tt, *J* = 6.9, 2.4 Hz, CH_2), 3.74 (2 H, t, *J* = 2.4 Hz, CH_2), 7.36 - 7.53 (5 H, m, ArC*H*), 7.64 - 7.70 (2 H, m, ArC*H*), 7.86 (1 H, dt, *J* = 7.7, 0.9 Hz, ArC*H*), 7.98 (1 H, dd, *J* = 8.0, 0.5 Hz, ArC*H*); **δ_C** (101 MHz, $CDCl_3$) 13.6 (CH_3), 17.4 (CH_2), 18.5 (CH_2), 21.9 (CH_2), 31.0 (CH_2), 77.2 (*C*C), 81.5 (C*C*), 122.1 (Ar*C*H), 122.6 (Ar*C*H), 124.2 (Ar*C*H), 124.4 (Ar*C*H), 127.2 (Ar*C*), 128.1 (Ar*C*H), 128.7 (Ar*C*H), 129.6 (Ar*C*H), 134.1 (Ar*C*), 139.0 (Ar*C*), 139.3 (Ar*C*), 140.1 (Ar*C*); **ν_{max}** (neat)/cm^{-1} 697, 715, 730, 751, 907, 1028, 1079, 1323, 1434, 1601, 2859, 2869, 2928, 2955; **HRMS** (EI): Calcd. for $C_{21}H_{21}S$ $(M+H)^+$, 305.1357; found 305.1358.

3-(Hept-2-yn-1-yl)benzo[*b*]thiophene 8g

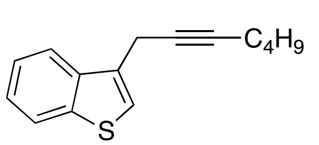

As described in general procedure **E**, benzo[b]thiophene (67 mg, 0.5 mmol) and H_2O_2 (60 µl, 30% aq., 0.5 mmol) in CH_2Cl_2 and TFA (2 mL, 1:1), *then* hept-2-yn-1-yltrimethylsilane (**7a**) (126 mg, 0.75 mmol) and trifluoroacetic anhydride (105 µl, 0.75 mmol), gave **8g** (68 mg, 0.3 mmol, 60 %) as a colourless oil; **δ_H** (400 MHz, $CDCl_3$) 0.94 (3 H, t, *J* = 7.3 Hz, CH_3), 1.41 - 1.58 (4 H, m, 2 × CH_2), 2.25 (2 H, m, CH_2), 3.73 (2 H,dq, *J* = 2.8, 1.7, 1.3 Hz, CH_2), 7.34 - 7.42 (3 H, m, ArC*H*), 7.75 - 7.78 (1 H, m, ArC*H*), 7.85 - 7.88 (1 H, m, ArC*H*); **δ_C** (101 MHz, $CDCl_3$) 13.8 (CH_3), 18.7 (CH_2), 19.3 (CH_2), 22.2 (CH_2), 31.2 (CH_2), 76.4 (C*C*), 83.0 (C*C*), 121.6 (Ar*C*H), 122.8 (Ar*C*H), 123.0 (Ar*C*H), 124.0 (Ar*C*H), 124.4 (Ar*C*H), 132.2 (Ar*C*), 138.2 (Ar*C*), 140.8 (Ar*C*); **ν_{max}** (neat)/cm^{-1} 1072, 1252, 1430, 1460, 2870, 2929, 2955; **HRMS** (APCI): Calcd. for $C_{15}H_{17}S$ $(M+H)^+$, 229.1045; found 229.1043.

3-(Hept-2-yn-1-yl)-5-nitrobenzo[b]thiophene 8h

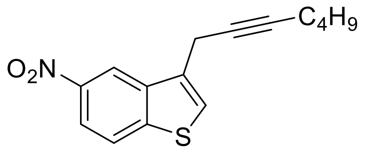

As described in general procedure **D**, 5-nitrobenzo[b]thiophene *S*-oxide (**1b**) (39 mg, 0.2 mmol), hept-2-yn-1-yltrimethylsilane (**7a**) (50 mg, 0.3 mmol) and trifluoroacetic anhydride (56 µl, 0.4 mmol), gave **8h** (42 mg, 0.15 mmol, 76 %) as a yellow oil; **δ_H** (400 MHz, $CDCl_3$) 0.92 (3 H, t, *J* = 7.3 Hz, CH_3), 1.40 - 1.56 (4 H, m, 2 × CH_2), 2.25 (2 H, tt, *J* = 7.0, 2.4 Hz, CH_2), 3.79 (2 H, td, *J* = 2.4, 1.3 Hz, CH_2), 7.55 (1 H, t, *J* = 1.0 Hz, ArC*H*), 7.93 - 7.97 (1 H, m, ArC*H*), 8.18 - 8.25 (1 H, m, ArC*H*), 8.67 - 8.74 (1 H, m, ArC*H*); **δ_C** (101 MHz, $CDCl_3$) 13.6 (CH_3), 18.4 (CH_2), 19.2 (CH_2), 22.0 (CH_2), 30.9 (CH_2), 75.3 (*C*C), 83.6 (C*C*), 117.5 (Ar*C*H), 118.7 (Ar*C*H), 123.4 (Ar*C*H), 126.2 (Ar*C*H), 133.4 (Ar*C*), 138.0 (Ar*C*), 145.2 (Ar*C*NO_2), 146.6 (Ar*C*); **ν_{max}** (neat)/cm^{-1} 729, 819, 907, 1035, 1119, 1247, 1341, 1379, 1510, 1533, 1682,

1708, 2871, 2931, 2957; **HRMS** (EI): Calcd. for $C_{15}H_{16}O_2NS$ $(M+H)^+$, 274.0896; found 274.0887.

5-Bromo-3-(hept-2-yn-1-yl)benzo[b]thiophene 8i

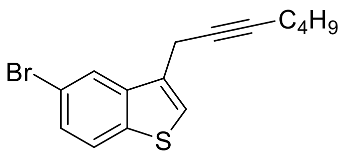

As described in general procedure **D**, 5-bromobenzo[b]thiophene *S*-oxide (**1a**) (46 mg, 0.2 mmol) hept-2-yn-1-yltrimethylsilane (**7a**) (50 mg, 0.3 mmol) and trifluoroacetic anhydride (56 µl, 0.4 mmol), gave **8i** (42 mg, 0.13 mmol, 68 %) as a yellow oil; δ_H (400 MHz, $CDCl_3$) 0.94 (3 H, t, *J* = 7.3 Hz, C*H*$_3$), 1.41 - 1.58 (4 H, m, 2 × C*H*$_2$), 2.25 (2 H, tt, *J* = 7.0, 2.3 Hz, C*H*$_2$), 3.68 (2 H, td, *J* = 2.4, 1.3 Hz, C*H*$_2$), 7.38 - 7.41 (1 H, m, ArC*H*), 7.45 (1 H, dt, *J* = 8.5, 1.0 Hz, ArC*H*), 7.71 (1 H, dd, *J* = 8.5, 0.5 Hz, ArC*H*), 7.93 (1 H, d, *J* = 1.8 Hz, ArC*H*); δ_C (101 MHz, $CDCl_3$) 13.6 (*C*H$_3$), 18.5 (*C*H$_2$), 19.1 (*C*H$_2$), 22.0 (*C*H$_2$), 31.0 (*C*H$_2$), 75.8 (*C*C), 83.1 (C*C*), 118.1 (Ar*C*Br), 124.2 (Ar*C*H), 124.4 (2 × Ar*C*H), 127.3 (Ar*C*H), 131.5 (Ar*C*), 139.3 (Ar*C*), 139.8 (Ar*C*); ν_{max} (neat)/cm^{-1} 787, 867, 1067, 1247, 1418, 1498, 1662, 1706, 2858, 2928, 2952; **HRMS** (EI): Calcd. for $C_{15}H_{15}SBr$ $(M)^+$, 306.0072; found 306.0065.

3-(Hept-2-yn-1-yl)-5-(4-(trifluoromethyl)phenyl)benzo[b]thiophene 8j

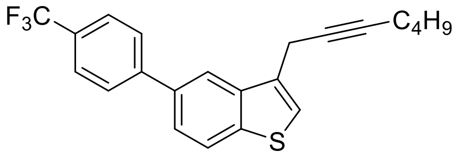

As described in general procedure **D**, 5-(4-(trifluoromethyl)phenyl)benzo[b]thiophene *S*-oxide (**1i**) (57 mg, 0.2 mmol), hept-2-yn-1-yltrimethylsilane (**7a**) (50.0 mg, 0.3 mmol) and trifluoroacetic anhydride (56 µl, 0.4 mmol), gave **8j** (63 mg, 0.17 mmol, 84 %) as a yellow oil;δ_H (400 MHz, $CDCl_3$) 0.91 (3 H, t, *J* = 7.3 Hz, C*H*$_3$), 1.41 - 1.57 (4 H, m, 2 × C*H*$_2$), 2.26 (2 H, tt, *J* = 7.0, 2.3 Hz, C*H*$_2$), 3.76 - 3.82 (2 H, m, C*H*$_2$), 7.45 (1 H, s, ArC*H*), 7.60 (1 H, dd, *J* = 8.4, 1.6 Hz, ArC*H*), 7.71 - 7.80 (4 H, m, ArC*H*), 7.95 (1 H, d, *J* = 8.5 Hz, ArC*H*), 7.98 (1 H, d, *J* = 1.3 Hz, ArC*H*); δ_C (101 MHz, $CDCl_3$) 13.6 (*CH*$_3$), 18.5 (*CH*$_2$), 19.2 (*CH*$_2$), 22.0 (*CH*$_2$), 31.0 (*CH*$_2$), 76.0 (*C*C), 83.0 (C*C*), 120.2 (Ar*C*H), 125.3 (d, *J* = 272.0 Hz, *C*F$_3$), 123.4 (Ar*C*H), 123.8 (Ar*C*H), 123.8 (Ar*C*H), 127.7 (Ar*C*H), 129.2 (q, *J* = 32.4 Hz, Ar*C*CF$_3$), 132.3 (Ar*C*), 136.0 (Ar*C*), 138.7 (Ar*C*), 140.6 (Ar*C*), 145.0 (Ar*C*); ν_{max} (neat)/cm^{-1} 731, 808, 907, 1069, 1121, 1272, 1321, 1615, 1709, 2931, 2958; **HRMS** (EI): Calcd. for $C_{22}H_{20}F_3S$ $(M+H)^+$, 373.1232; found 373.1227.

5-Bromo-3-(5-phenylpent-2-yn-1-yl)benzo[b]thiophene 8k

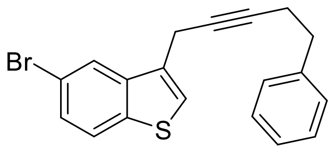

As described in general procedure **D**, 5-bromobenzo[b]thiophene *S*-oxide (**1a**) (46 mg, 0.2 mmol), trimethyl(5-phenylpent-2-yn-1-yl)silane (**7b**) (64 mg, 0.3 mmol) and trifluoroacetic anhydride (56 µl, 0.4 mmol), gave **8k** (57 mg, 0.16 mmol, 81 %) as a yellow oil; **δ_H** (400 MHz, $CDCl_3$) 2.57 (2 H, tt, *J* = 7.4, 2.4 Hz, C*H*$_2$), 2.88 (2 H, t, *J* = 7.4 Hz, C*H*$_2$), 3.64 (2 H, t, *J* = 2.4 Hz, C*H*$_2$), 7.21 - 7.33 (6 H, m, ArC*H*), 7.46 (1 H, dd, *J* = 8.5, 1.8 Hz, ArC*H*), 7.71 (1 H, d, *J* = 8.5 Hz, ArC*H*), 7.88 (1 H, d, *J* = 1.8 Hz, ArC*H*); **δ_C** (101 MHz, $CDCl_3$) 19.0 (*C*H$_2$), 20.9 (*C*H$_2$), 35.2 (*C*H$_2$), 76.7 (C*C*), 82.3 (*C*C), 118.1 (Ar*C*Br), 124.2 (Ar*C*H), 124.3 (Ar*C*H), 124.6 (Ar*C*H), 126.3 (Ar*C*H), 127.3 (Ar*C*H), 128.4 (2 × Ar*C*H), 128.5 (2 × Ar*C*H), 131.2 (Ar*C*), 139.3 (Ar*C*), 139.7 (Ar*C*), 140.7 (Ar*C*); **ν_{max}** (neat)/cm^{-1} 731, 860, 907, 1026, 1062, 1149, 1249, 1418, 1494, 1681, 1707, 2922, 3025; **HRMS** (ASAP): Calcd. for $C_{19}H_{16}SBr$ $(M+H)^+$, 355.0151; found 355.0145.

5-Bromo-3-(6-chlorohex-2-yn-1-yl)benzo[b]thiophene 8l

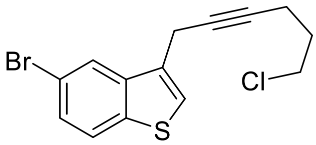

As described in general procedure **D**, 5-bromo-benzo[b]thiophene *S*-oxide (**1a**) (46 mg, 0.2 mmol), (6-chlorohex-2-yn-1-yl)trimethylsilane (56 mg, 0.3 mmol) and trifluoroacetic anhydride (56 µL, 0.4 mmol), gave **8l** (55 mg, 0.17 mmol, 84%) as a colourless oil; **δ_H** (400 MHz, $CDCl_3$) 2.00 (2 H, quin, *J* = 6.6 Hz, C*H*$_2$), 2.45 (2 H, tt, *J* = 6.8, 2.4 Hz, C*H*$_2$), 3.65 - 3.72 (4 H, m, 2 × C*H*$_2$), 7.39 (1 H, s, ArC*H*), 7.46 (1 H, dd, *J* = 8.5, 1.9 Hz, ArC*H*), 7.72 (1 H, d, *J* = 8.5 Hz, ArC*H*), 7.92 (1 H, d, *J* = 1.9 Hz, ArC*H*); **δ_C** (101 MHz, $CDCl_3$) 16.2 (*C*H$_2$), 19.1 (*C*H$_2$), 31.5 (*C*H$_2$), 43.7 (*C*H$_2$), 77.2 (C*C*CH$_2$), 81.0 (*C*CCH$_2$), 118.2 (Ar*C*Br), 124.2 (Ar*C*H), 124.4 (Ar*C*H), 124.5 (Ar*C*H), 127.4 (Ar*C*H), 131.1 (Ar*C*), 139.3 (Ar*C*), 139.7 (Ar*C*); **ν_{max}** (neat)/cm^{-1} 704, 797, 1037, 1064, 1149, 1207, 1262, 1418, 1498, 1544, 1580, 1678, 1708, 2920, 3093; **HRMS** (APCI): Calcd. for $C_{14}H_{12}BrClS$ (M)+, 325.9532; found 325.9530.

5-Bromo-3-(3-cyclohexylprop-2-yn-1-yl)benzo[b]thiophene 8m

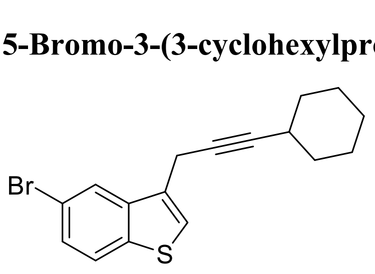

As described in general procedure **D**, 5-bromobenzo[b]thiophene *S*-oxide (**1a**) (46 mg, 0.2 mmol), (3-cyclohexylprop-2-yn-1-yl)trimethylsilane (**7c**) (54 mg, 0.3 mmol) and trifluoroacetic anhydride (56 µl, 0.4 mmol), gave **8m** (54 mg, 0.16 mmol, 82 %) as a yellow oil; **δ_H** (400 MHz, $CDCl_3$) 1.32 - 1.36 (2 H, m, C*H*$_2$), 1.44 - 1.57 (4 H, m, 2 × C*H*$_2$), 1.71 - 1.87 (4 H, m, 2 × C*H*$_2$), 2.44 (1 H, t, *J* = 9.0 Hz,

C*H*), 3.70 (2 H, t, *J* = 1.5 Hz, CH_2), 7.40 (1 H, s, ArC*H*), 7.45 (1 H, dd, *J* = 8.5, 1.8 Hz, ArC*H*), 7.71 (1 H, d, *J* = 8.5 Hz, ArC*H*), 7.94 (1 H, d, *J* = 2.0 Hz, ArC*H*); **δ_C** (101 MHz, $CDCl_3$) 19.2 (CH_2), 24.9 (2 × CH_2), 25.9 (2 × CH_2), 29.2 (*C*H), 32.9 (CH_2), 75.7 (C*C*), 87.5 (*C*C), 118.1 (Ar*C*Br), 124.2 (Ar*C*H), 124.4 (Ar*C*H), 124.5 (Ar*C*H), 127.3 (Ar*C*H), 131.6 (Ar*C*), 139.3 (Ar*C*), 139.8 (Ar*C*); **ν_{max}** (neat)/cm^{-1} 777, 814, 863, 906, 1026, 1059, 1071, 1149, 1250, 1344, 1421, 1446, 1556, 1580, 2849, 2897, 2930; **HRMS** (EI): Calcd. for $C_{17}H_{17}SBr$ $(M)^+$, 332.0229; found 332.0227.

3-(3-Cyclohexylprop-2-yn-1-yl)benzo[b]thiophene 8n

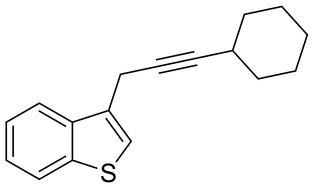

As described in general procedure **E**, benzo[b]thiophene (67 mg, 0.5 mmol) and H_2O_2 (60 µl, 30% aq., 0.5 mmol) in CH_2Cl_2 and TFA (2 mL, 1:1), *then* (3-cyclohexylprop-2-yn-1-yl)trimethylsilane (**7c**) (135 mg, 0.75 mmol) and trifluoroacetic anhydride (0.14 mL, 1.0 mmol), gave **8n** (74 mg, 0.30 mmol, 60 %) as a yellow oil; **δ_H** (400 MHz, $CDCl_3$) 1.25 - 1.58 (6 H, m, 3 × CH_2), 1.69 - 1.92 (4 H, m, 2 × CH_2), 2.45 (1 H, t, *J* = 9.0 Hz, C*H*), 3.73 - 3.77 (2 H, m, CH_2), 7.33 - 7.44 (3 H, m, ArC*H*), 7.77 (1 H, dt, *J* = 7.7, 0.7 Hz, ArC*H*), 7.83 - 7.90 (1 H, m, ArC*H*); **δ_C** (101 MHz, $CDCl_3$) 19.2 (2 × CH_2), 24.9 (2 × CH_2), 25.9 (2 × CH_2), 29.2 (*C*H), 33.0 (CH_2), 76.1 (*C*C), 87.2 (C*C*), 121.4 (Ar*C*H), 122.6 (Ar*C*H), 122.9 (Ar*C*H), 123.9 (Ar*C*H), 124.3 (Ar*C*H), 132.1 (Ar*C*), 138.1 (Ar*C*), 140.7 (Ar*C*); **ν_{max}** (neat)/cm^{-1} 725, 750, 906, 1429, 1447, 1681, 2851, 2926; **HRMS** (EI): Calcd. for $C_{17}H_{19}S$ $(M+H)^+$, 255.1202; found 255.1199.

5-Bromo-3-(3-phenylprop-2-yn-1-yl)benzo[b]thiophene 8o

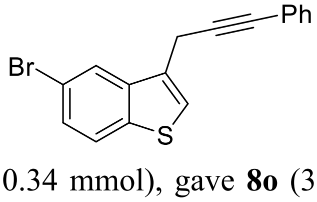

As described in general procedure **D**, 5-bromo-benzo[b]thiophene *S*-oxide (**1a**) (40 mg, 0.17 mmol), trimethyl(3-phenylprop-2-yn-1-yl)silane (49 mg, 0.26 mmol) and trifluoroacetic anhydride (49 µL, 0.34 mmol), gave **8o** (33 mg, 0.10 mmol, 59%) as a yellow solid; **m.p:** 91-92 °C; **δ_H** (400 MHz, $CDCl_3$) 3.95 (2 H, d, *J* = 1.3 Hz, CH_2), 7.30 - 7.35 (3 H, m, 3 × ArC*H*), 7.45 - 7.52 (4 H, m, 4 × ArC*H*), 7.73 (1 H, d, *J* = 8.5 Hz, ArC*H*), 7.99 (1 H, d, *J* = 1.8 Hz, ArC*H*); **δ_C** (101 MHz, $CDCl_3$) 19.7 (CH_2), 83.1 (C*C*Ph), 85.6 (*C*CPh), 118.3 (Ar*C*Br), 123.3 (Ar*C*), 124.3 (Ar*C*H), 124.4 (Ar*C*H), 124.9 (Ar*C*H), 127.5 (Ar*C*H), 128.1 (2 × Ar*C*H), 128.3 (Ar*C*H), 130.5 (Ar*C*), 131.7 (2 × Ar*C*H), 139.3 (Ar*C*), 139.8 (Ar*C*); **ν_{max}** (neat)/cm^{-1} 721, 813, 871, 910, 968, 1032, 1149, 1245, 1417, 1439, 1555, 1579, 1738, 3055; **HRMS** (APCI): Calcd. for $C_{17}H_{11}BrS$ (M)+, 325.9759; found 325.9759.

[3-(5-Bromobenzo[b]thiophen-3-yl)prop-1-yn-1-yl]trimethylsilane 8p

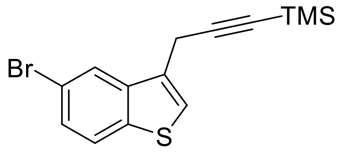

As described in general procedure **D**, 5-bromobenzo[b]thiophene *S*-oxide (**1a**) (46 mg, 0.2 mmol), prop-1-yne-1,3-diylbis(trimethylsilane) (**7d**) (55 mg, 0.3 mmol) and trifluoroacetic anhydride (56 μl, 0.4 mmol), gave **8p** (59 mg, 0.18 mmol, 91 %) as a yellow oil; **δ_H** (400 MHz, $CDCl_3$) 0.24 (9 H, s, Si(C*H*$_3$)$_3$), 3.75 (2 H, d, *J* = 1.3 Hz, C*H*$_2$), 7.41 (1 H, s, ArC*H*), 7.46 (1 H, dd, *J* = 8.5, 1.8 Hz, ArC*H*), 7.71 (1 H, d, *J* = 8.5 Hz, ArC*H*), 7.93 (1 H, d, *J* = 1.8 Hz, ArC*H*); **δ_C** (101 MHz, $CDCl_3$) 0.0 (Si(*C*H$_3$)$_3$), 20.2 (*C*H$_2$), 87.7 (*C*C Si(*C*H$_3$)$_3$), 102.3 (C*C* Si(*C*H$_3$)$_3$), 118.2 (Ar*C*Br), 124.2 (Ar*C*H), 124.4 (Ar*C*H), 124.8 (Ar*C*H), 127.4 (Ar*C*H), 130.1 (Ar*C*), 139.3 (Ar*C*), 139.6 (Ar*C*); **ν_{max}** (neat)/cm^{-1} 730, 777, 815, 999, 1012, 1045, 1149, 1248, 1419, 1581, 2178, 2957; **HRMS** (EI): Calcd. for $C_{14}H_{15}SSiBr$ $(M)^+$, 321.9842; found 321.9842.

[3-(Benzo[b]thiophen-3-yl)prop-1-yn-1-yl]trimethylsilane[3] 8q

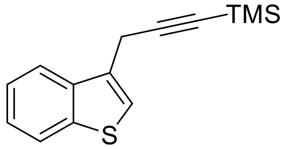

As described in general procedure **E** benzo[b]thiophene (67 mg, 0.5 mmol) and H_2O_2 (60 μl, 30% aq., 0.5 mmol) in CH_2Cl_2 and TFA (2 mL, 1:1), *then* prop-1-yne-1,3-diylbis(trimethylsilane) (**7d**) (0.13 g, 0.75 mmol) and trifluoroacetic anhydride (0.14 mL, 1.0 mmol), gave **8q** (97 mg, 0.39 mmol, 80 %) as a yellow oil; **δ_H** (400 MHz, $CDCl_3$) 0.89 (3 H, t, *J* = 7.3 Hz, C*H*$_3$), 1.33 - 1.52 (4 H, m, 2 × C*H*$_2$), 2.14 (2 H, tt, *J* = 7.0, 2.4 Hz, C*H*$_2$), 3.74 (2 H, t, *J* = 2.4 Hz, C*H*$_2$), 7.32 - 7.42 (2 H, m, 2 × ArC*H*), 7.73 (1 H, ddd, *J* = 7.8, 1.4, 0.7 Hz, C*H*$_2$), 7.88 - 7.96 (1 H, m, C*H*$_2$); **δ_C** (101 MHz, $CDCl_3$) 0.7 (Si(*C*H$_3$)$_3$), 20.2 (*C*H$_2$), 87.3 (*C*CSi(*C*H$_3$)$_3$), 102.9 (C*C*Si(*C*H$_3$)$_3$), 121.3 (ArC*H*), 122.9 (ArC*H*), 123.0 (ArC*H*), 124.0 (ArC*H*), 124.4 (ArC*H*), 130.6 (ArC), 137.9 (ArC), 140.6 (ArC); **ν_{max}** (neat)/cm^{-1} 739, 788, 840, 999, 1021, 1052, 1149, 1237, 1424, 1590, 2163; **HRMS** (EI): Calcd. for $C_{14}H_{15}SSi$ $(M-H)^+$ 244.0746; found 244.0738.

5-Bromo-3-(prop-2-yn-1-yl)benzo[b]thiophene 8r

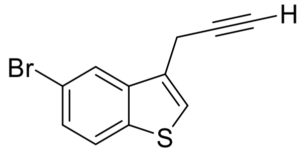

As described in general procedure **D**, 5-bromo-benzo[b]thiophene *S*-oxide (**1a**) (46 mg, 0.2 mmol), propargyl silane (44 μL, 0.3 mmol) and trifluoroacetic anhydride (56 μL, 0.4 mmol), gave **8r** (30 mg, 0.11 mmol, 58%) as a white solid; **m.p:** 82-84 °C; **δ_H** (400 MHz, $CDCl_3$) 2.26 (1 H, t, *J* = 2.8 Hz, CC*H*), 3.72 (2 H, dd, *J* = 2.8, 1.3 Hz, C*H*$_2$), 7.44 - 7.50 (2 H, m, 2 × ArC*H*), 7.72 (1 H, d, *J* = 8.5 Hz, ArC*H*), 7.90 (1 H, d, *J* = 1.8 Hz, ArC*H*); **δ_C** (101 MHz, $CDCl_3$) 18.7 (*C*H$_2$), 71.0 (C*C*H), 80.1 (*C*CH), 118.3 (Ar*C*Br), 124.2 (ArC*H*), 124.2 (ArC*H*), 124.9 (ArC*H*), 127.5

(ArC*H*), 129.8 (ArC), 139.2 (ArC), 139.5 (ArC); **ν_{max}** (neat)/cm^{-1} 721, 791, 799, 862, 1035, 1061, 1153, 1247, 1307, 1422, 1436, 1580, 1722, 3274; **HRMS** (APCI): Calcd. for $C_{11}H_7BrS$ (M)+, 249.9446; found 249.9446.

[3-(5-Bromobenzo[b]thiophen-3-yl)-3-cyclopentylprop-1-yn-1-yl]trimethylsilane 8s

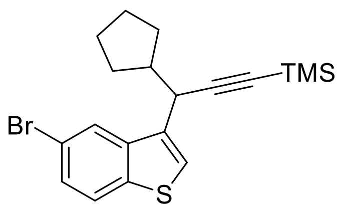

As described in general procedure **D**, 5-bromobenzo[b]thiophene *S*-oxide (**1a**) (46 mg, 0.2 mmol), (3-cyclopentylprop-1-yne-1,3-diyl)bis(trimethylsilane) (**7e**) (76 mg, 0.3 mmol) and trifluoroacetic anhydride (56 µl, 0.4 mmol), gave **8s** (67 mg, 0.17 mmol, 85 %) as a yellow oil; **δ_H** (400 MHz, $CDCl_3$) 0.21 (9 H, s, Si(CH_3)$_3$), 1.41 - 1.72 (8 H, m, 4 × CH_2), 2.36 - 2.46 (1 H, m, C*H*), 3.94 (1 H, d, *J* = 6.8 Hz, C*H*), 7.36 (1 H, s, ArC*H*), 7.44 (1 H, dd, *J* = 8.5, 1.8 Hz, ArC*H*), 7.71 (1 H, d, *J* = 8.5 Hz, ArC*H*), 8.09 (1 H, d, *J* = 1.8 Hz, ArC*H*); **δ_C** (101 MHz, $CDCl_3$) 0.1 (Si(*C*H$_3$)$_3$), 25.3 (*C*H$_2$), 25.5 (*C*H$_2$), 29.8 (*C*H$_2$), 31.3 (*C*H$_2$), 38.0 (*C*H), 44.1 (*C*H), 87.8 (*C*CSi(CH$_3$)$_3$), 106.2 (C*C*Si(CH$_3$)$_3$), 117.9 (Ar*C*Br), 124.2 (Ar*C*H), 124.6 (Ar*C*H), 125.1 (Ar*C*H), 127.2 (Ar*C*H), 135.3 (Ar*C*), 139.2 (Ar*C*), 139.5 (Ar*C*); **ν_{max}** (neat)/cm^{-1} 697, 732, 758, 785, 820, 838, 907, 938, 1042, 1066, 1150, 1247, 1415, 1429, 1580, 2170, 2865, 2954; **HRMS** (EI): Calcd. for $C_{19}H_{24}SSiBr$ $(M)^+$, 391.0546; found 391.0544.

[3-(5-Bromobenzo[b]thiophen-3-yl)hex-1-yn-1-yl]trimethylsilane 8t

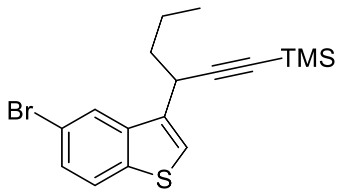

As described in general procedure **D**, 5-bromobenzo[b]thiophene *S*-oxide (**1a**) (46 mg, 0.2 mmol), hex-1-yne-1,3-diylbis(trimethylsilane) (**7f**) (68 mg, 0.3 mmol) and trifluoroacetic anhydride (56 µl, 0.4 mmol), gave **8t** (60 mg, 0.16 mmol, 83 %) as a yellow oil; **δ_H** (400 MHz, $CDCl_3$) 0.18 - 0.23 (9 H, s, Si(CH_3)$_3$), 0.97 (3 H, t, *J* = 7.4 Hz, CH_3), 1.55 (2 H, m, CH_2), 1.85 (2 H, d, *J* = 8.0 Hz, CH_2), 3.98 (1 H, s, C*H*), 7.36 (1 H, s, ArC*H*), 7.45 (1 H, dd, *J* = 8.7, 1.9 Hz, ArC*H*), 7.71 (1 H, d, *J* = 8.8 Hz, ArC*H*), 8.07 (1 H, d, *J* = 1.8 Hz, ArC*H*); **δ_C** (101 MHz, $CDCl_3$) 0.1 (Si(*C*H$_3$)$_3$), 13.7 (*C*H$_3$), 20.5 (*C*H$_2$), 32.9 (*C*H$_2$), 37.8 (*C*H), 87.5 (C*C*), 106.9 (*C*C), 118.0 (Ar*C*), 124.2 (Ar*C*H), 124.3 (Ar*C*H), 125.0 (Ar*C*H), 127.2 (Ar*C*H), 135.4 (Ar*C*), 139.1 (Ar*C*), 139.5 (Ar*C*); **ν_{max}** (neat)/cm^{-1} 731, 839, 907, 1068, 1248, 1323, 1431, 1580, 2170, 2932, 2957; **HRMS** (ESI): Calcd. for $C_{17}H_{21}SSi$ $(M)^+$, 364.0311; found 364.0298.

[3-(Benzo[b]thiophen-3-yl)hex-1-yn-1-yl]trimethylsilane 8u

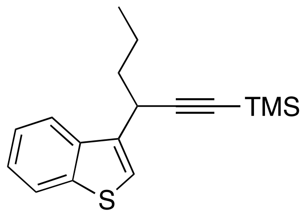

As described in general procedure **E**, benzo[b]thiophene (67 mg, 0.5 mmol) and H_2O_2 (60 µl, 30% aq., 0.5 mmol) in CH_2Cl_2 and TFA (2 mL, 1:1), *then* hex-1-yne-1,3-diylbis(trimethylsilane) (**7f**) (167 mg, 0.75 mmol) and trifluoroacetic anhydride (0.14 mL, 1.0 mmol), gave **8u** (0.11 g, 0.40 mmol, 80 %) as a colourless oil; **δ_H** (400 MHz, $CDCl_3$) 0.09 (9 H, s, Si(C*H*$_3$)$_3$), 0.85 (3 H, t, *J* = 7.4 Hz, C*H*$_3$), 1.43 (2 H, m, C*H*$_2$), 1.75 (2 H, m, C*H*$_2$), 3.95 (1 H, ddd, *J* = 8.5, 5.7, 0.7 Hz, C*H*), 7.25 (3 H, m, ArC*H*), 7.74 (2H, ddt, *J* = 8.3, 1.7, 0.8 Hz, ArC*H*); **δ_C** (126 MHz, $CDCl_3$) 0.31 (Si(*C*H$_3$)$_3$), 13.9 (*C*H$_3$), 20.7 (*C*H$_2$), 33.0 (*C*H), 38.1 (*C*H$_2$), 87.1 (C*C*), 107.7 (C*C*), 122.1 (Ar*C*H), 122.7 (Ar*C*H), 123.1 (Ar*C*H), 123.9 (Ar*C*H), 124.3 (Ar*C*H), 136.2 (Ar*C*), 137.6 (Ar*C*), 141.0 (Ar*C*); **ν_{max}** (neat)/cm^{-1} 730, 757, 837, 871, 943, 1018, 1072, 1140, 1248, 1458, 2169, 2871, 2956; **HRMS** (APCI): Calc. for $C_{17}H_{23}SSi$ (M+H$^+$), 287.1284; found 287.1282.

[3-(6-Bromobenzo[*b*]thiophen-3-yl)hex-1-yn-1-yl]trimethylsilane 8v

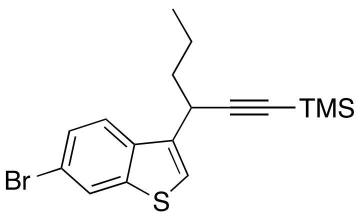

As described in general procedure **E**, 6-bromo-benzo[b]thiophene (106 mg, 0.5 mmol) and H_2O_2 (60 µl, 30% aq., 0.5 mmol) in CH_2Cl_2 and TFA (2 mL, 1:1), *then* hex-1-yne-1,3-diylbis(trimethylsilane (**7g**) (170 mg, 0.75 mmol) and trifluoroacetic anhydride (105 µl, 0.75 mmol), gave **8v** (157 mg, 0.3 mmol, 86 %) as a colourless oil; **δ_H** (400 MHz, $CDCl_3$) 0.18 (9 H, s, Si(C*H*$_3$)$_3$), 0.95 (3 H, t, *J* = 7.4 Hz, C*H*$_3$), 1.49 - 1.56 (2 H, m, C*H*$_2$), 1.80 - 1.86 (2 H, m, C*H*$_2$), 3.97 - 4.03 (1 H, m, C*H*), 7.32 (1 H, s, ArC*H*), 7.47 (1 H, dd, *J* = 8.6, 1.7 Hz, ArC*H*), 7.72 (1 H, d, *J* = 8.6 Hz, ArC*H*), 7.89 (1 H, m, *J* = 1.7 Hz, ArC*H*); **δ_C** (101 MHz, $CDCl_3$) 0.3 (Si(*C*H$_3$)$_3$), 13.9 (*C*H$_3$), 20.7 (*C*H$_2$), 33.0 (*C*H), 38.0 (*C*H$_2$), 87.4 (C*C*), 107.2 (C*C*), 118.3 (Ar*C*), 123.2 (Ar*C*H), 123.3 (Ar*C*H), 125.6 (Ar*C*H), 127.3 (Ar*C*H), 136.0 (Ar*C*), 136.4 (Ar*C*), 142.5 (Ar*C*); **ν_{max}** (neat)/cm^{-1} 758, 808, 838, 942, 1248, 1446, 1584, 2169, 2871, 2956; **HRMS** (ESI): Calcd. for $C_{17}H_{21}SSiBr$ (M)$^+$, 364.0311; found 364.0309.

Supplementary Figures

^{1}H and ^{13}C NMR spectra

Supplementary Figure 1.

1a ^{1}H NMR (400 MHz, $CDCl_3$)

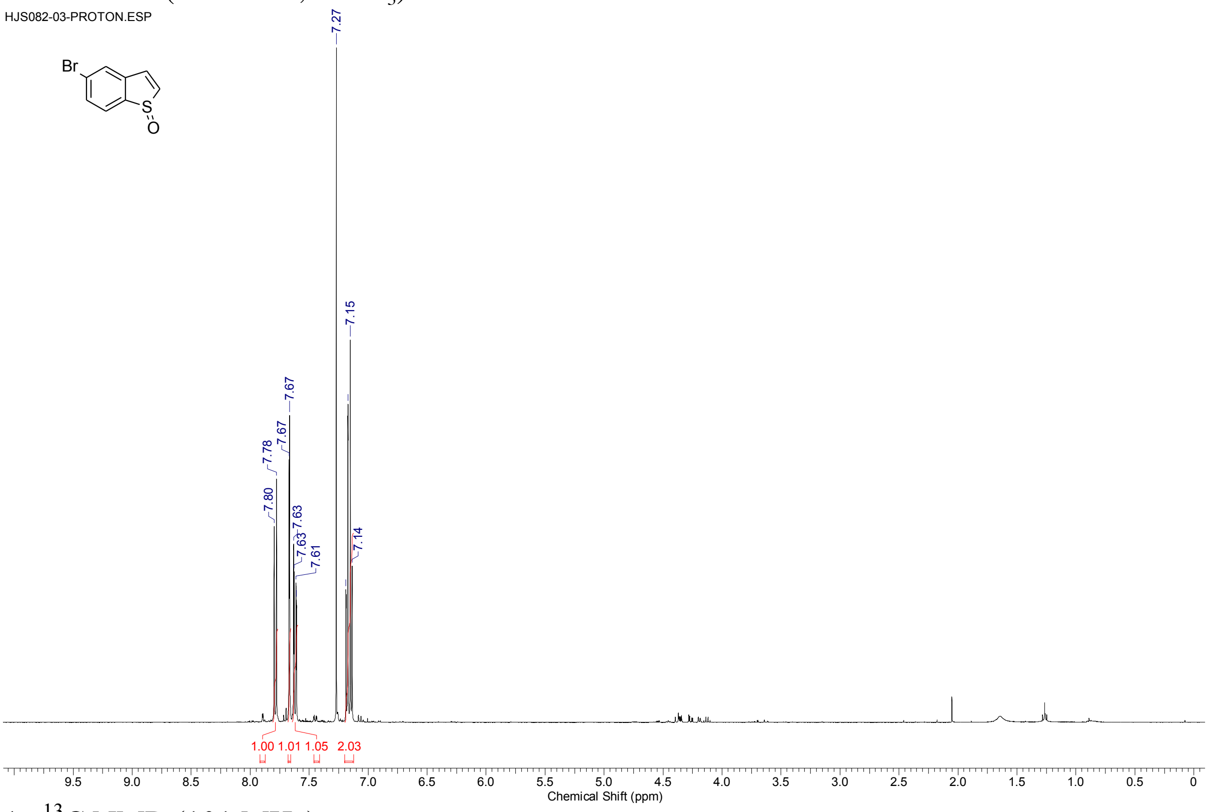

1a ^{13}C NMR (101 MHz)

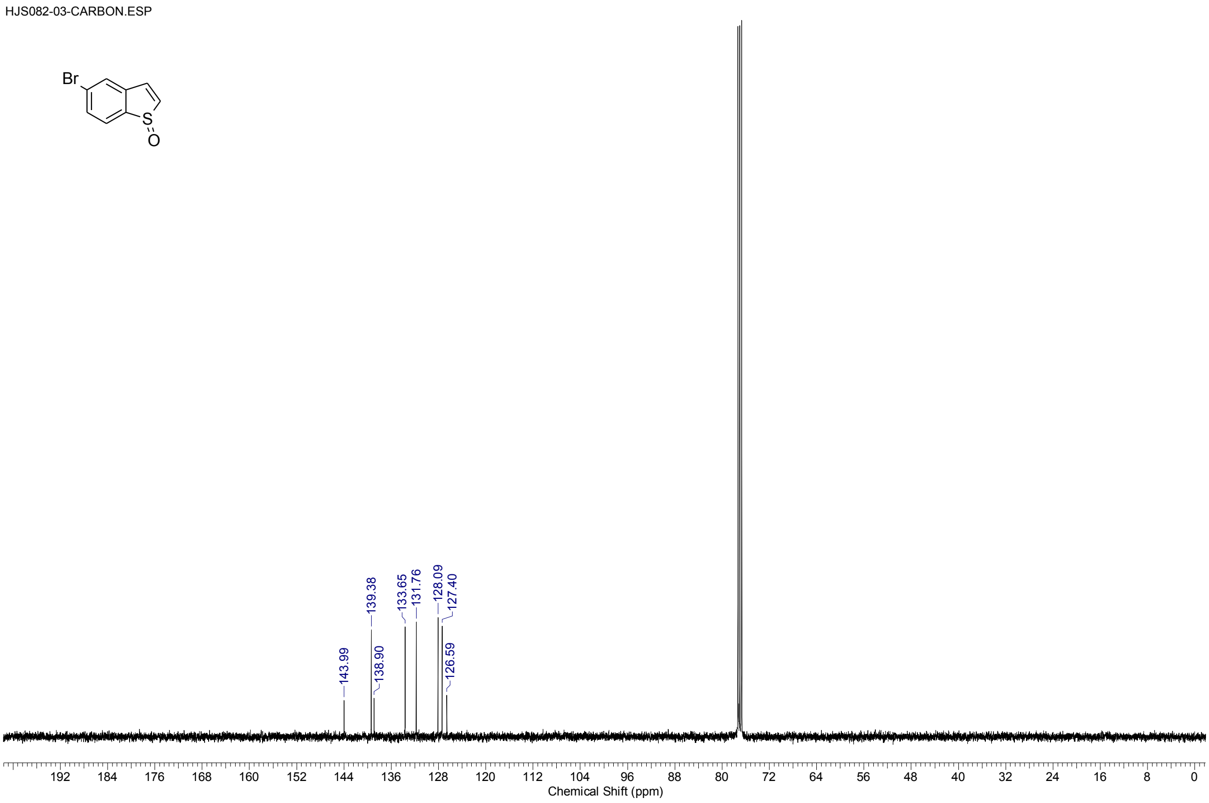

Supplementary Figure 2.

1b ^{1}H NMR (400 MHz, $CDCl_3$)

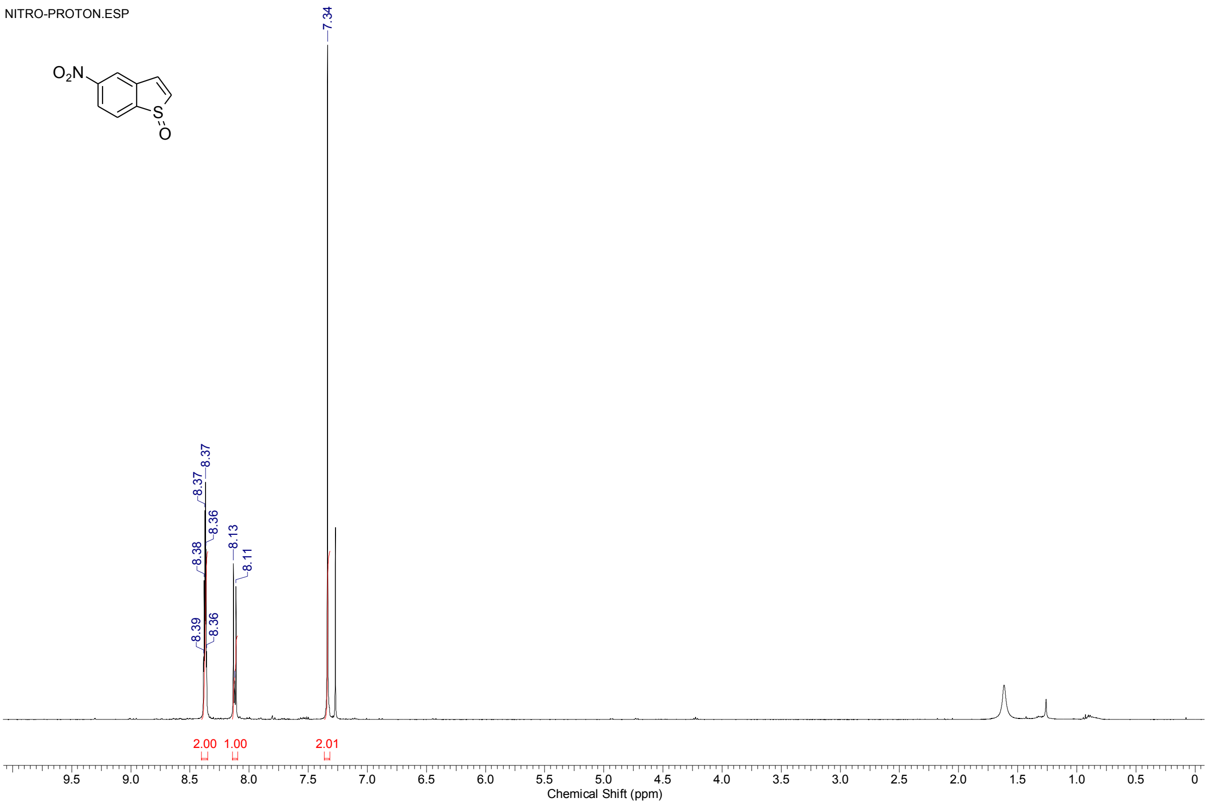

1b ^{13}C NMR (101 MHz, $CDCl_3$)

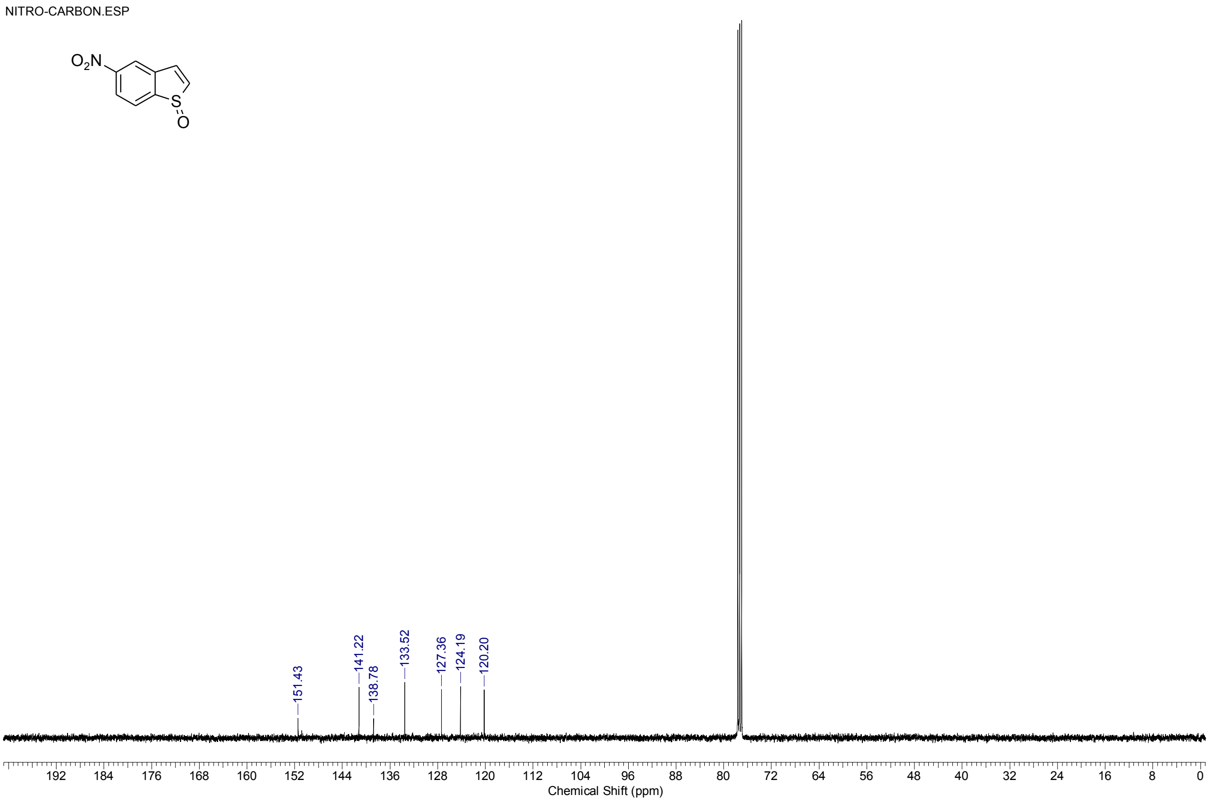

Supplementary Figure 3.

1c ^{1}H NMR (400 MHz, $CDCl_3$)

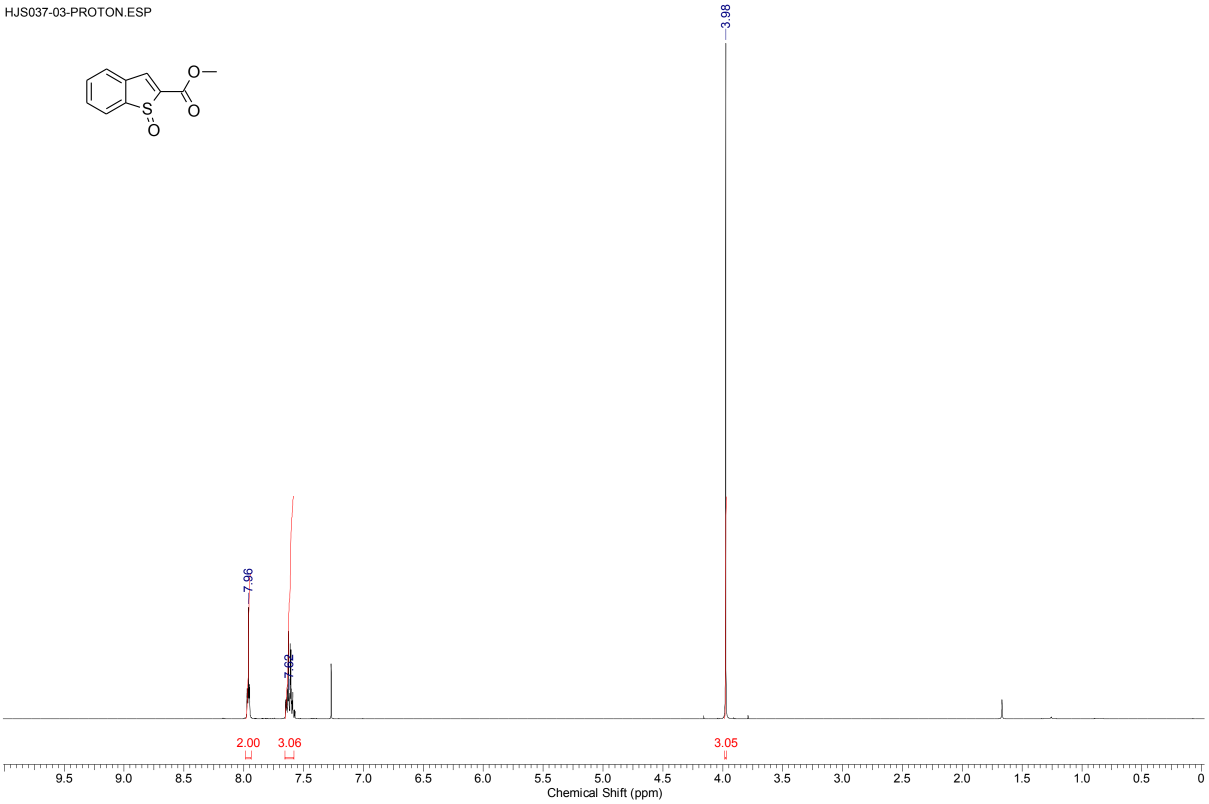

1c ^{13}C NMR (101 MHz, $CDCl_3$)

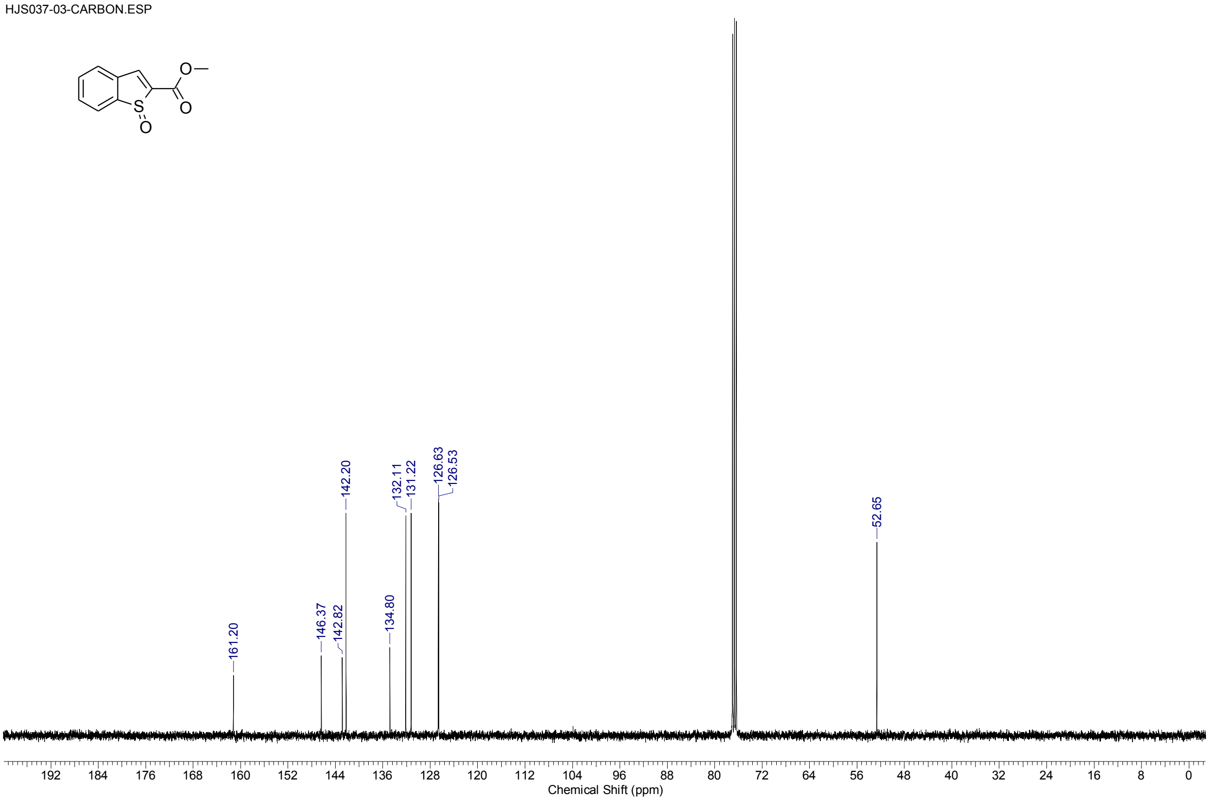

Supplementary Figure 4.

1d ^{1}H NMR (400 MHz, $CDCl_3$)

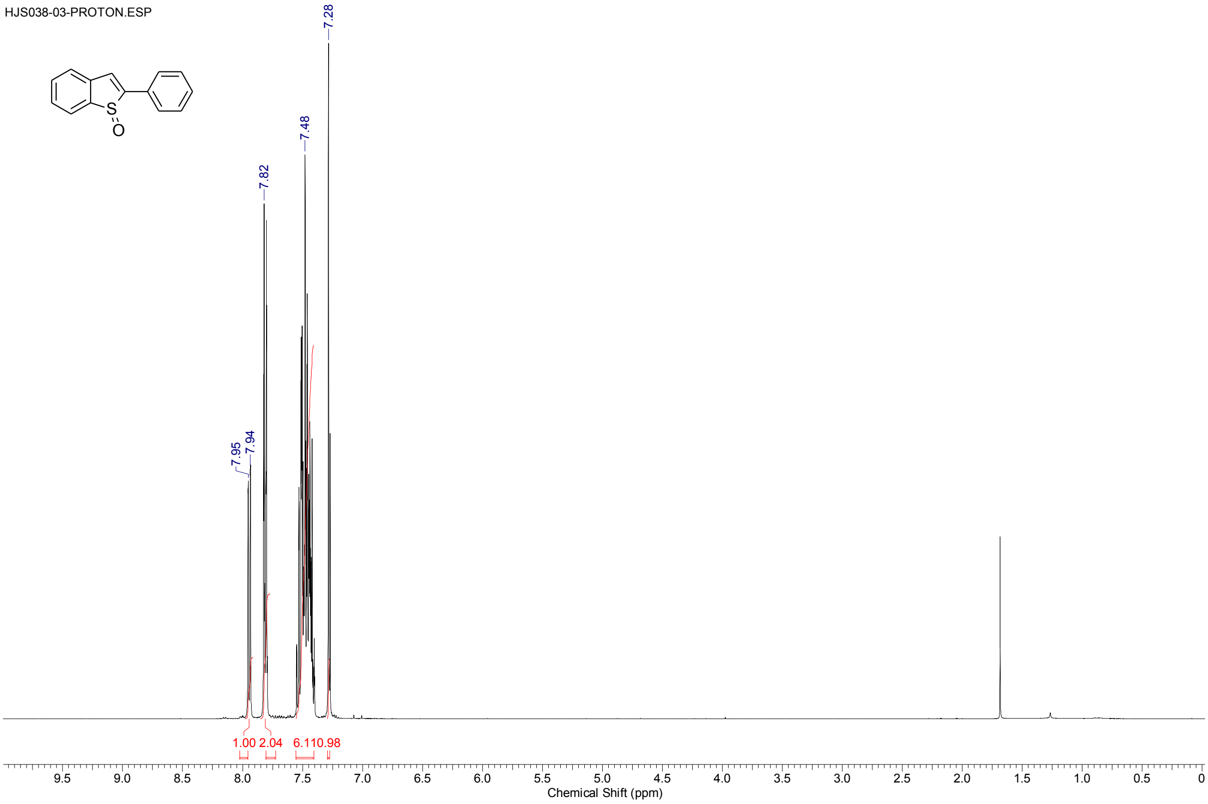

1d ^{13}C NMR (101 MHz, $CDCl_3$)

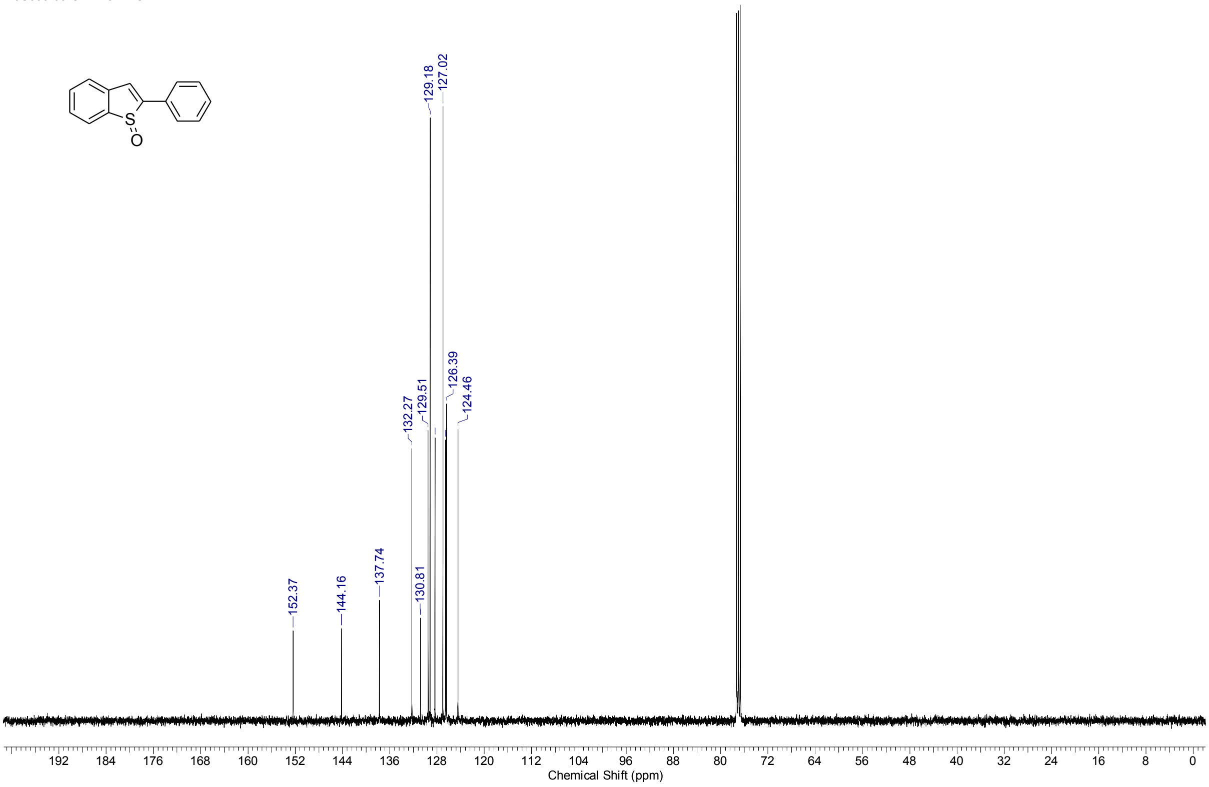

Supplementary Figure 5.

1e ^{1}H NMR (400 MHz, $CDCl_3$)

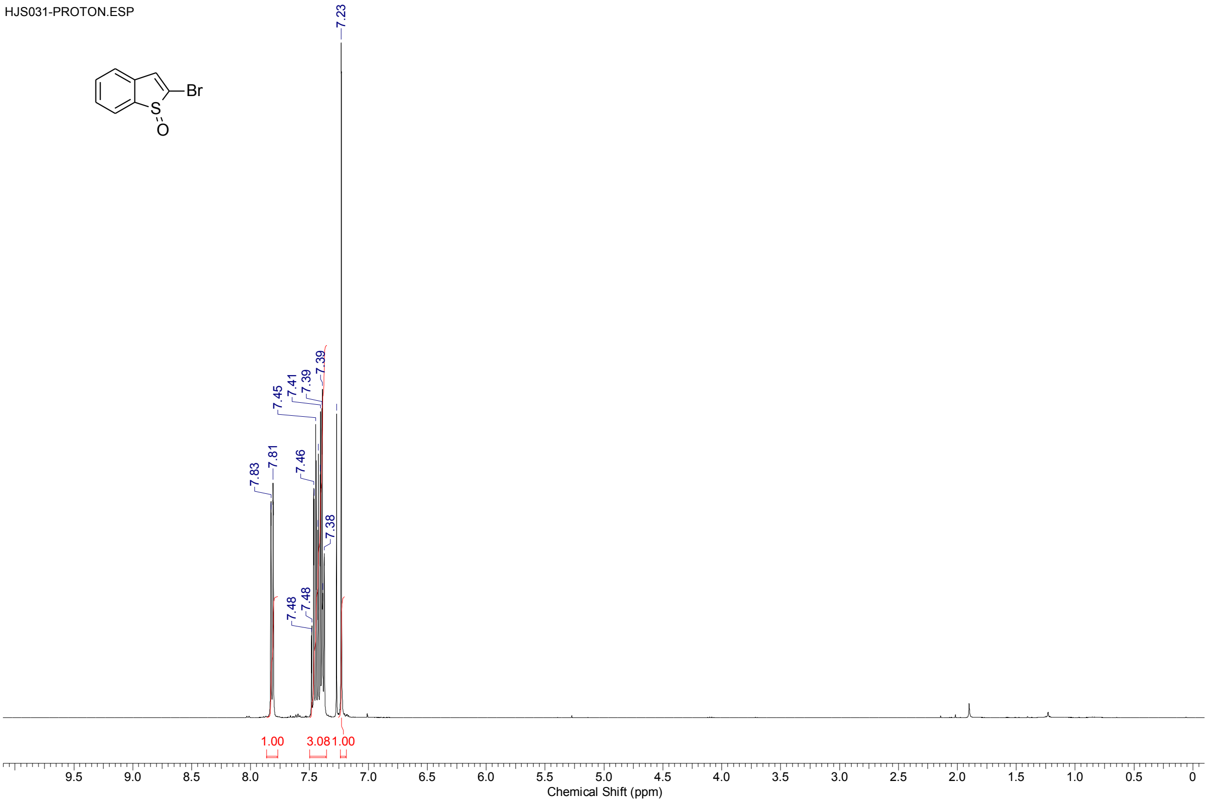

1e ^{13}C NMR (101 MHz, $CDCl_3$)

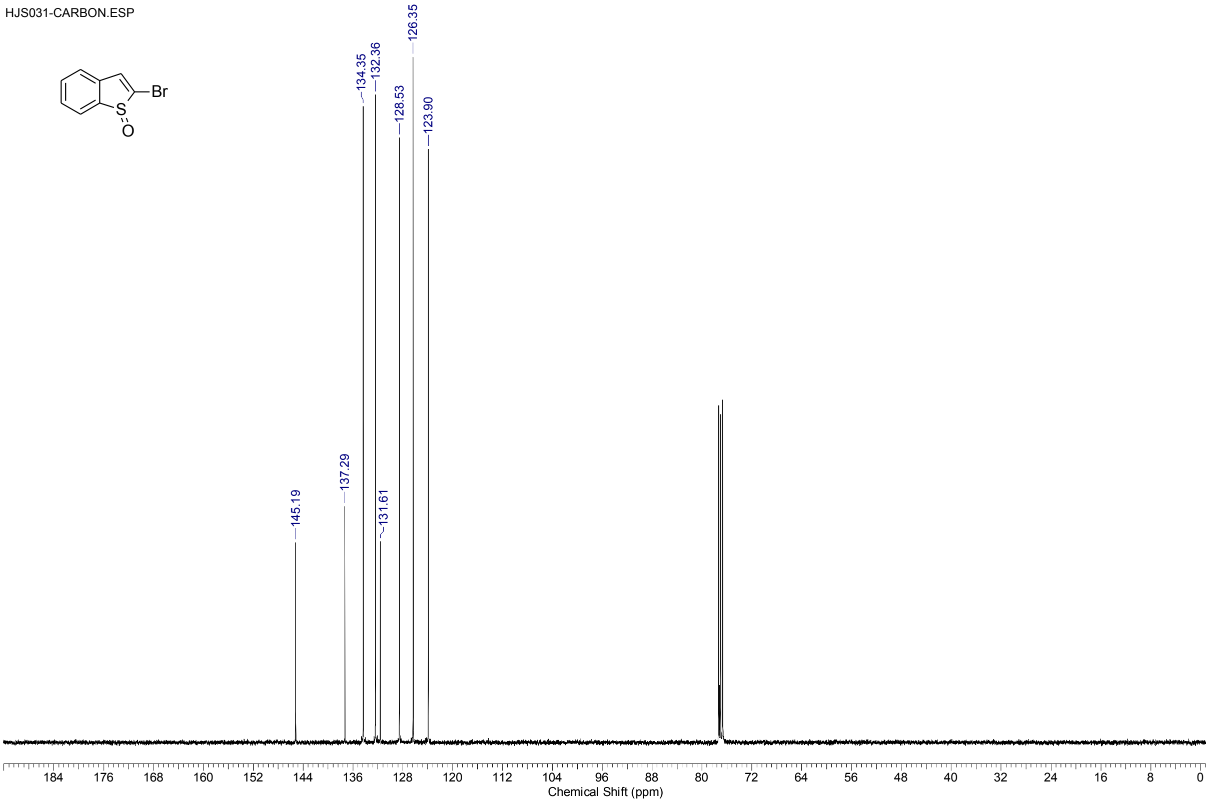

Supplementary Figure 6.

1f ^{1}H NMR (400 MHz, $CDCl_3$)

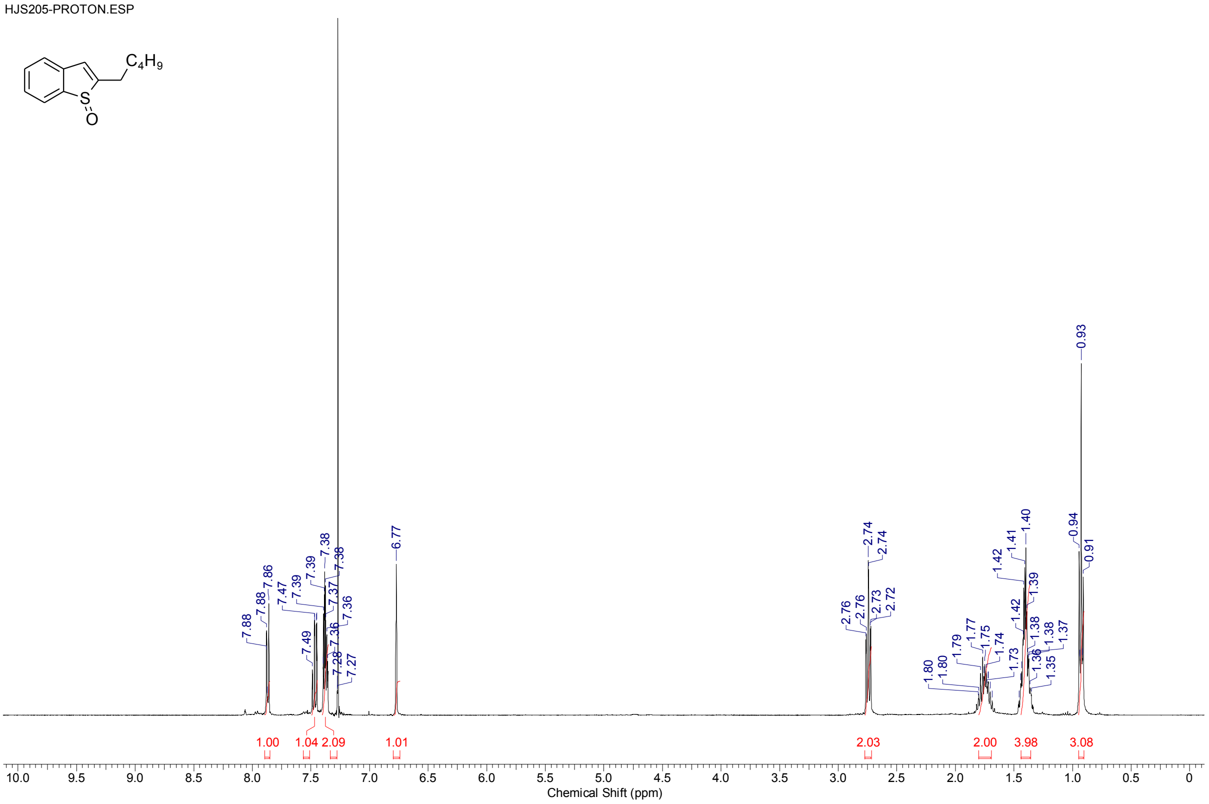

1f ^{13}C NMR (101 MHz, $CDCl_3$)

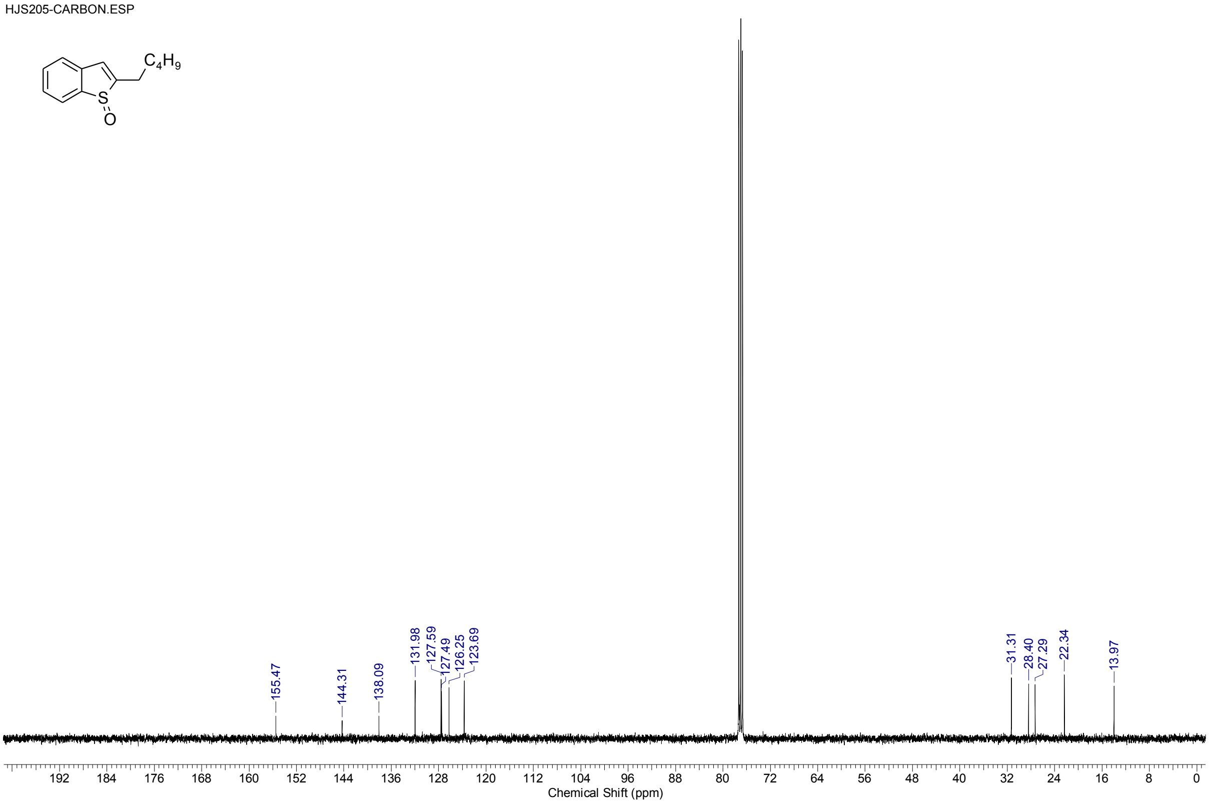

Supplementary Figure 7.

1g ^{1}H NMR (400 MHz, $CDCl_3$)

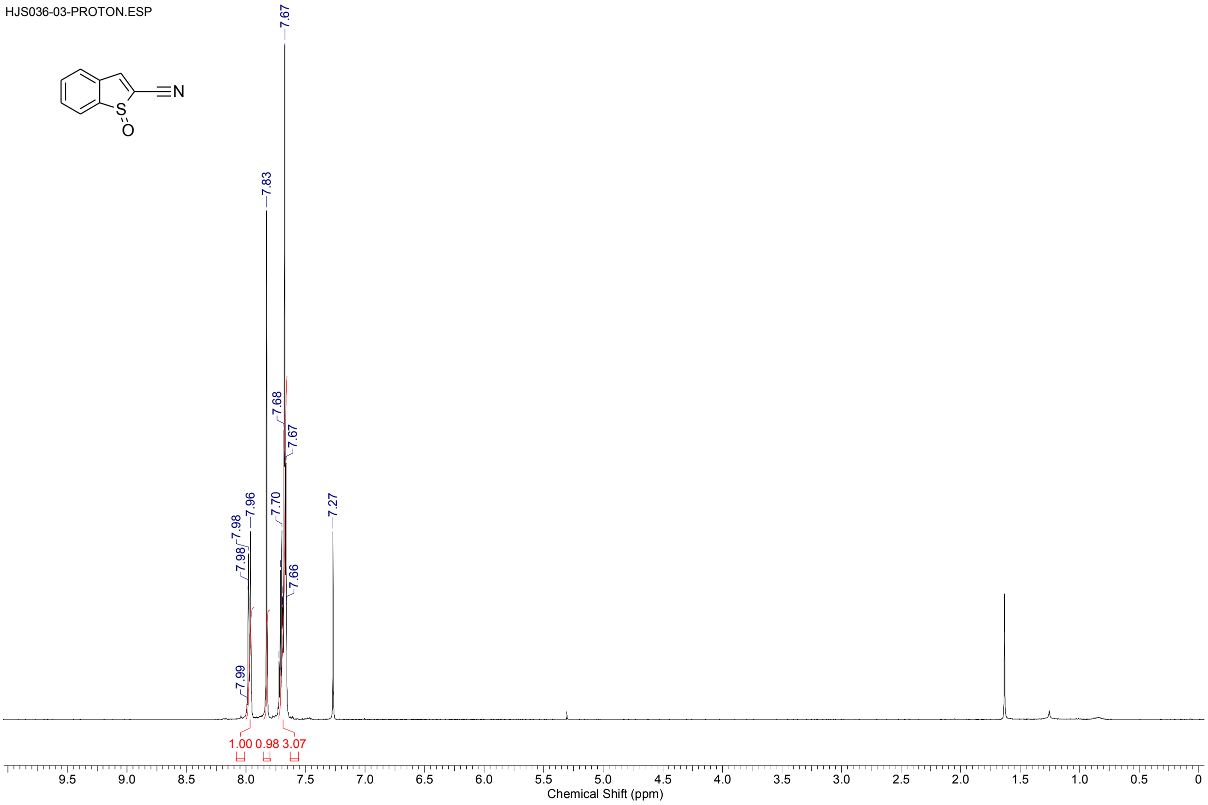

1g ^{13}C NMR (101 MHz, $CDCl_3$)

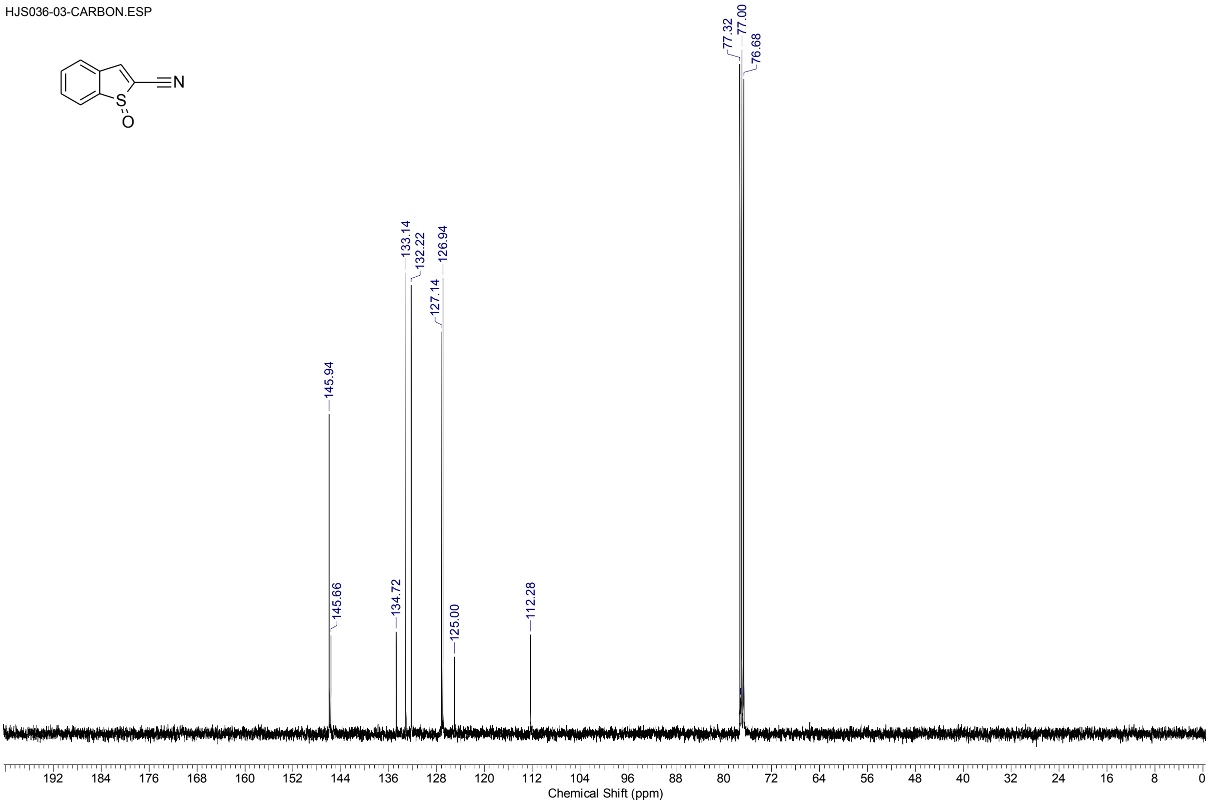

Supplementary Figure 8.

1h ^{1}H NMR (400 MHz, $CDCl_3$)

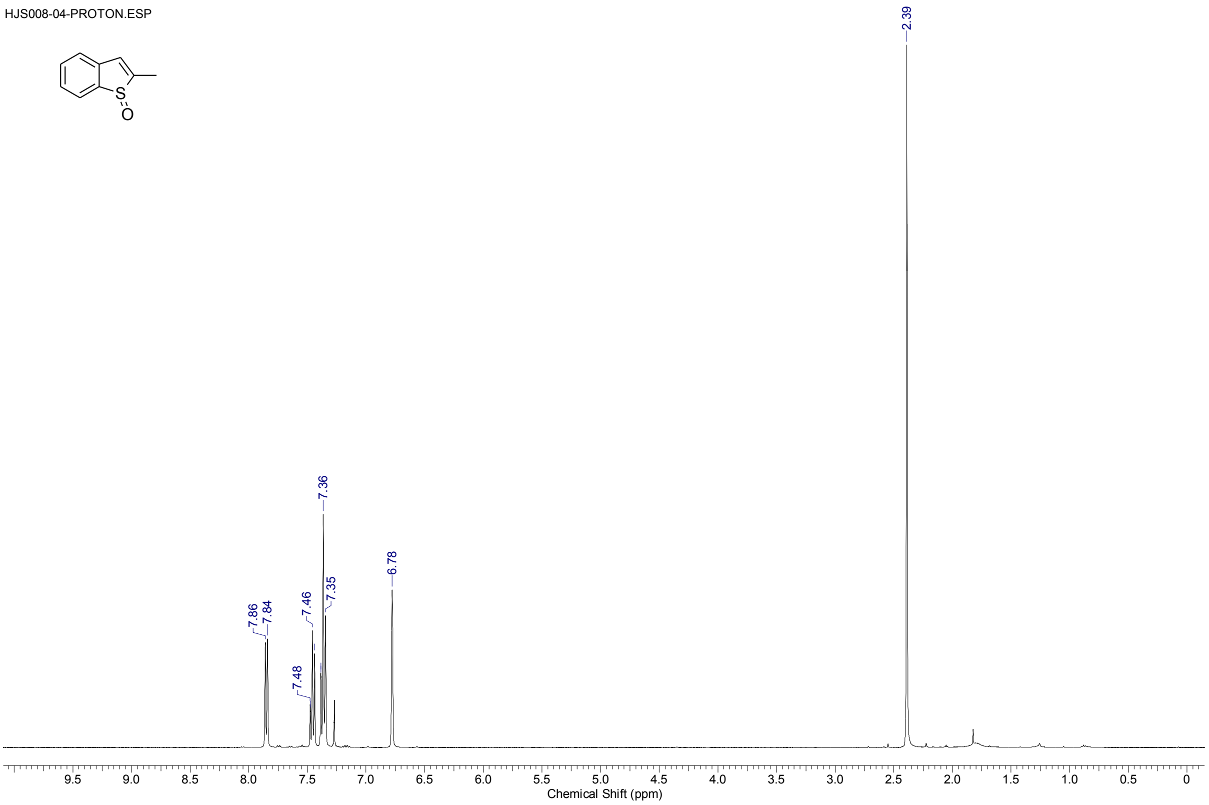

1h ^{13}C NMR (101 MHz, $CDCl_3$)

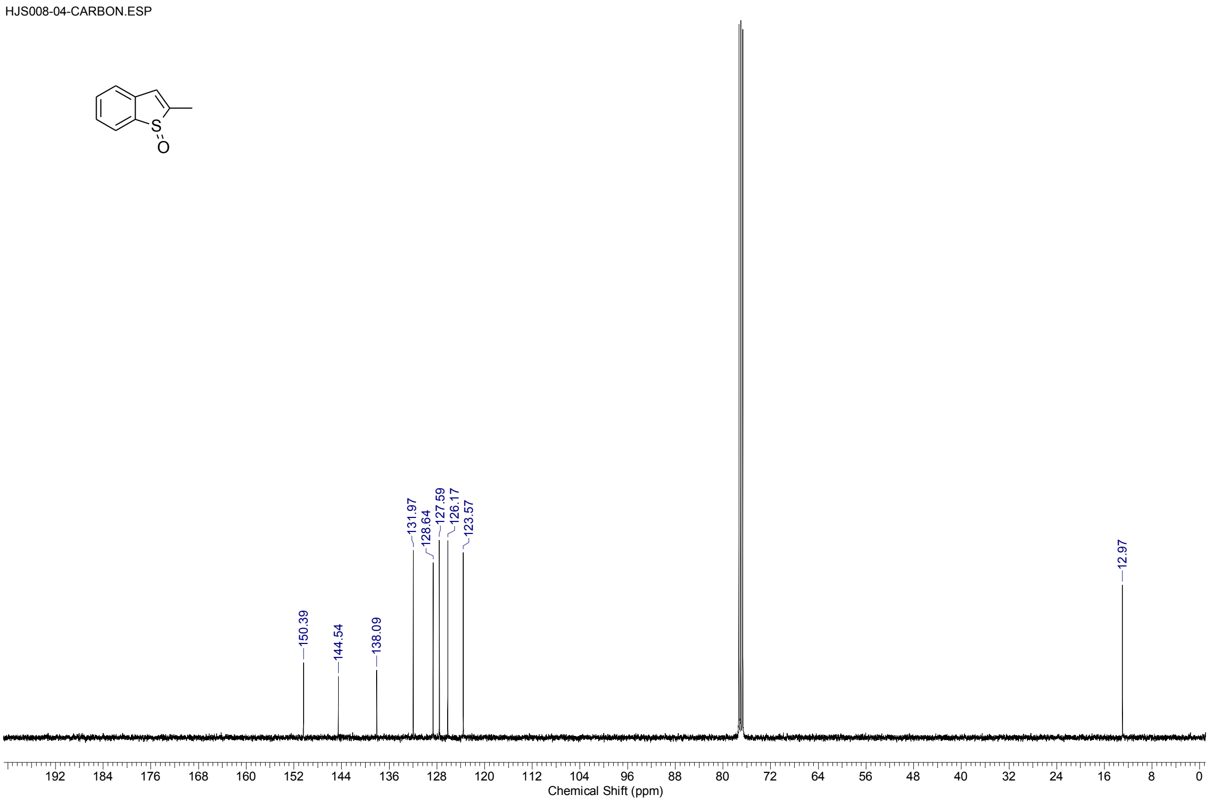

Supplementary Figure 9.

1i ^{1}H NMR (400 MHz, $CDCl_3$)

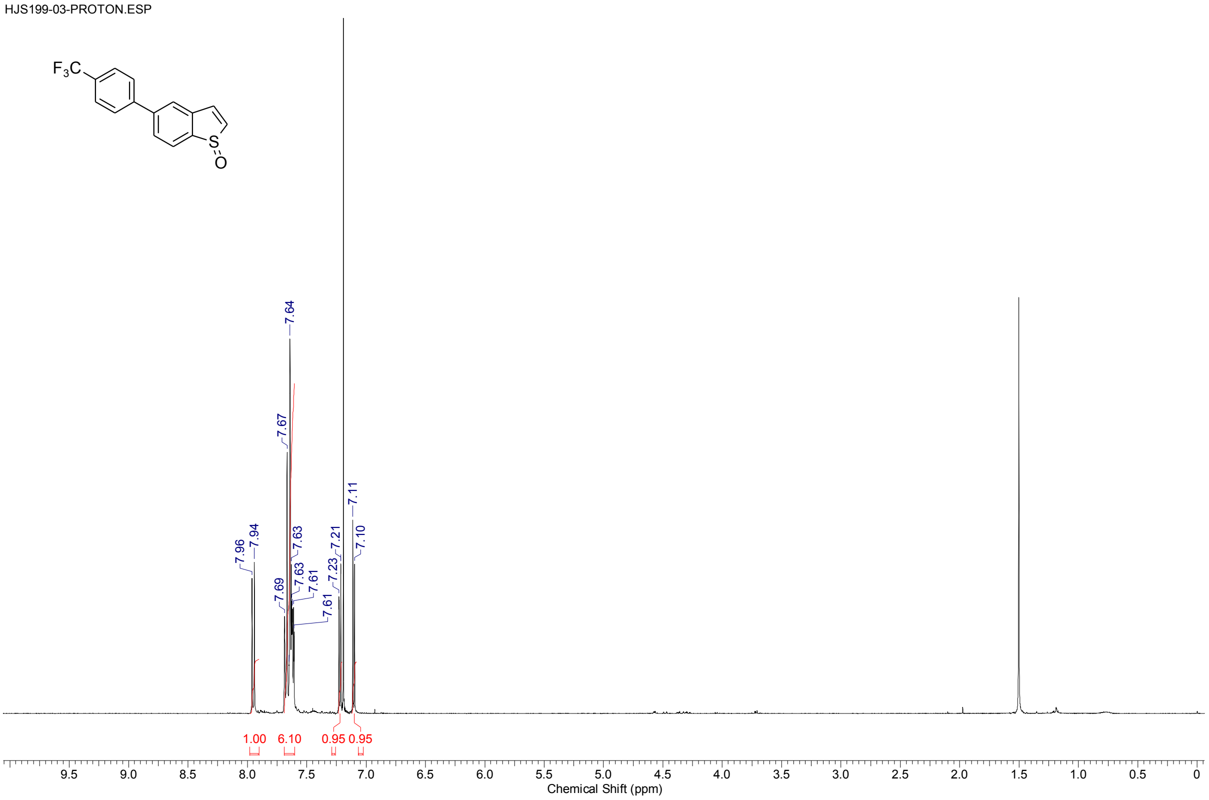

1i ^{13}C NMR (101 MHz, $CDCl_3$)

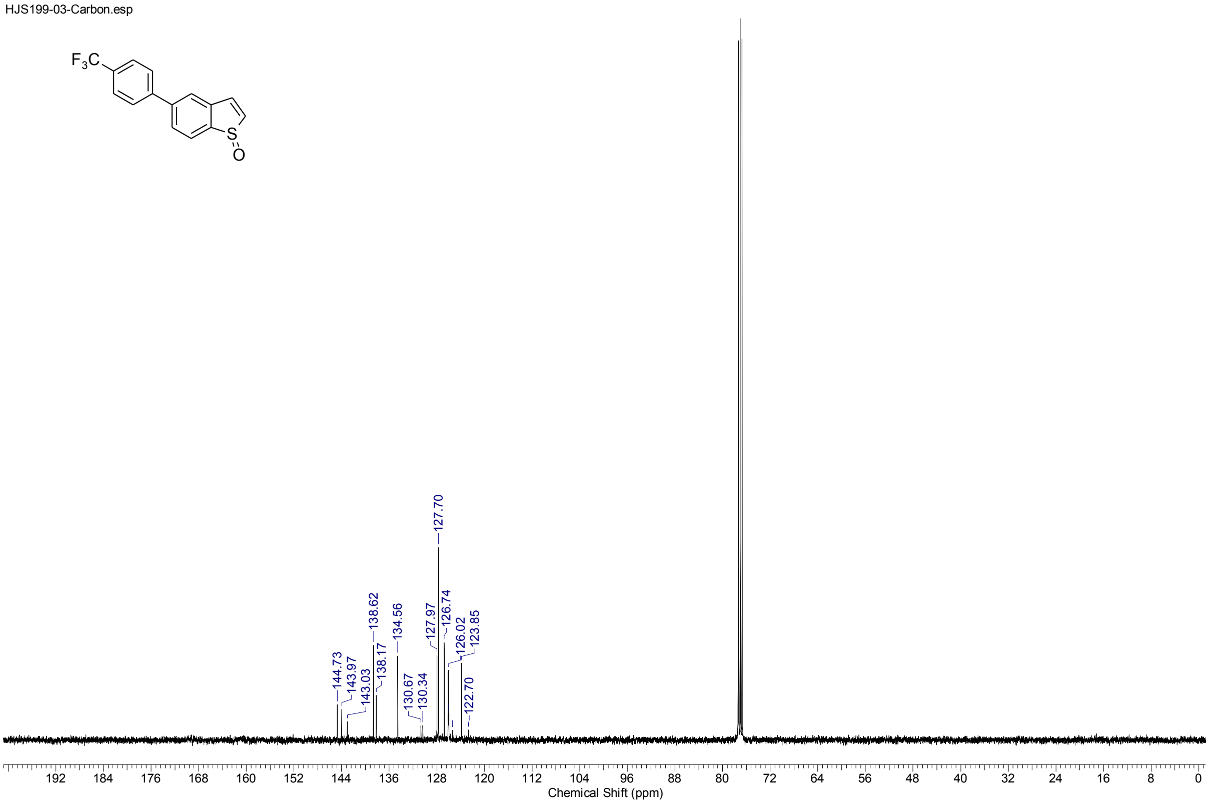

Supplementary Figure 10.

3a ^{1}H NMR (400 MHz, $CDCl_3$)

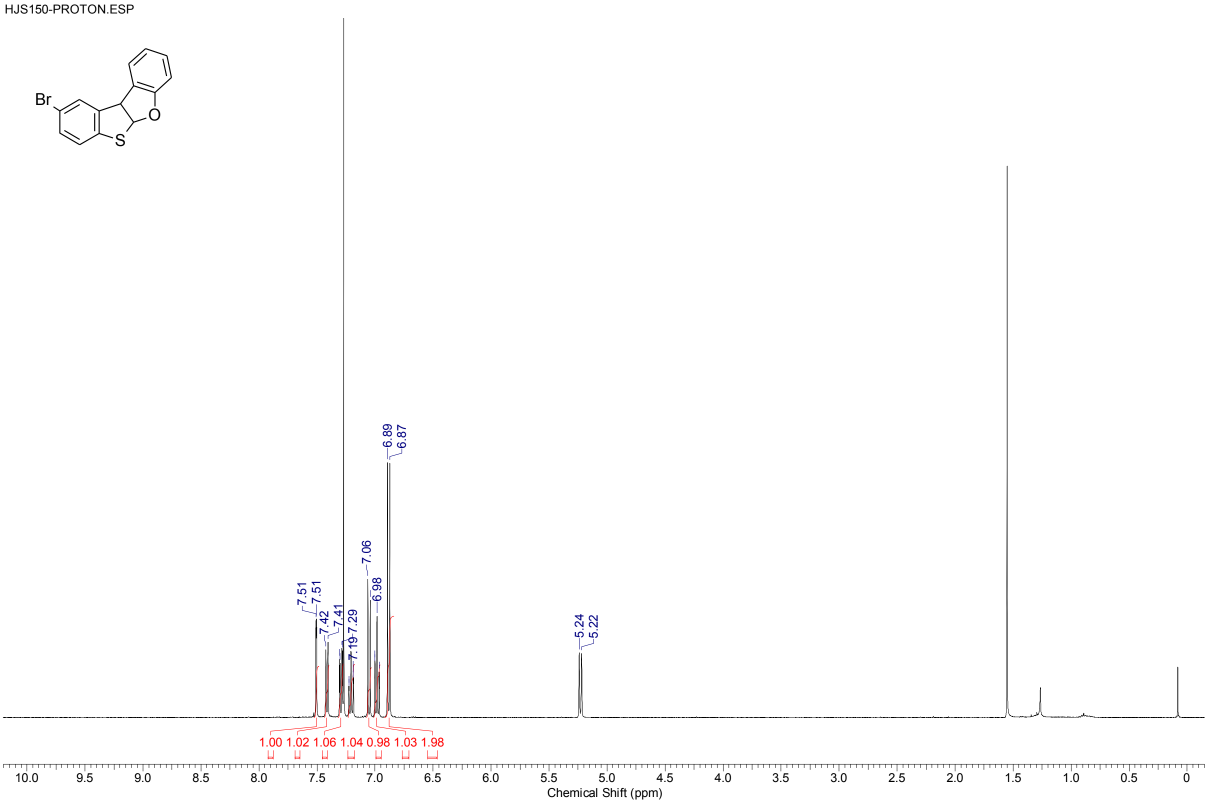

3a ^{13}C NMR (101 MHz, $CDCl_3$)

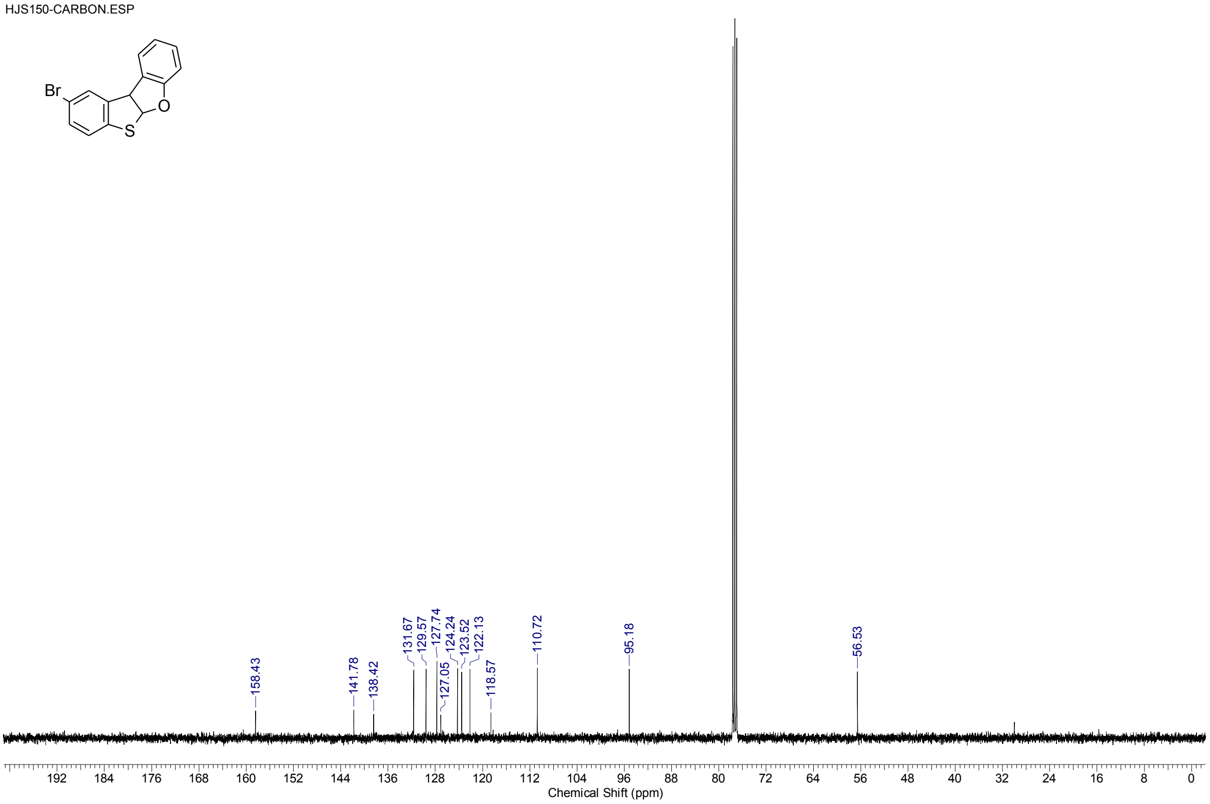

Supplementary Figure 11.

4a ^{1}H NMR (400 MHz, $CDCl_3$)

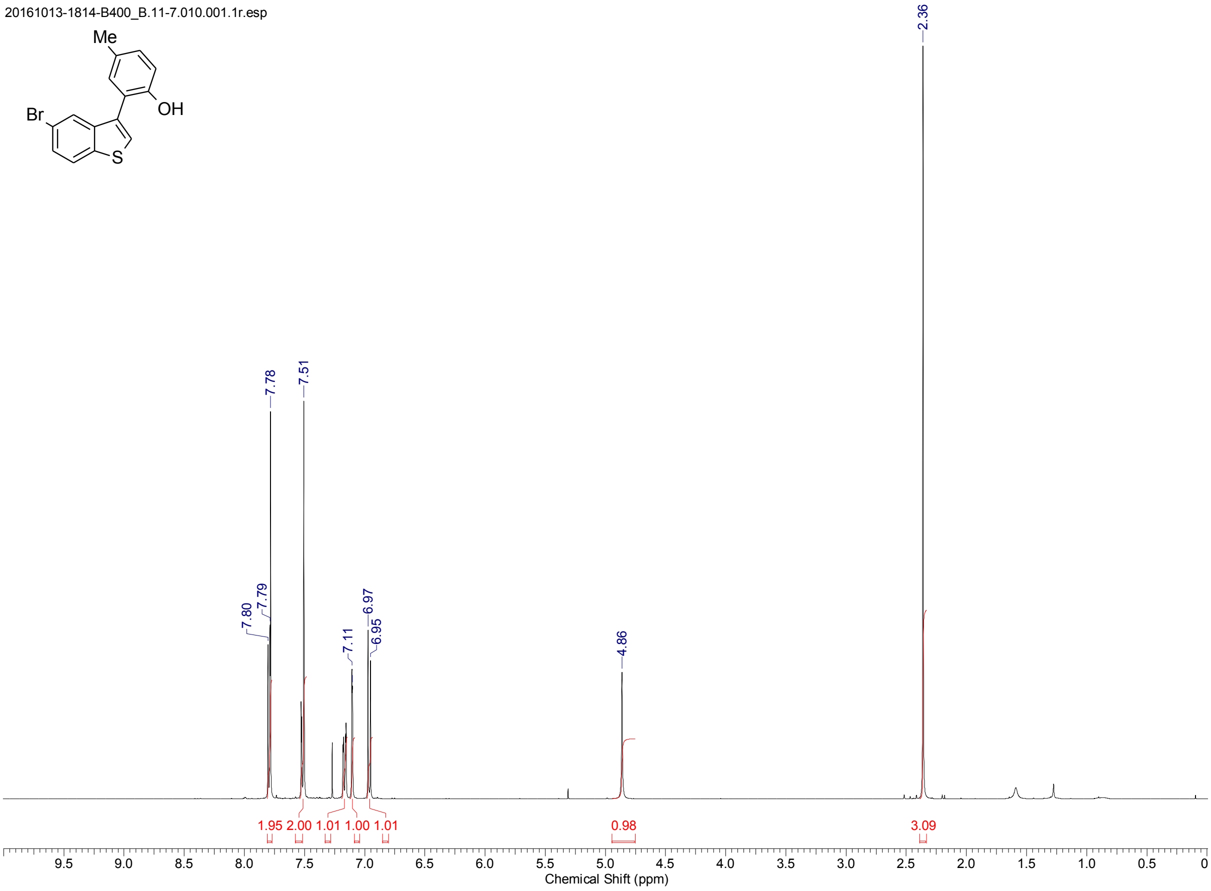

4a ^{13}C NMR (101 MHz, $CDCl_3$)

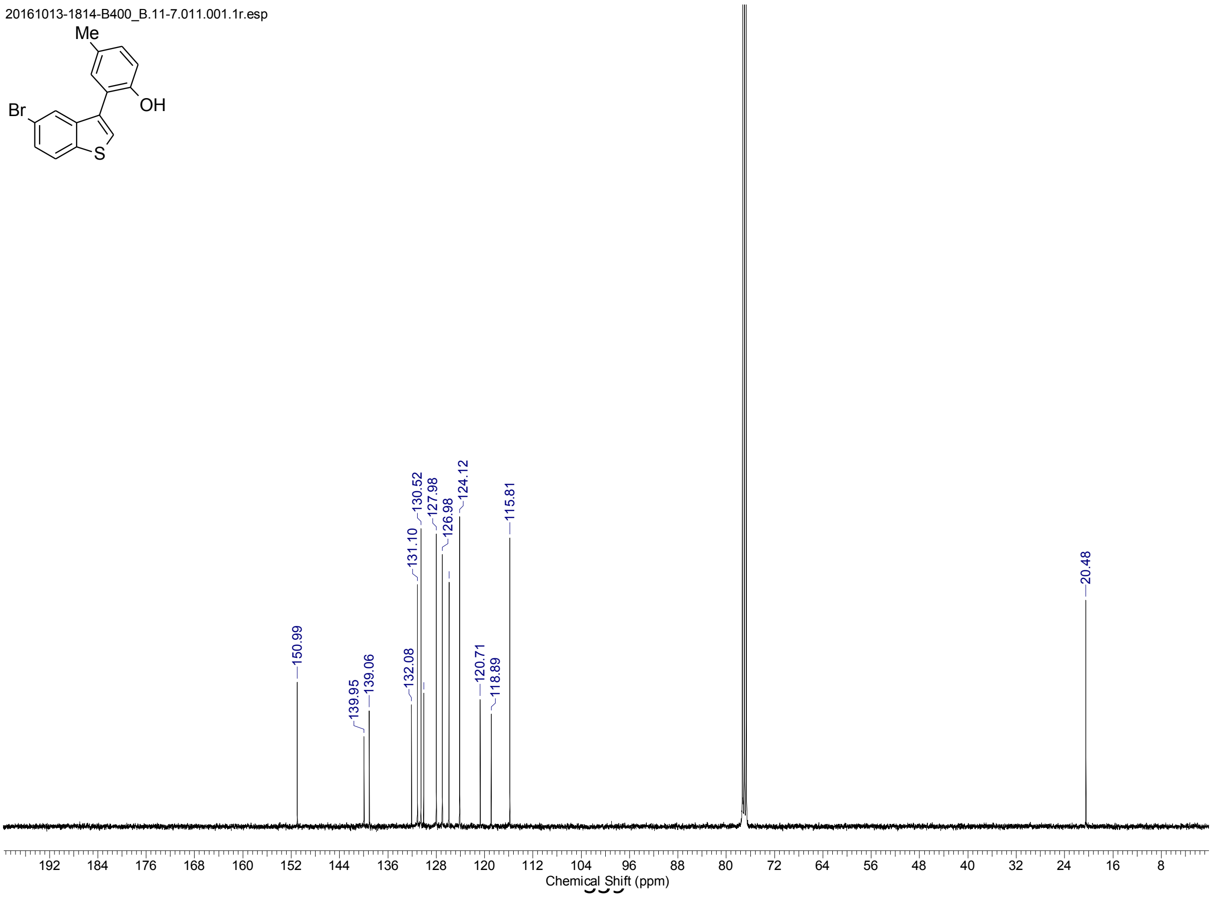

Supplementary Figure 12.

4b ^{1}H NMR (400 MHz, $CDCl_3$)

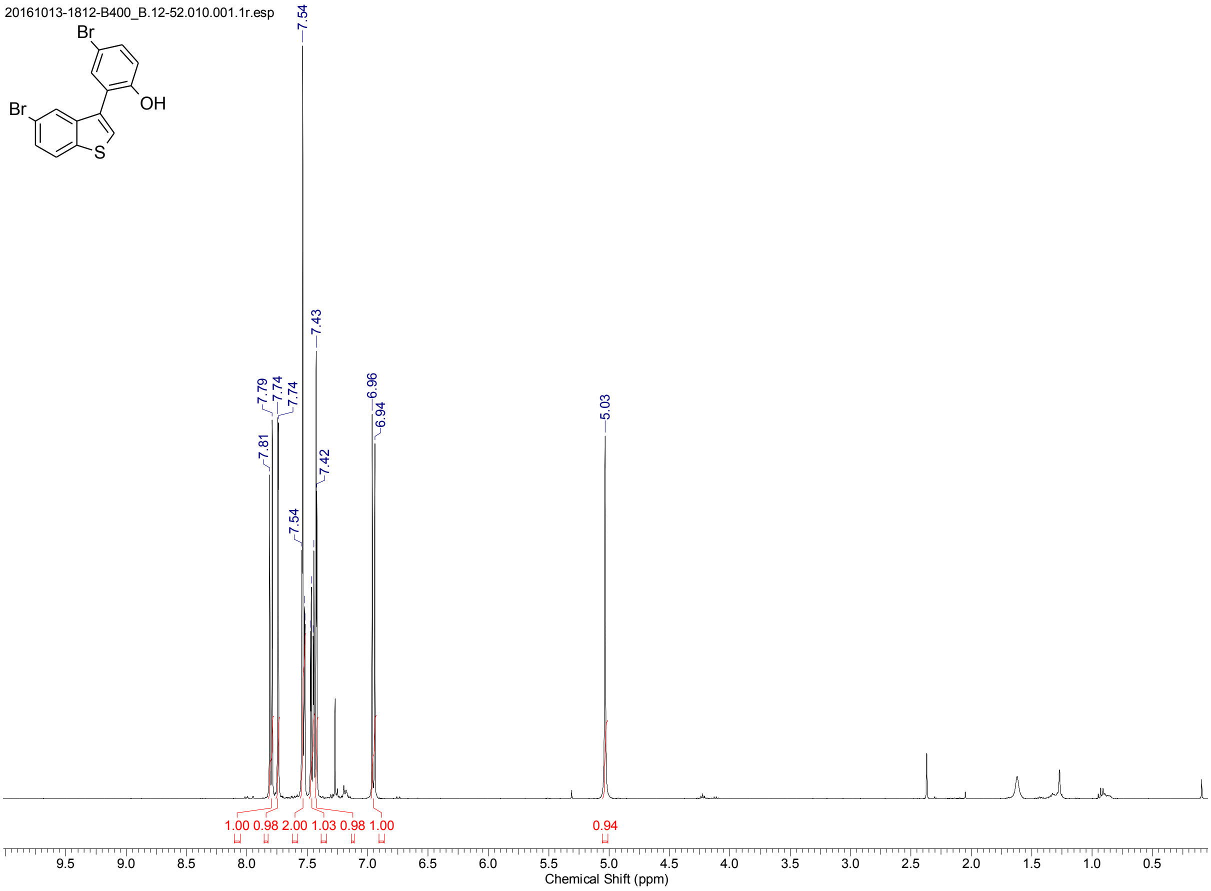

4b ^{13}C NMR (101 MHz, $CDCl_3$)

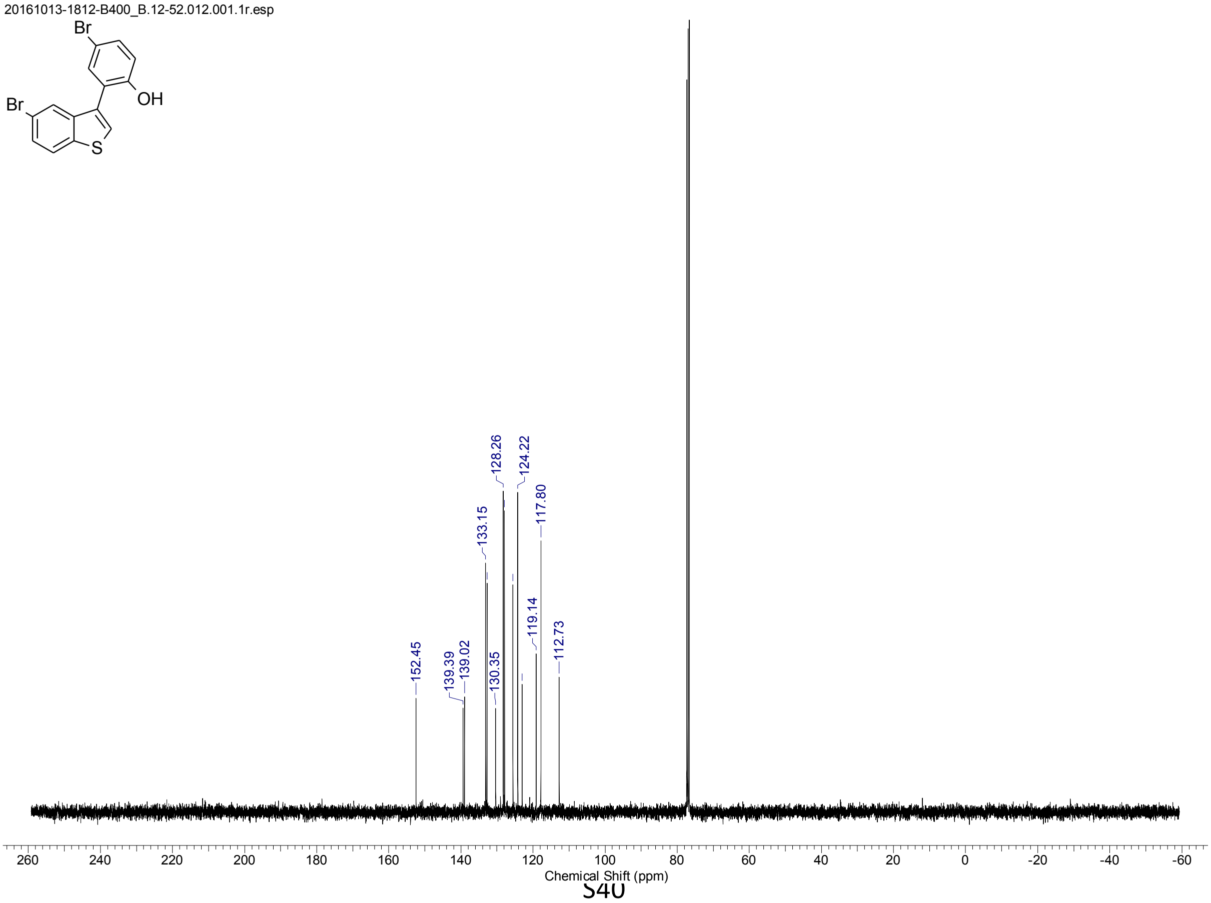

Supplementary Figure 13.

4c ^{1}H NMR (400 MHz, $CDCl_3$)

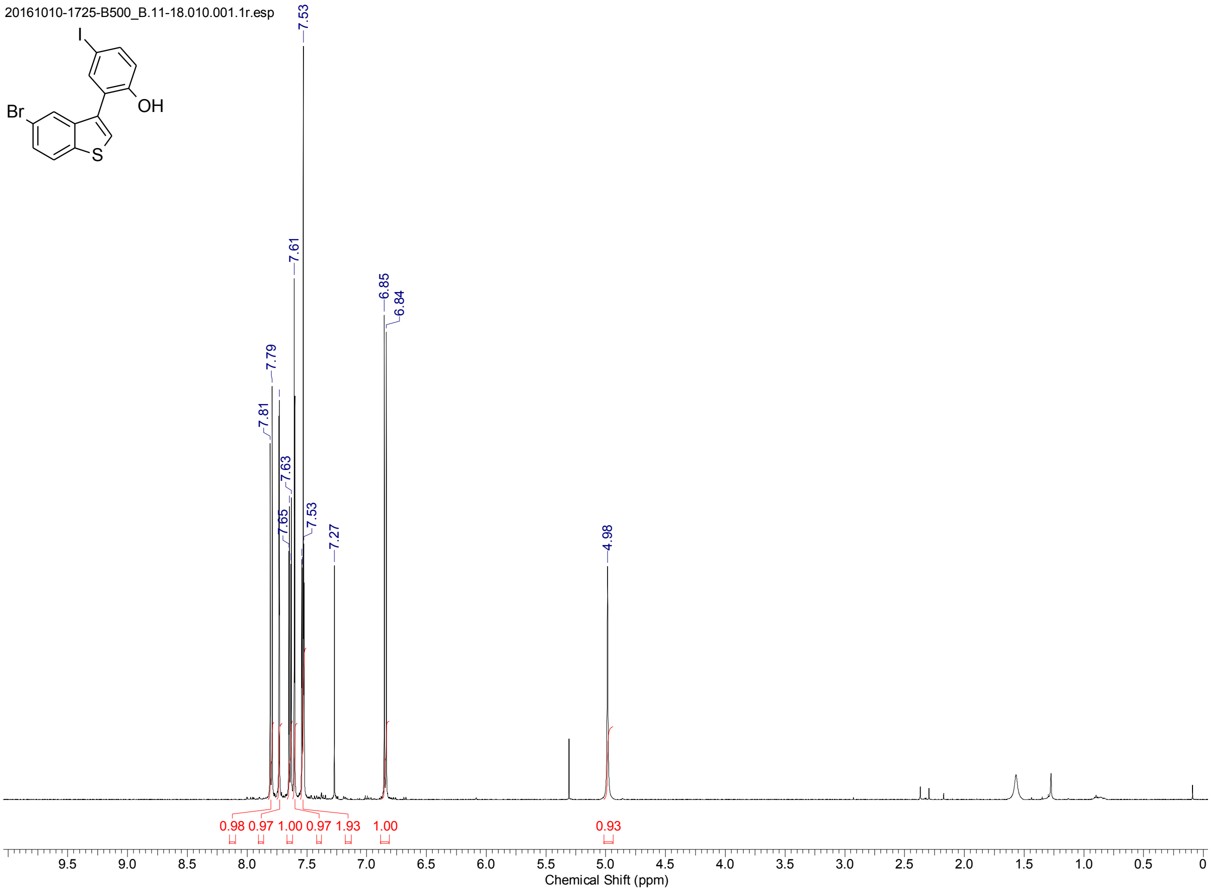

4c ^{13}C NMR (101 MHz, $CDCl_3$)

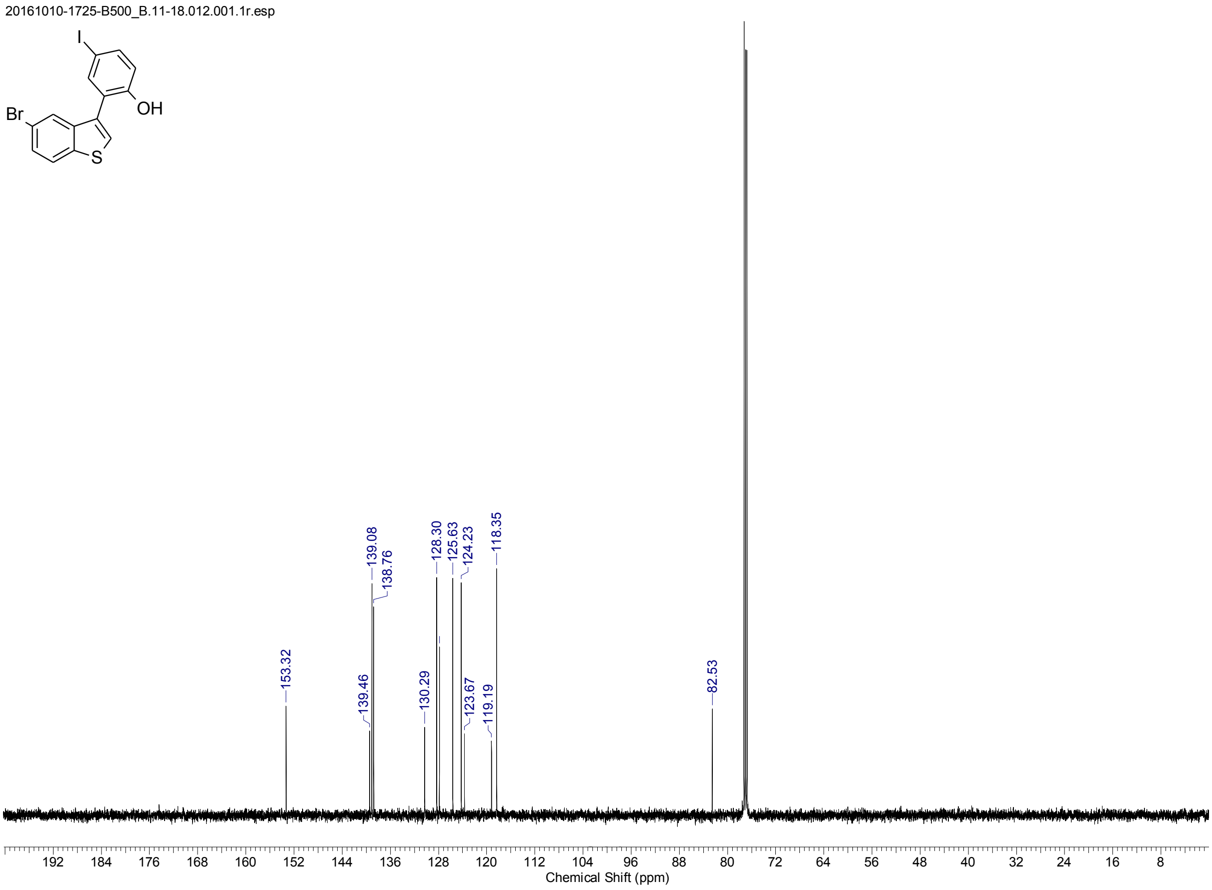

Supplementary Figure 14.

4d ^{1}H NMR (400 MHz, $CDCl_3$)

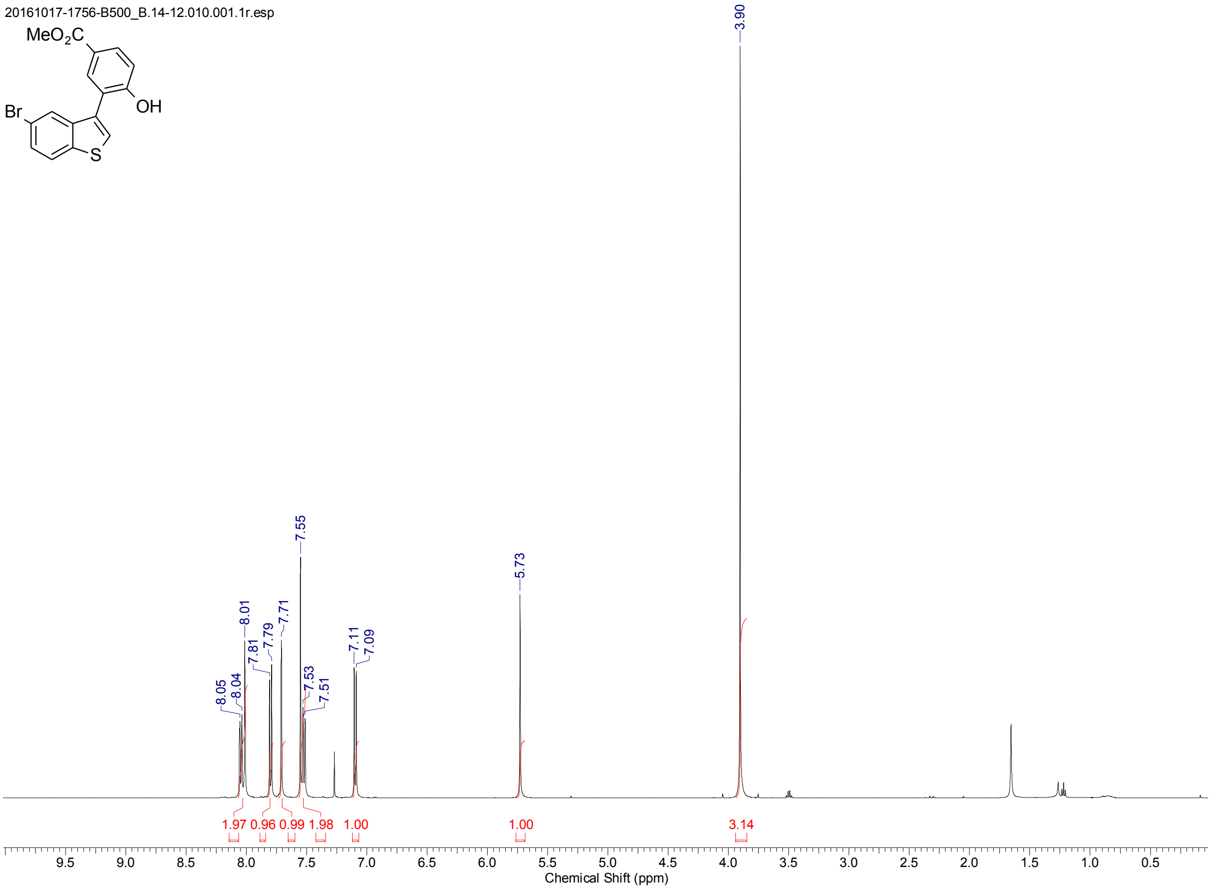

4d ^{13}C NMR (101 MHz, $CDCl_3$)

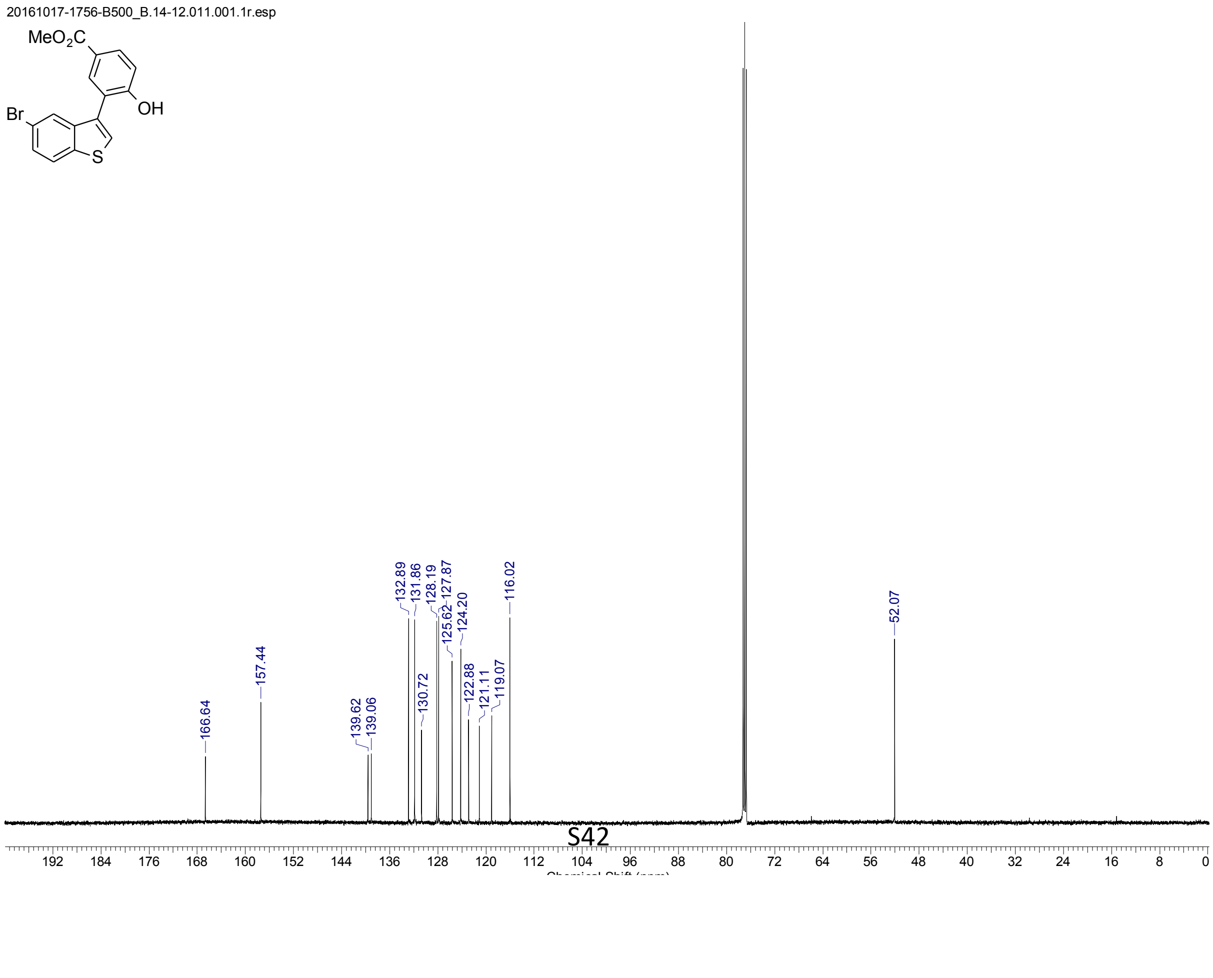

Supplementary Figure 15.

4e ^{1}H NMR (400 MHz, $CDCl_3$)

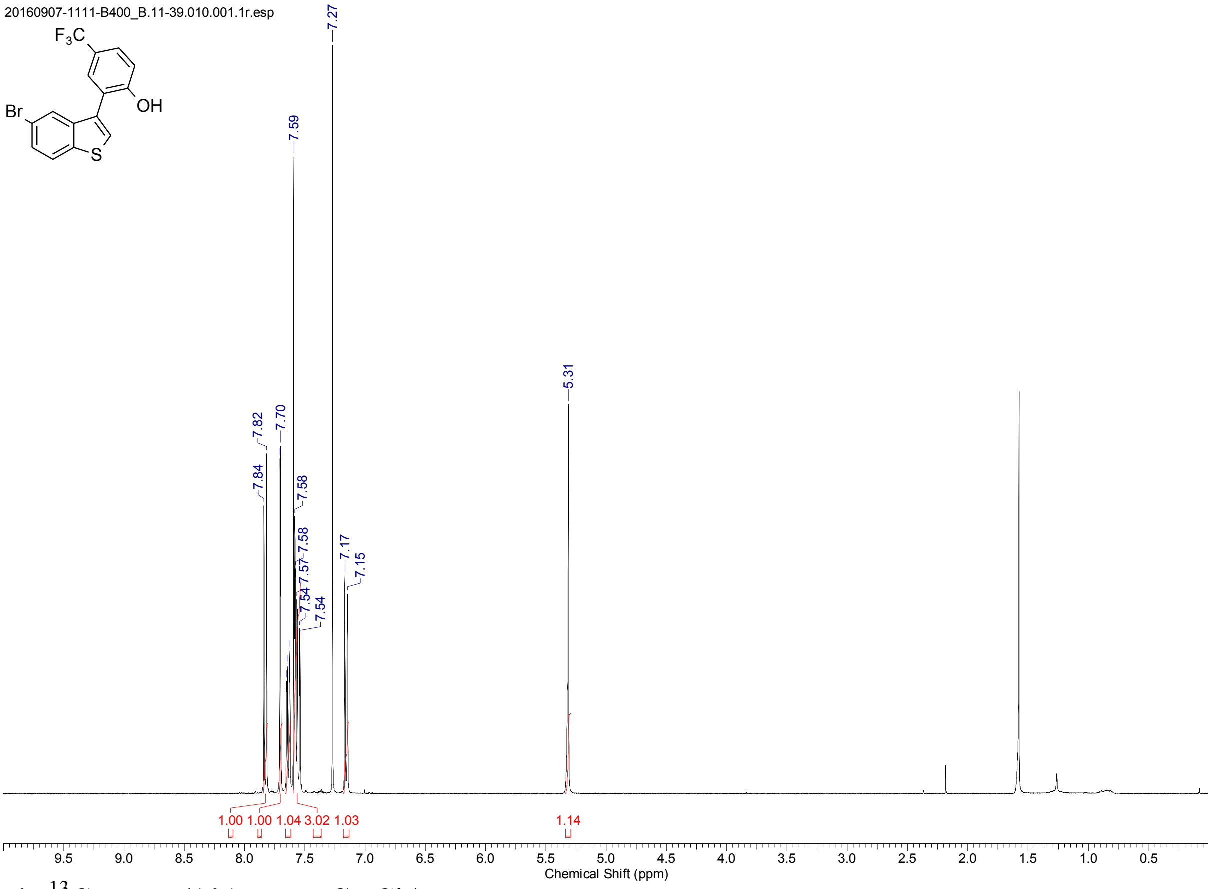

4e ^{13}C NMR (101 MHz, $CDCl_3$)

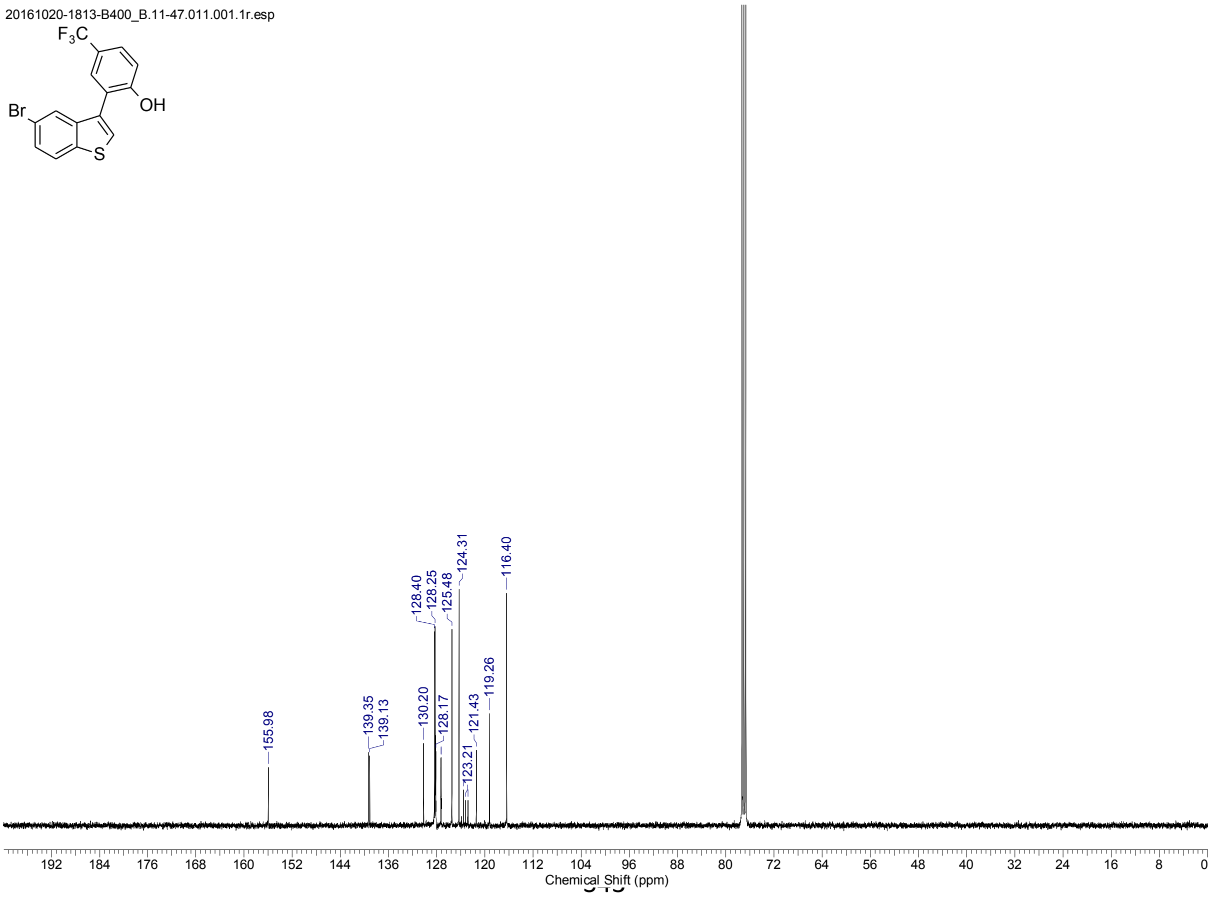

Supplementary Figure 16.

4f ^{1}H NMR (400 MHz, $CDCl_3$)

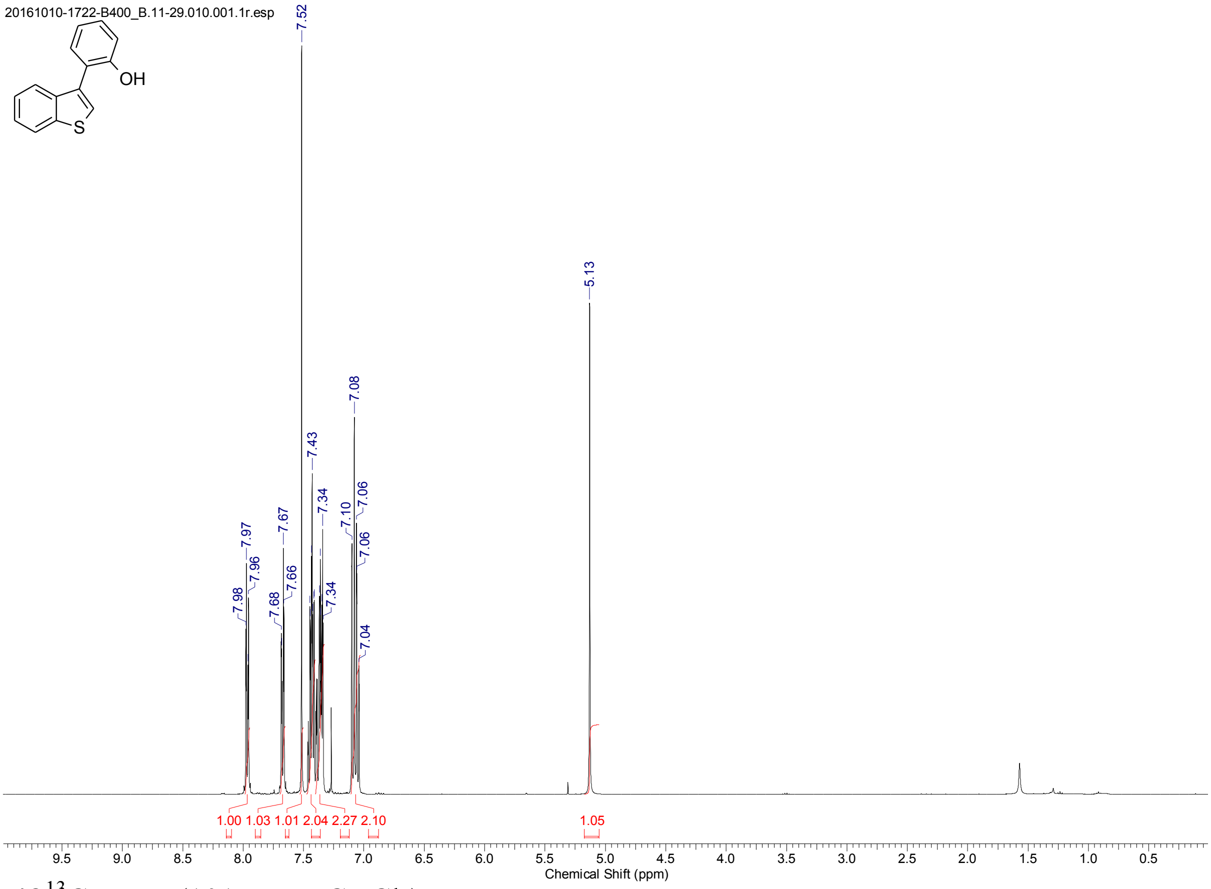

4f ^{13}C NMR (101 MHz, $CDCl_3$)

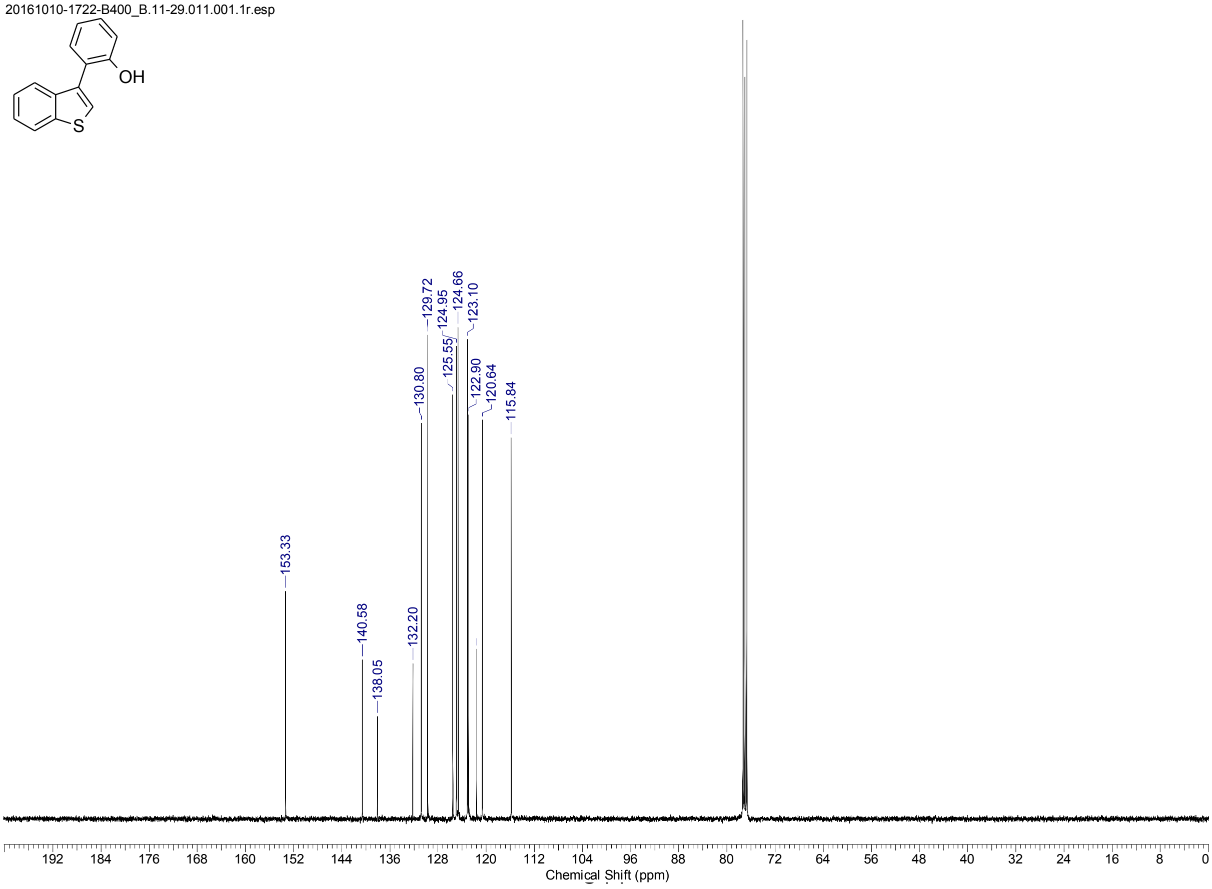

Supplementary Figure 17.

4g ^{1}H NMR (400 MHz, $CDCl_3$)

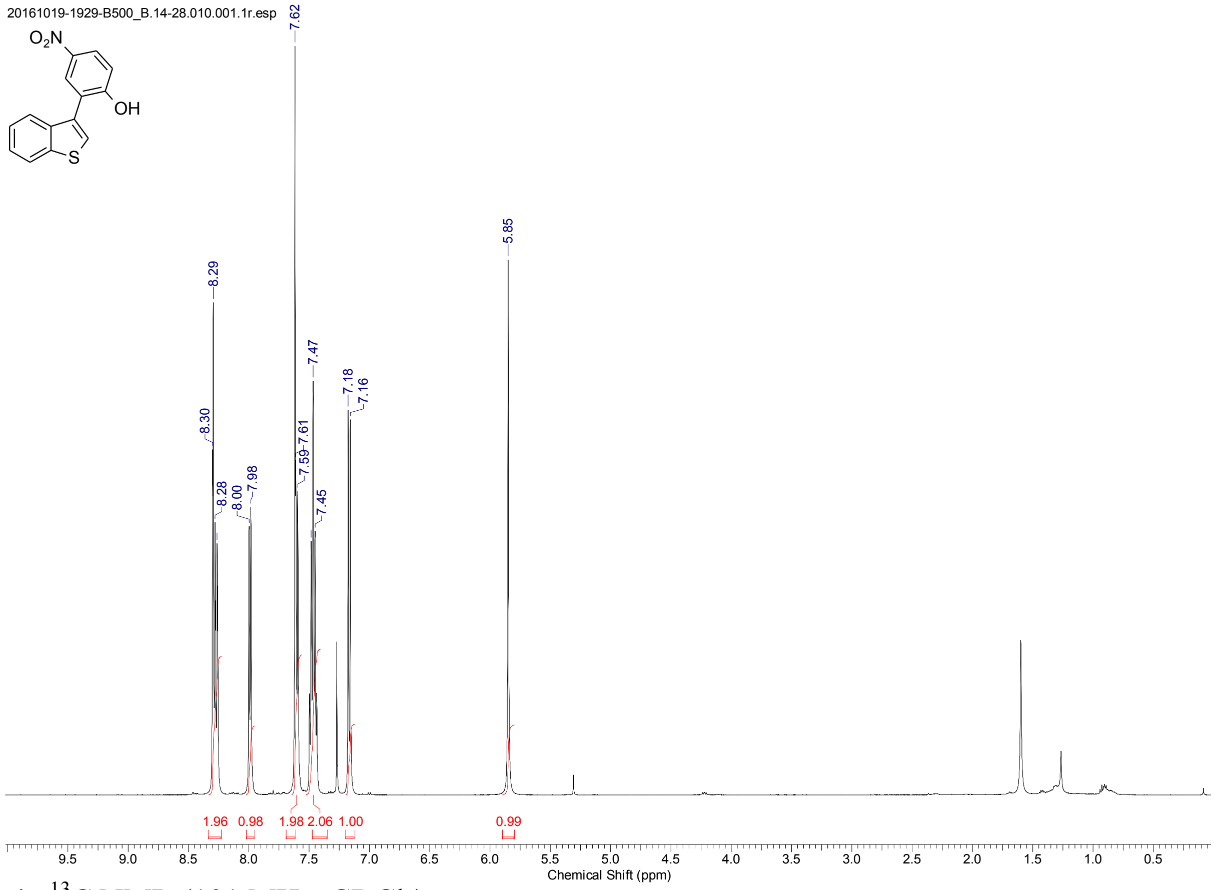

4g ^{13}C NMR (101 MHz, $CDCl_3$)

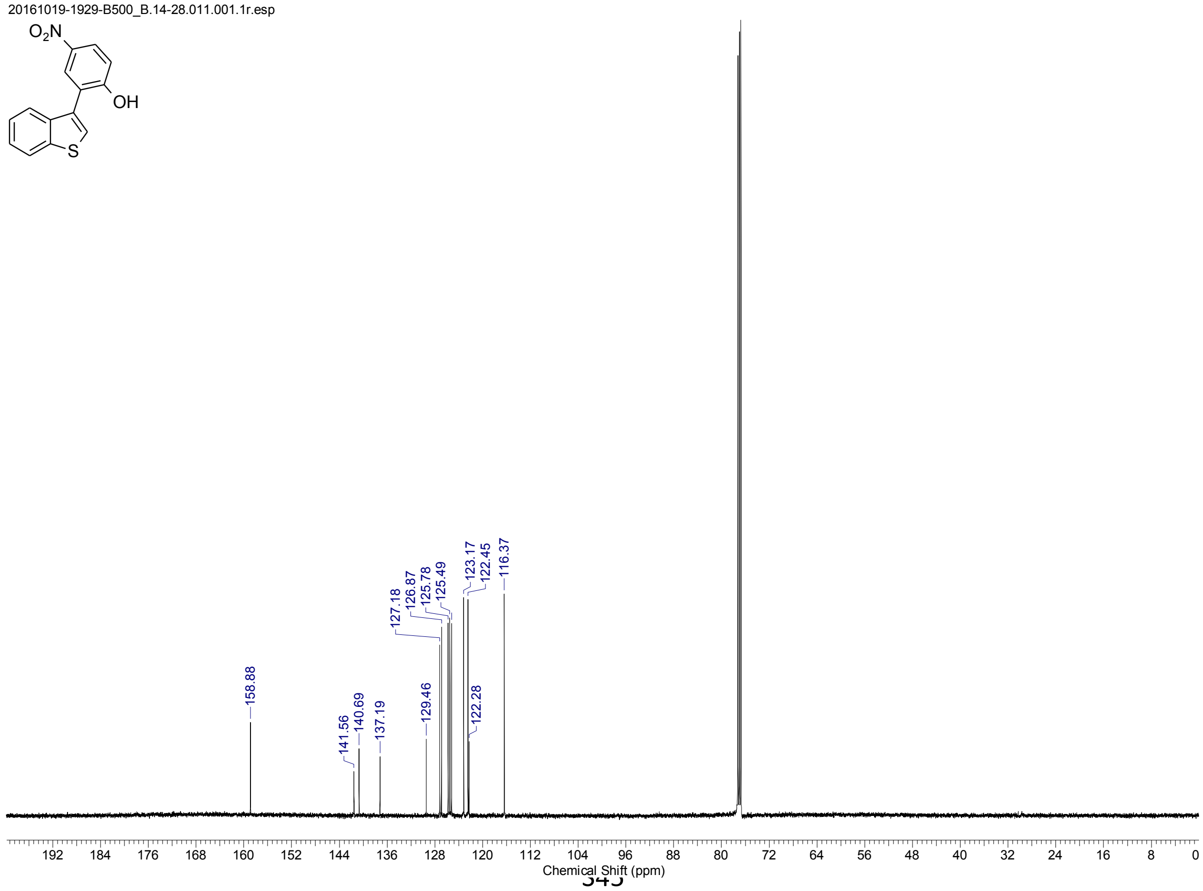

Supplementary Figure 18.

4h ^{1}H NMR (400 MHz, $CDCl_3$)

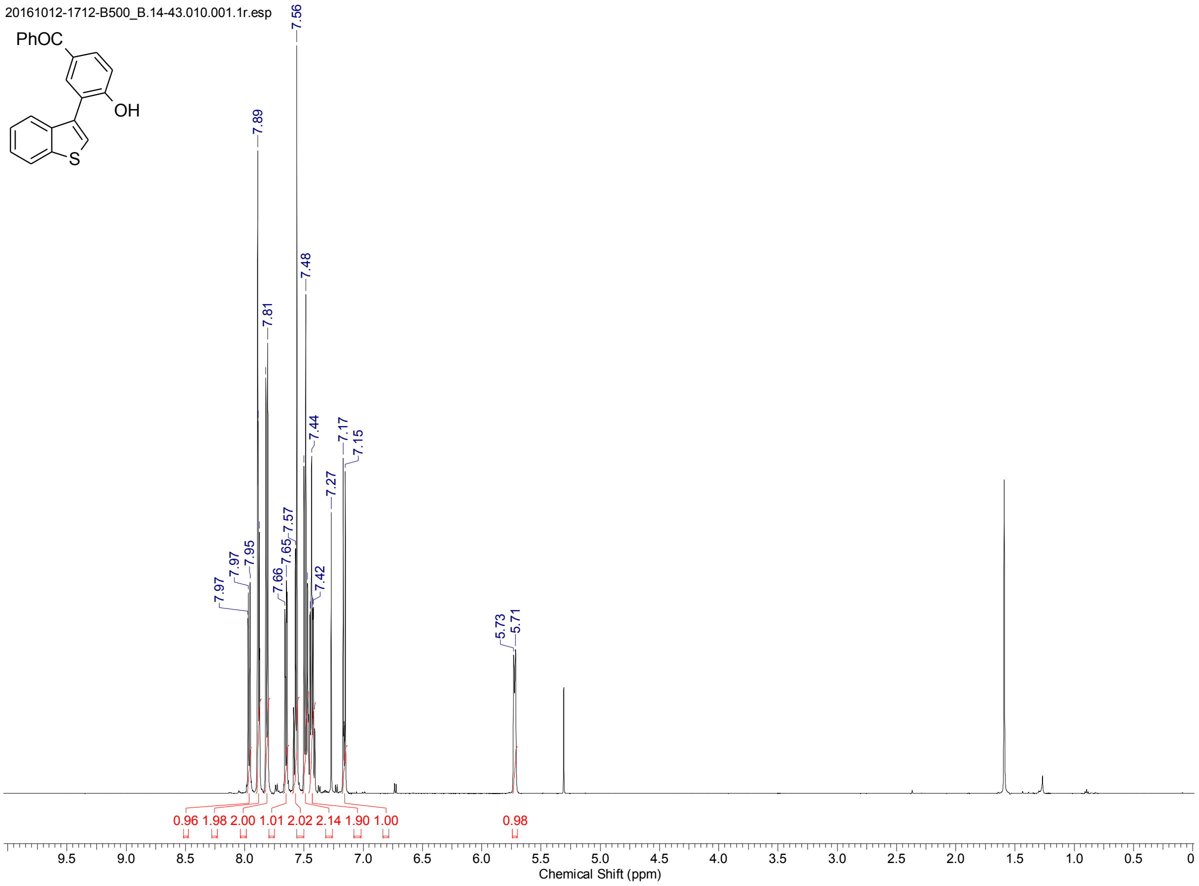

4h ^{13}C NMR (101 MHz, $CDCl_3$)

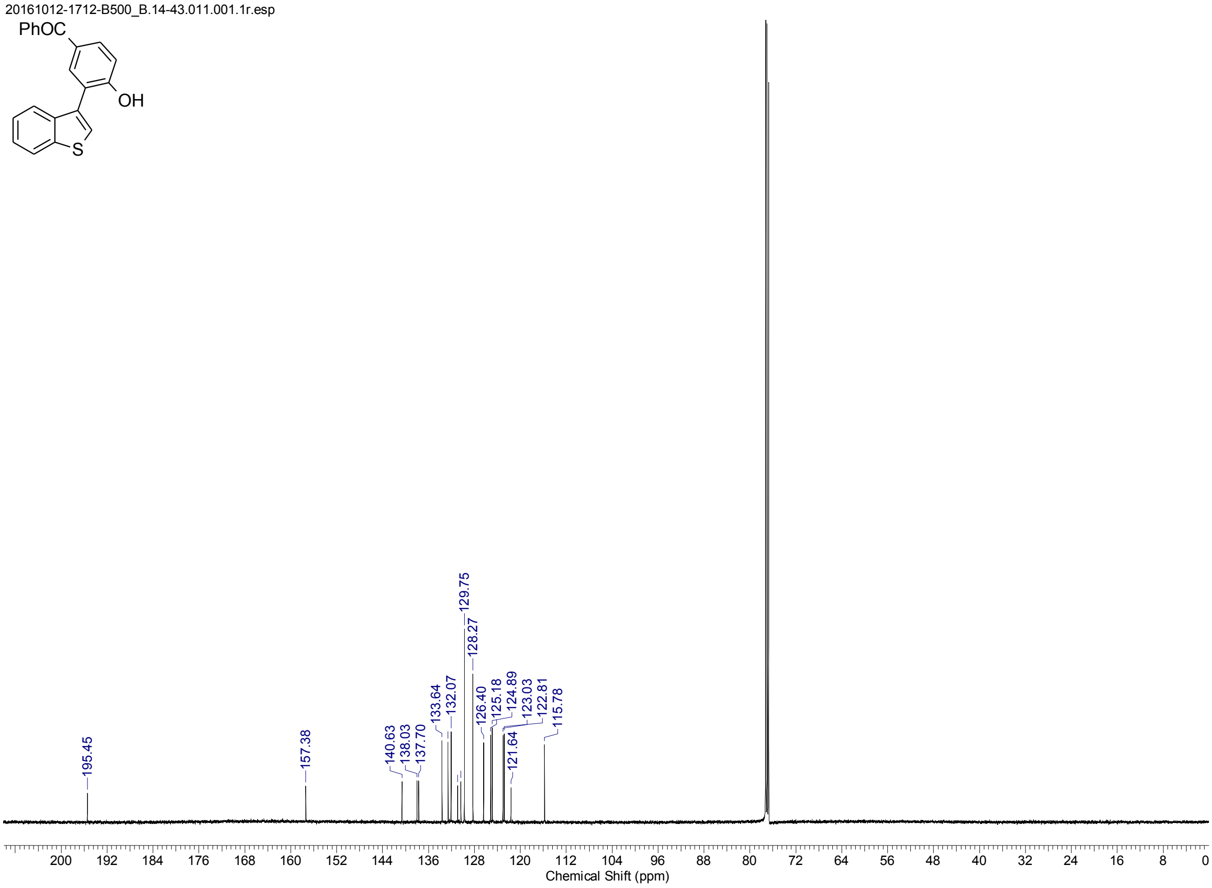

Supplementary Figure 19.

4i ^{1}H NMR (400 MHz, $CDCl_3$)

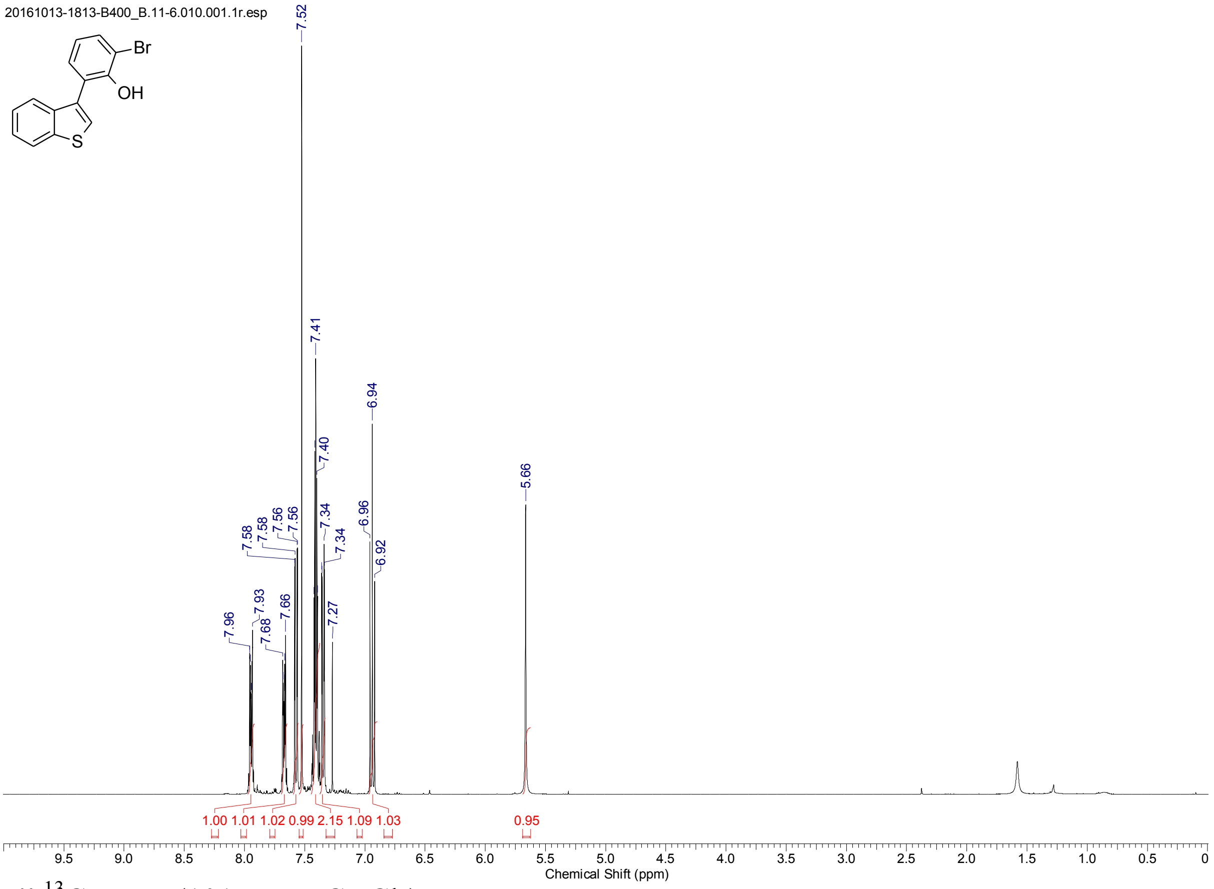

4i ^{13}C NMR (101 MHz, $CDCl_3$)

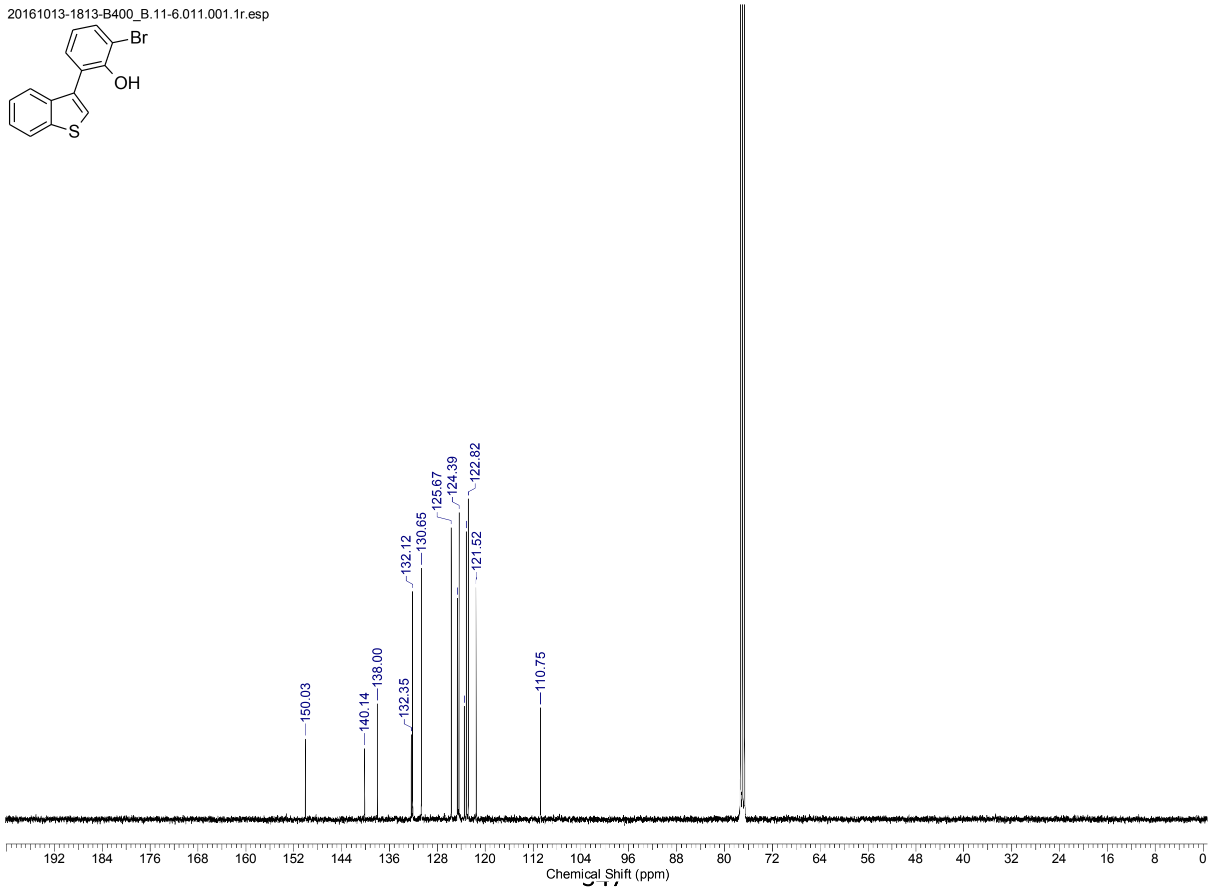

Supplementary Figure 20.

4j ^{1}H NMR (400 MHz, $CDCl_3$)

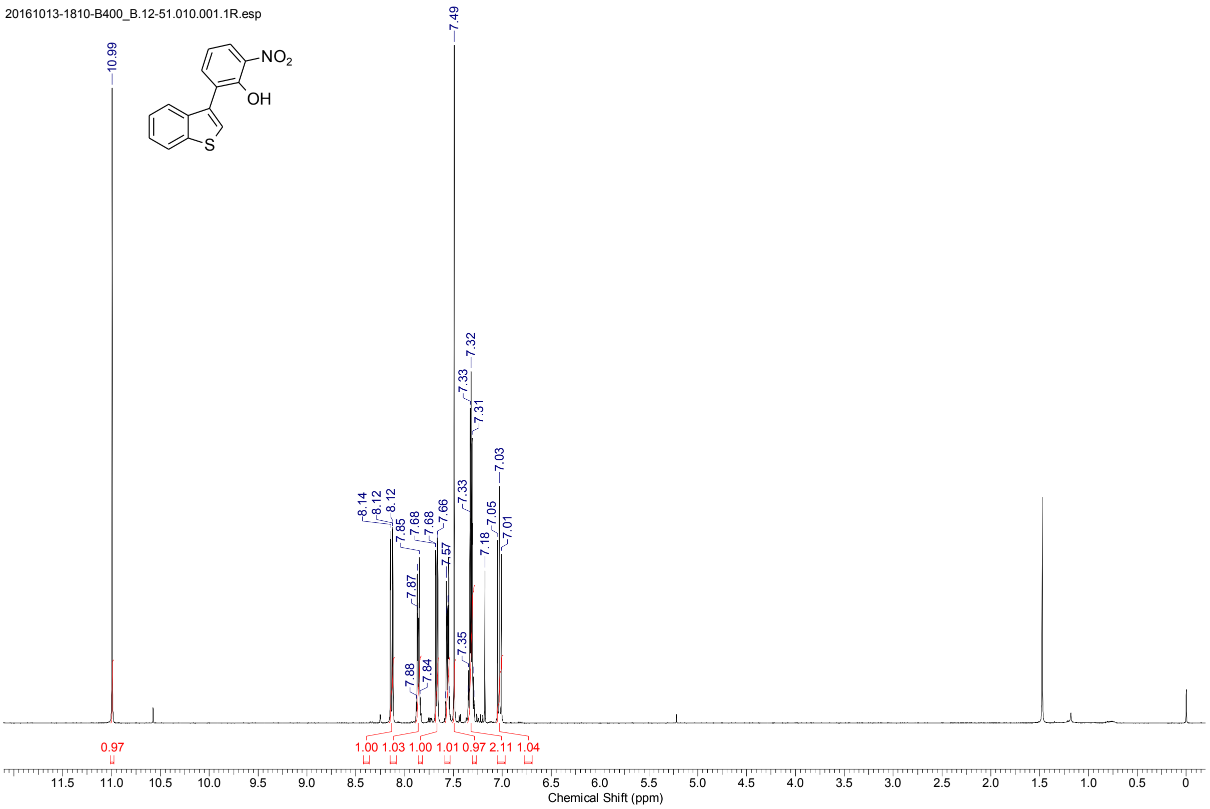

4j ^{13}C NMR (101 MHz, $CDCl_3$)

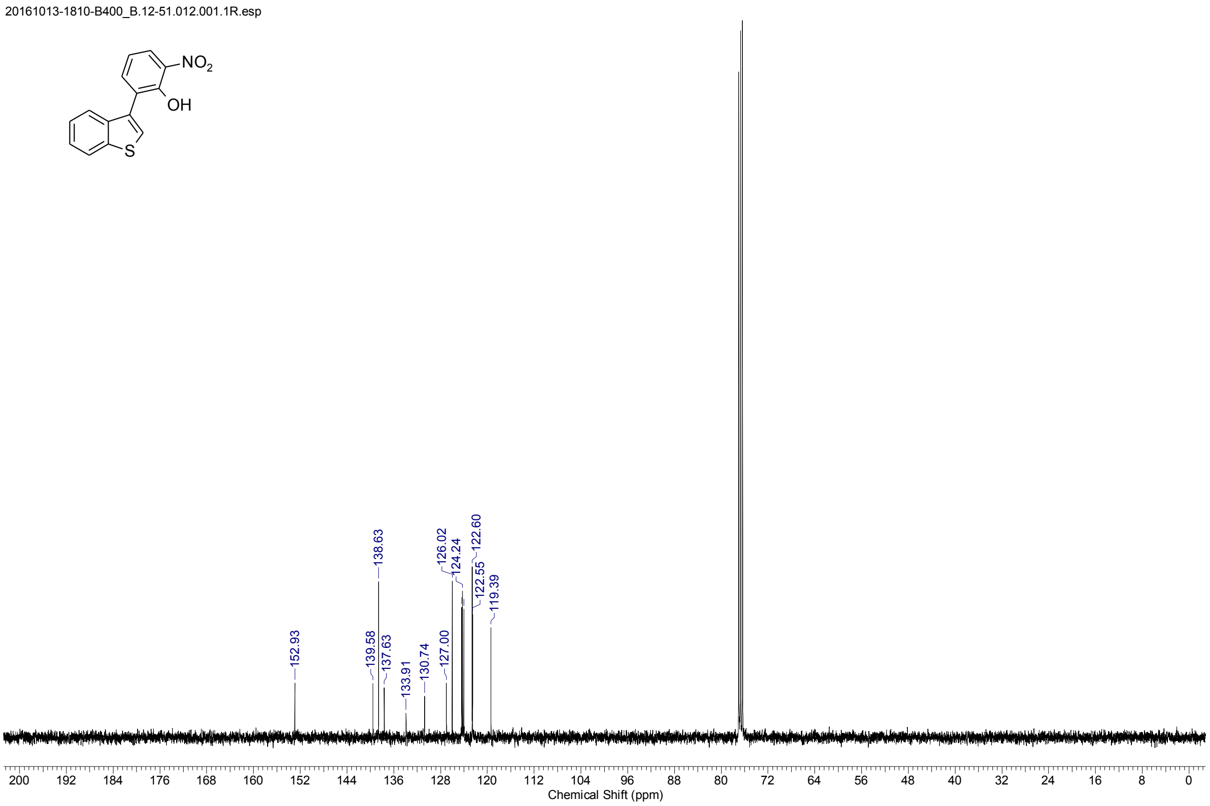

Supplementary Figure 21.

4k ^{1}H NMR (400 MHz, $CDCl_3$)

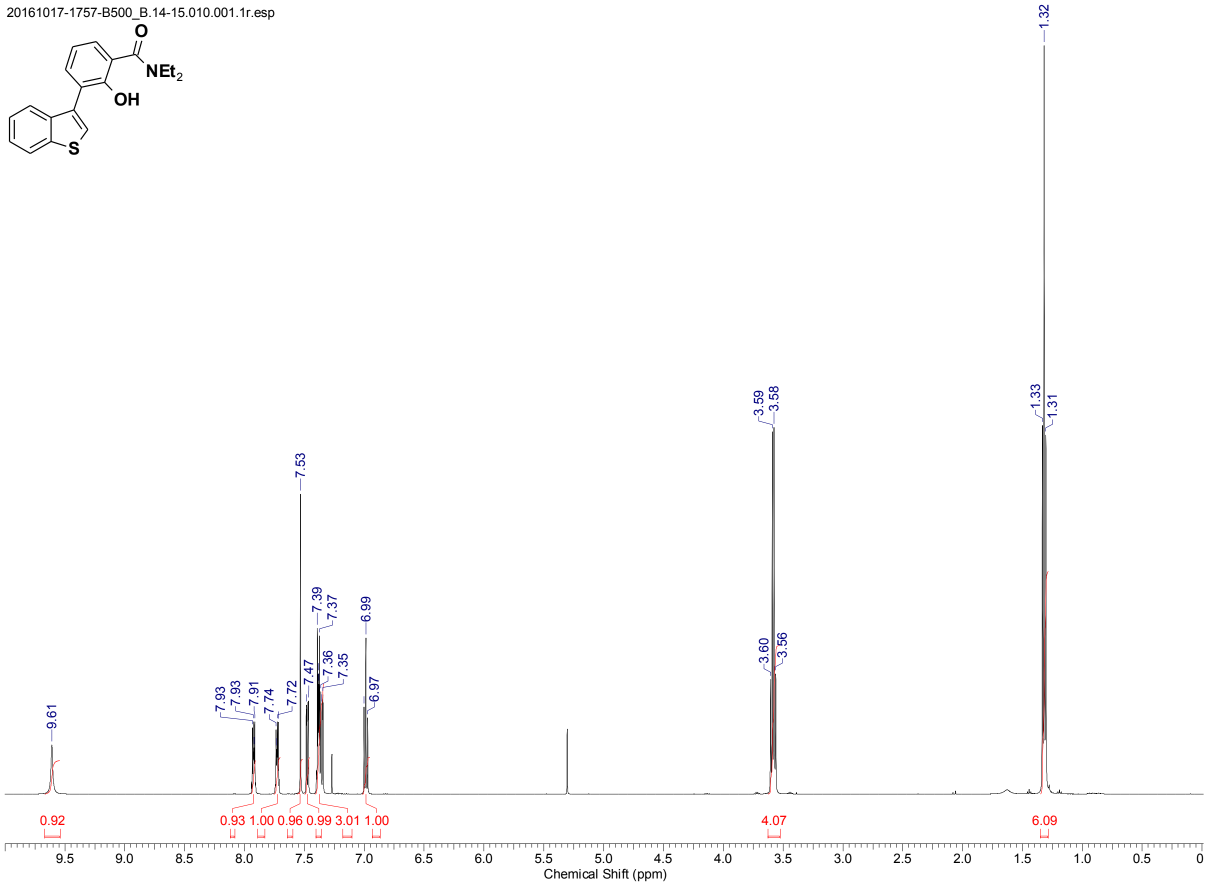

4k ^{13}C NMR (101 MHz, $CDCl_3$)

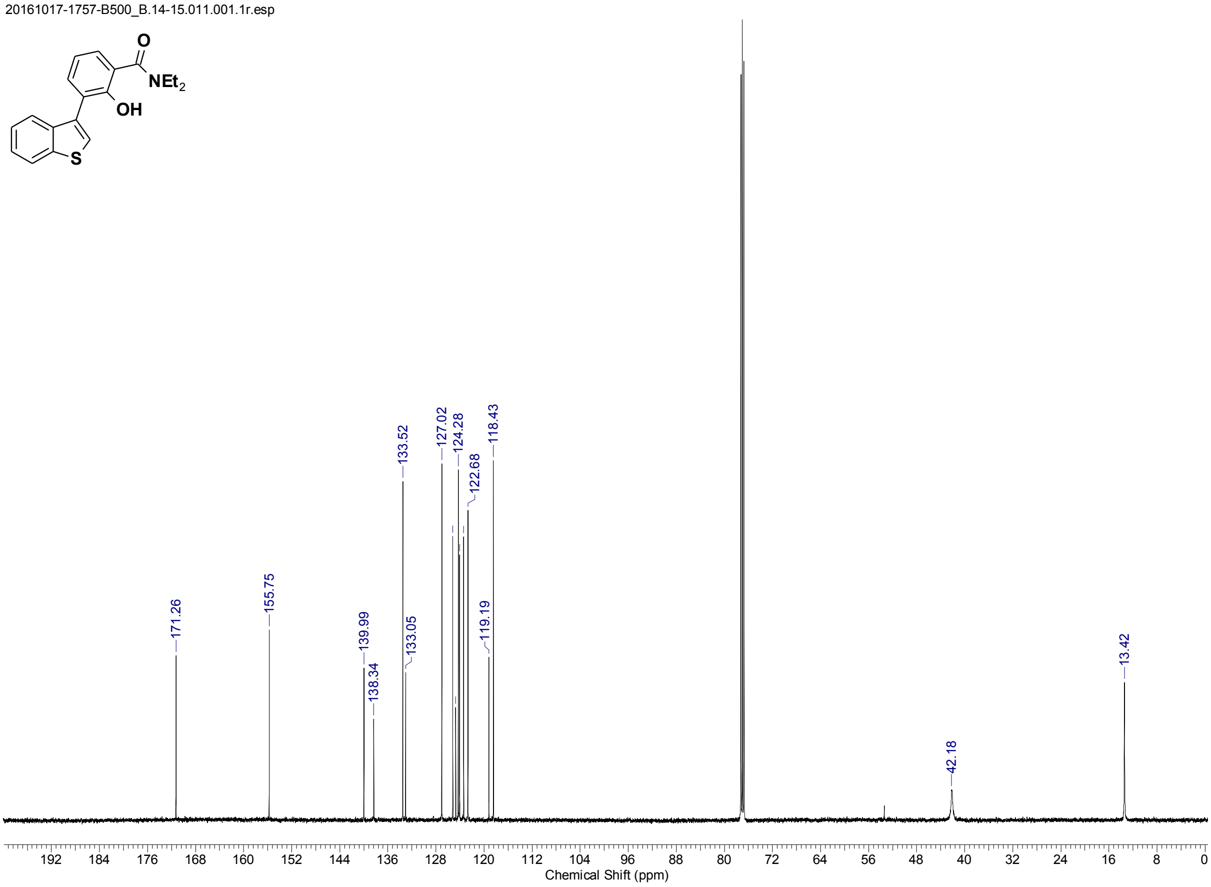

Supplementary Figure 22.

4l ^{1}H NMR (400 MHz, $CDCl_3$)

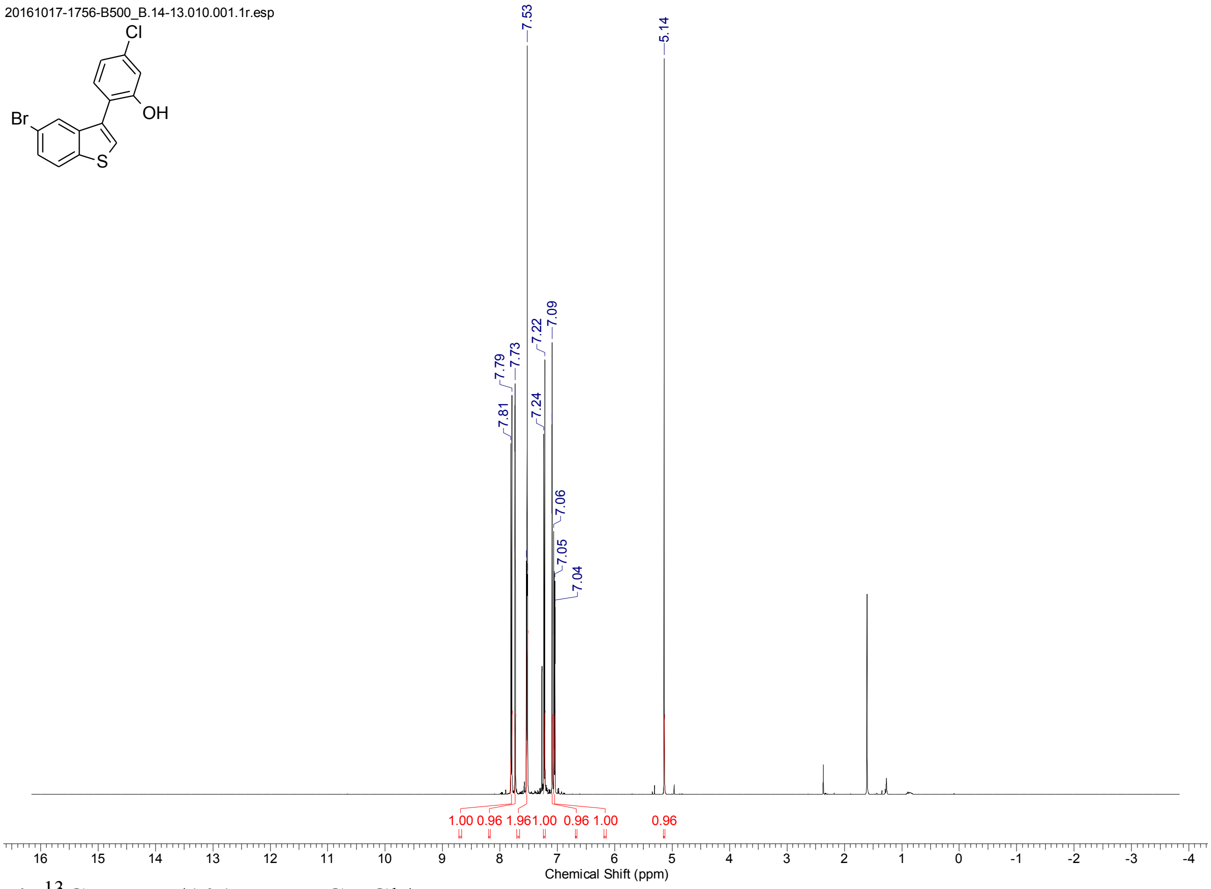

4l ^{13}C NMR (101 MHz, $CDCl_3$)

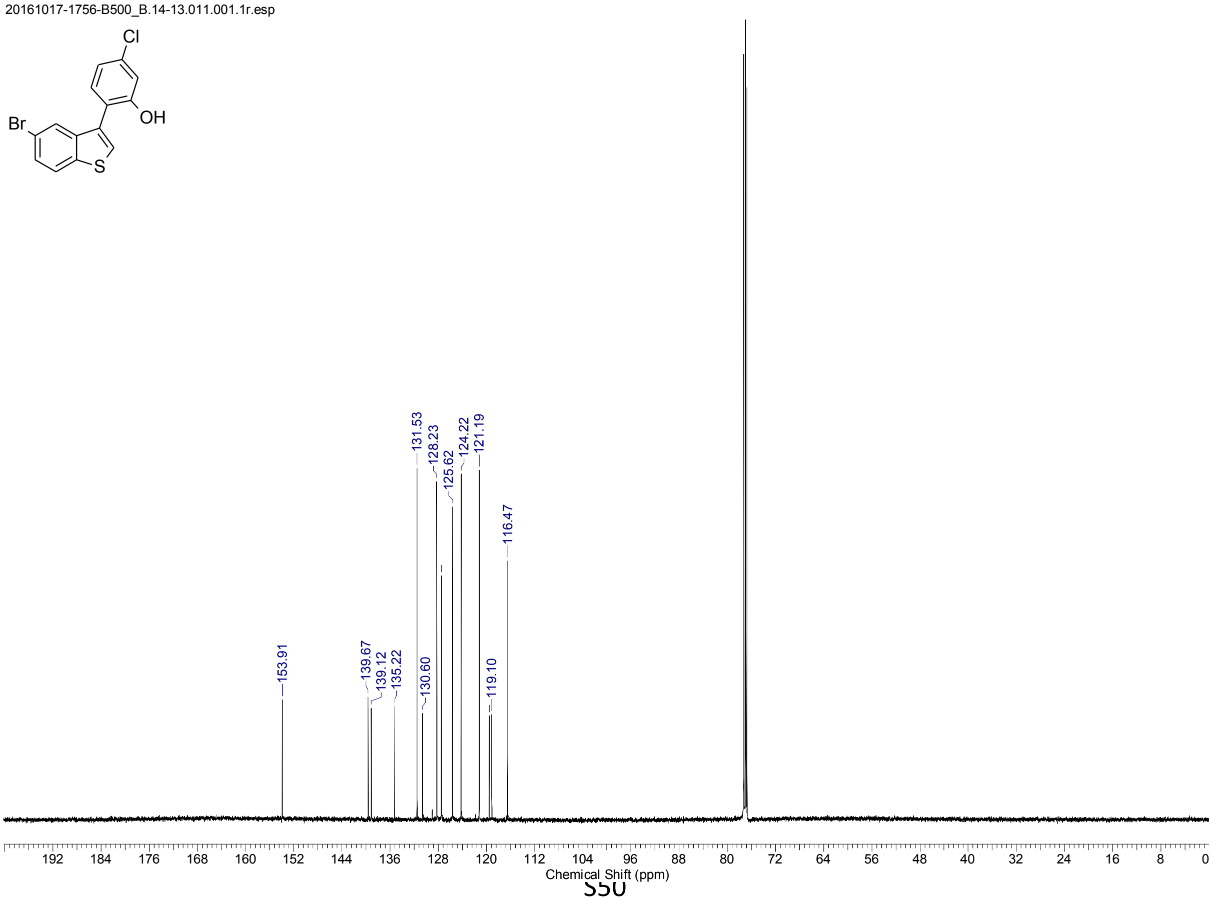

Supplementary Figure 23.

4m ^{1}H NMR (400 MHz, $CDCl_3$)

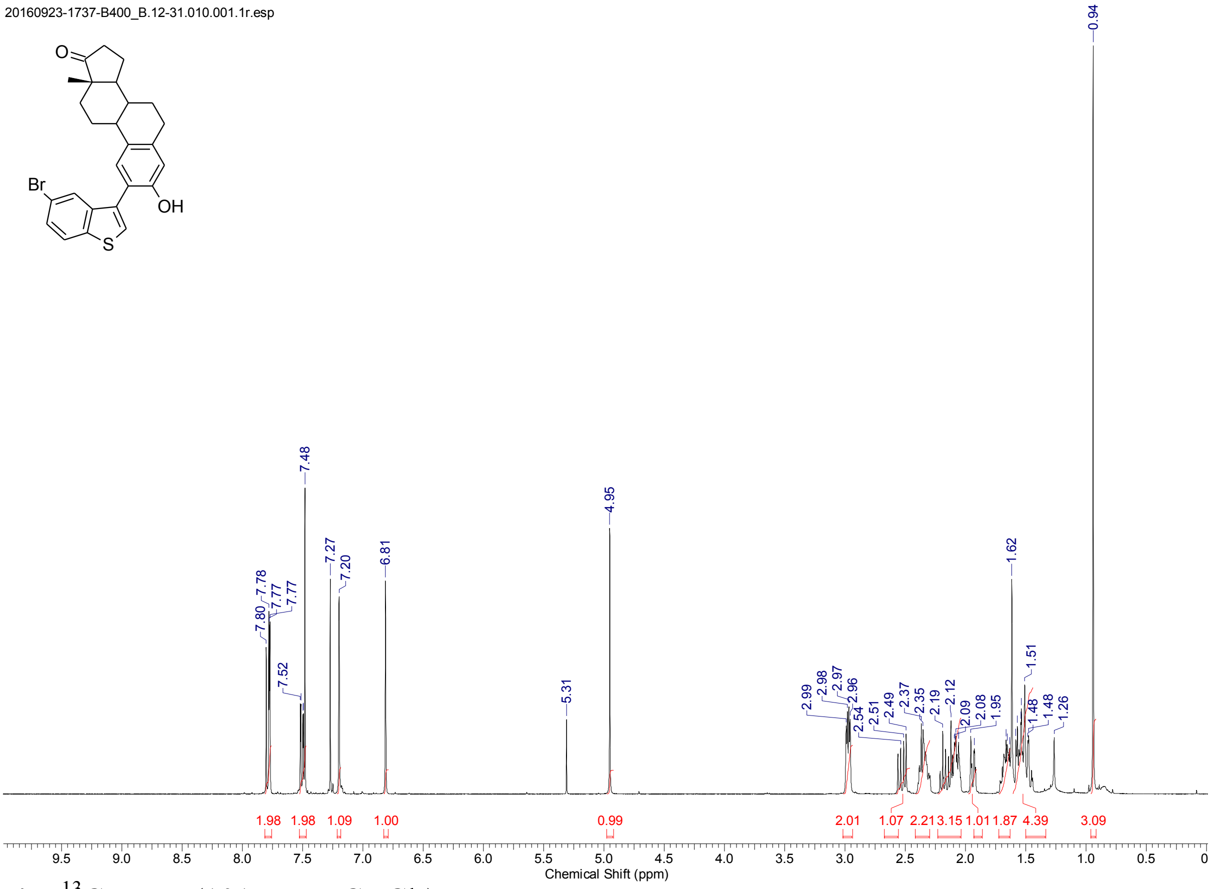

4m ^{13}C NMR (101 MHz, $CDCl_3$)

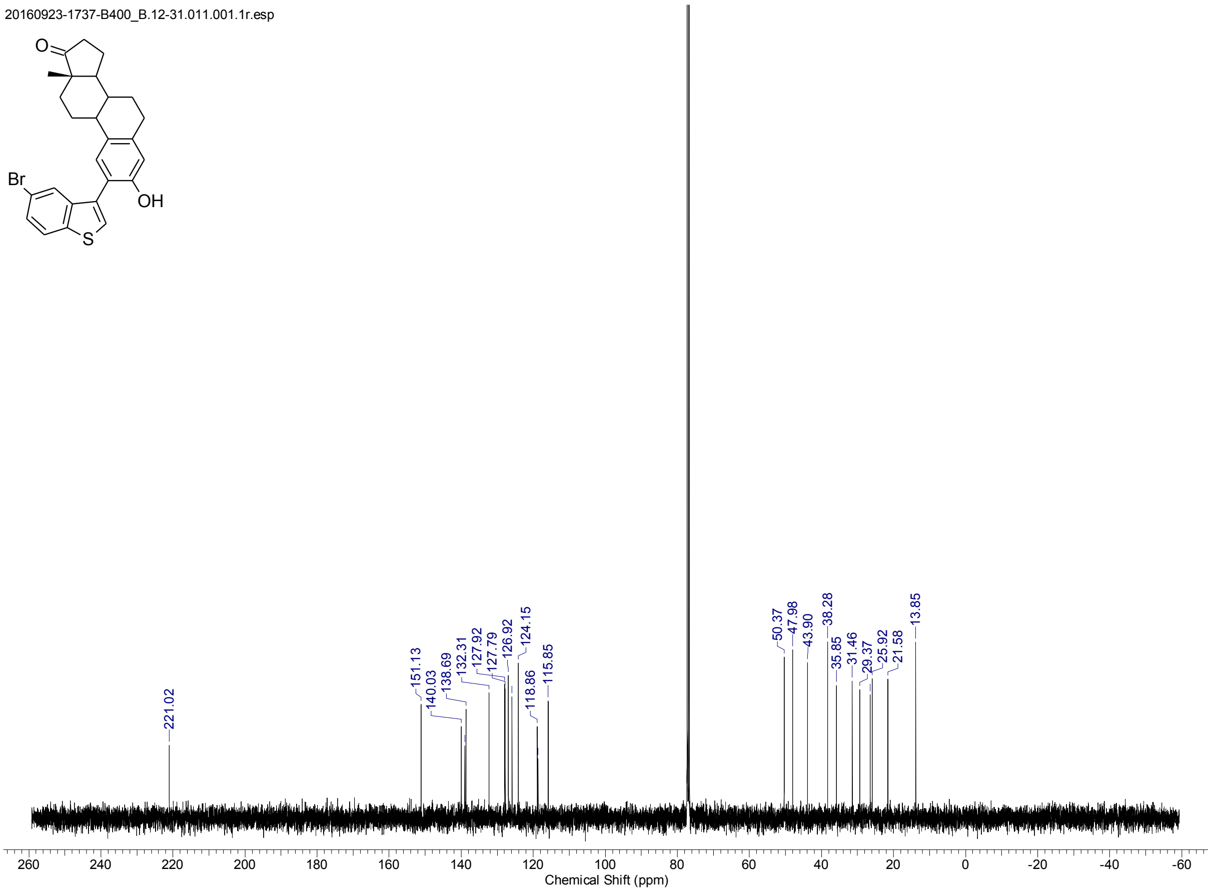

Supplementary Figure 24.

4n ^{1}H NMR (400 MHz, $CDCl_3$)

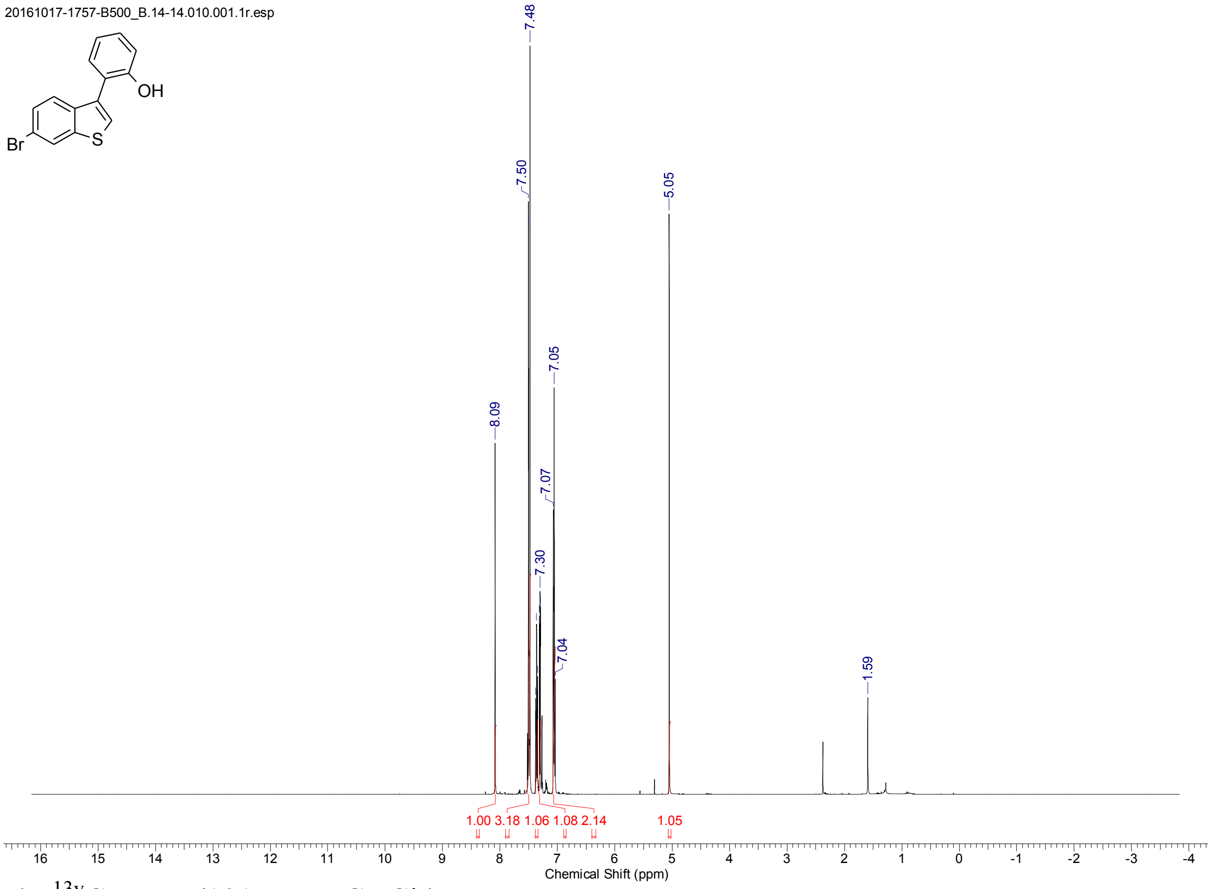

4n ^{13v}C NMR (101 MHz, $CDCl_3$)

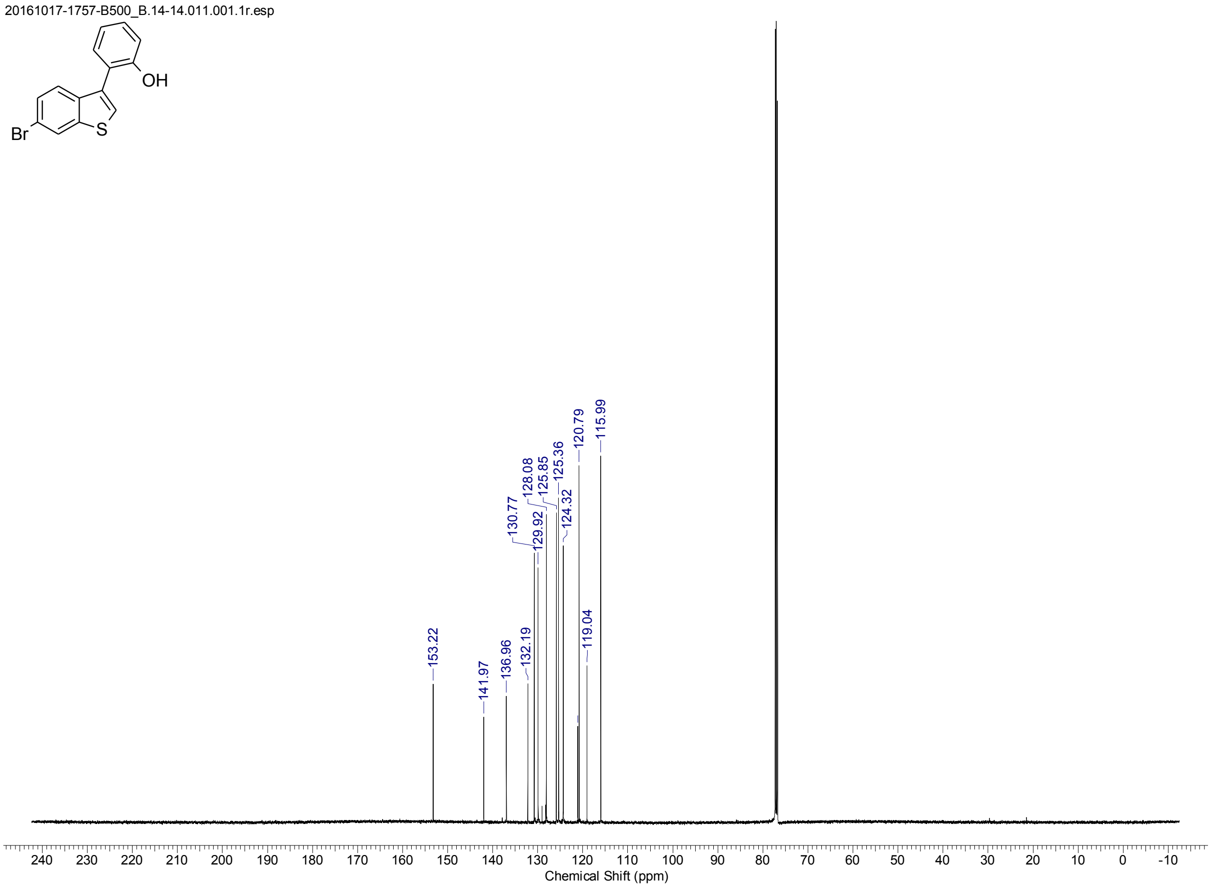

Supplementary Figure 25.

4o ^{1}H NMR (400 MHz, $CDCl_3$)

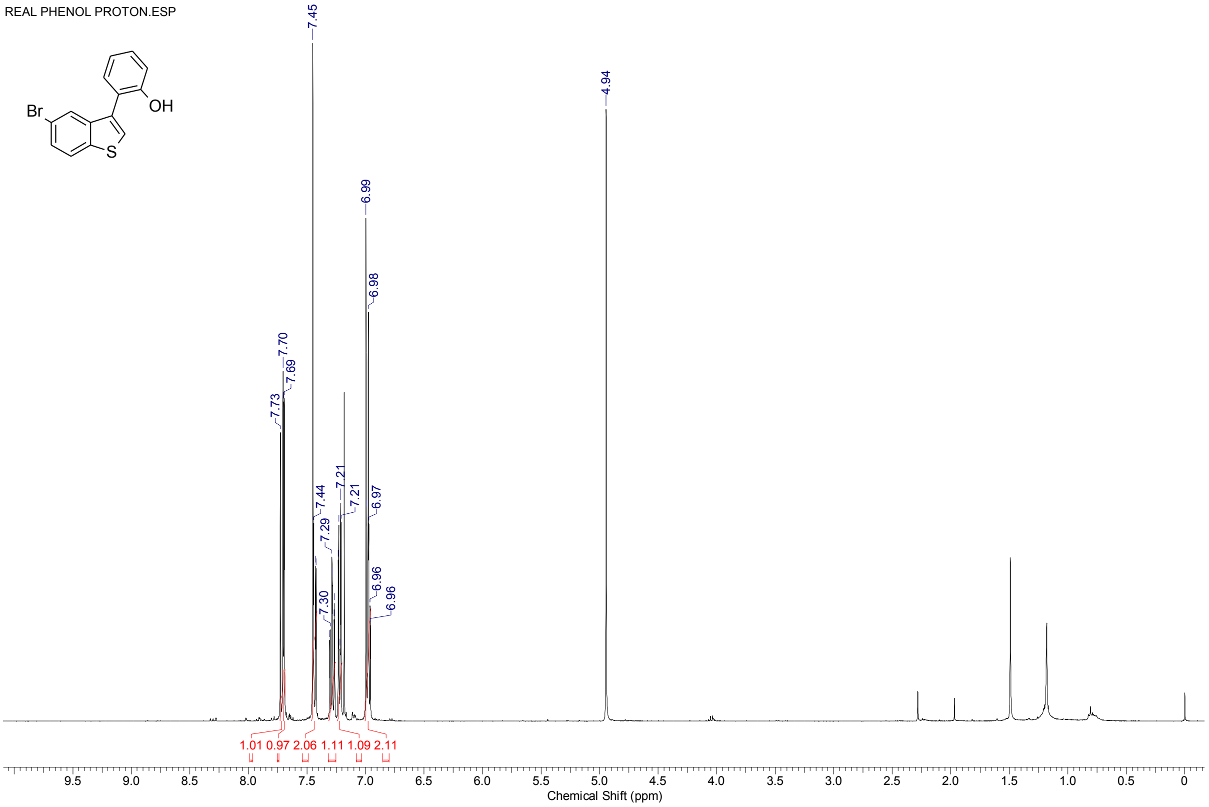

4o ^{13}C NMR (101 MHz, $CDCl_3$)

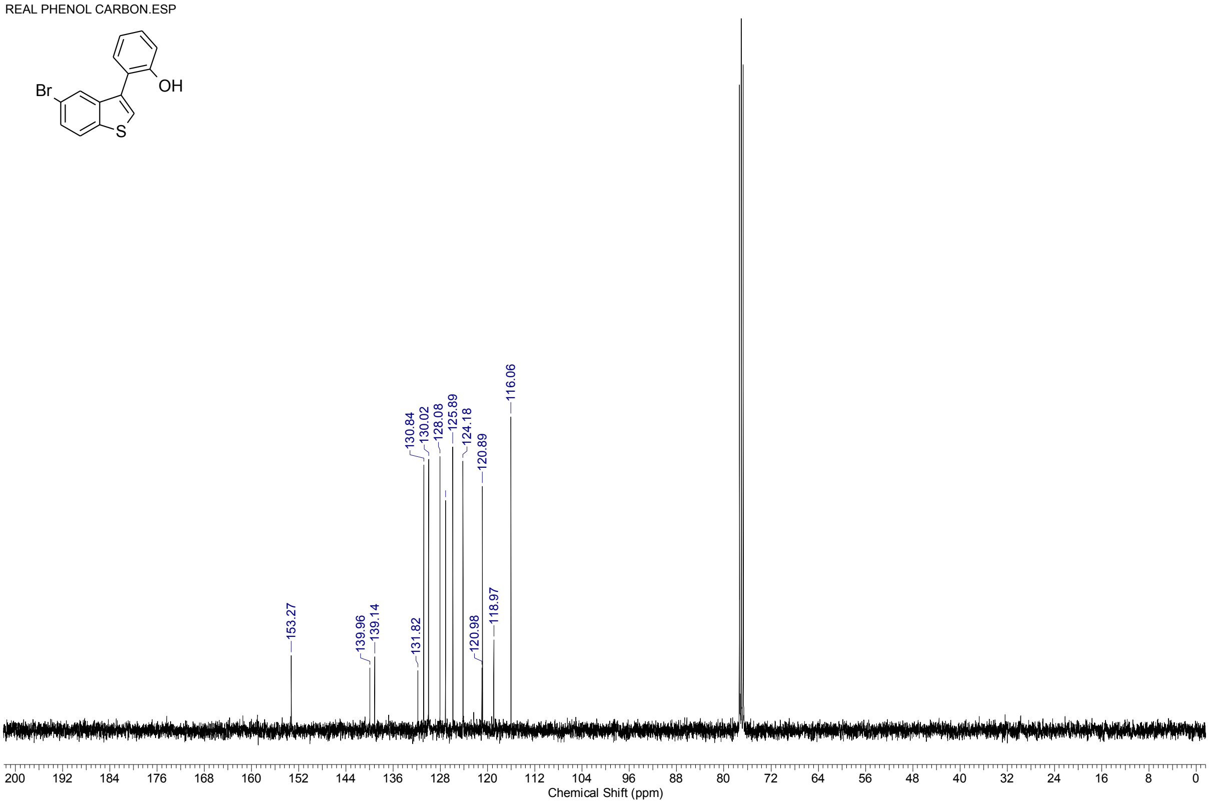

Supplementary Figure 26.

4p ^{1}H NMR (400 MHz, $CDCl_3$)

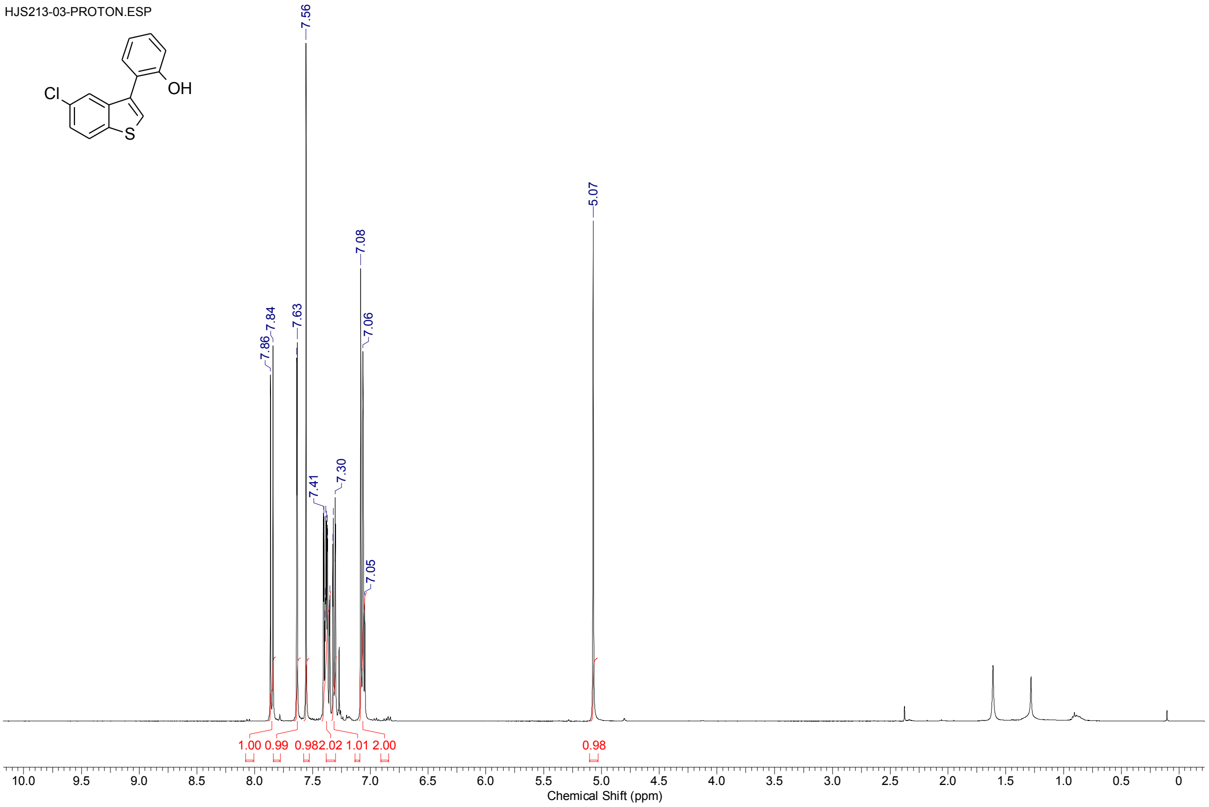

4p ^{13}C NMR (101 MHz, $CDCl_3$)

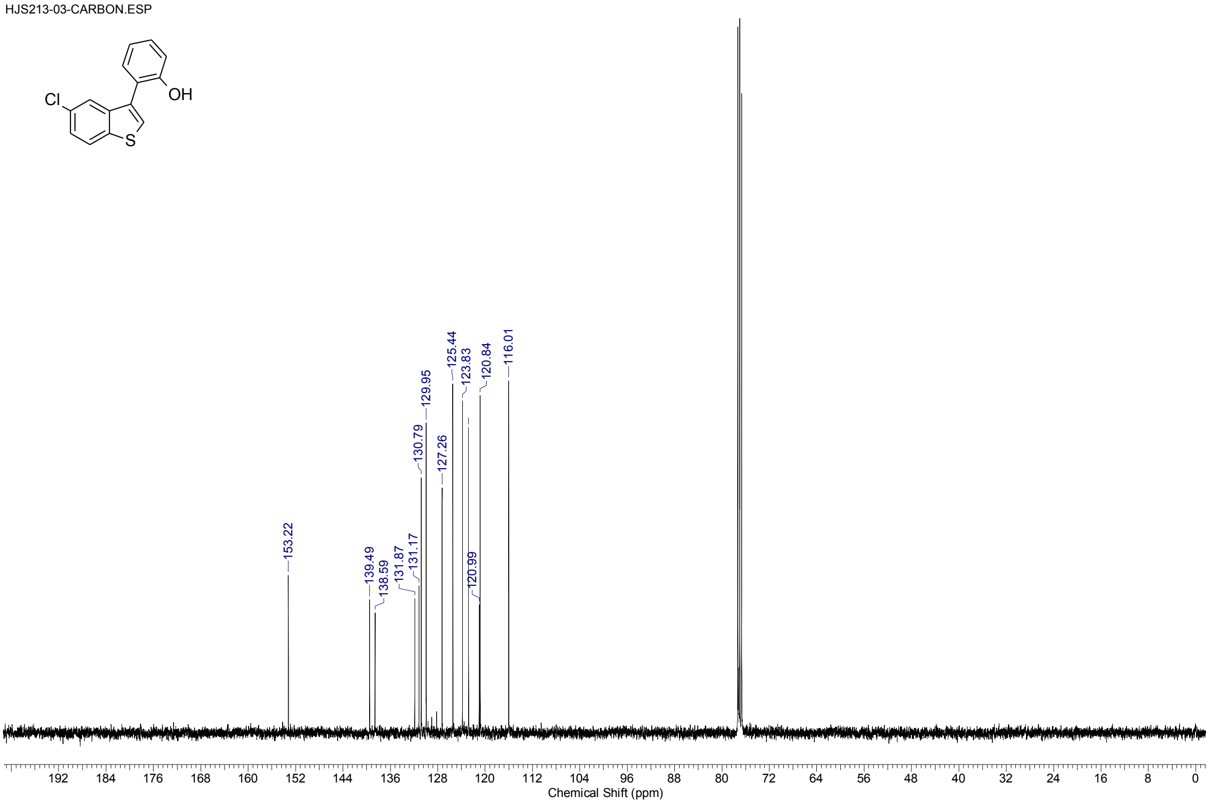

Supplementary Figure 27.

4q ^{1}H NMR (400 MHz, $CDCl_3$)

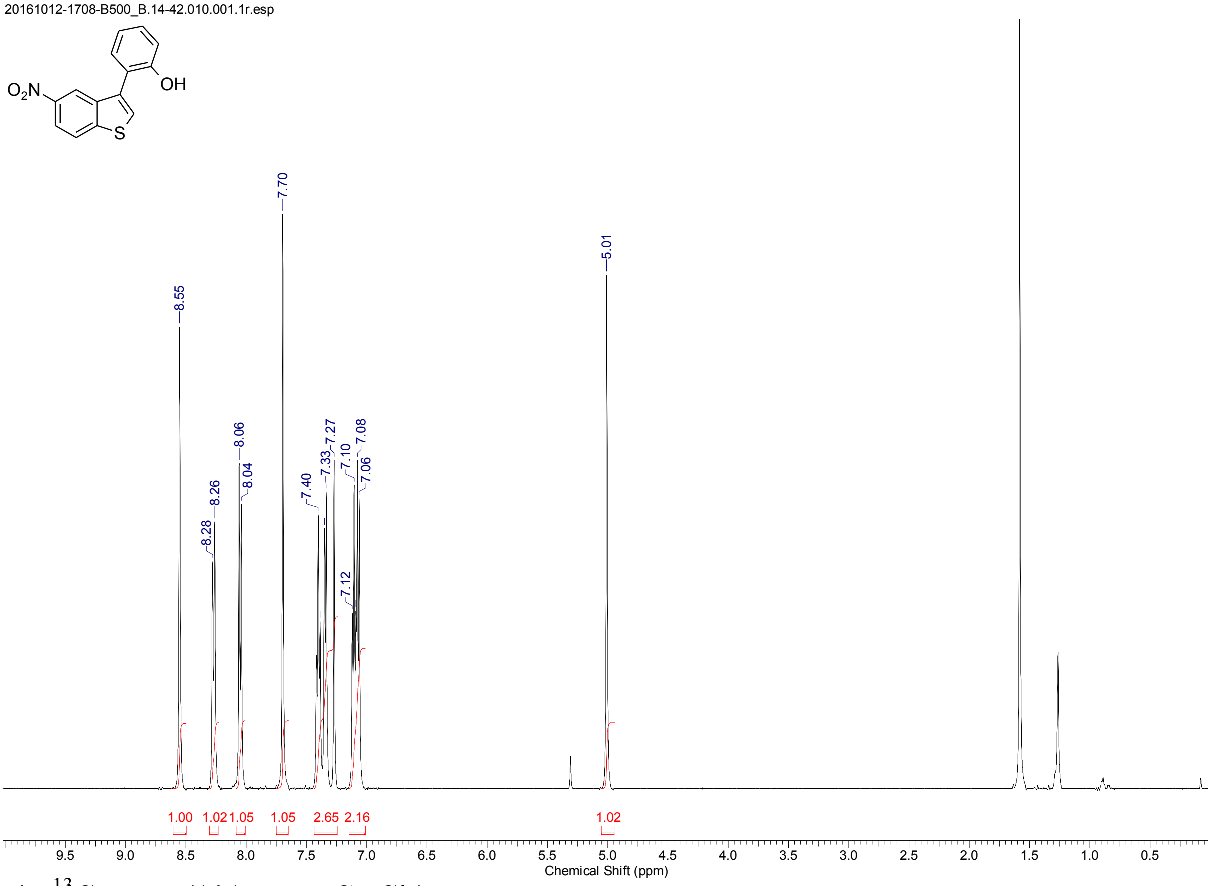

4q ^{13}C NMR (101 MHz, $CDCl_3$)

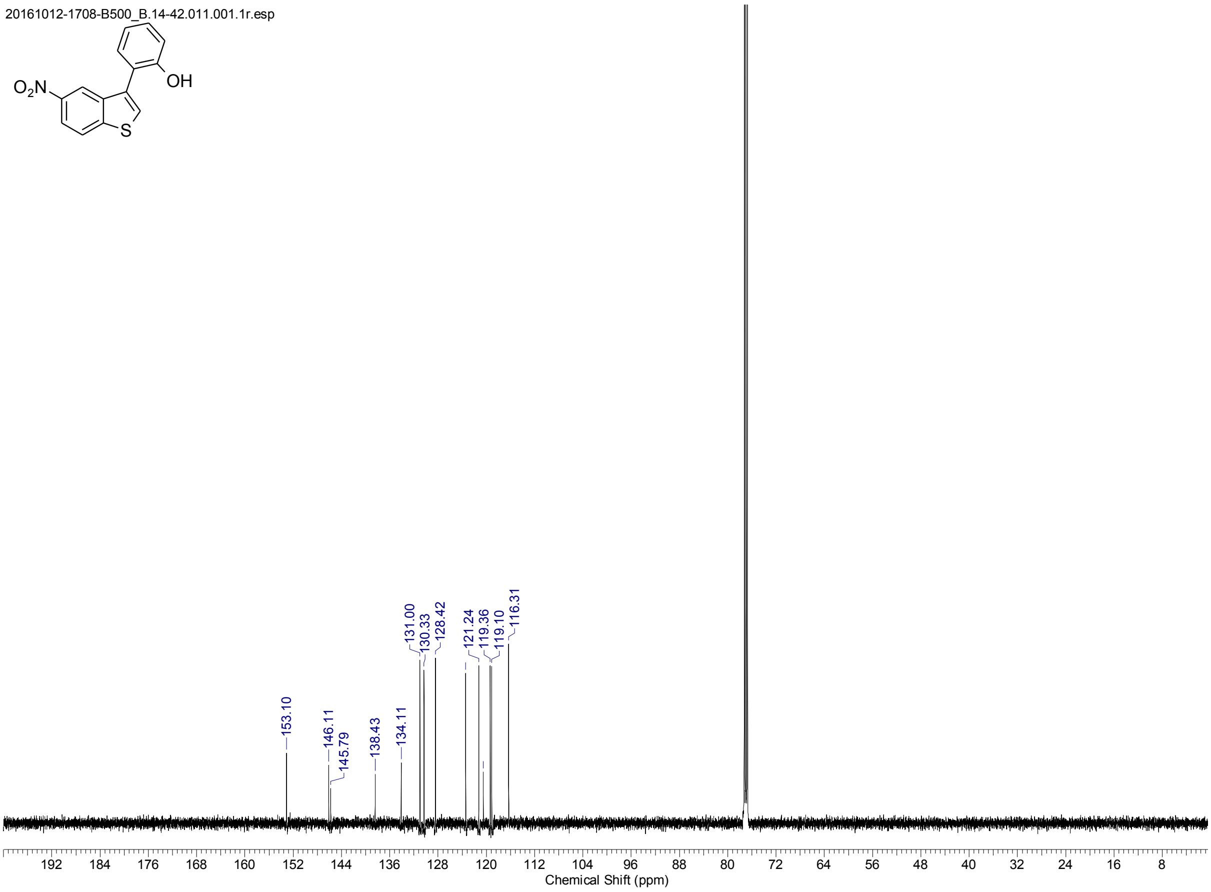

Supplementary Figure 28.

4r ^{1}H NMR (400 MHz, $CDCl_3$)

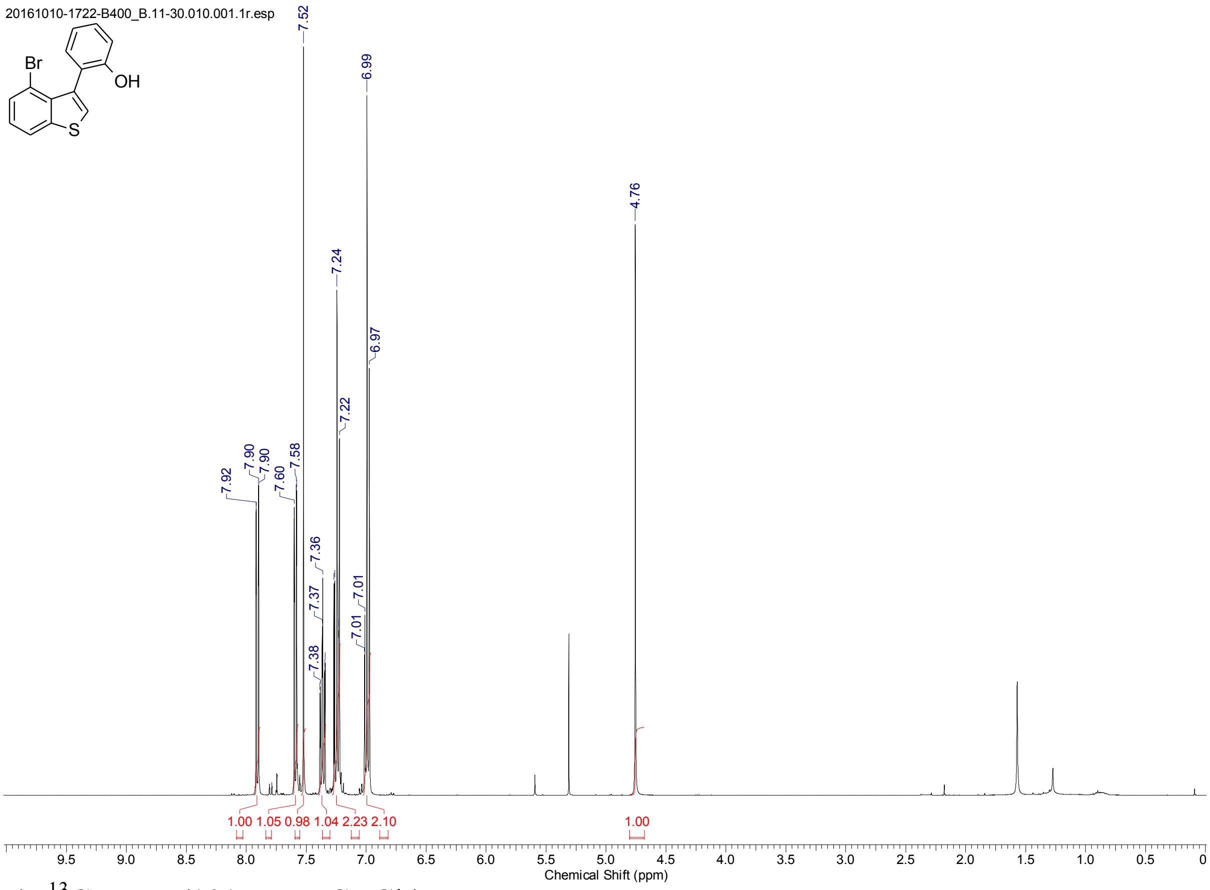

4r ^{13}C NMR (101 MHz, $CDCl_3$)

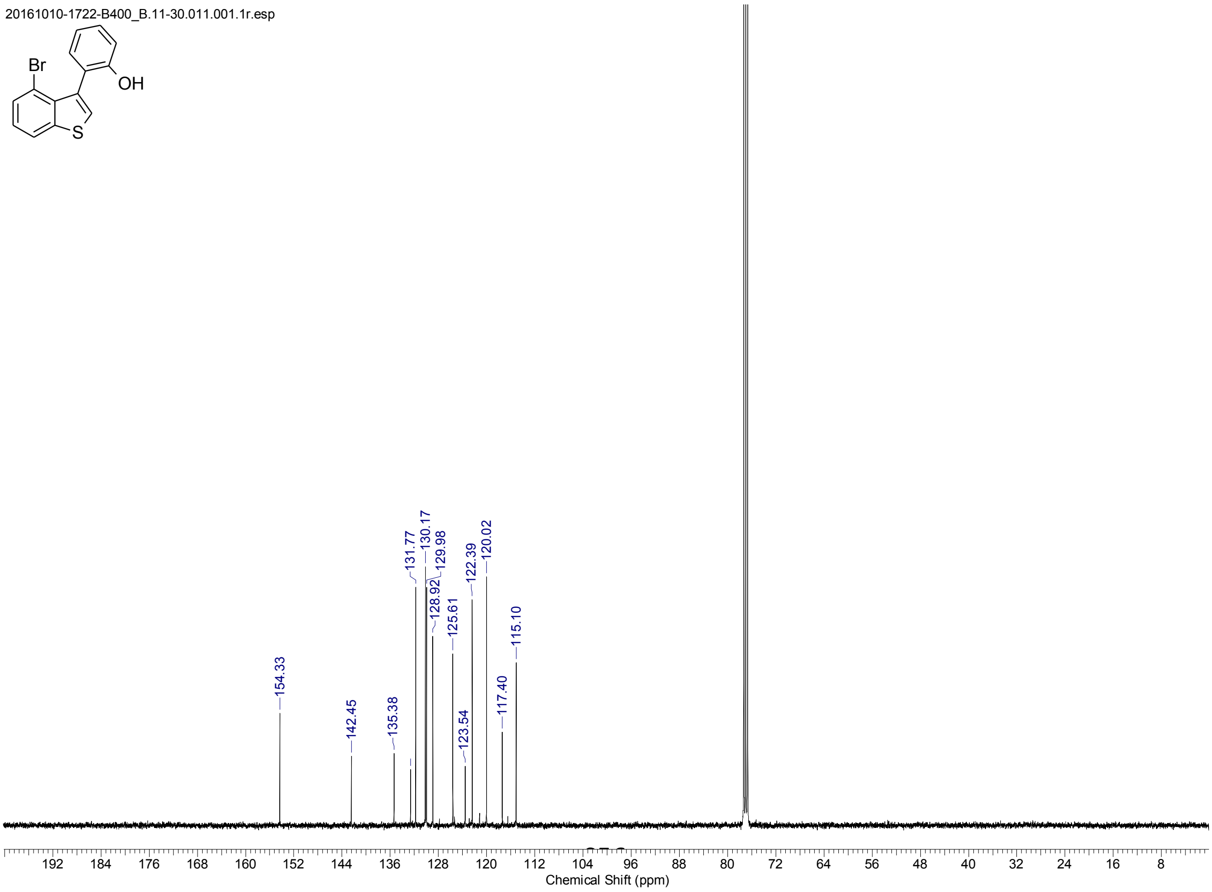

Supplementary Figure 29.

4s ¹H NMR (400 MHz, $CDCl_3$)

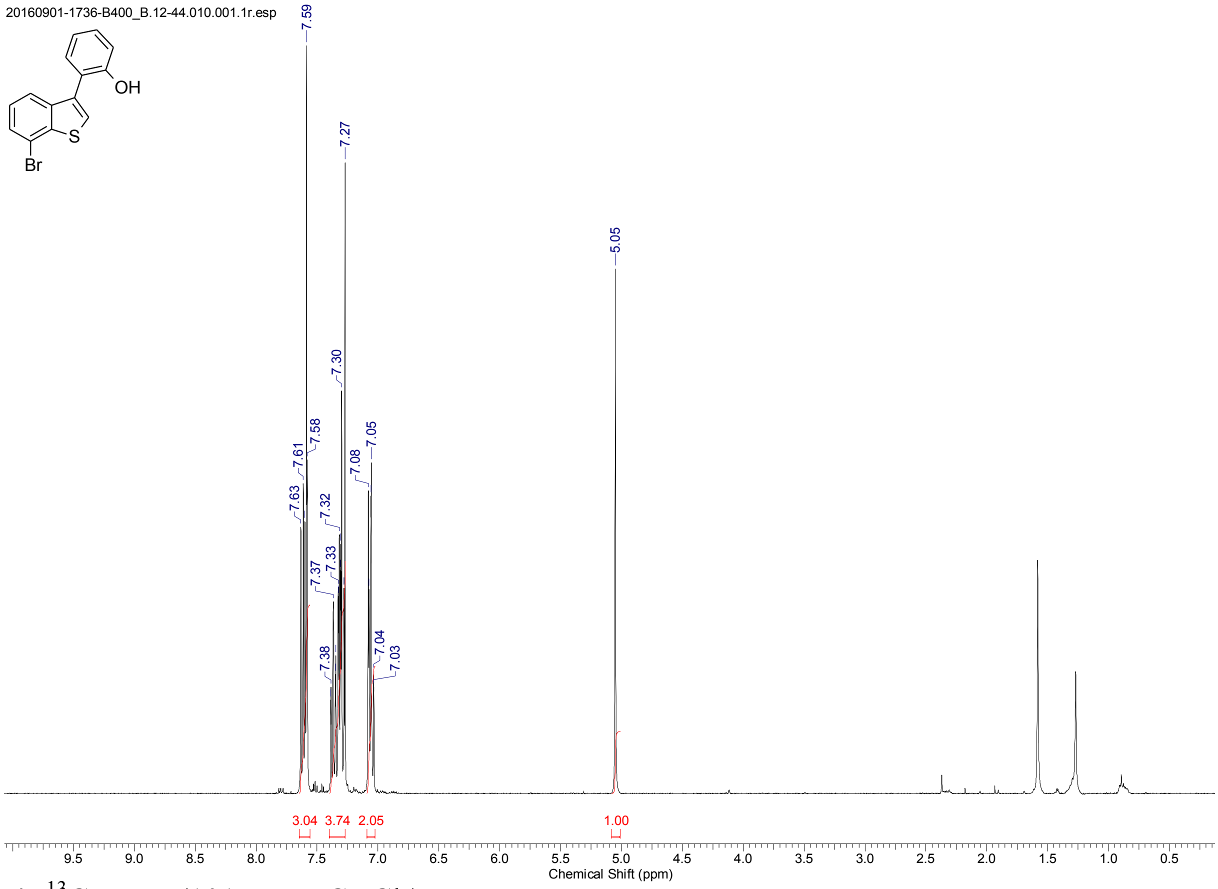

4s ¹³C NMR (101 MHz, $CDCl_3$)

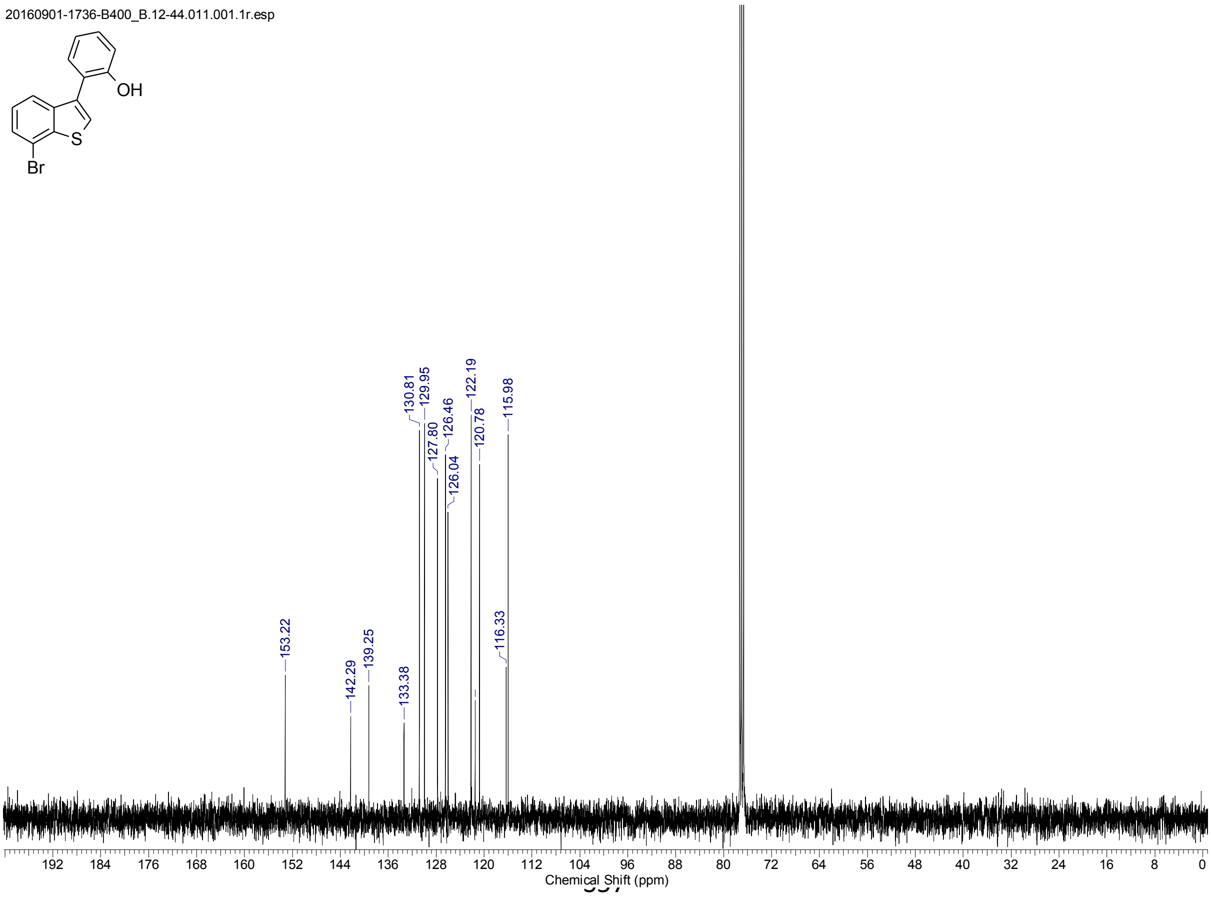

Supplementary Figure 30.

4t ^{1}H NMR (400 MHz, $CDCl_3$)

CH099.011.001.1r.esp

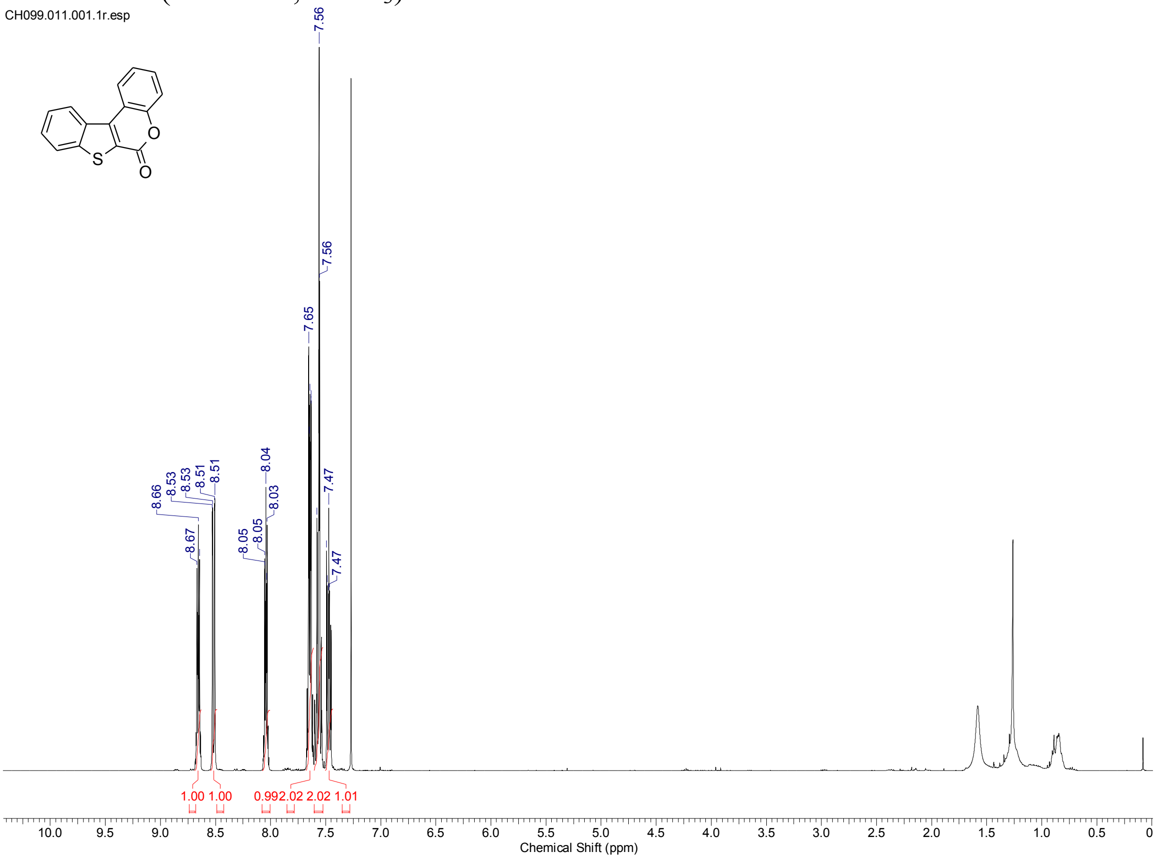

4t ^{13}C NMR (101 MHz, $CDCl_3$)

CH099.012.001.1r.esp

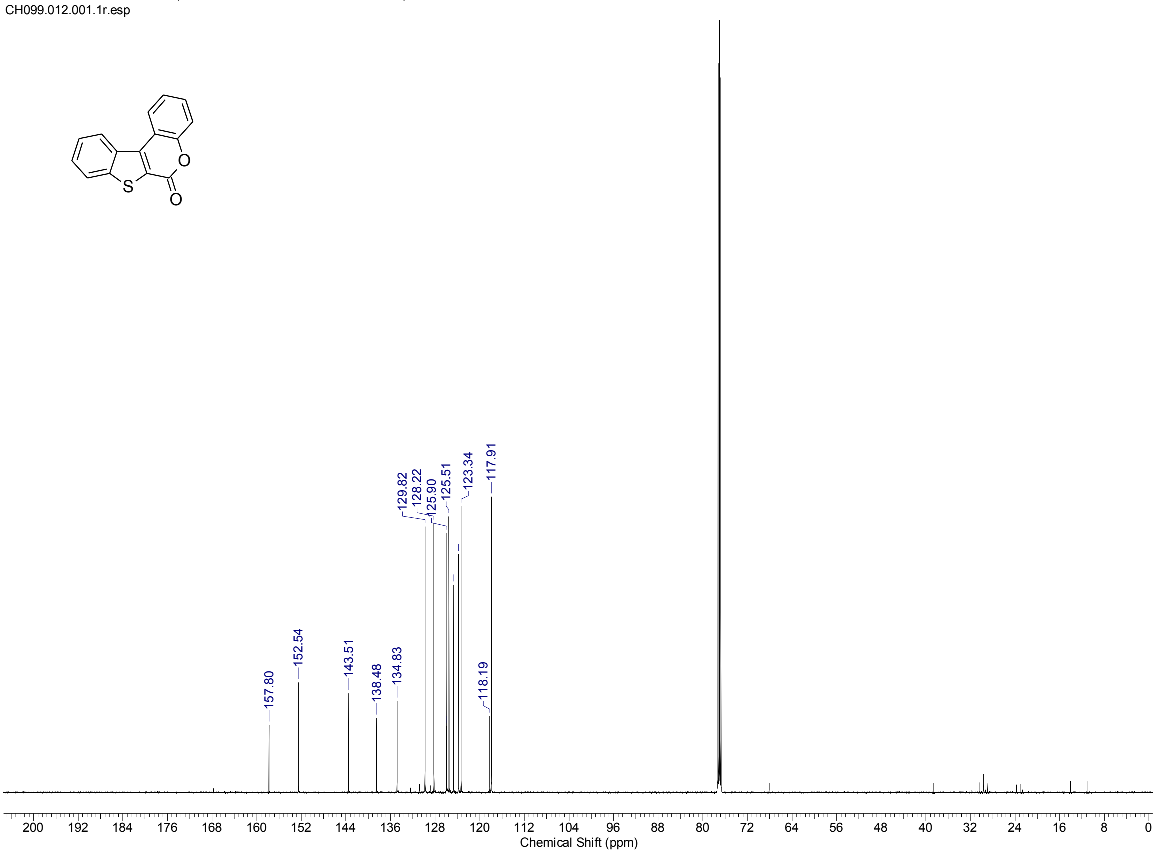

Supplementary Figure 31.

4u ^{1}H NMR (400 MHz, $CDCl_3$)

CH097.011.001.1r.esp

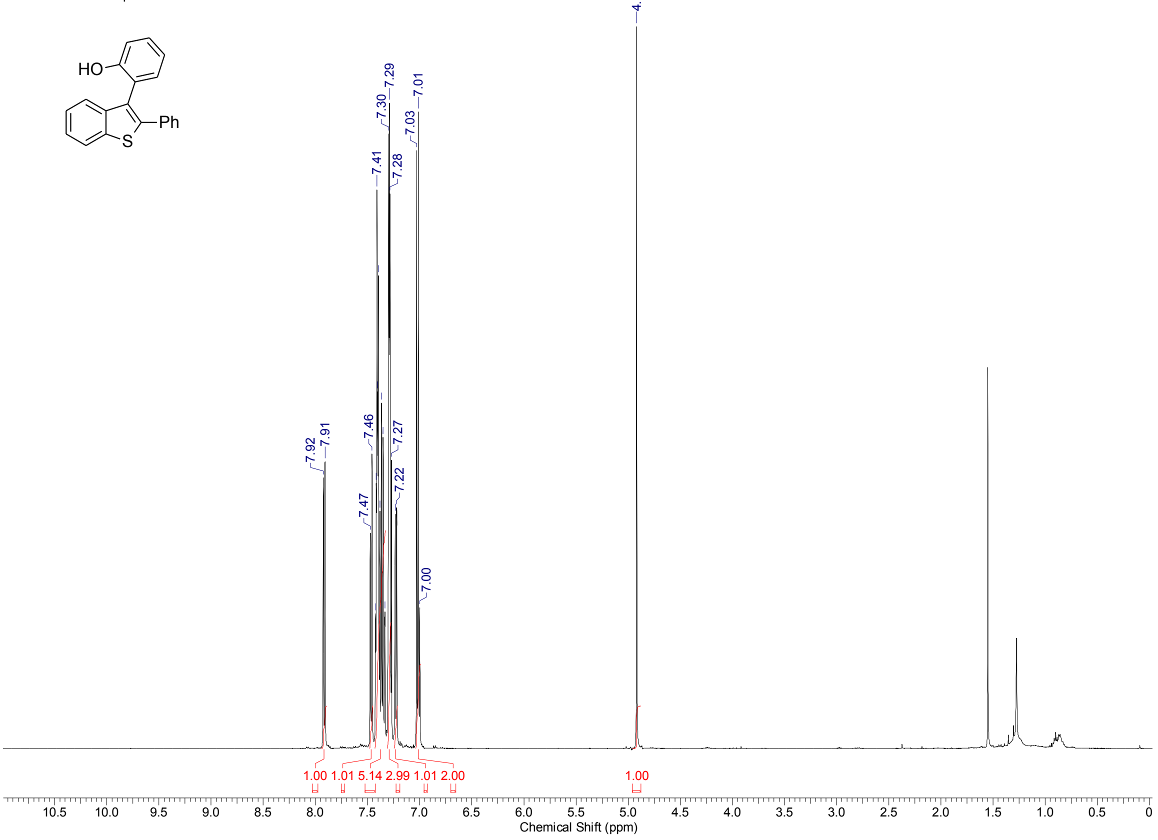

4u ^{13}C NMR (101 MHz, $CDCl_3$)

CH097.012.001.1r.esp

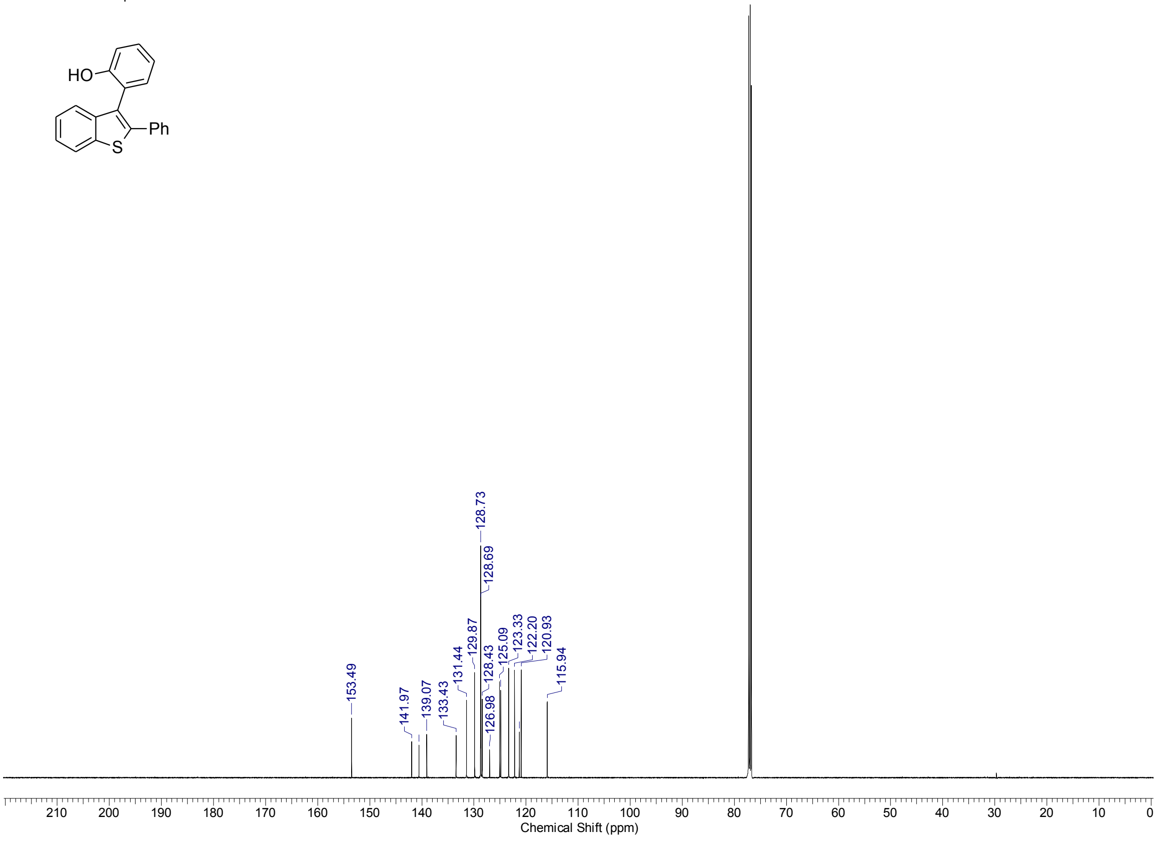

Supplementary Figure 32.

6a ^{1}H NMR (400 MHz, $CDCl_3$)

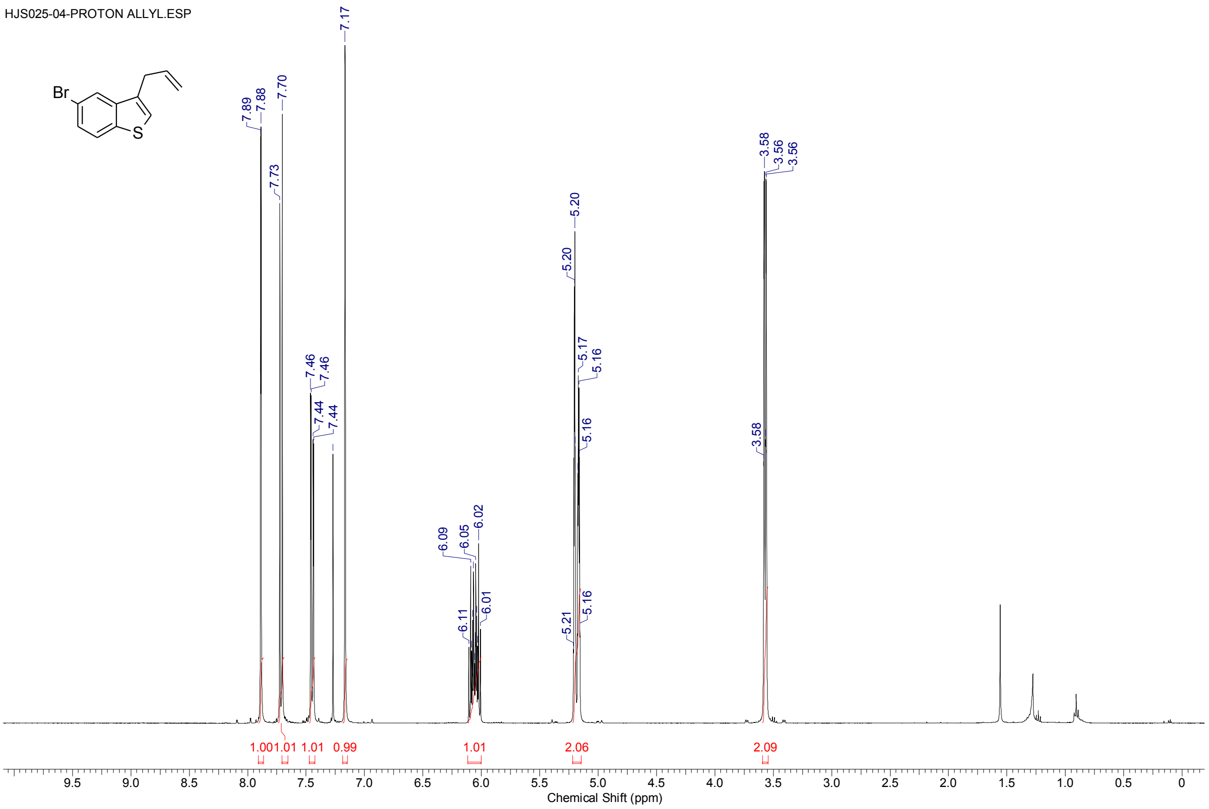

6a ^{13}C NMR (101 MHz, $CDCl_3$)

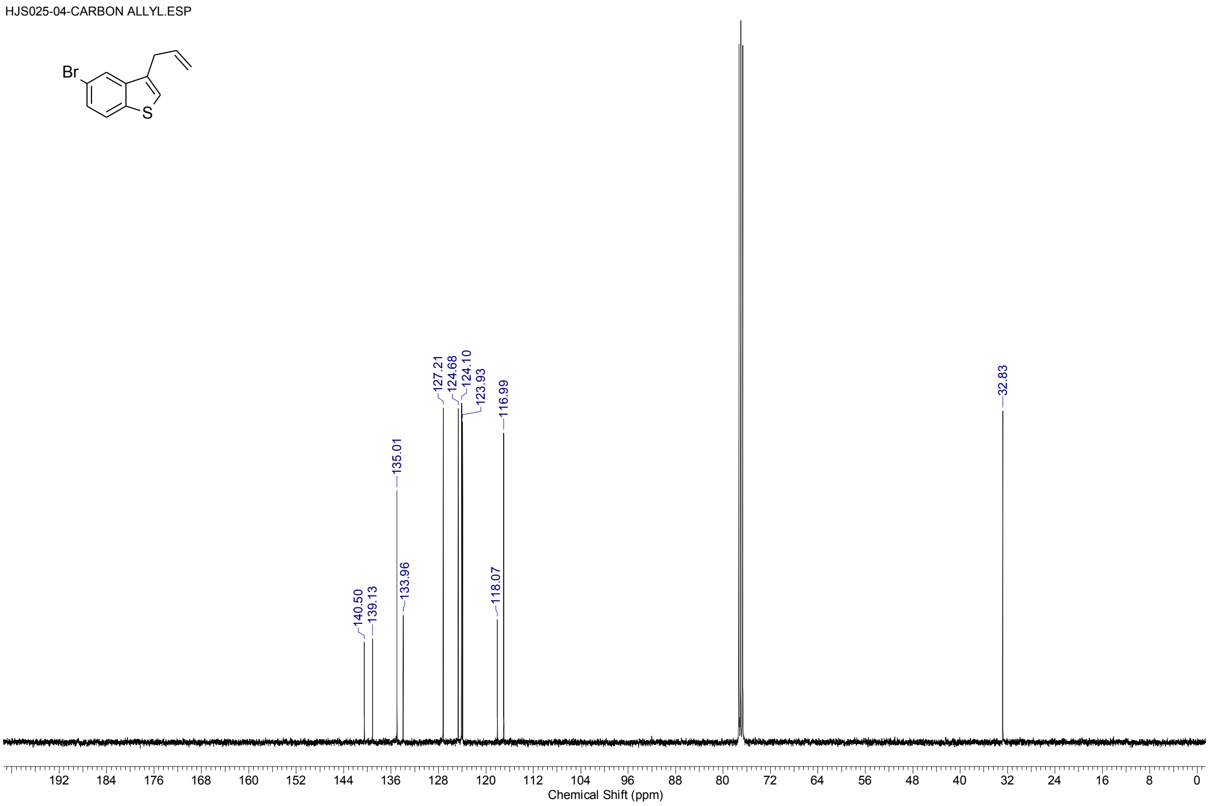

Supplementary Figure 33.

6b ^{1}H NMR (400 MHz, $CDCl_3$)

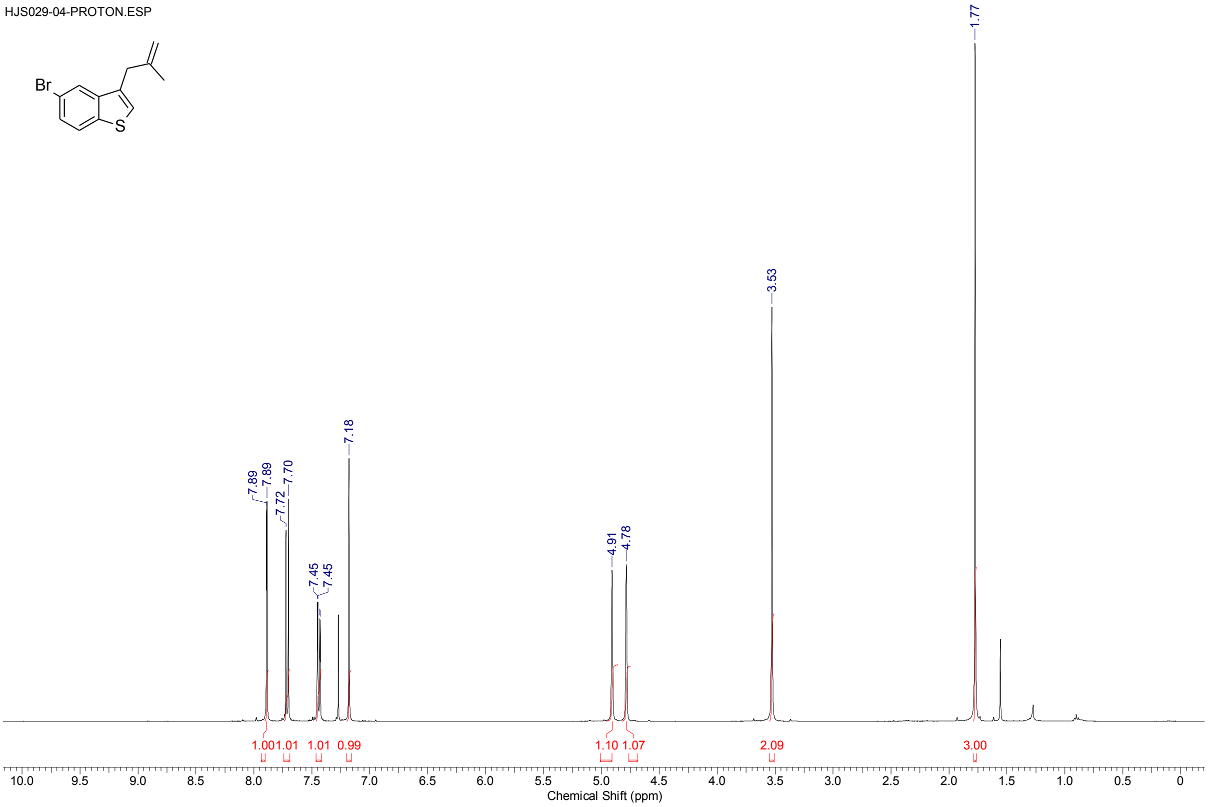

6b ^{13}C NMR (101 MHz, $CDCl_3$

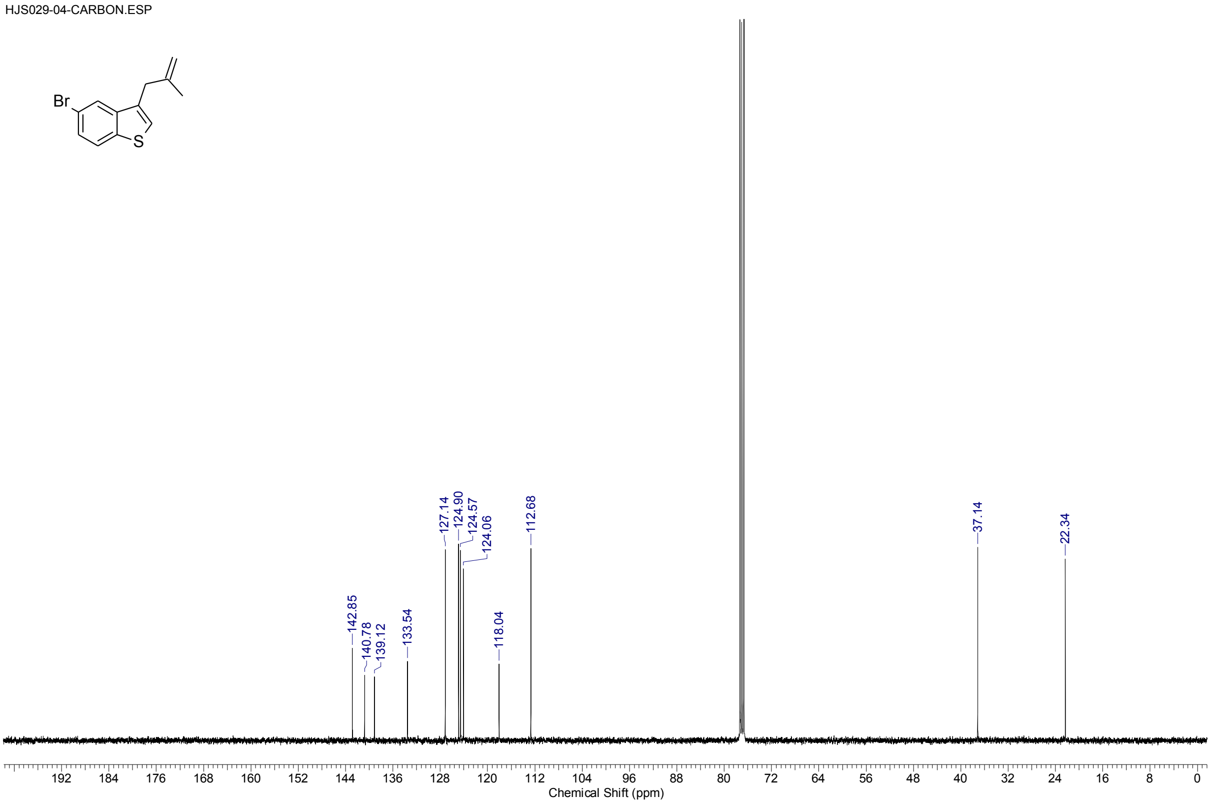

Supplementary Figure 34.

6c ^{1}H NMR (400 MHz, $CDCl_3$)

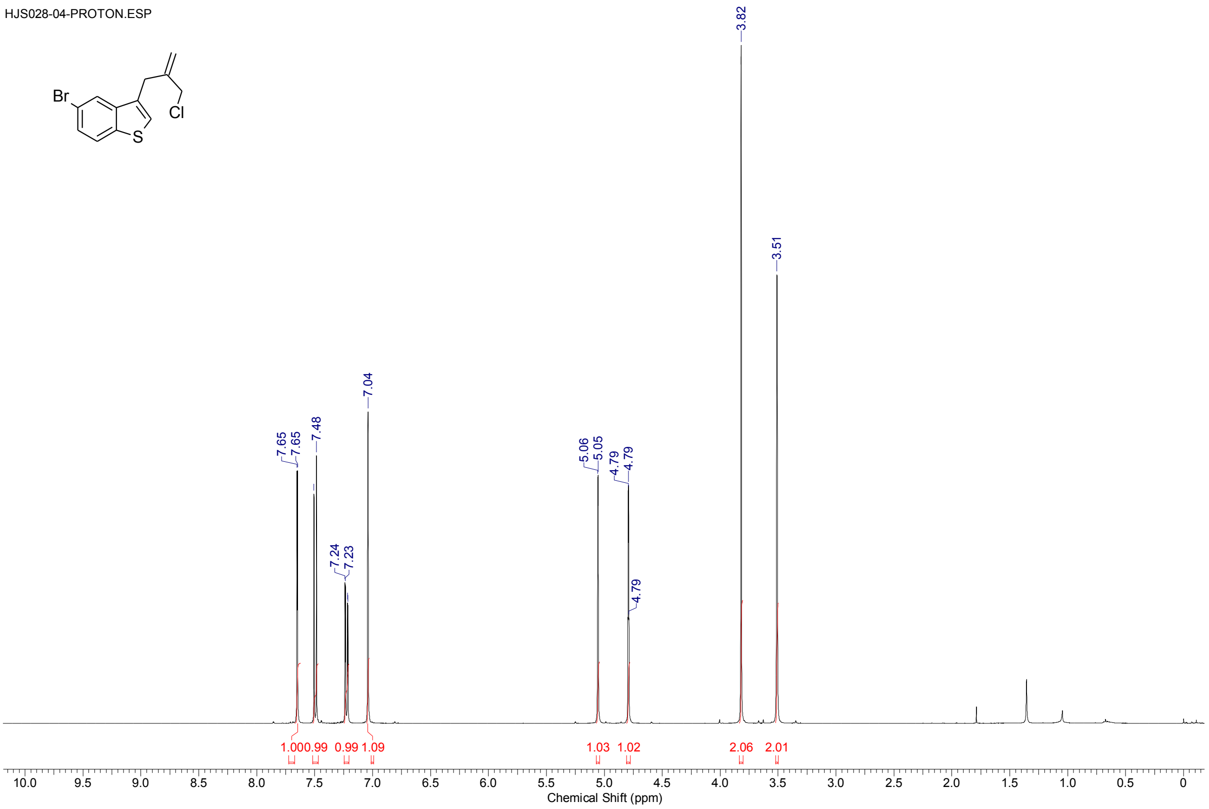

6c ^{13}C NMR (101 MHz, $CDCl_3$)

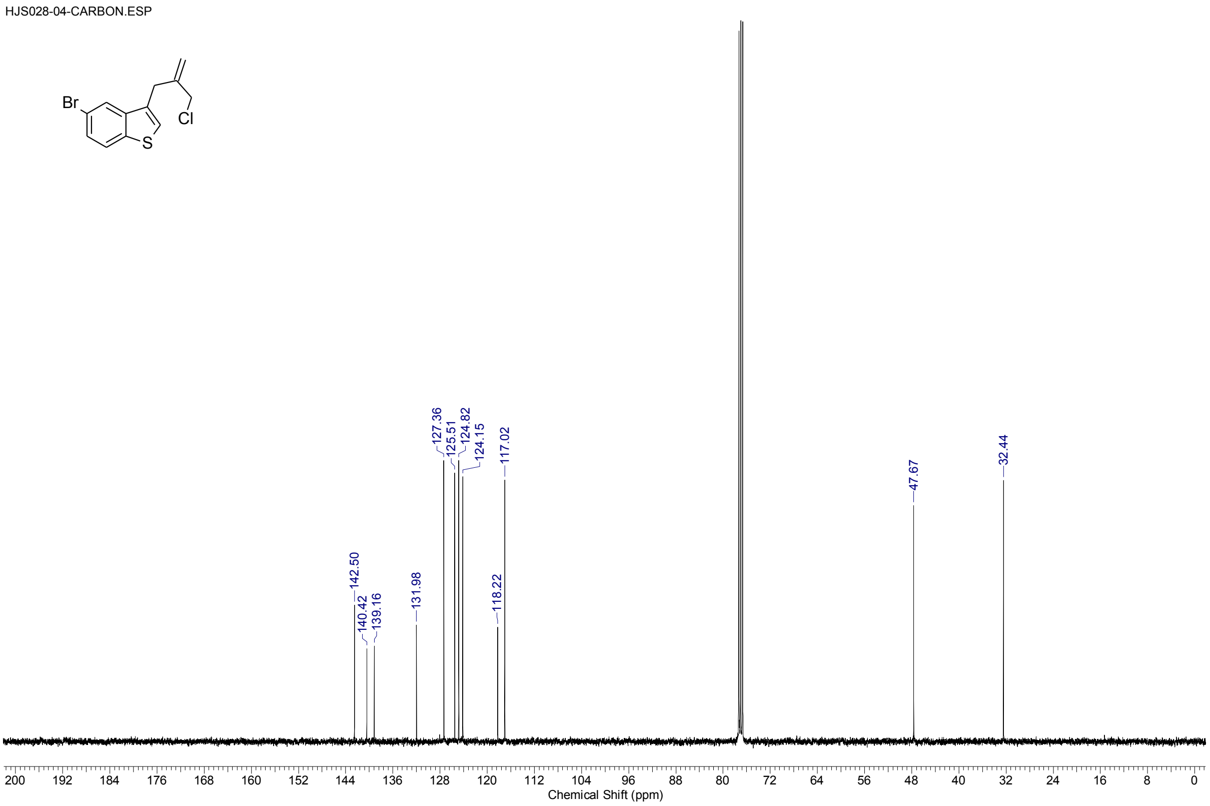

Supplementary Figure 35.

6d ^{1}H NMR (400 MHz, $CDCl_3$)

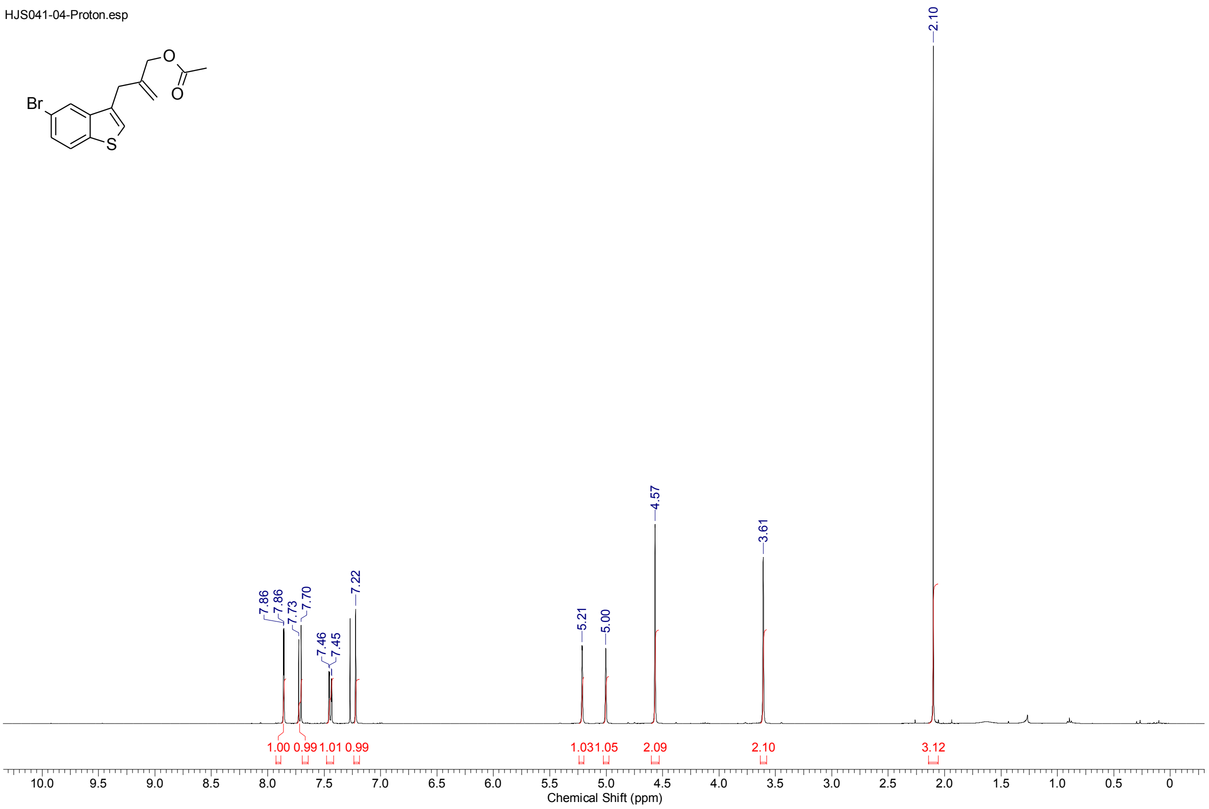

6d ^{13}C NMR (101 MHz, $CDCl_3$)

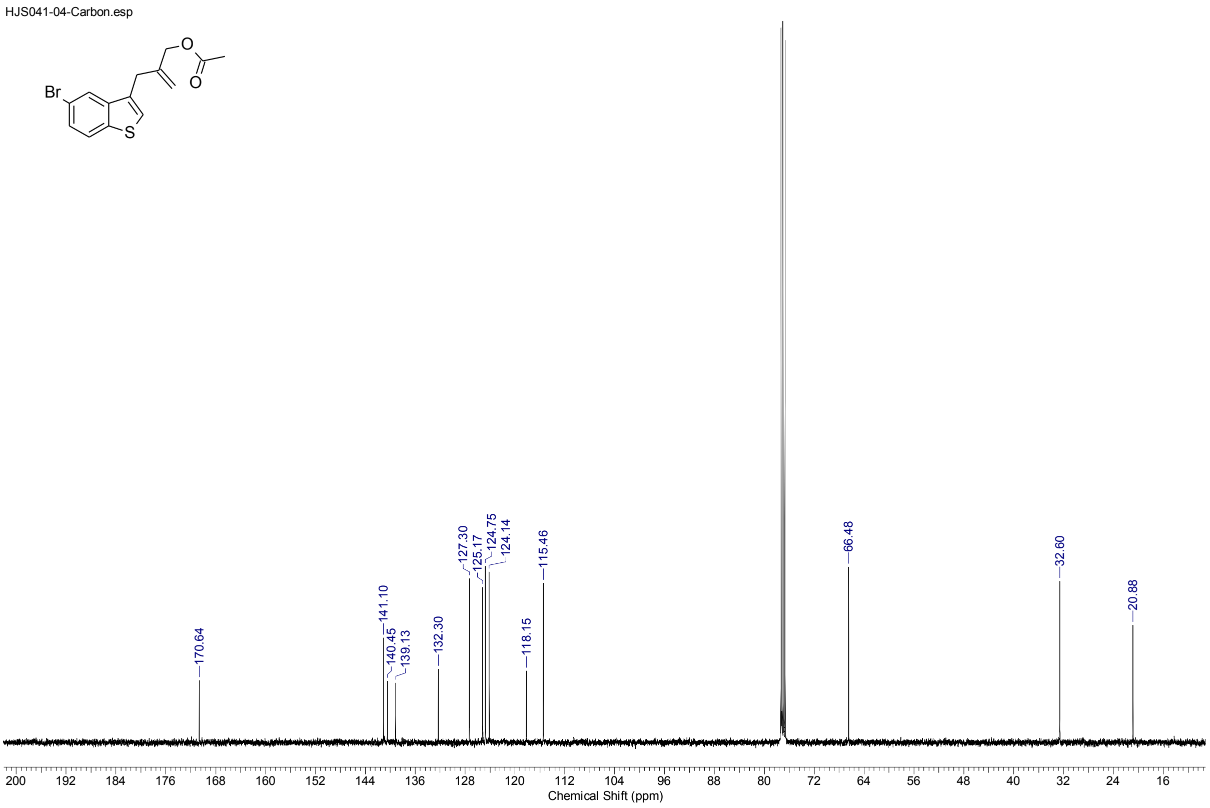

Supplementary Figure 36.

6e ^{1}H NMR (400 MHz, $CDCl_3$)

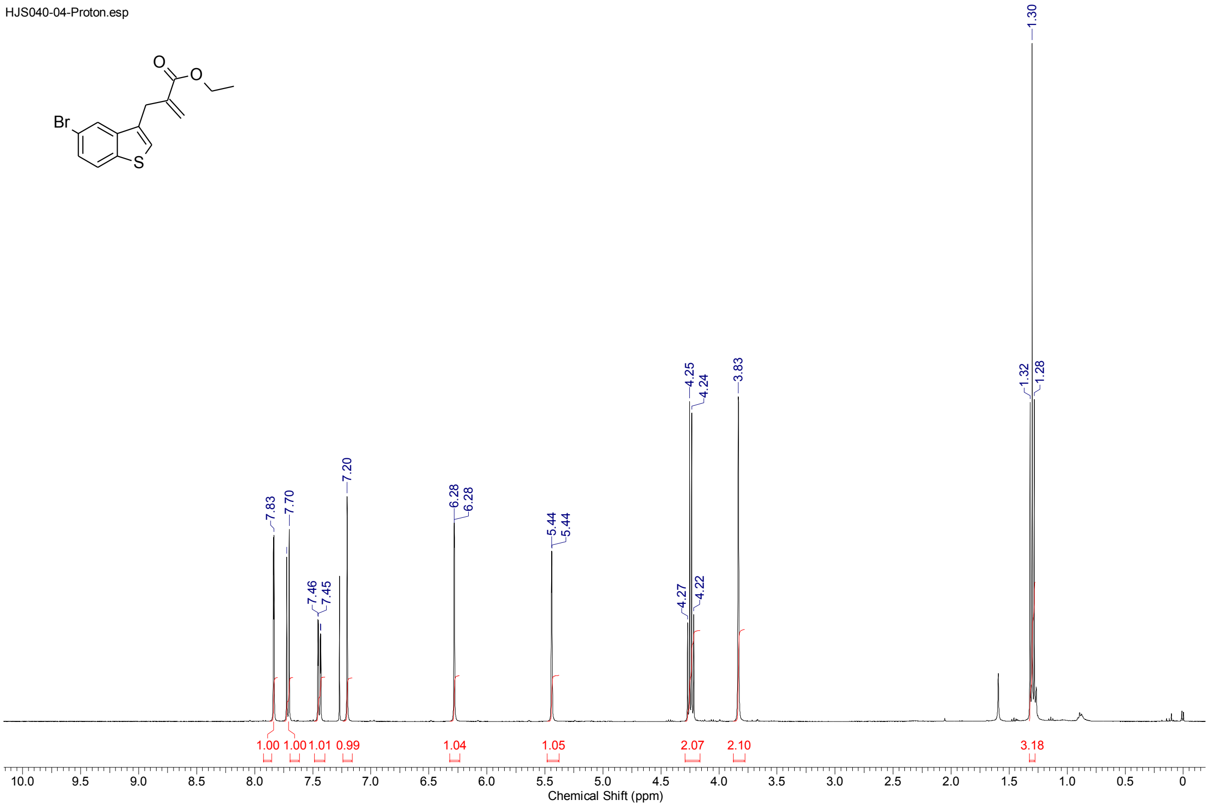

6e ^{13}C NMR (101 MHz, $CDCl_3$)

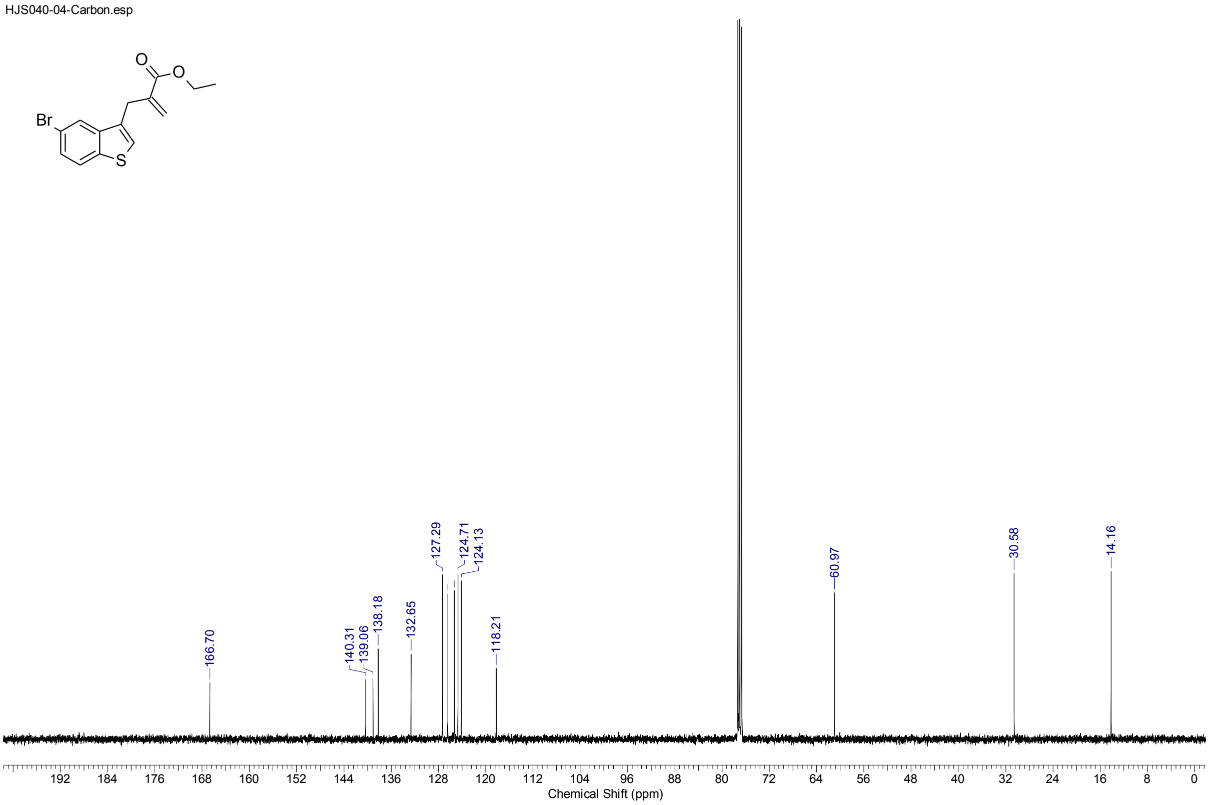

Supplementary Figure 37.

6f ^{1}H NMR (400 MHz, $CDCl_3$)

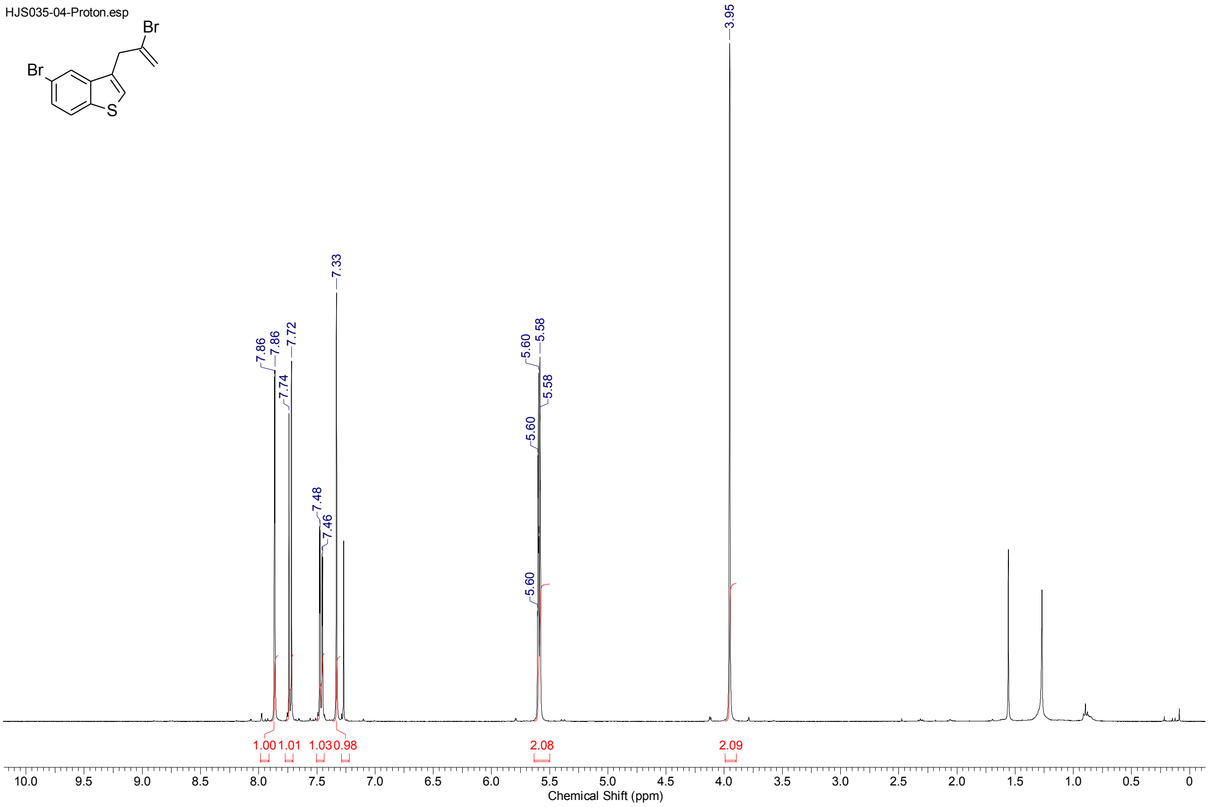

6f ^{13}C NMR (101 MHz, $CDCl_3$)

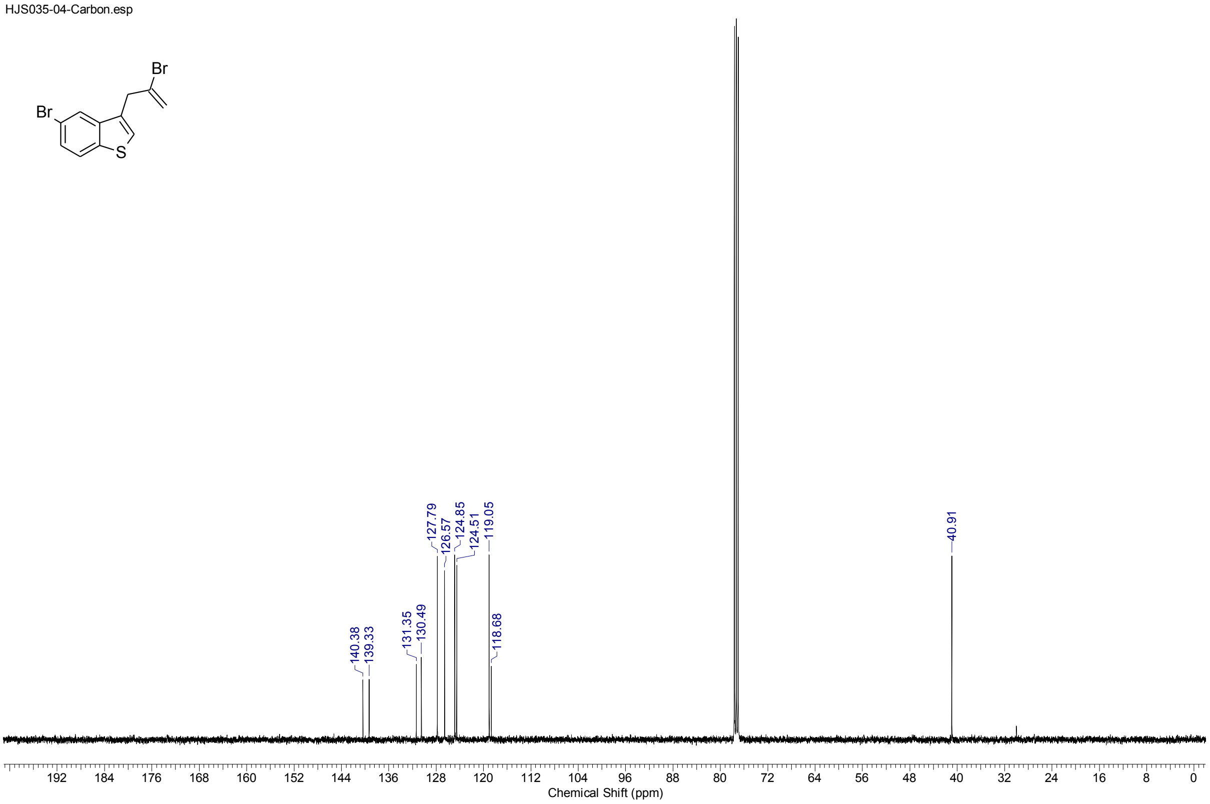

Supplementary Figure 38.

6g H^1 NMR (400 MHz, $CDCl_3$)

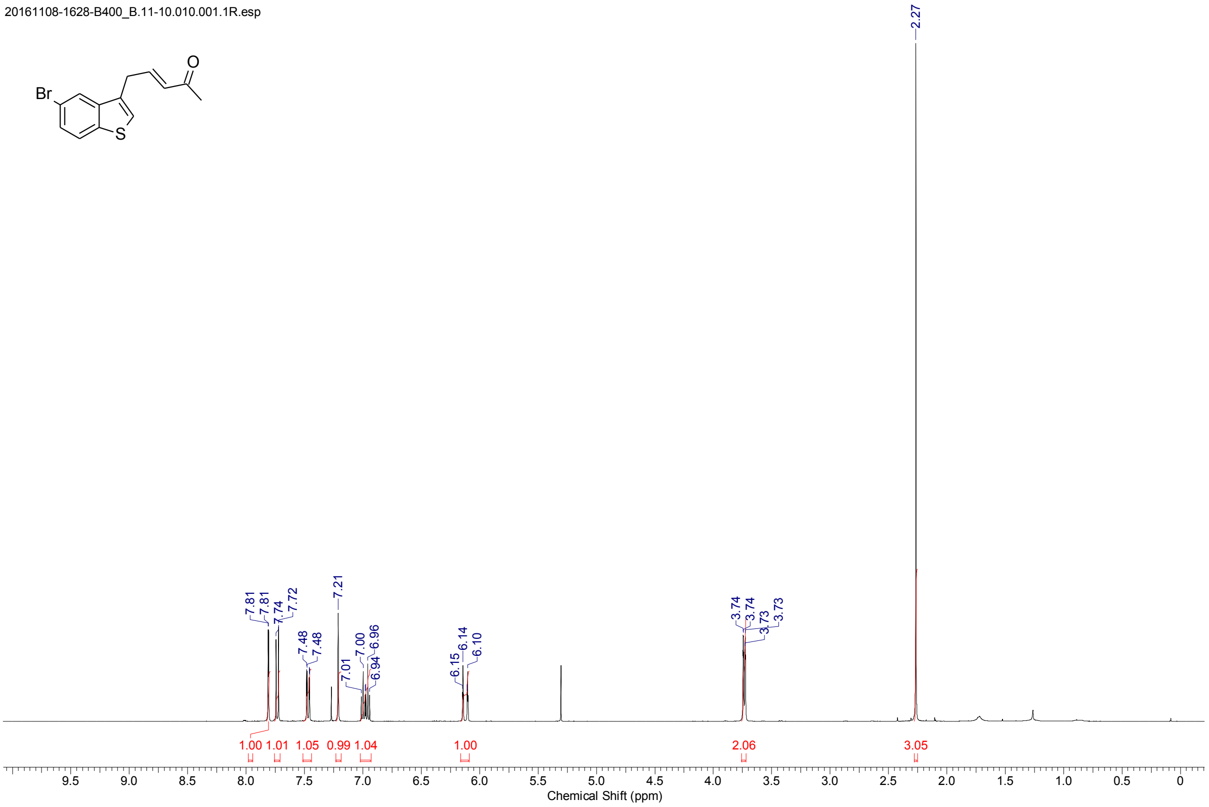

6g C^{13} NMR (101 MHz, $CDCl_3$)

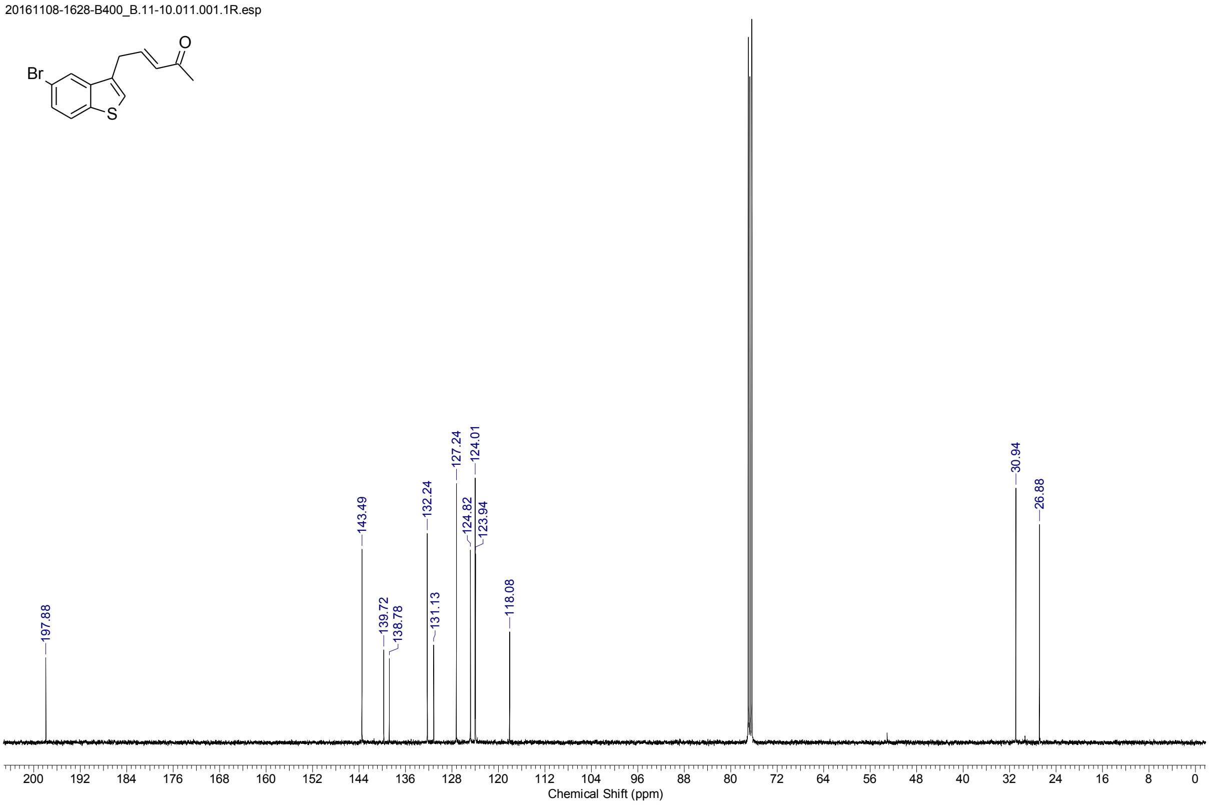

Supplementary Figure 39.

6h H^1 NMR (400 MHz, $CDCl_3$)

20161108-1716-B400_B.11-40.010.001.1R.esp

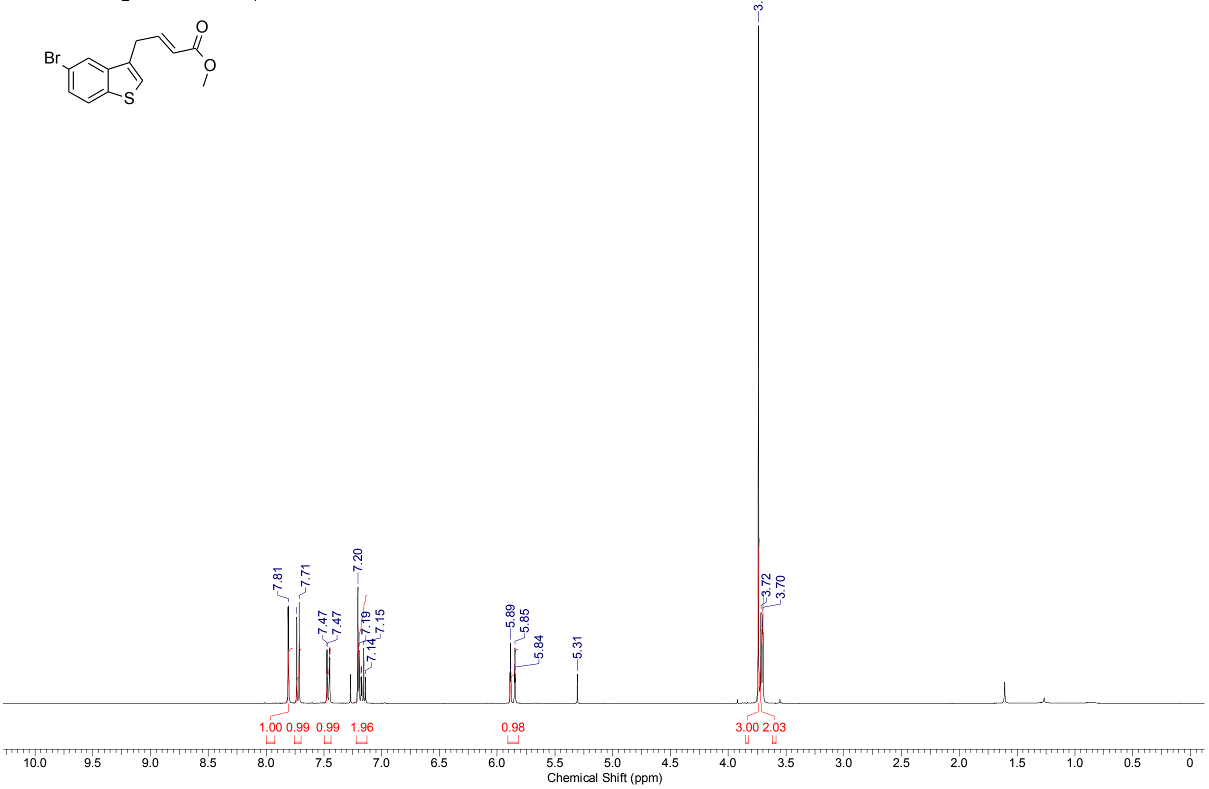

6h C^{13} NMR (101 MHz, $CDCl_3$)

20161108-1716-B400_B.11-40.012.001.1R.esp

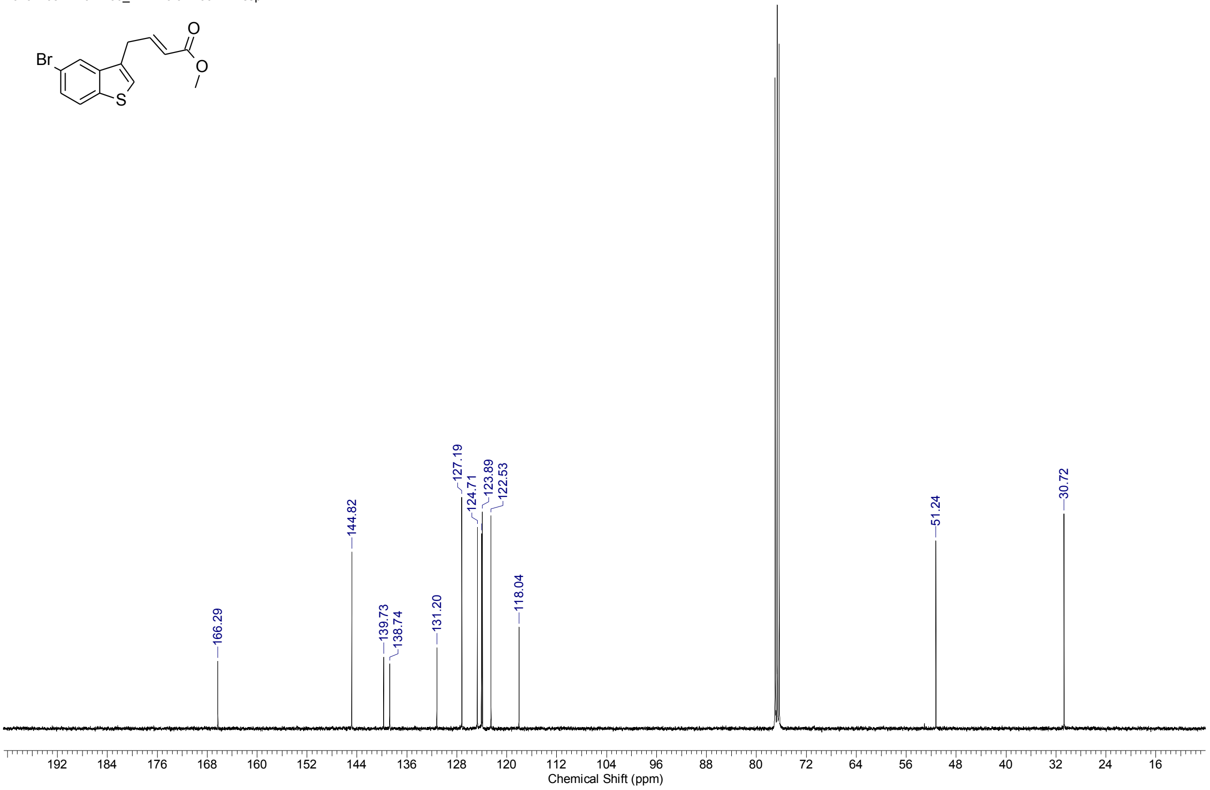

Supplementary Figure 40.

8a ^{1}H NMR (400 MHz, $CDCl_3$)

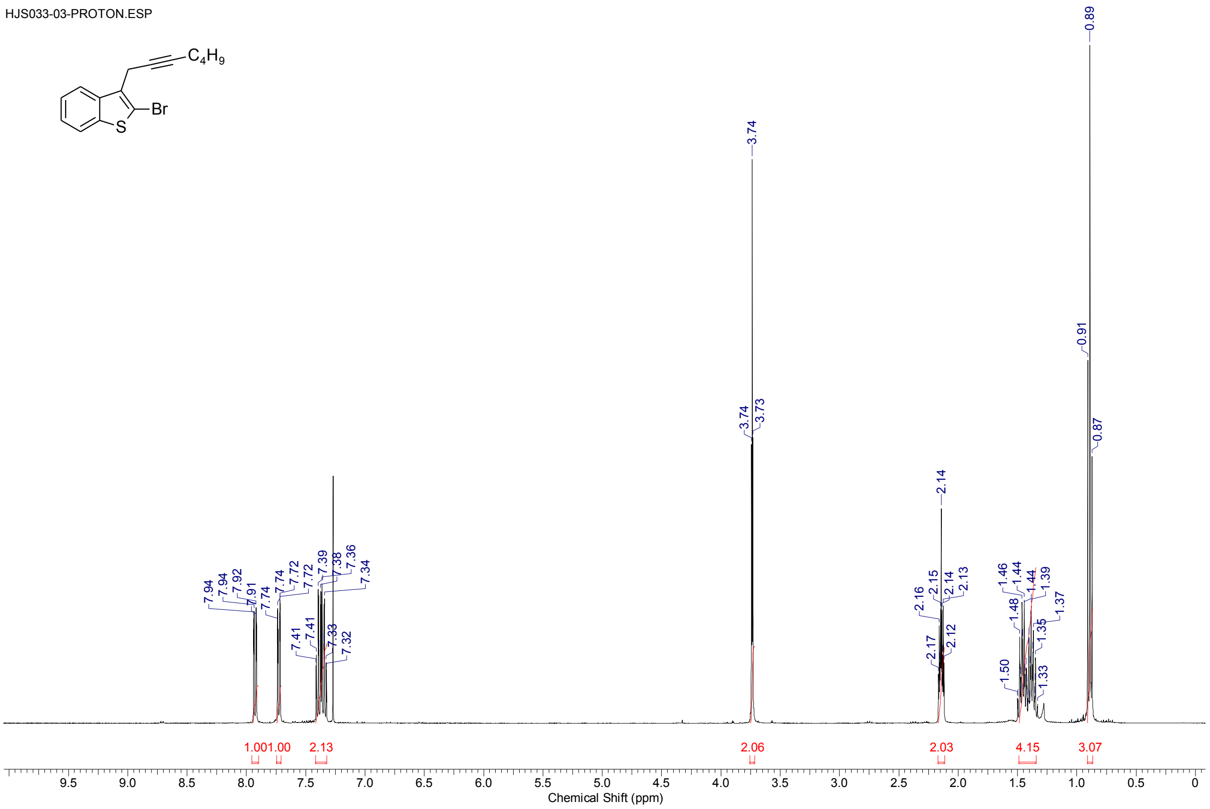

8a ^{13}C NMR (101 MHz, $CDCl_3$)

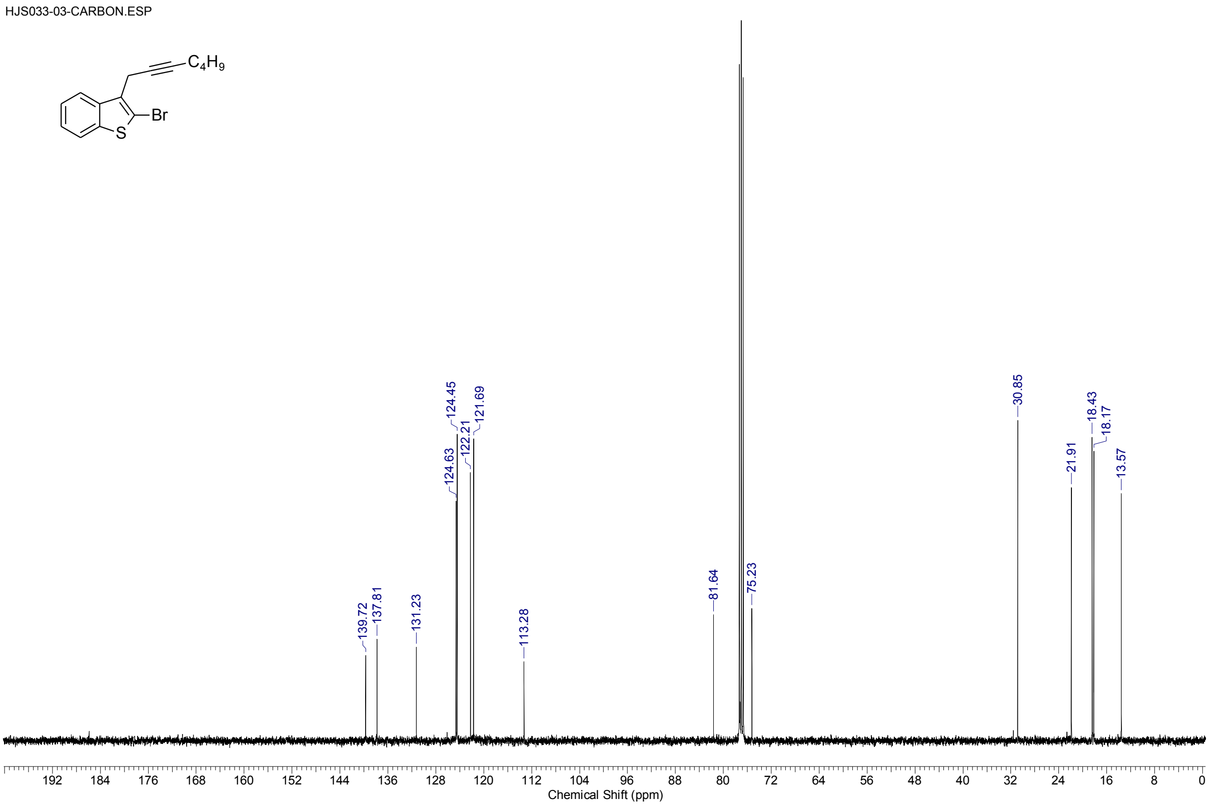

Supplementary Figure 41.

8b ^{1}H NMR (400 MHz, $CDCl_3$)

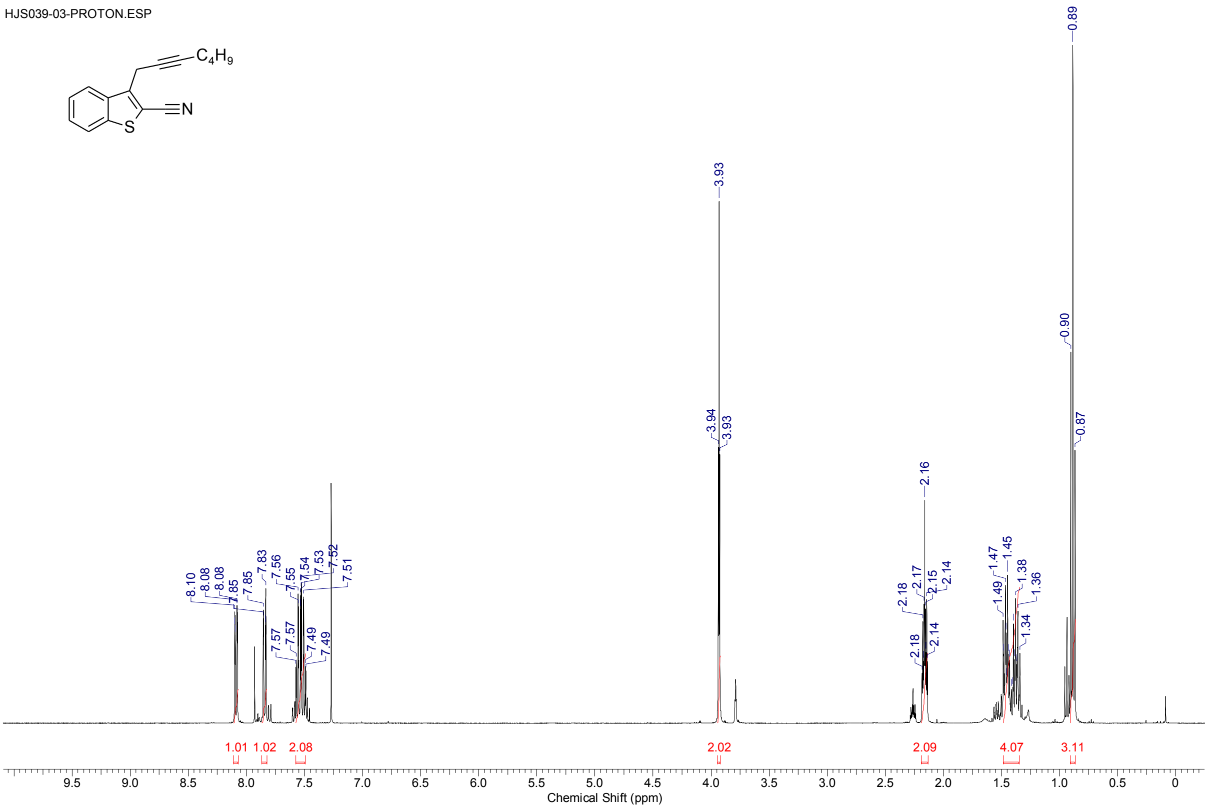

8b ^{13}C NMR (101 MHz, $CDCl_3$)

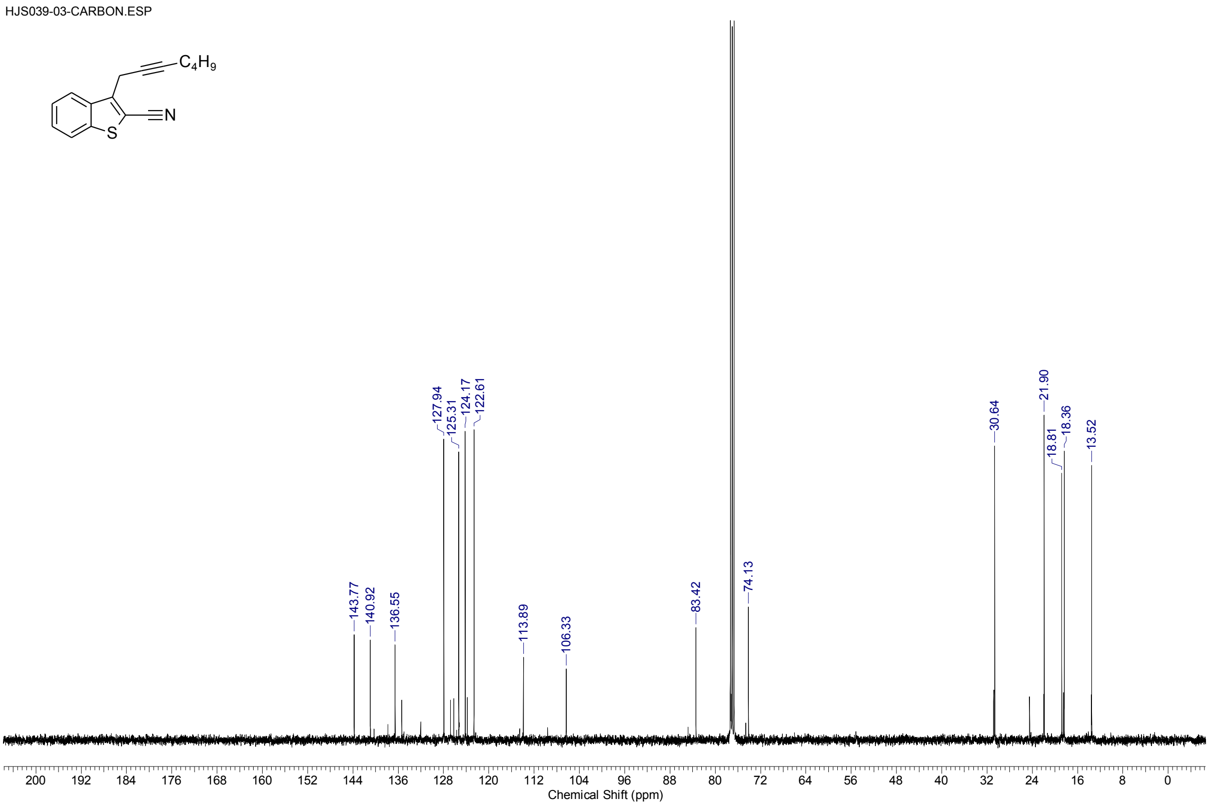

Supplementary Figure 42.

8c ^{1}H NMR (400 MHz, $CDCl_3$)

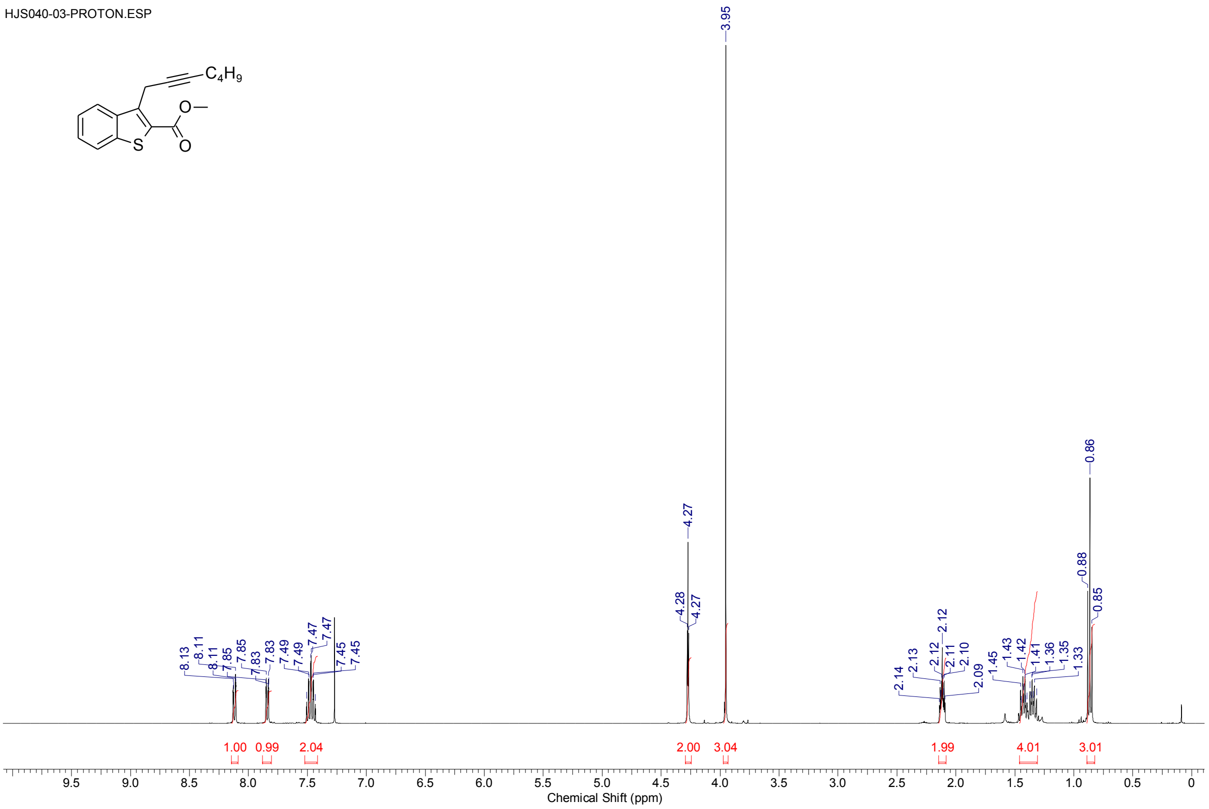

8c ^{13}C NMR (101 MHz, $CDCl_3$)

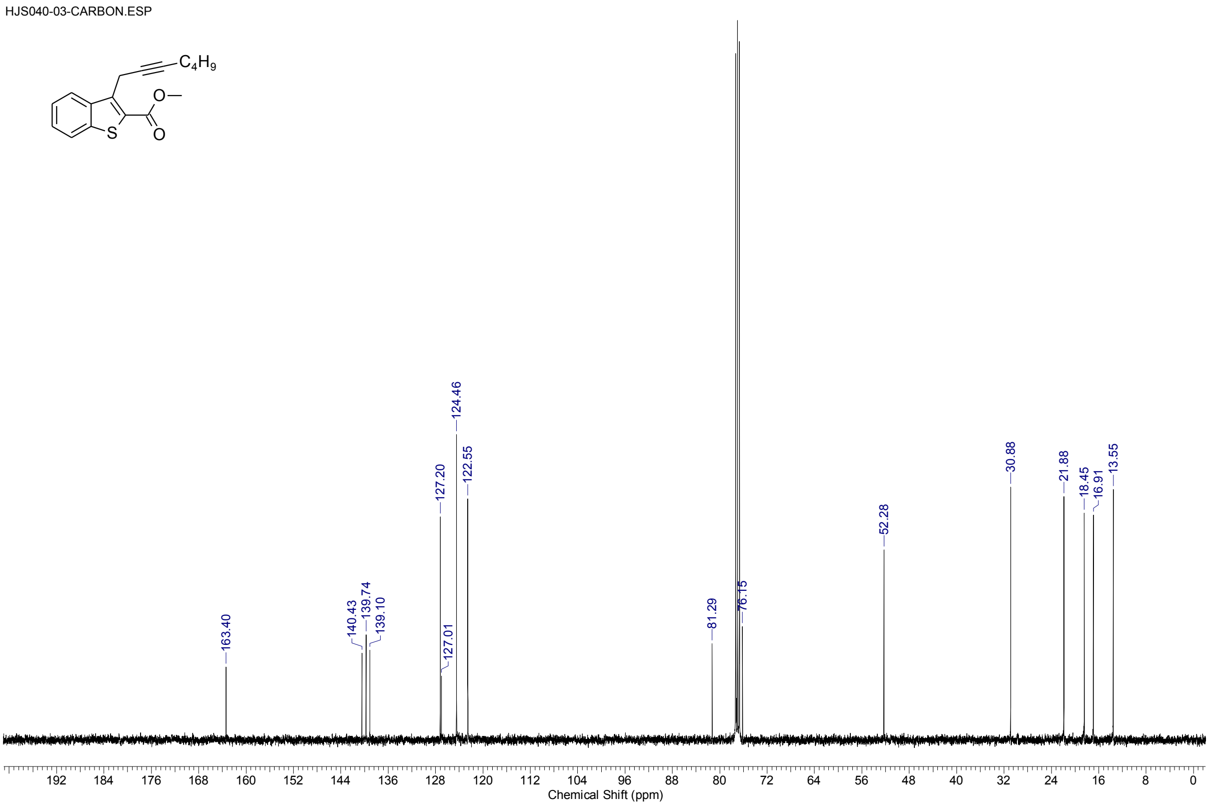

Supplementary Figure 43.

8d ^{1}H NMR (400 MHz, $CDCl_3$)

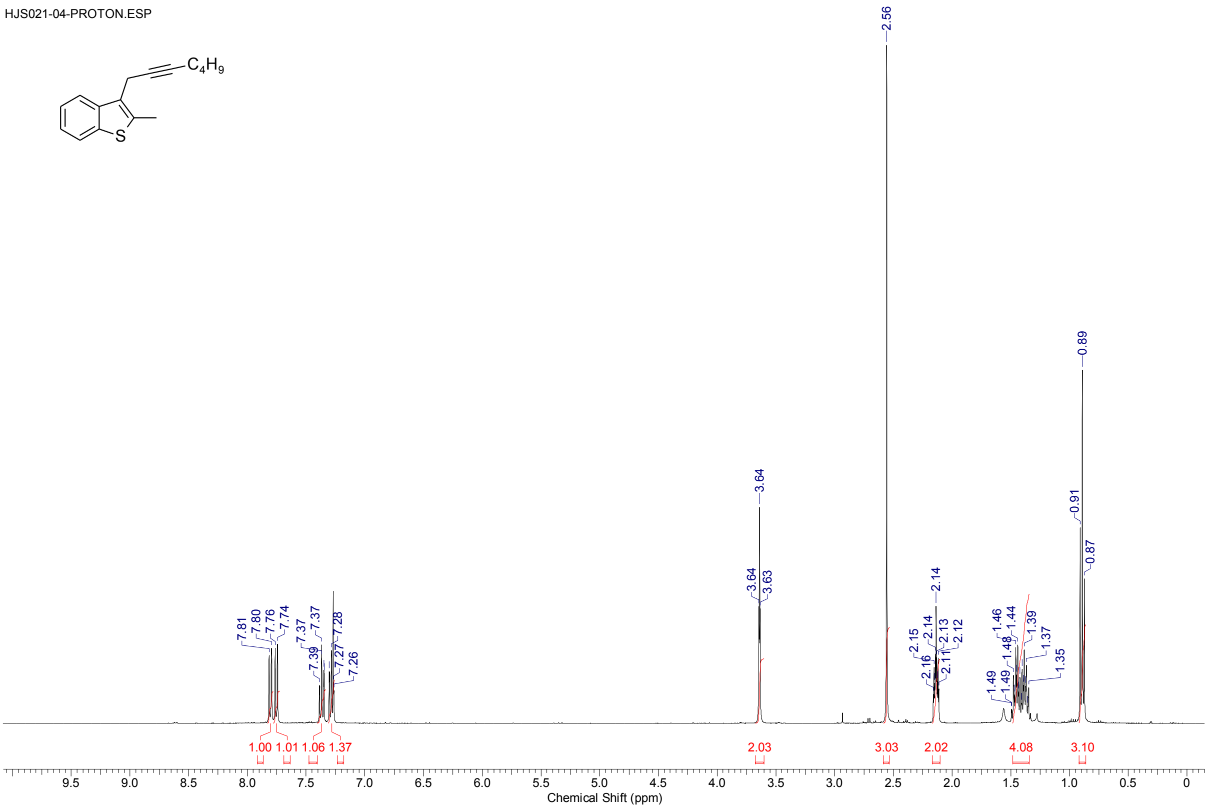

8d ^{13}C NMR (101 MHz, $CDCl_3$)

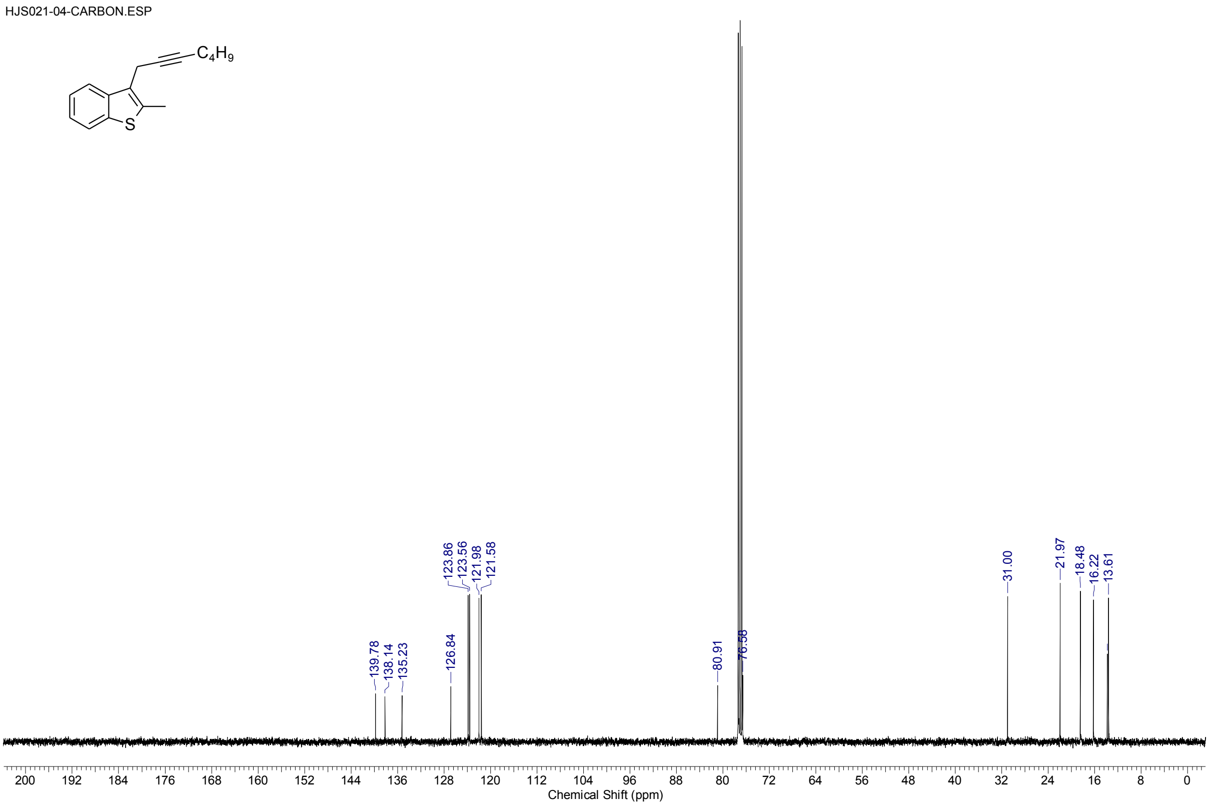

Supplementary Figure 44.

8e ^{1}H NMR (400 MHz, $CDCl_3$)

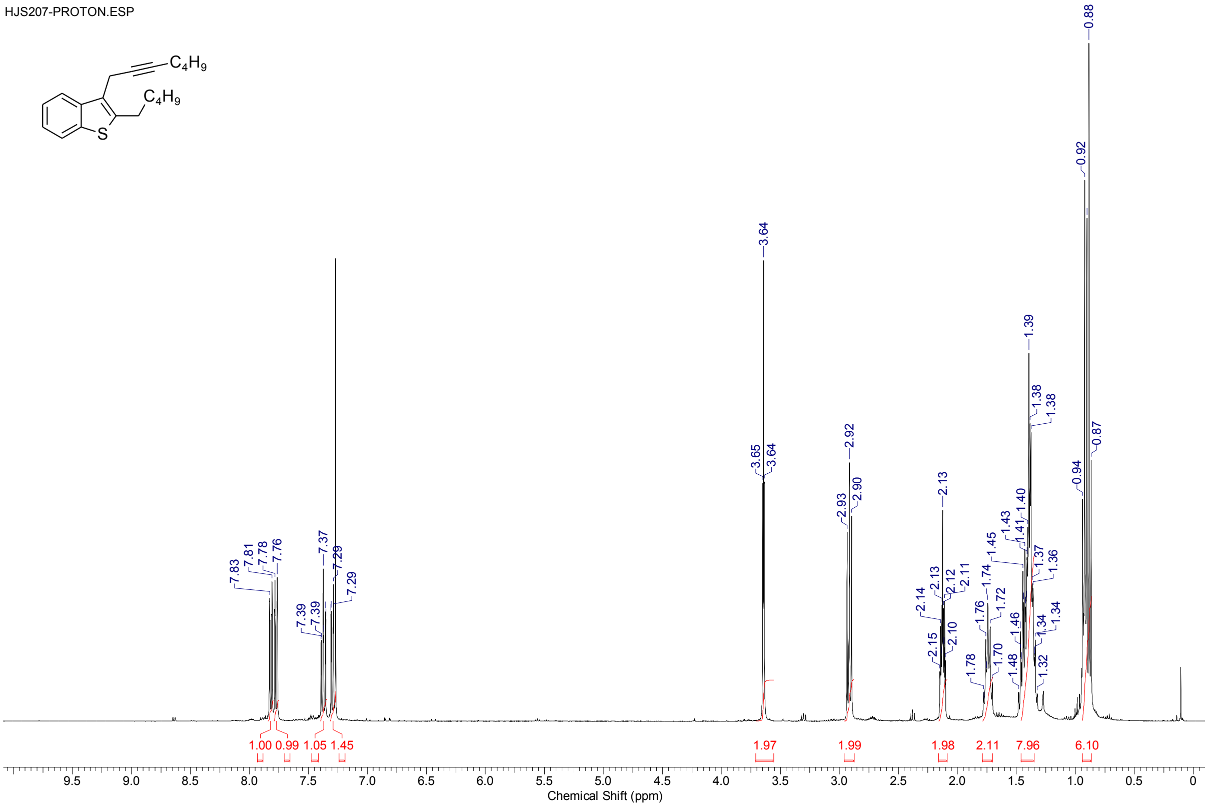

8e ^{13}C NMR (101 MHz, $CDCl_3$)

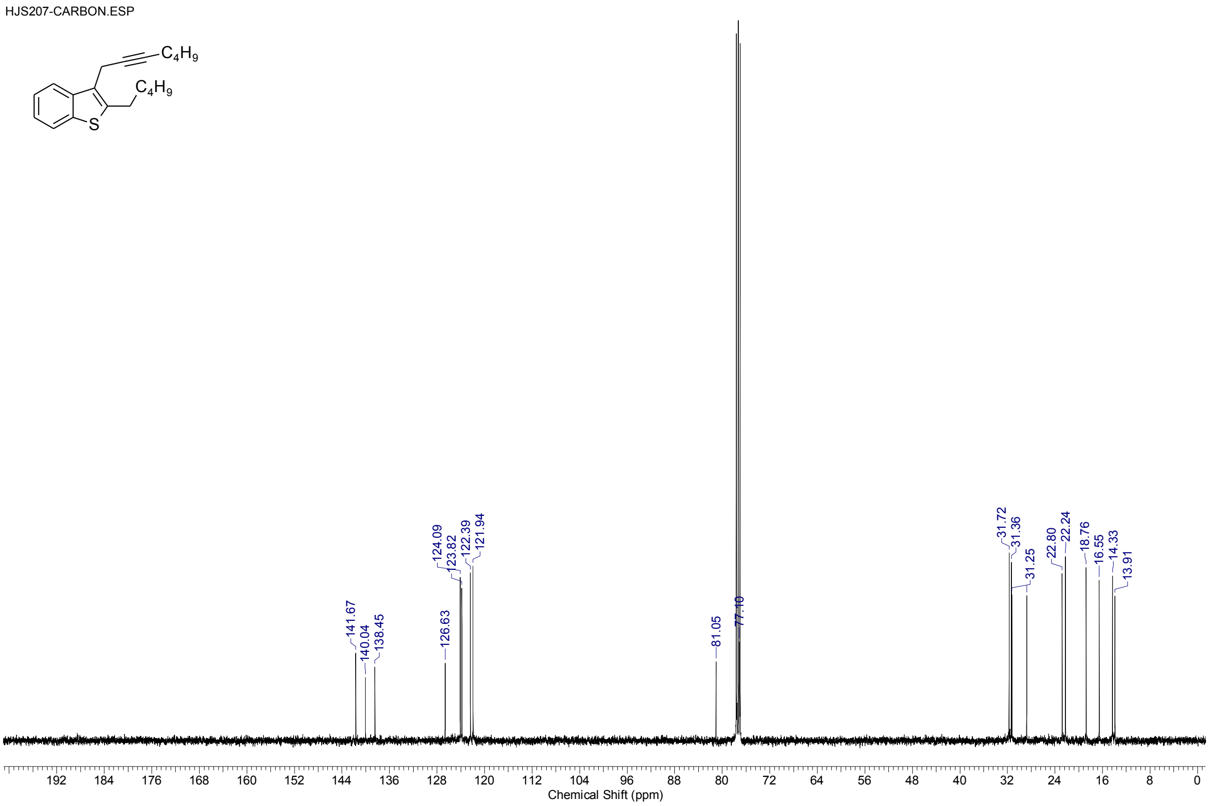

Supplementary Figure 45.

8f ^{1}H NMR (400 MHz, $CDCl_3$)

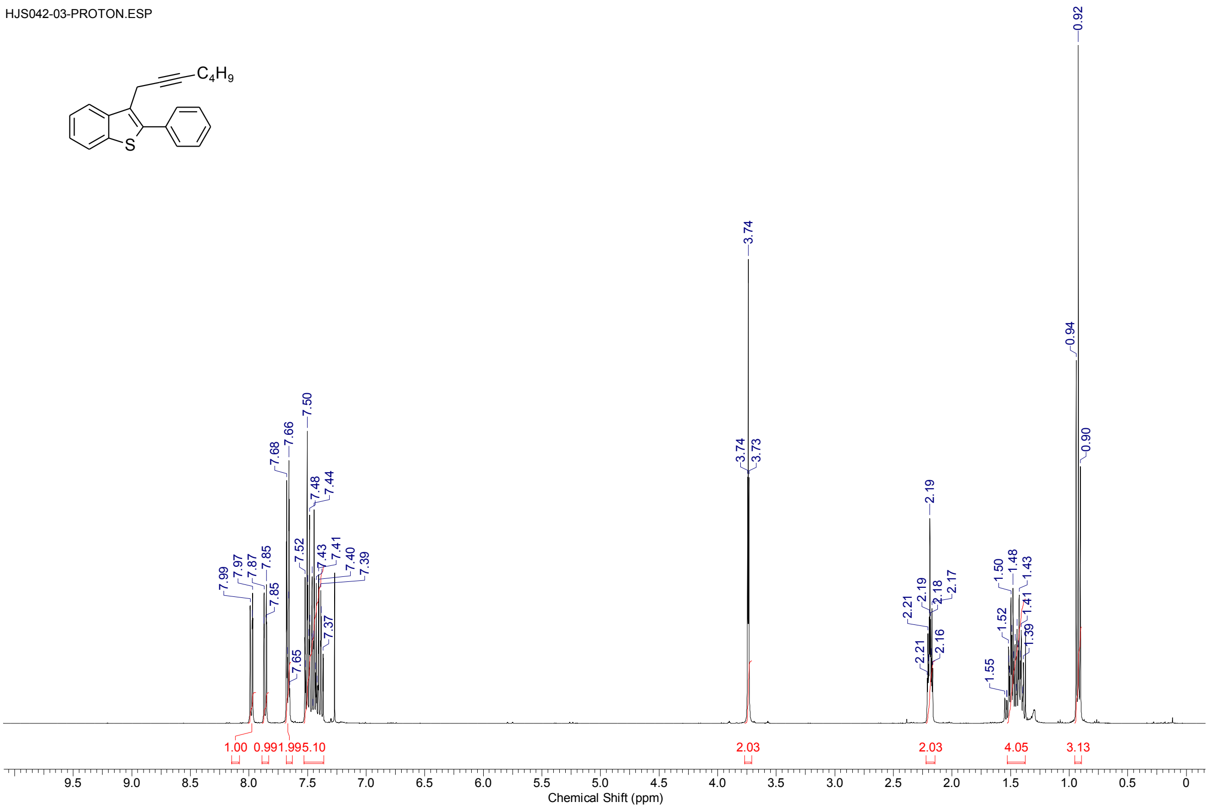

8f ^{13}C NMR (101 MHz, $CDCl_3$)

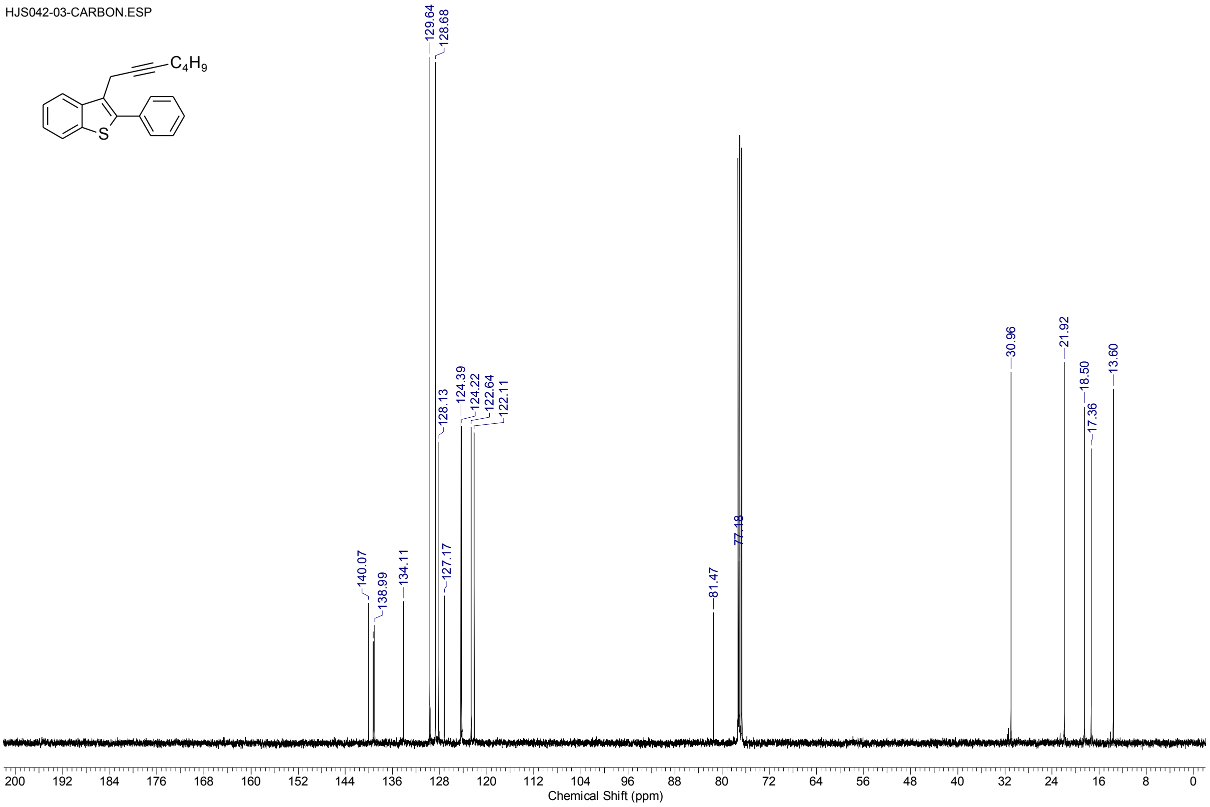

Supplementary Figure 46.

8g ^{1}H NMR (400 MHz, $CDCl_3$)

20161020-1649-B400_B.11-30.010.001.1r.esp

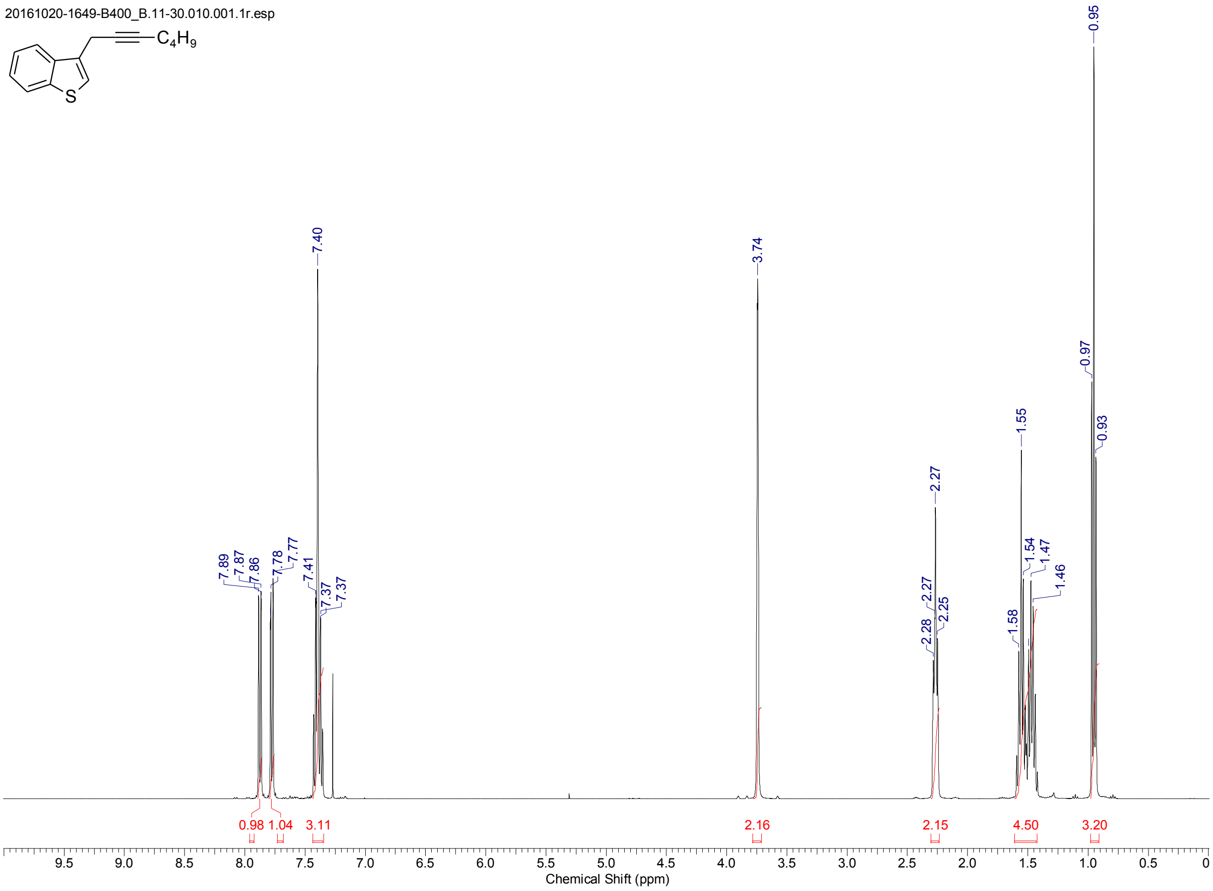

8g ^{13}C NMR (101 MHz, $CDCl_3$)

20161020-1649-B400_B.11-30.012.001.1r.esp

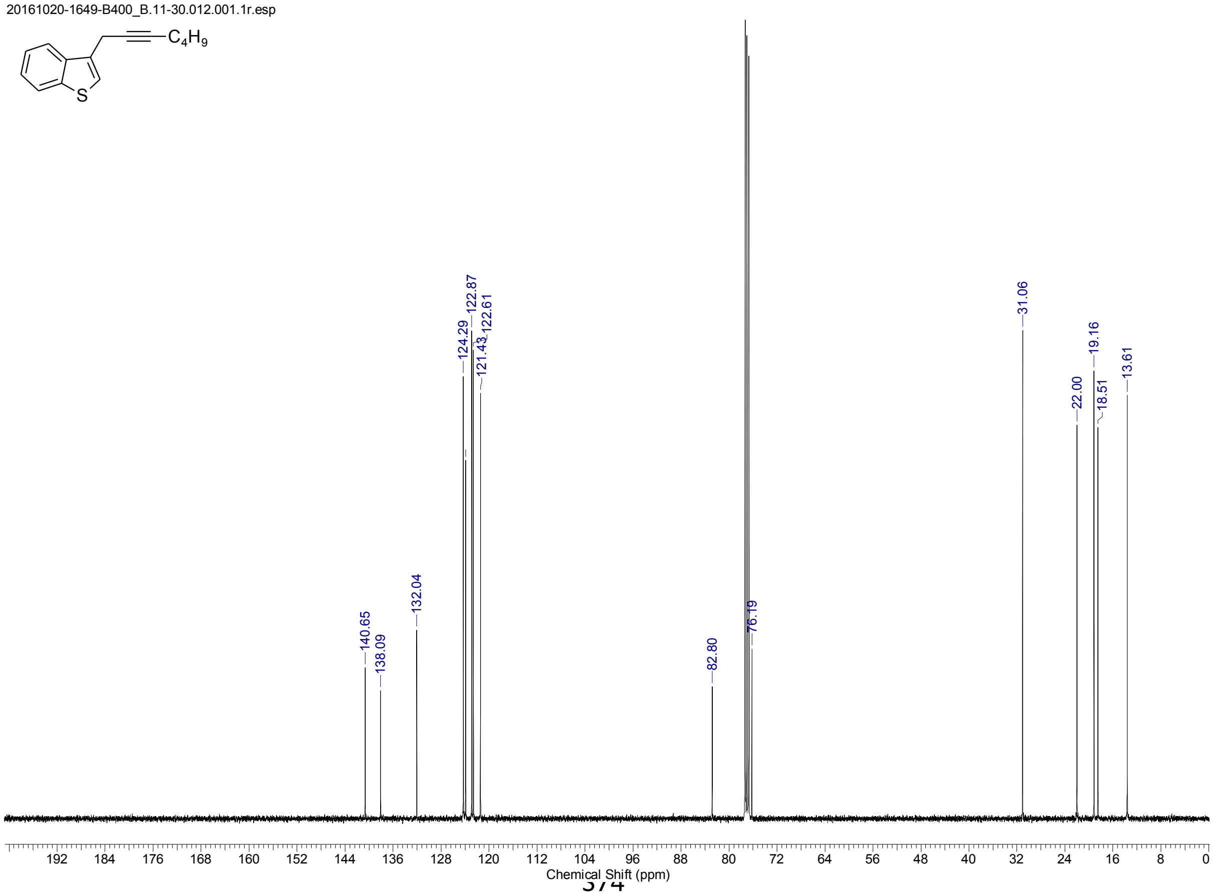

Supplementary Figure 47.

8h ^{1}H NMR (400 MHz, $CDCl_3$)

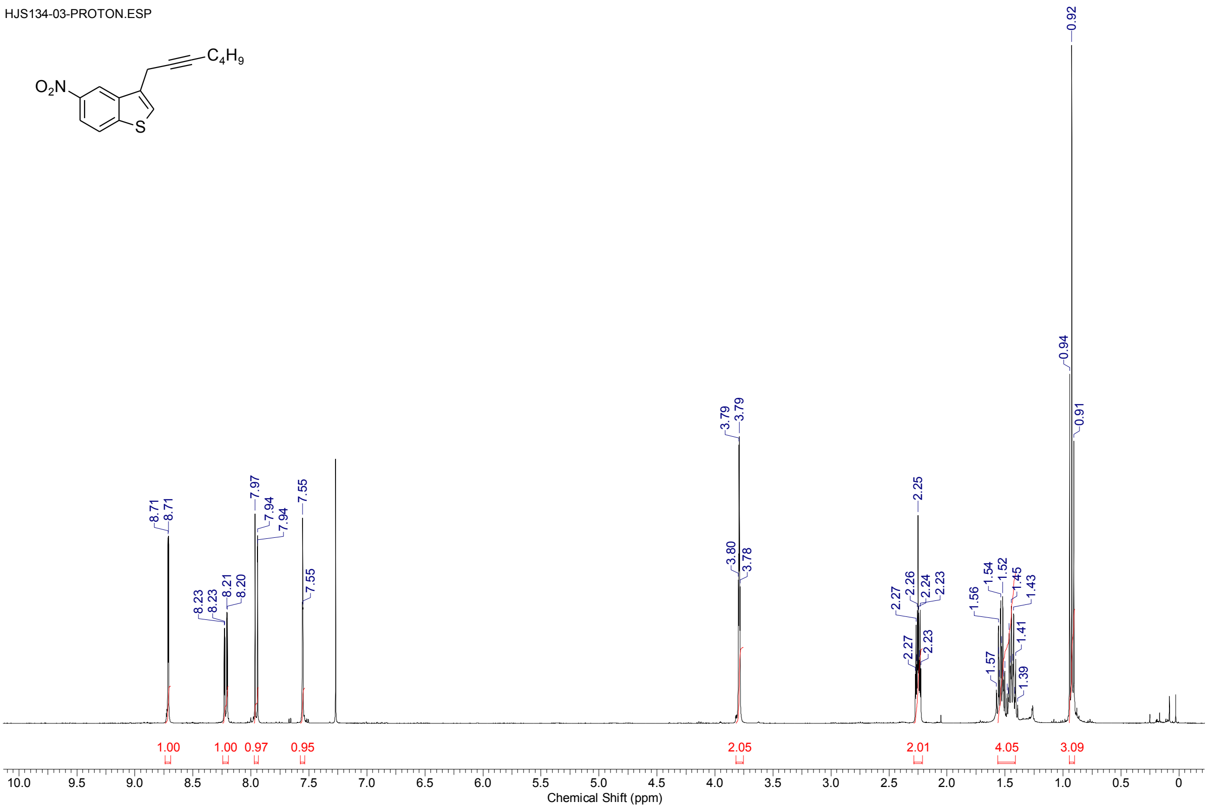

8h ^{13}C NMR (101 MHz, $CDCl_3$)

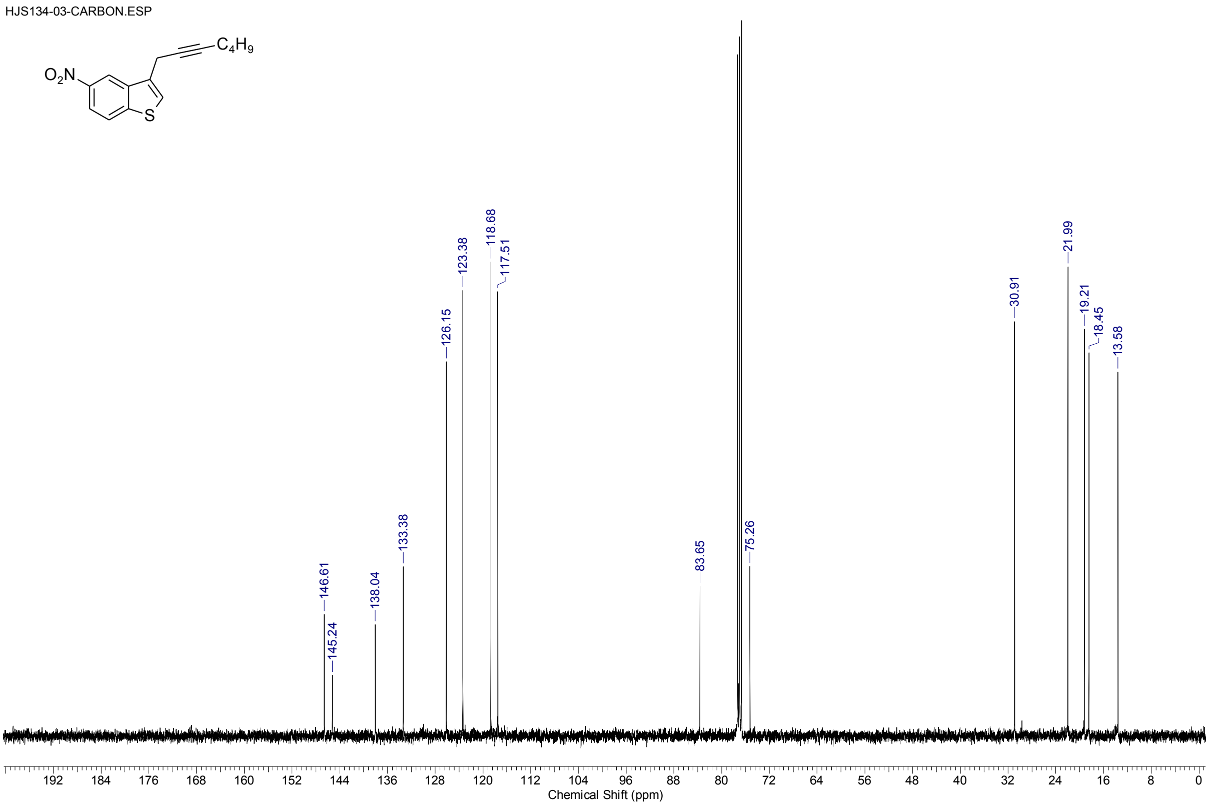

Supplementary Figure 48.

8i ^{1}H NMR (400 MHz, $CDCl_3$)

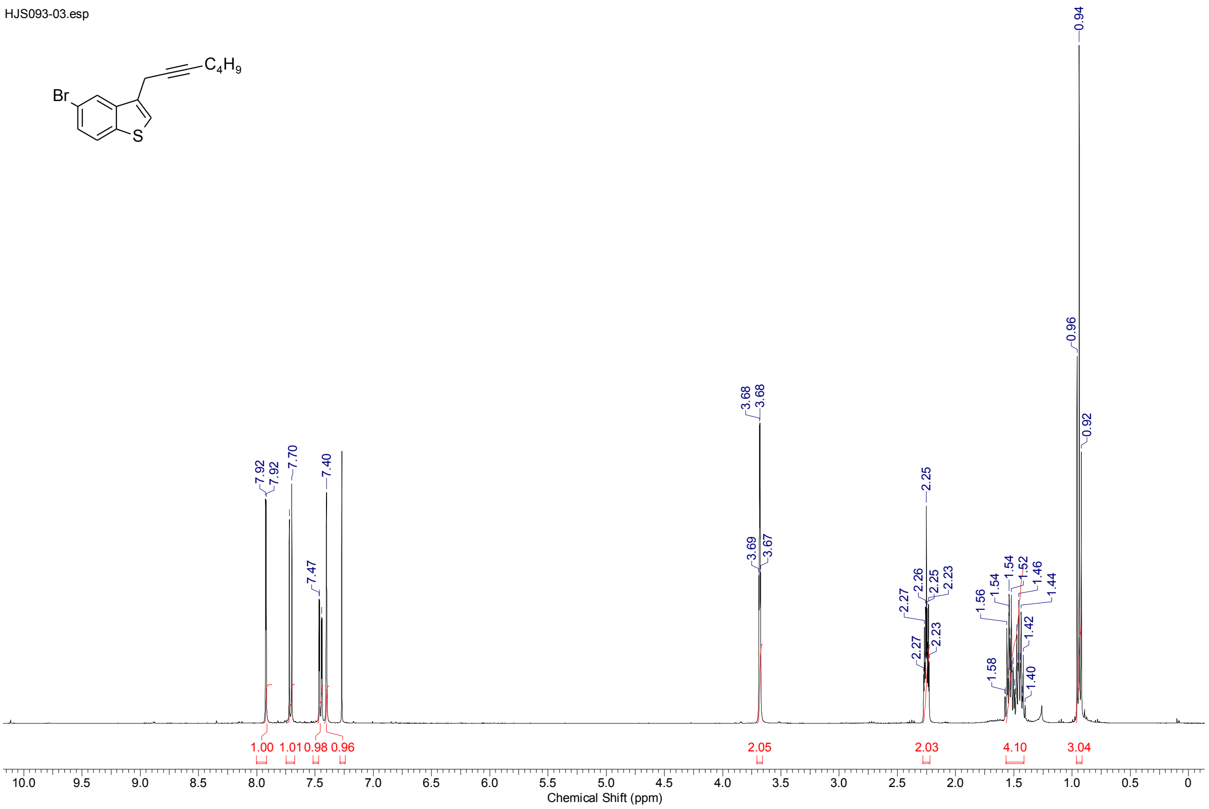

8i ^{13}C NMR (101 MHz, $CDCl_3$)

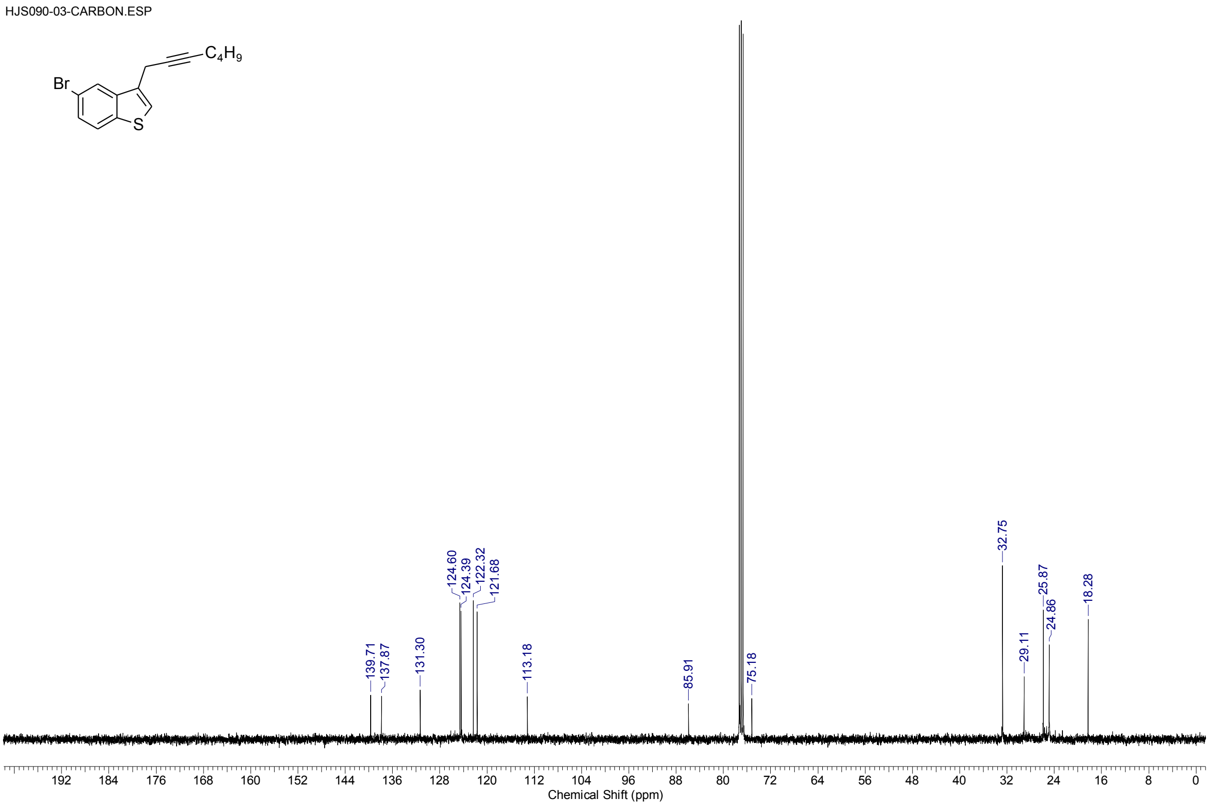

Supplementary Figure 49.

8j ^{1}H NMR (400 MHz, $CDCl_3$)

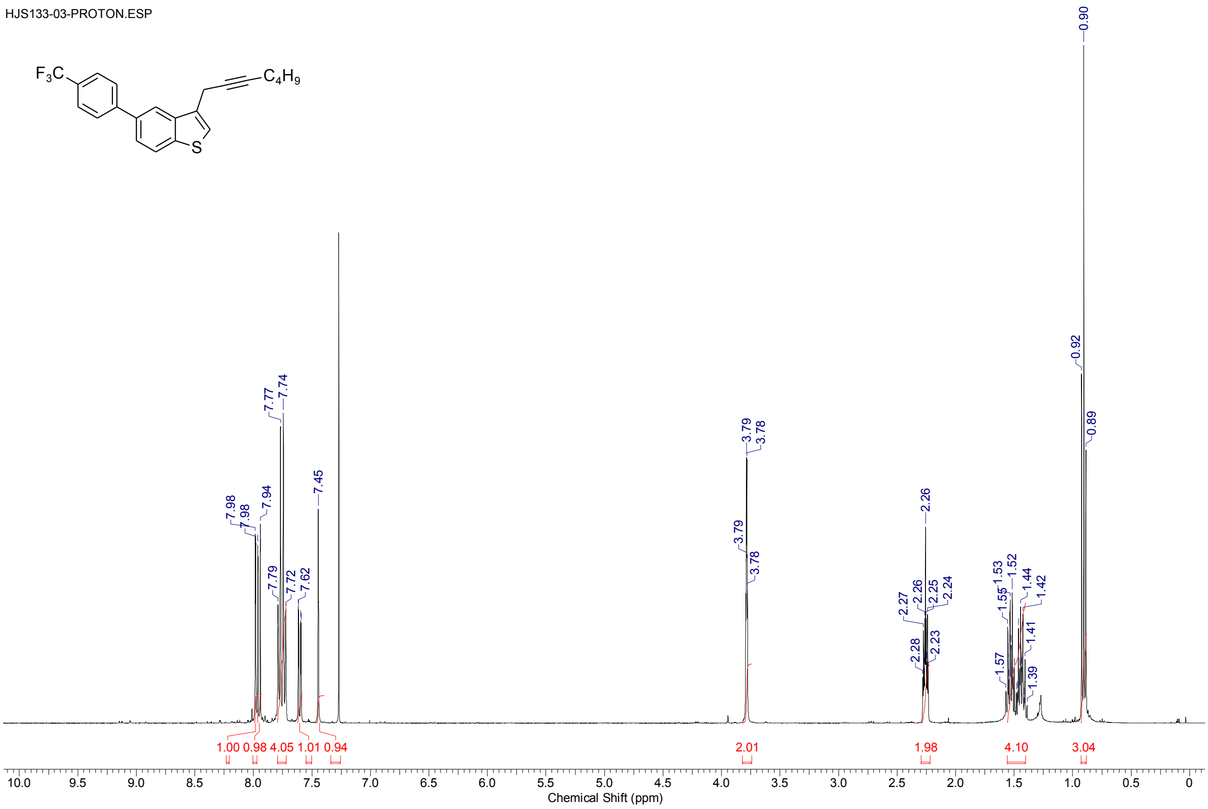

8j ^{13}C NMR (101 MHz, $CDCl_3$)

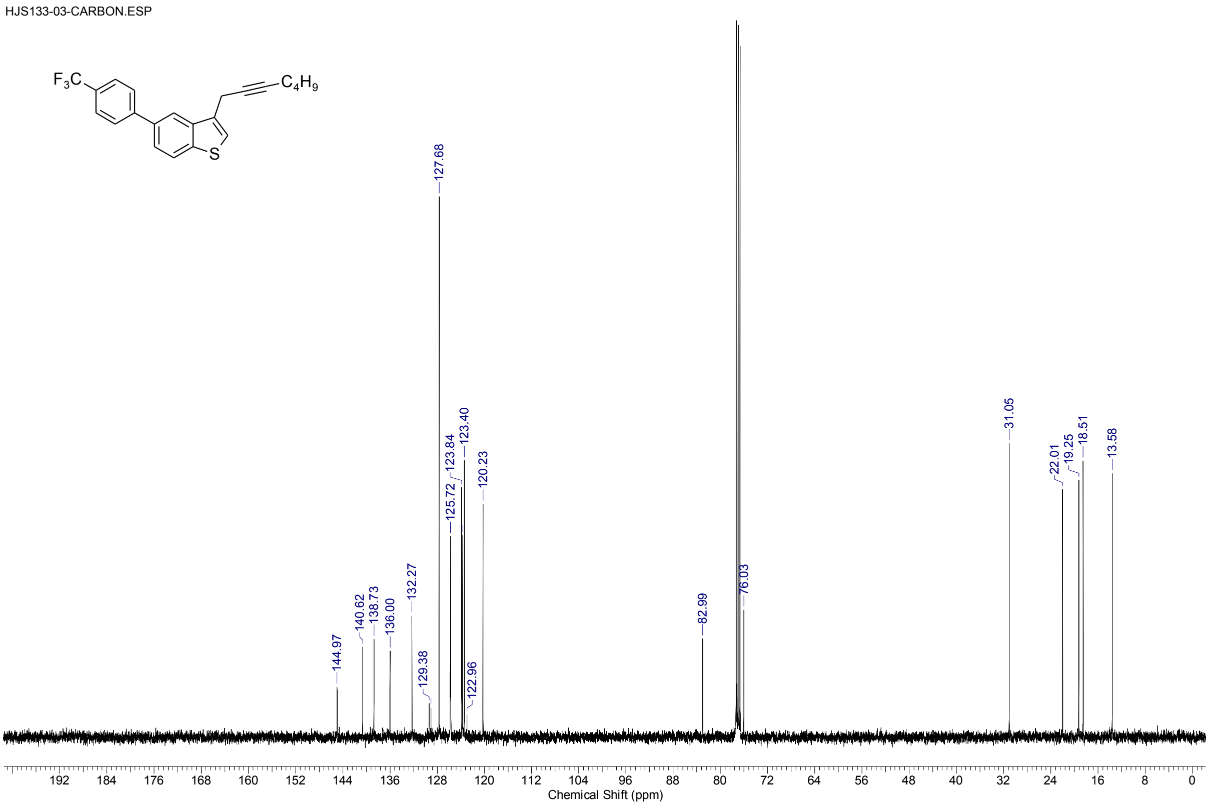

Supplementary Figure 50.
8k ^{1}H NMR (400 MHz, $CDCl_3$)

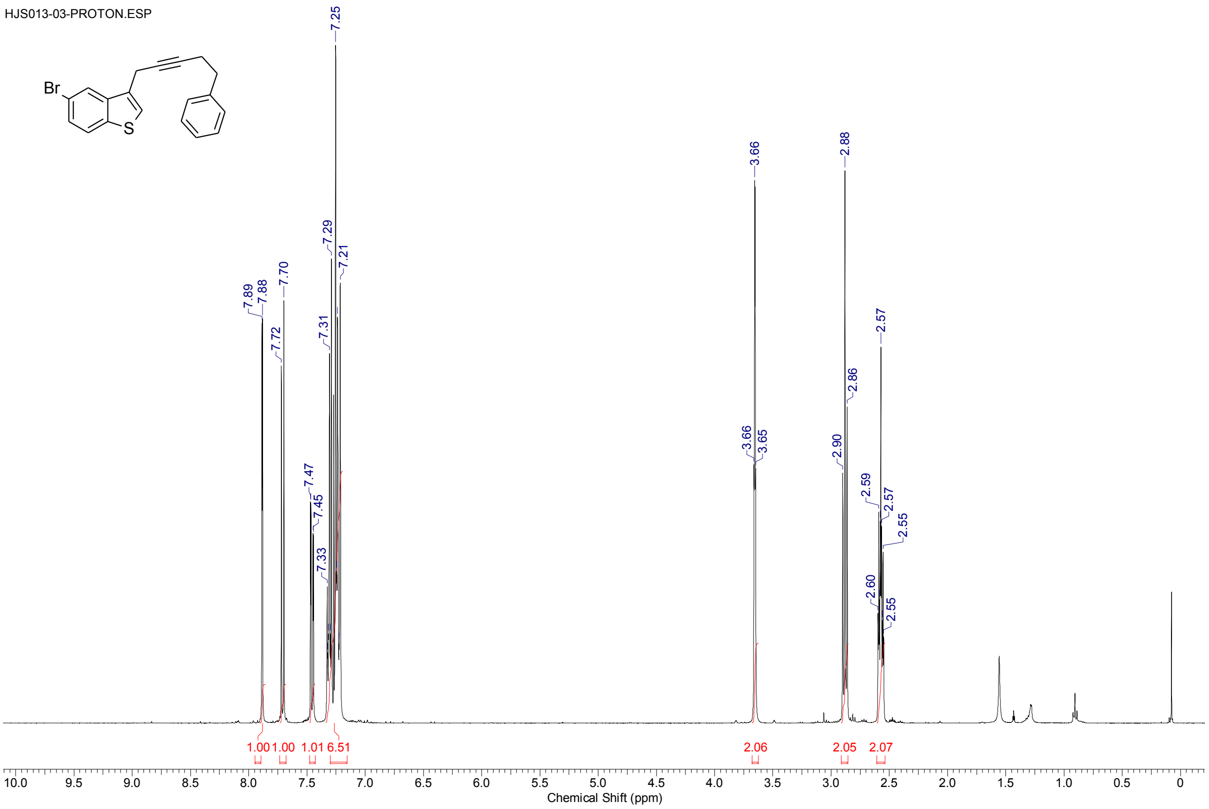

8k ^{13}C NMR (101 MHz, $CDCl_3$)

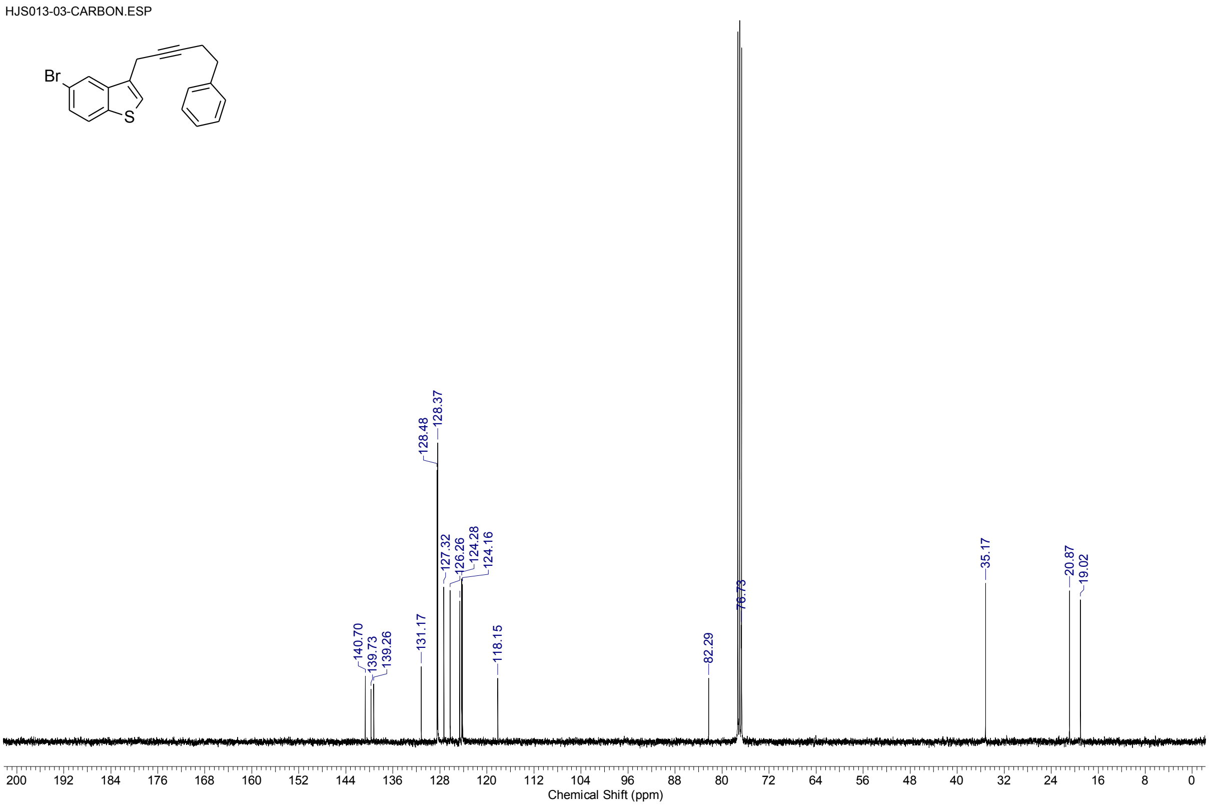

Supplementary Figure 51.

8l ^{1}H NMR (400 MHz, $CDCl_3$)

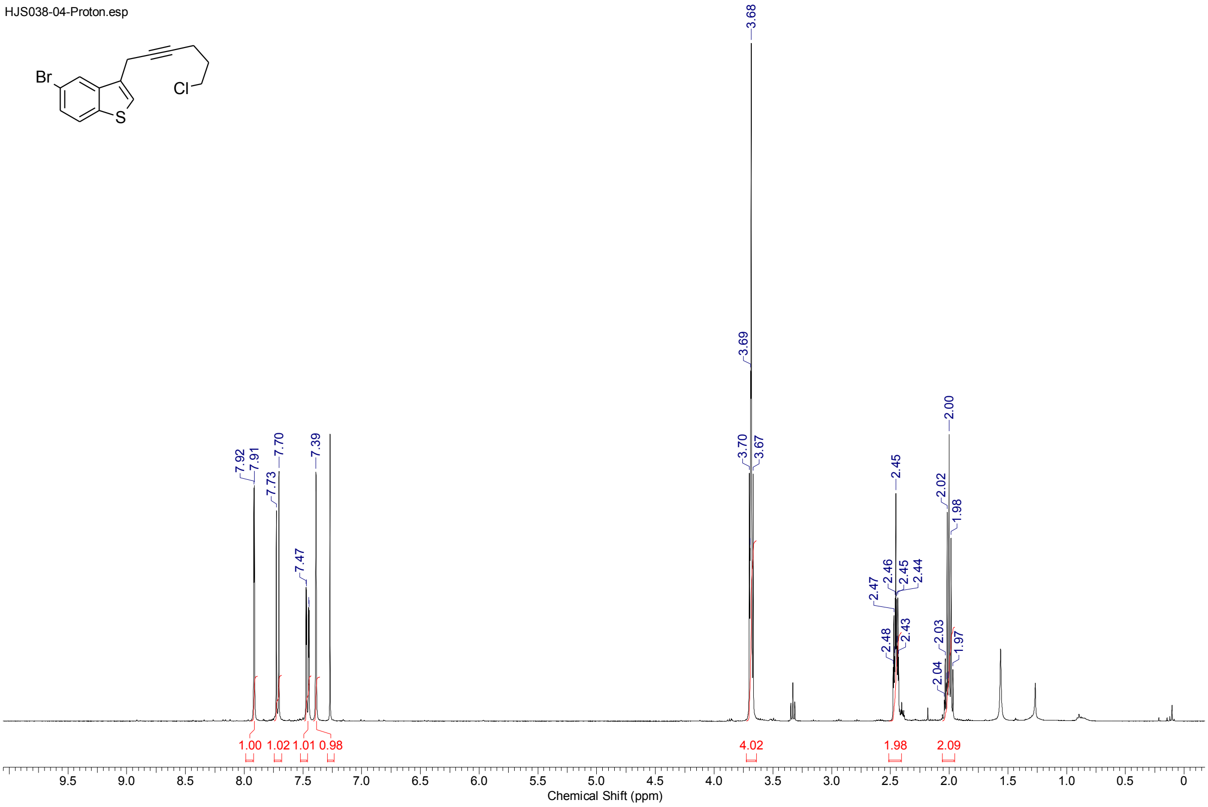

8l ^{13}C NMR (101 MHz, $CDCl_3$)

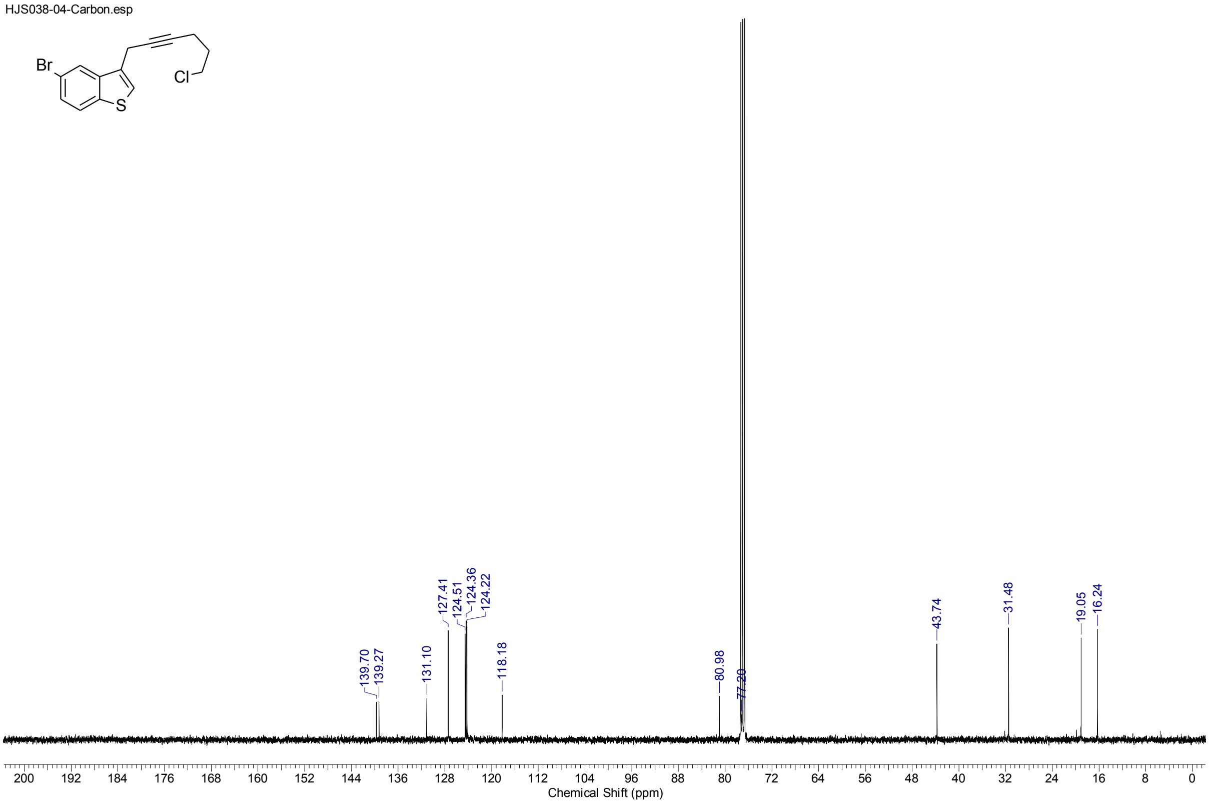

Supplementary Figure 52.

8m ^{1}H NMR (400 MHz, $CDCl_3$)

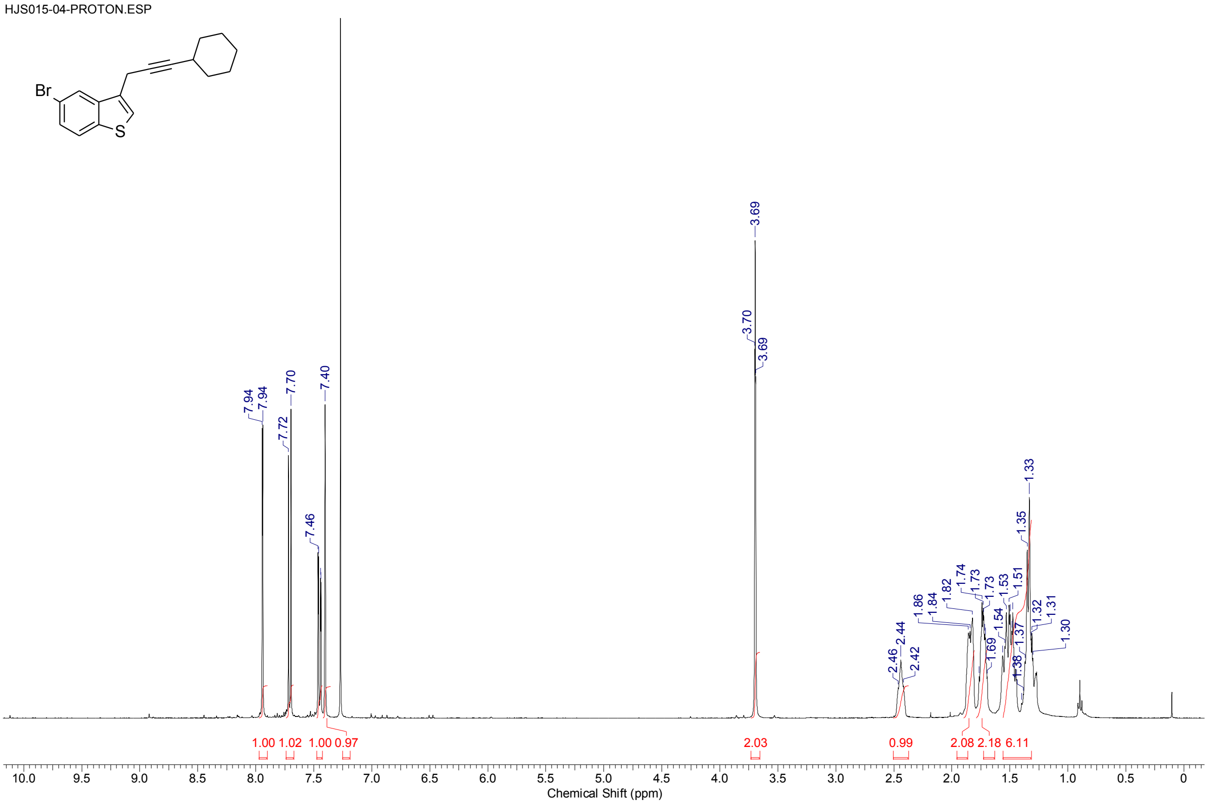

8m ^{13}C NMR (101 MHz, $CDCl_3$)

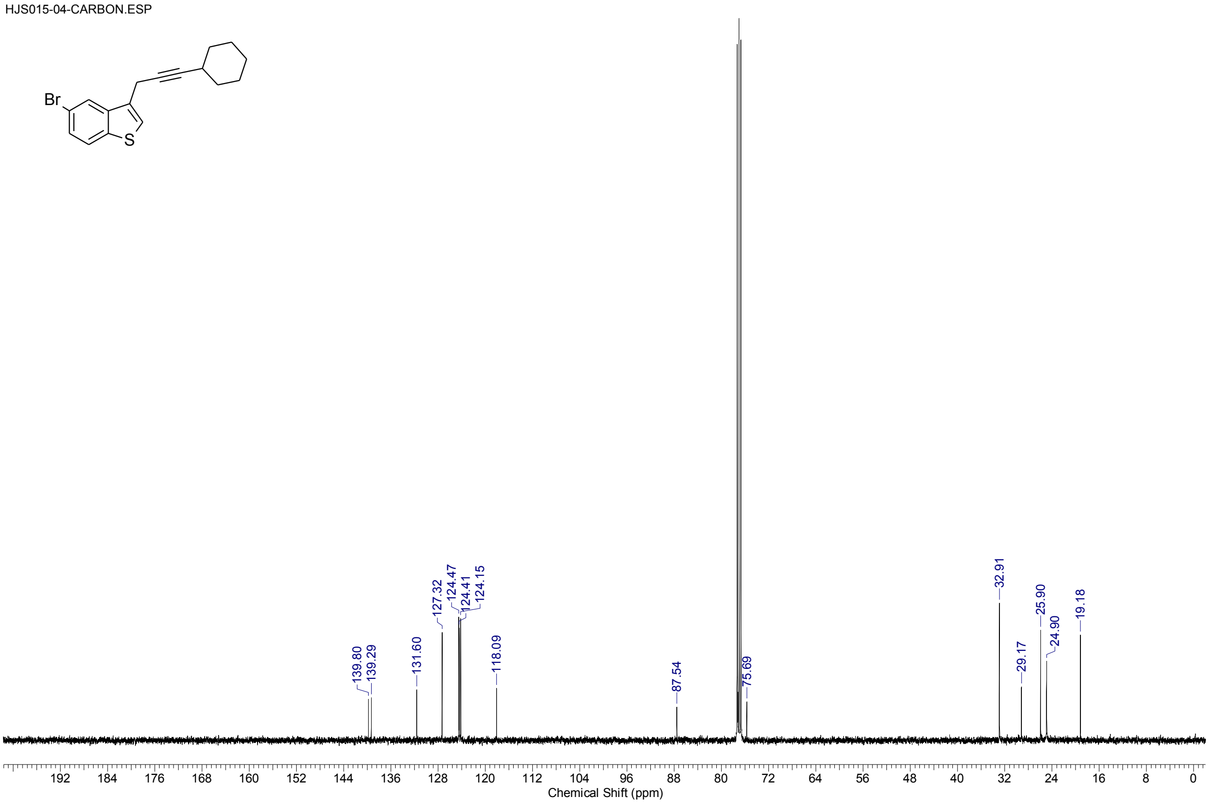

Supplementary Figure 53.

8n ^{1}H NMR (400 MHz, $CDCl_3$)

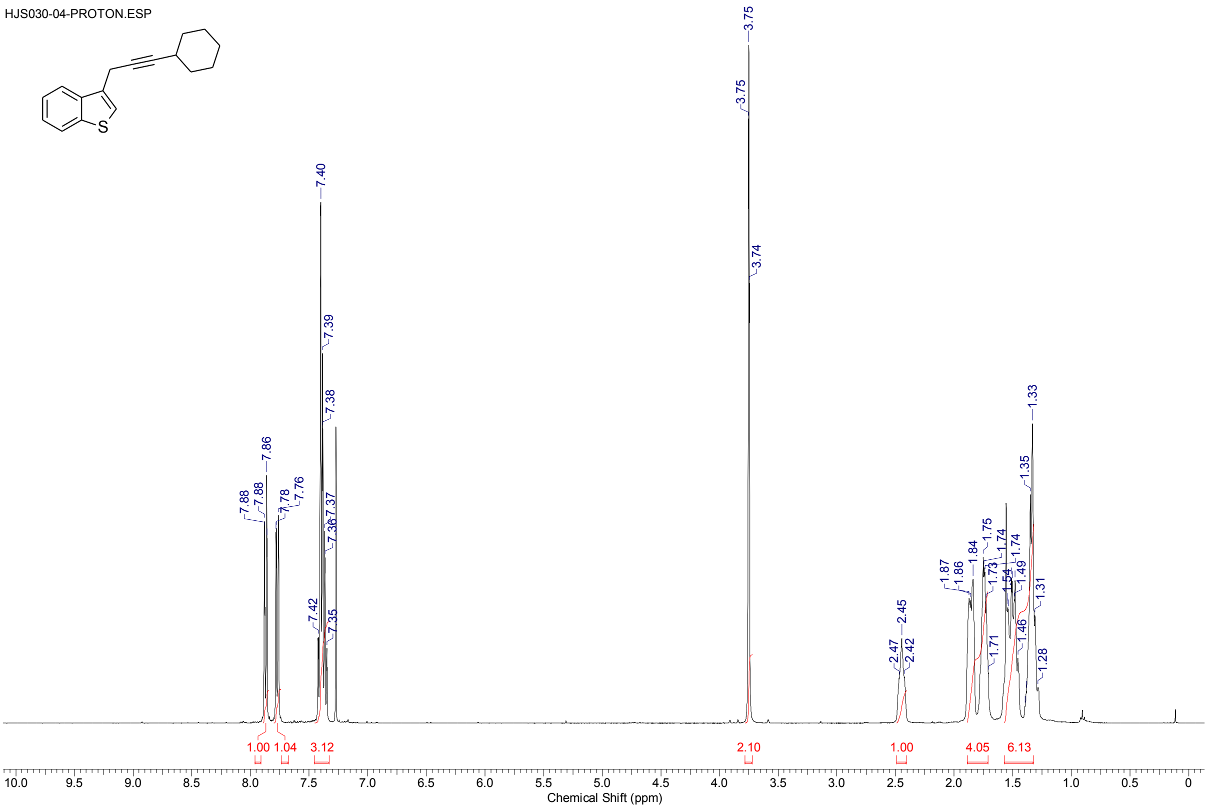

8n ^{13}C NMR (101 MHz, $CDCl_3$)

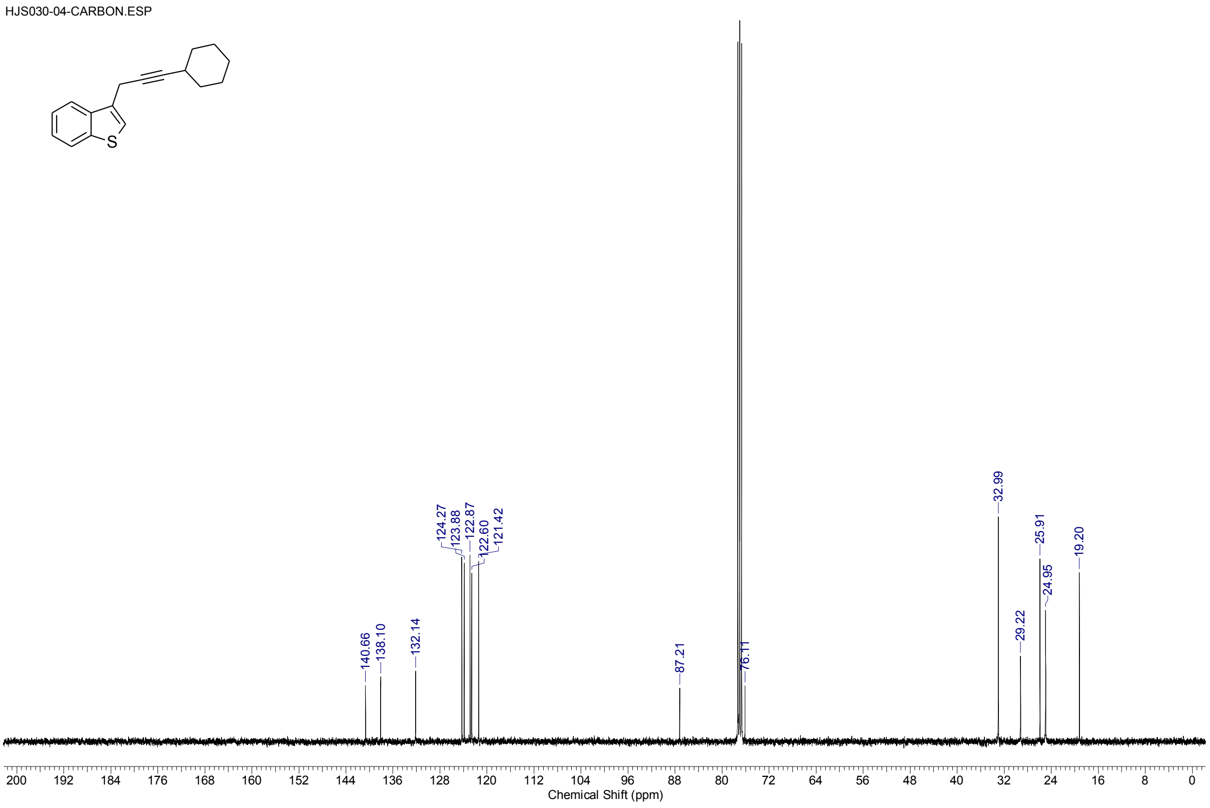

Supplementary Figure 54.

8o ^{1}H NMR (400 MHz, $CDCl_3$)

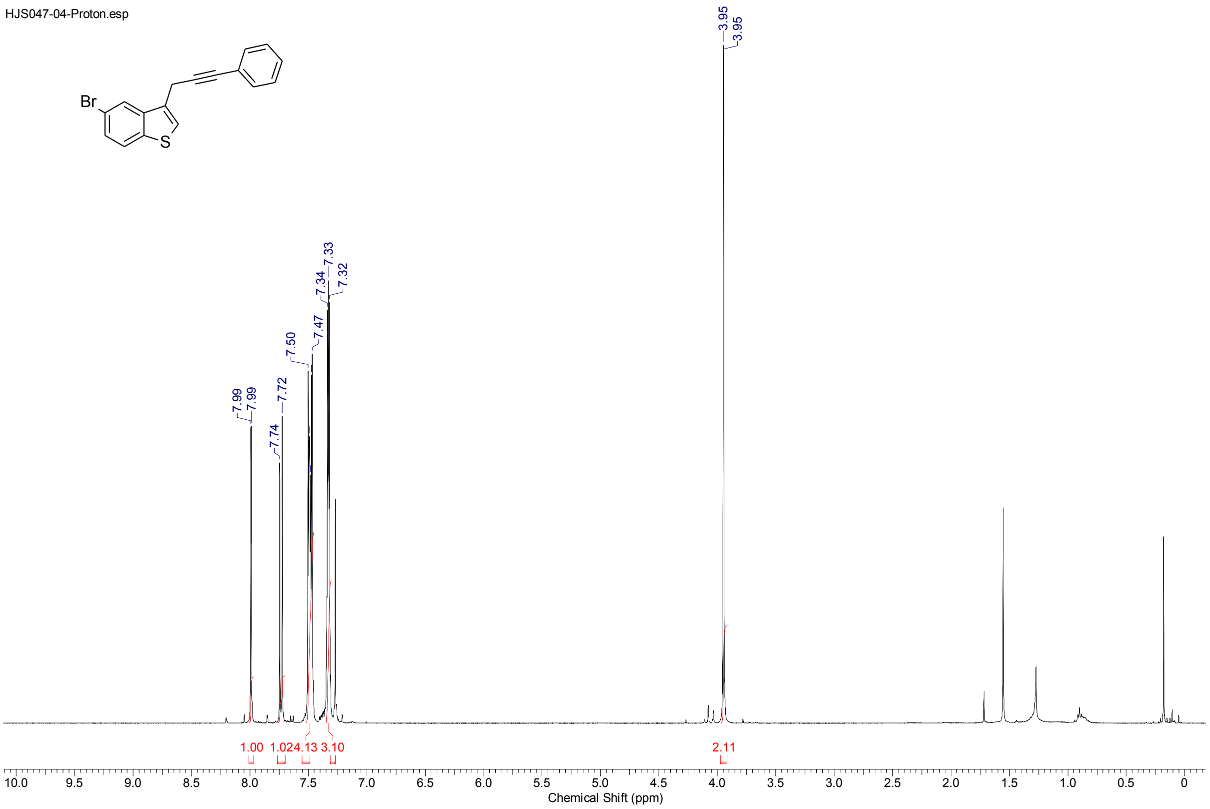

8o ^{13}C NMR (101 MHz, $CDCl_3$)

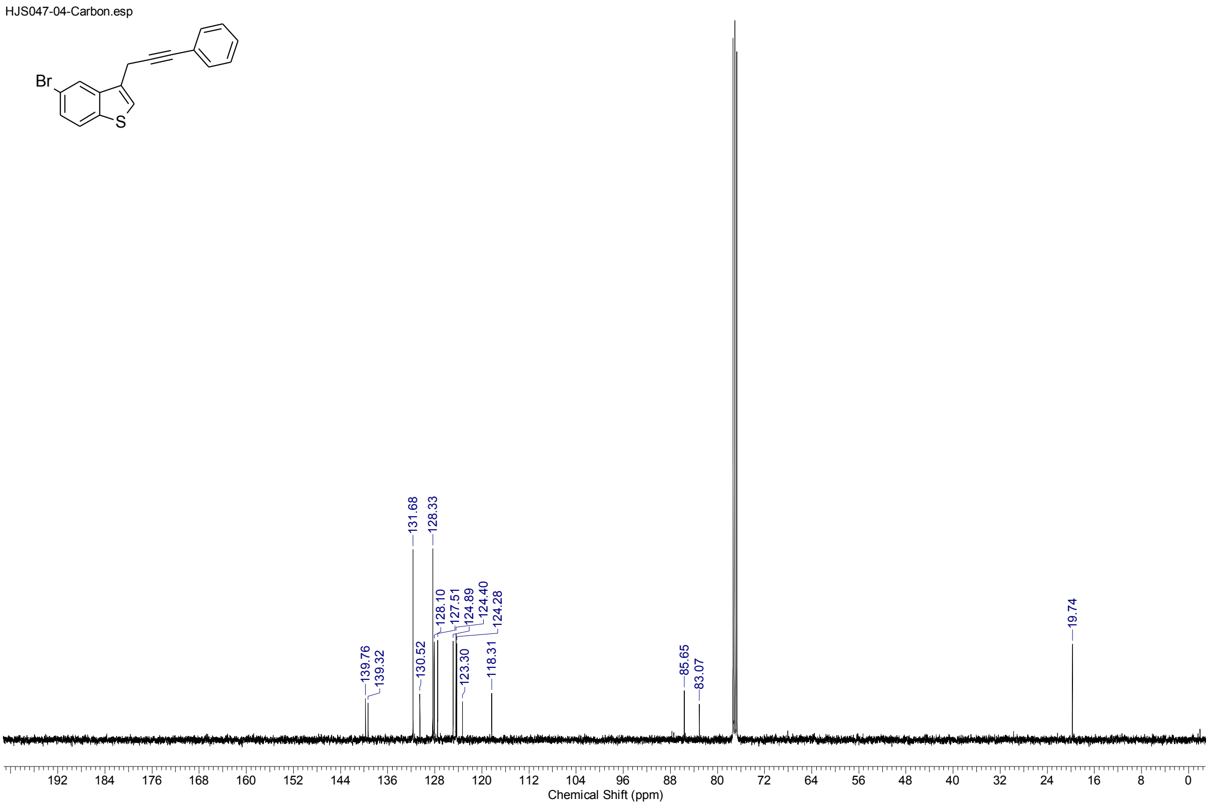

Supplementary Figure 55.

8p ^{1}H NMR (400 MHz, $CDCl_3$)

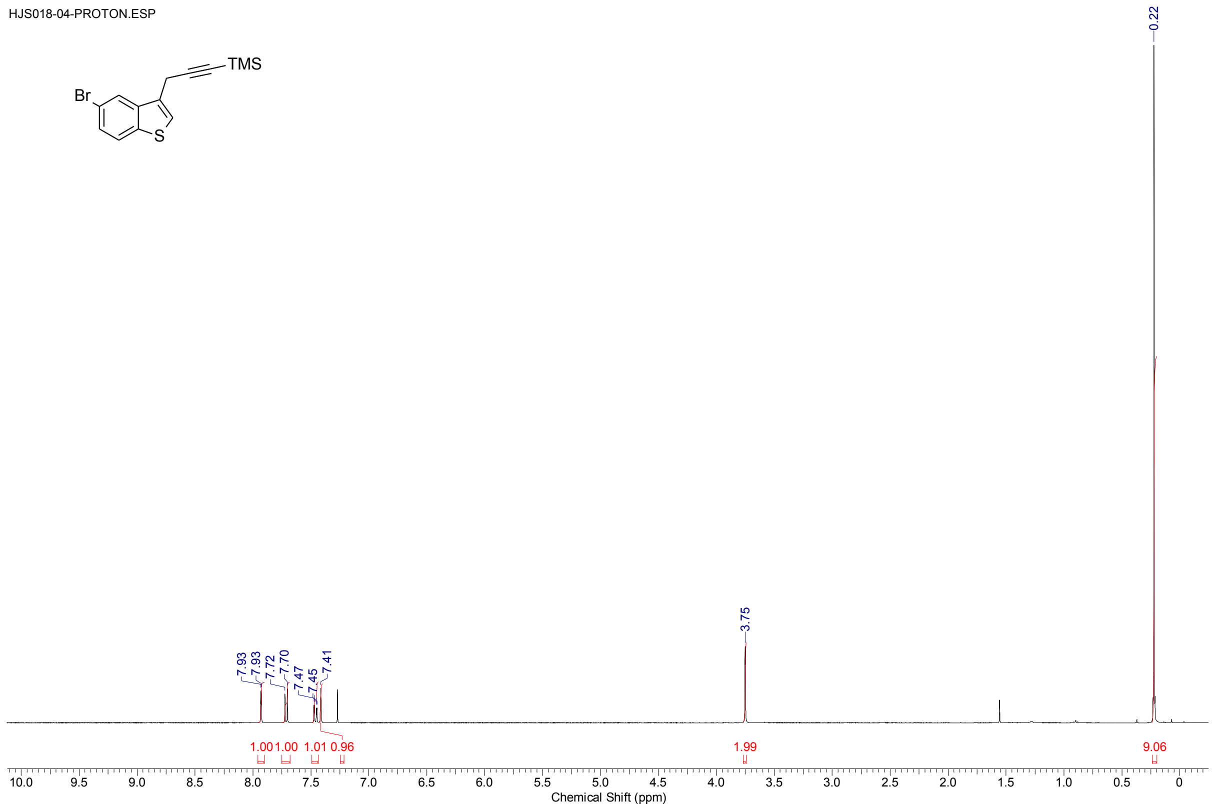

8p ^{13}C NMR (101 MHz, $CDCl_3$)

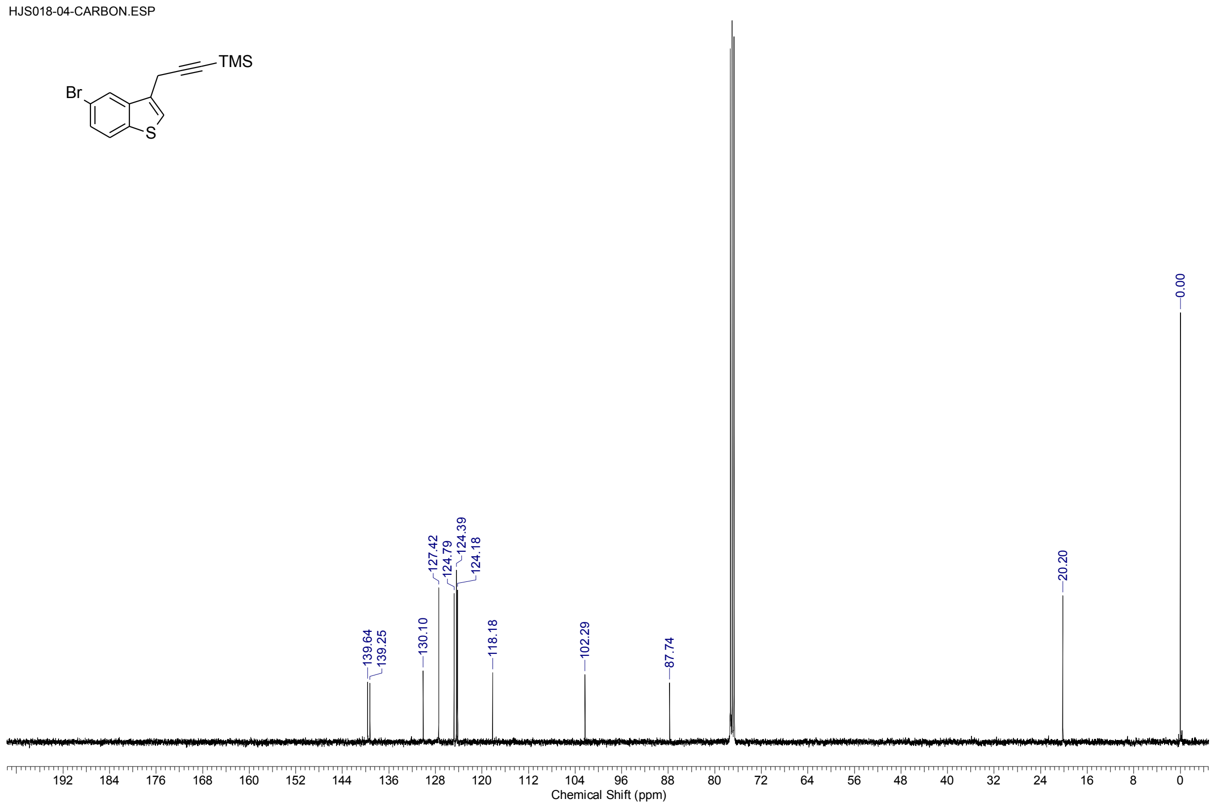

Supplementary Figure 56.

8q ^{1}H NMR (400 MHz, $CDCl_3$)

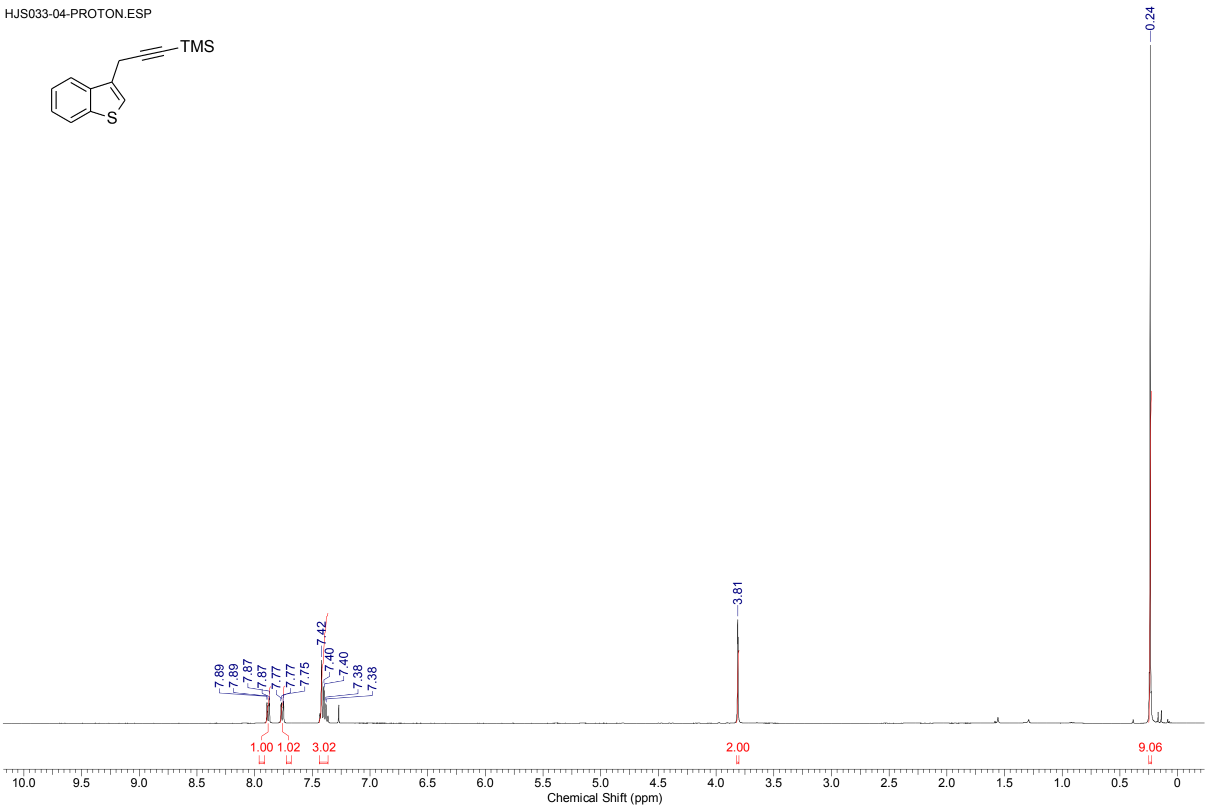

8q ^{13}C NMR (101 MHz, $CDCl_3$)

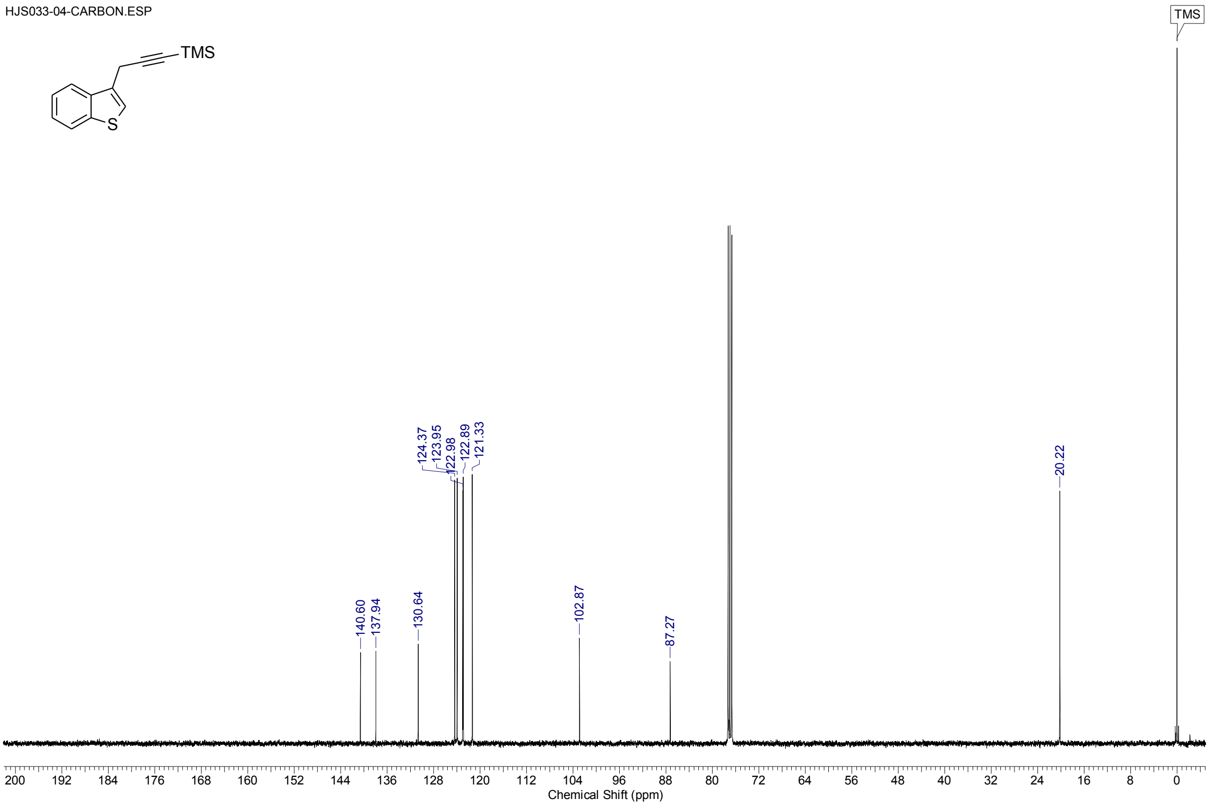

Supplementary Figure 57.

8r ^{1}H NMR (400 MHz, $CDCl_3$)

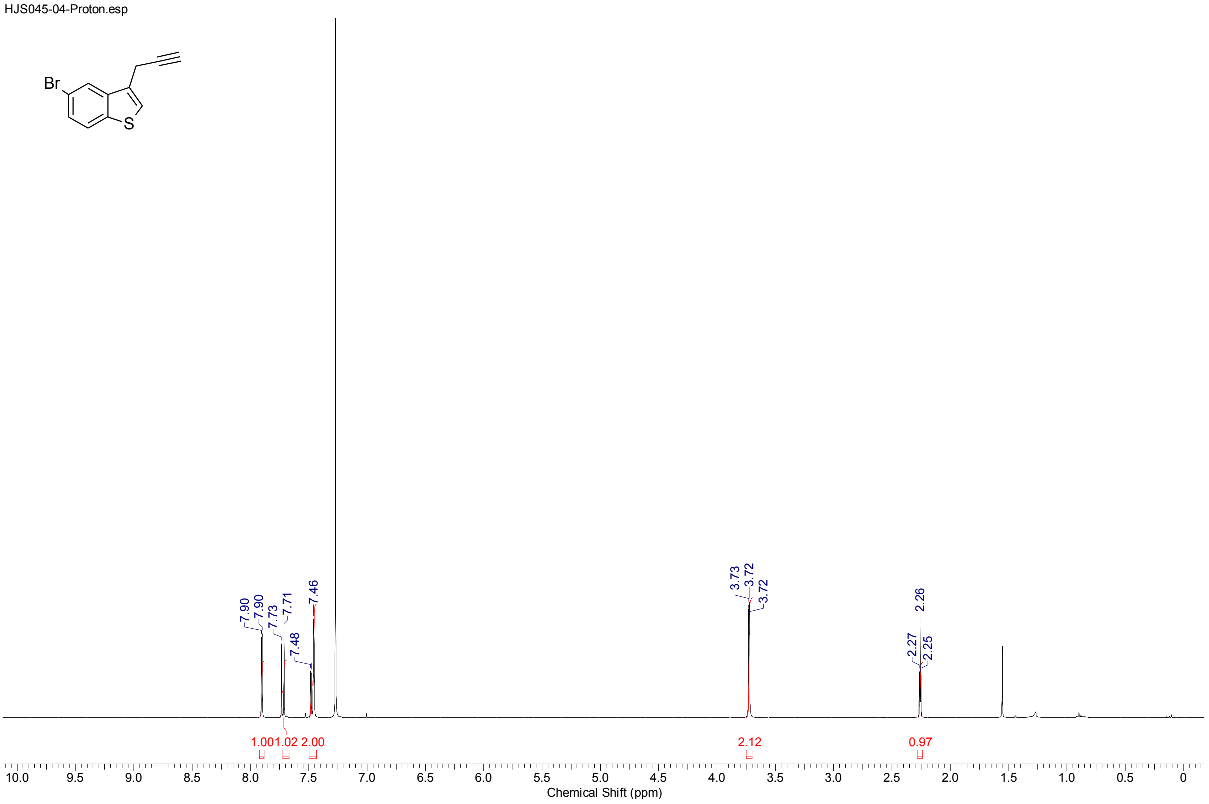

8r ^{13}C NMR (101 MHz, $CDCl_3$)

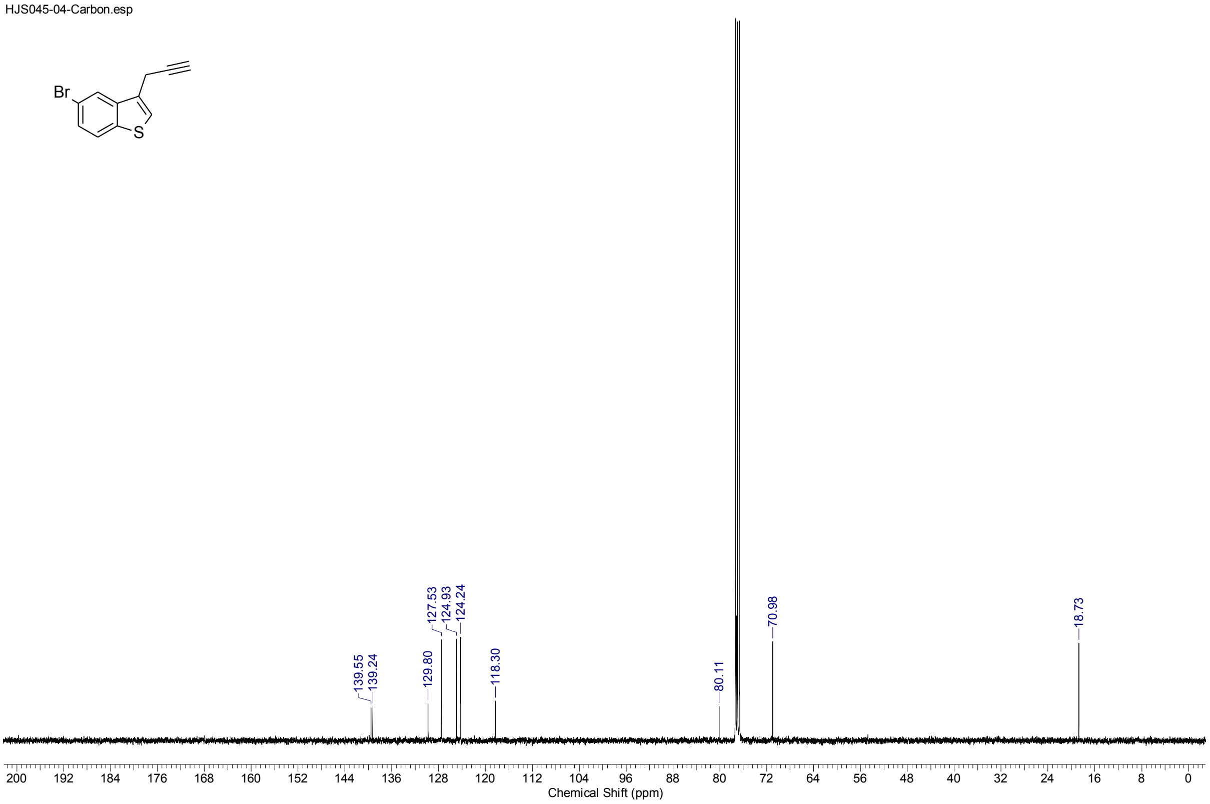

Supplementary Figure 58.

8s ^{1}H NMR (400 MHz, $CDCl_3$)

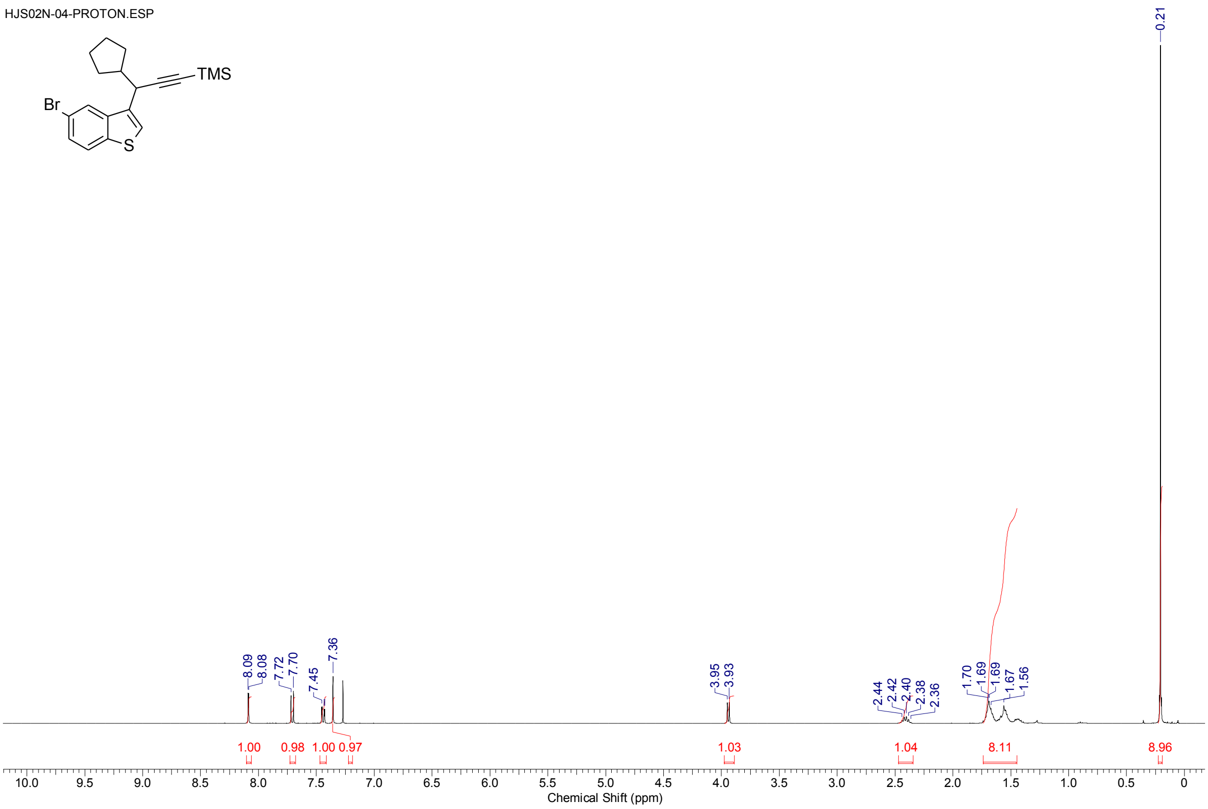

8s ^{13}C NMR (101 MHz, $CDCl_3$)

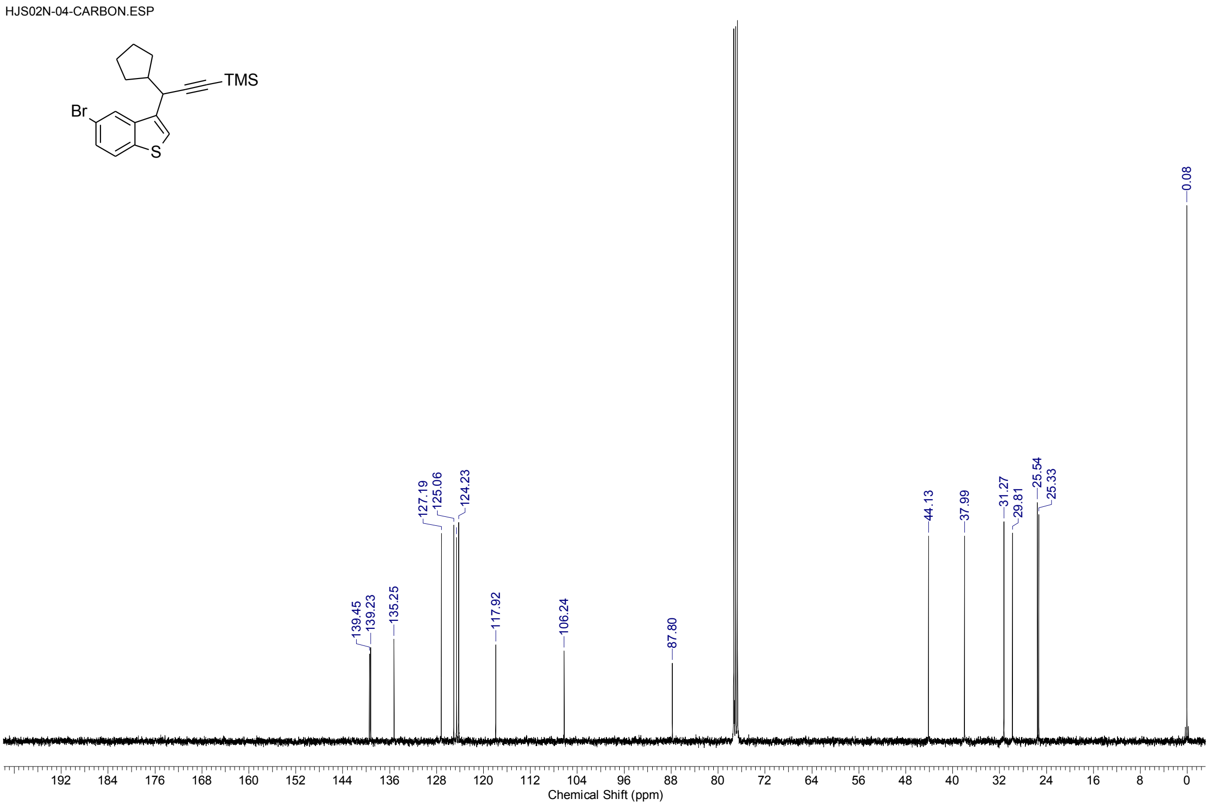

Supplementary Figure 59.

8t ^{1}H NMR (400 MHz, $CDCl_3$)

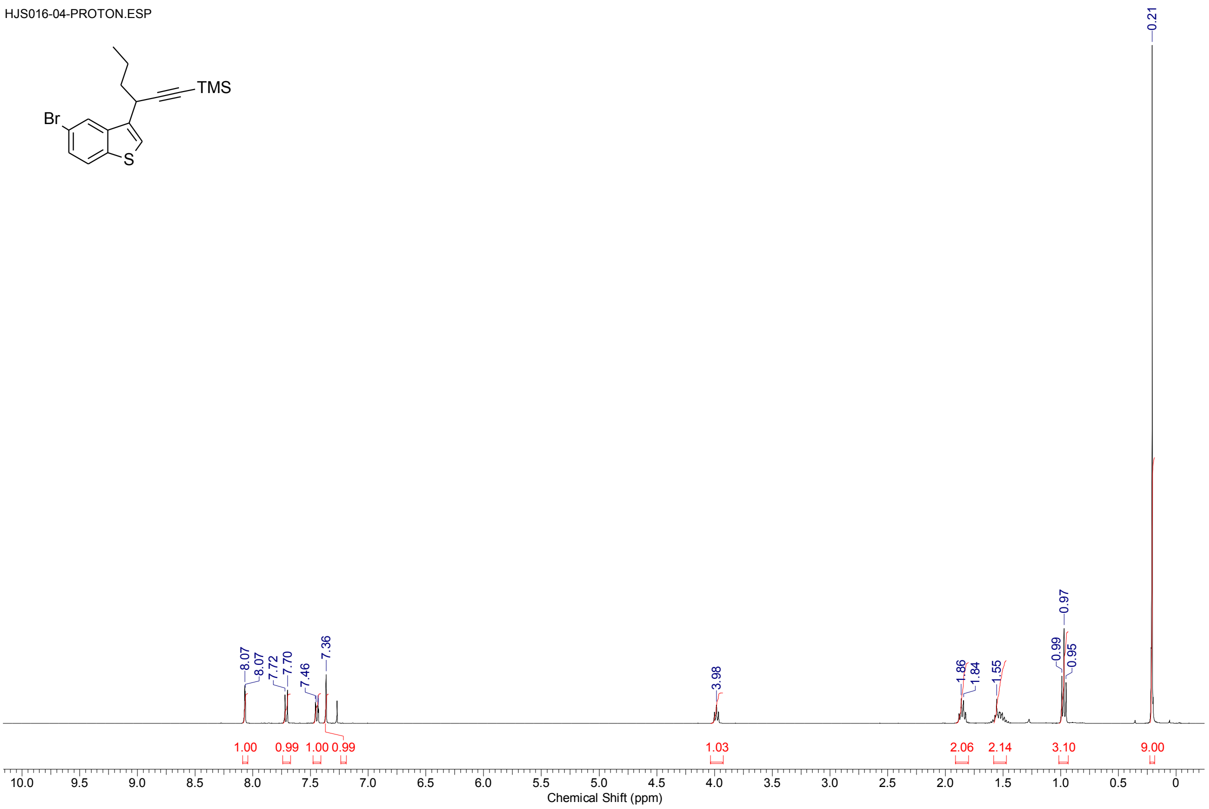

8t ^{13}C NMR (101 MHz, $CDCl_3$)

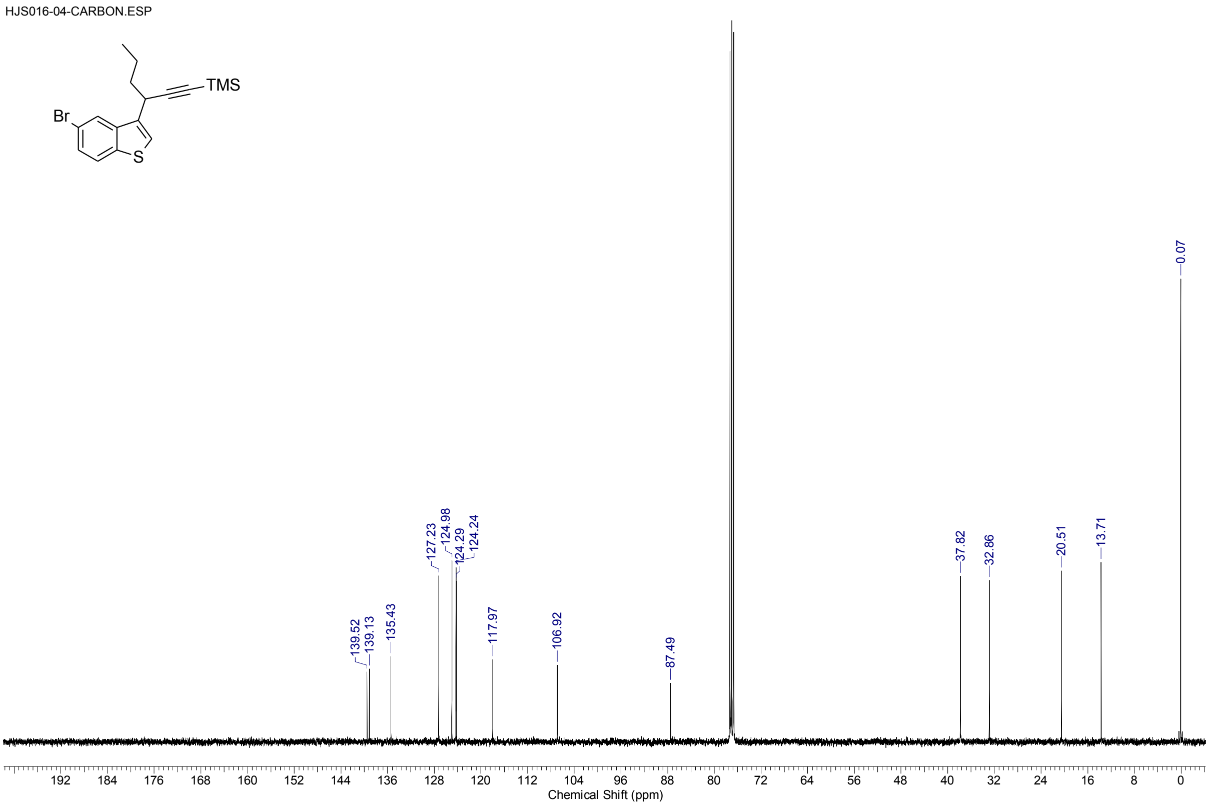

Supplementary Figure 60.

8u ^{1}H NMR (400 MHz, $CDCl_3$)

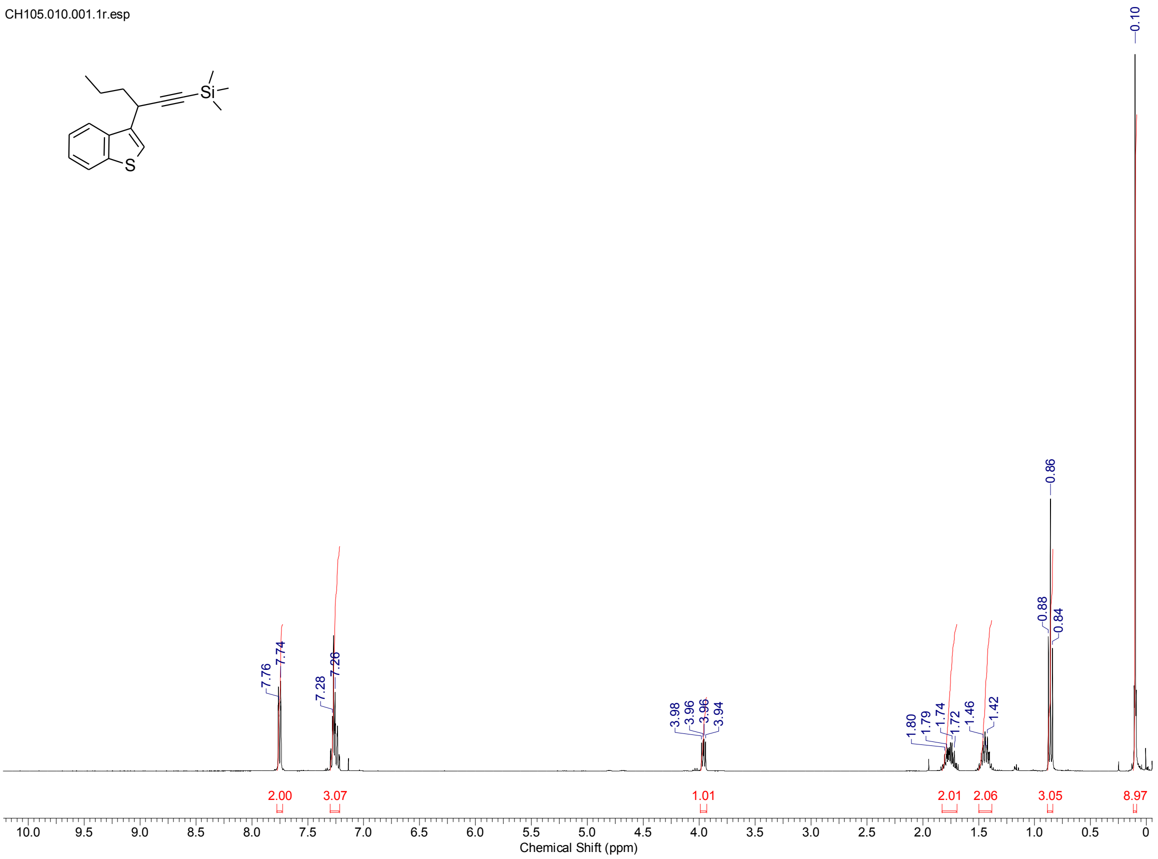

8u ^{13}C NMR (101 MHz, $CDCl_3$)

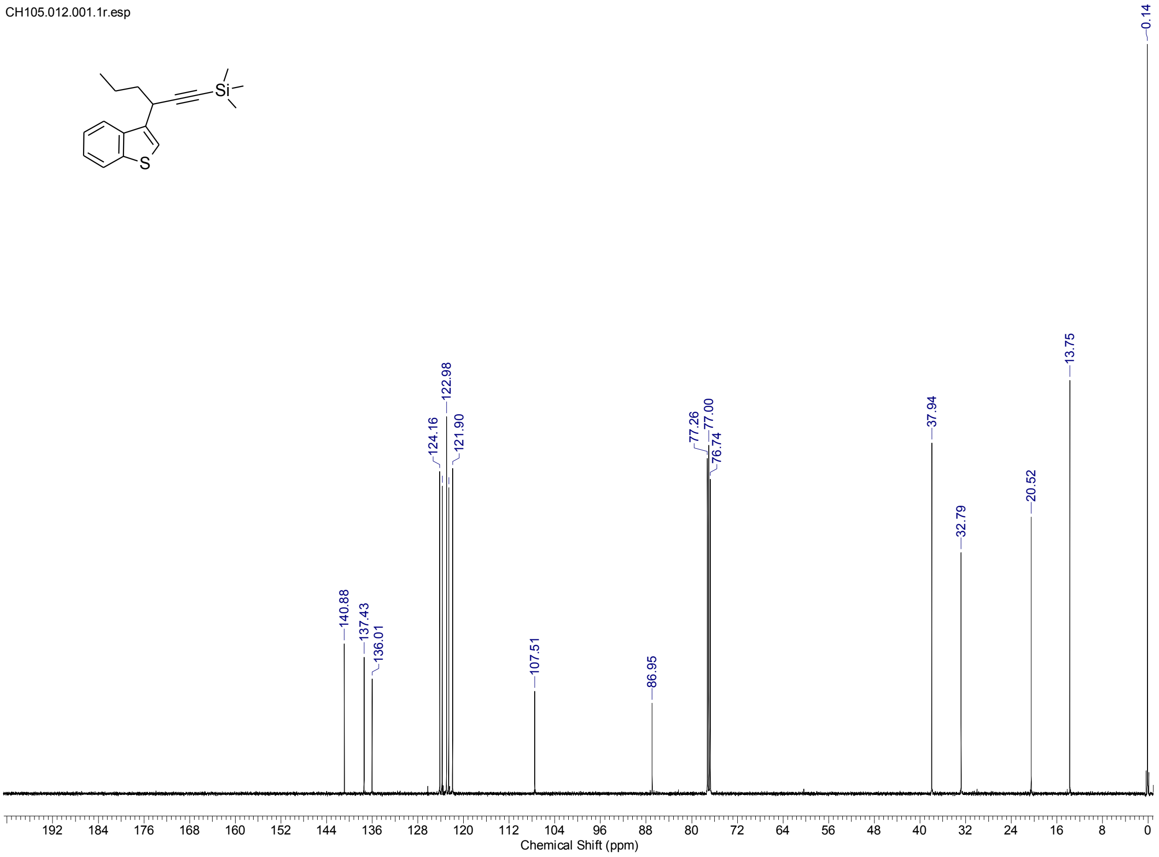

Supplementary Figure 61.

8v ^{1}H NMR (400 MHz, $CDCl_3$)

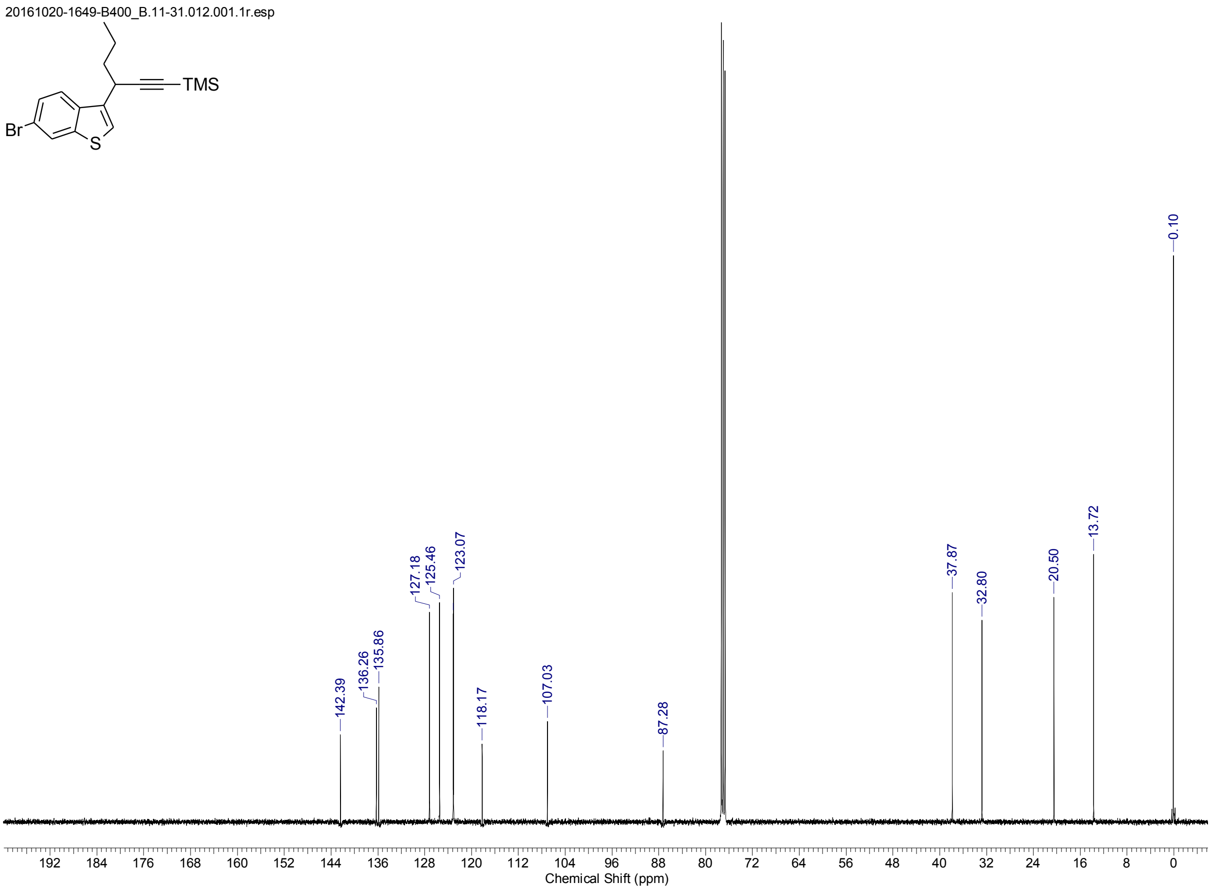

8v ^{13}C NMR (101 MHz, $CDCl_3$)

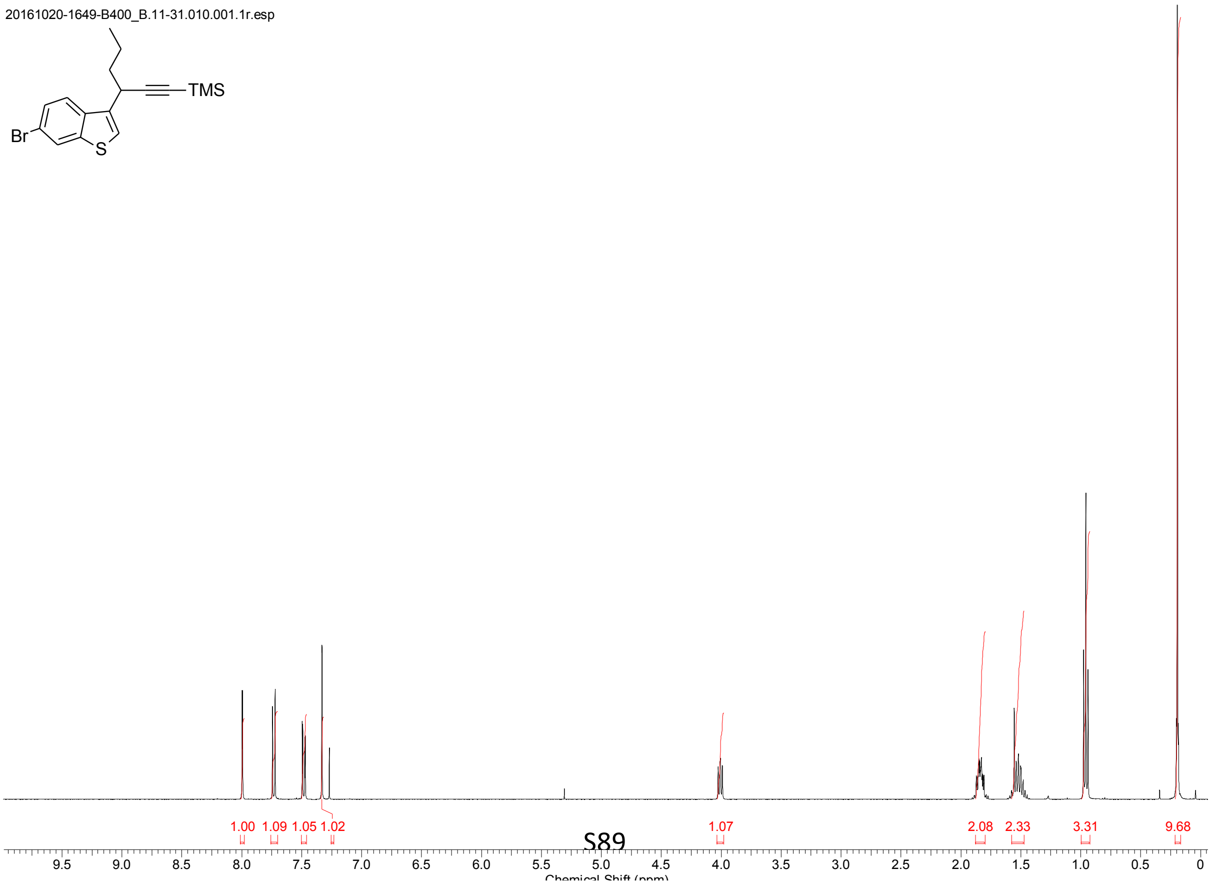

Supplementary Figure 62.
X-Ray structure and CCDC number. Compound 3a
CCDC 1511568

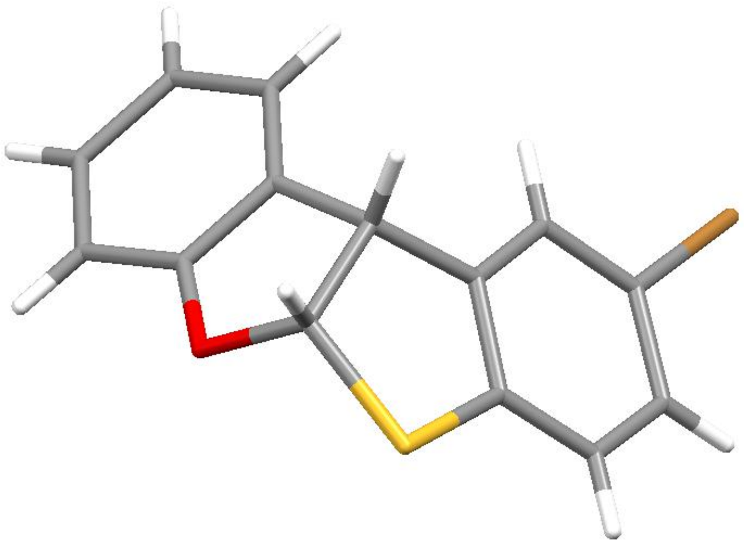

Supplementary Table 1.
Table 1. Crystal data and structure refinement for s4511ma.

Identification code	s4511ma
Empirical formula	C14 H9 Br O S
Formula weight	305.18
Temperature	150(2) K
Wavelength	1.54178 A
Crystal system, space group	Monoclinic, P2(1)
Unit cell dimensions	a = 5.9996(3) A alpha = 90 deg.<br>b = 4.9812(3) A beta = 91.680(4) deg.<br>c = 18.7925(10) A gamma = 90 deg.
Volume	561.38(5) A^3
Z, Calculated density	2, 1.805 Mg/m^3
Absorption coefficient	6.533 mm^-1
F(000)	304
Crystal size	0.190 x 0.150 x 0.030 mm
Theta range for data collection	2.352 to 71.995 deg.
Limiting indices	-7<=h<=7, -6<=k<=4, -23<=l<=23
Reflections collected / unique	3042 / 1605 [R(int) = 0.0529]

Completeness to theta = 67.679   97.8 %

Absorption correction   Semi-empirical from equivalents

Max. and min. transmission   0.828 and 0.586829

Refinement method   Full-matrix least-squares on F^2

Data / restraints / parameters   1605 / 1 / 154

Goodnes *S*-of-fit on F^2   1.065

Final R indices [I>2sigma(I)]   R1 = 0.0411, wR2 = 0.1100

R indices (all data)   R1 = 0.0432, wR2 = 0.1114

Absolute structure parameter   0.03(4)

Extinction coefficient   n/a

Largest diff. peak and hole   1.800 and -0.524 e.A^-3

Supplementary Table 2.

Table 2. Atomic coordinates (x 10^4) and equivalent isotropic displacement parameters (A^2 x 10^3) for s4511ma.
U(eq) is defined as one third of the trace of the orthogonalized Uij tensor.

	x	y	z	U(eq)
Br(1)	4115(1)	12489(2)	622(1)	27(1)
C(1)	2418(13)	9832(18)	1120(4)	25(2)
C(2)	322(13)	9219(17)	835(4)	26(2)
C(3)	-908(12)	7280(20)	1196(4)	26(2)
C(4)	-87(12)	6181(18)	1823(4)	24(2)
C(5)	655(12)	3977(17)	3050(4)	25(2)
C(6)	1557(11)	7260(20)	3838(4)	25(2)
C(7)	1548(14)	8947(19)	4435(4)	27(2)
C(8)	3332(14)	10680(19)	4549(4)	31(2)
C(9)	5118(13)	10761(18)	4081(4)	28(2)
C(10)	5111(12)	9022(19)	3496(4)	27(2)
C(11)	3307(11)	7330(20)	3375(3)	24(1)
C(12)	2734(12)	5445(17)	2771(4)	24(2)
C(13)	2041(11)	6862(14)	2086(4)	21(2)
C(14)	3295(11)	8760(17)	1735(4)	22(2)
O(1)	-104(9)	5554(12)	3650(3)	27(1)
S(1)	-1418(3)	3765(4)	2340(1)	27(1)

Supplementary Table 3.

Table 3. Bond lengths [A] and angles [deg] for s4511ma.

Br(1)-C(1)	1.929(8)
C(1)-C(14)	1.363(11)

C(1)-C(2)	1.386(11)
C(2)-C(3)	1.404(13)
C(2)-H(2)	0.9500
C(3)-C(4)	1.376(11)
C(3)-H(3)	0.9500
C(4)-C(13)	1.397(10)
C(4)-S(1)	1.754(8)
C(5)-O(1)	1.458(9)
C(5)-C(12)	1.550(10)
C(5)-S(1)	1.799(8)
C(5)-H(5)	1.0000
C(6)-O(1)	1.348(10)
C(6)-C(11)	1.384(9)
C(6)-C(7)	1.402(12)
C(7)-C(8)	1.387(12)
C(7)-H(7)	0.9500
C(8)-C(9)	1.406(11)
C(8)-H(8)	0.9500
C(9)-C(10)	1.401(12)
C(9)-H(9)	0.9500
C(10)-C(11)	1.384(12)
C(10)-H(10)	0.9500
C(11)-C(12)	1.505(12)
C(12)-C(13)	1.516(10)
C(12)-H(12)	1.0000
C(13)-C(14)	1.387(10)
C(14)-H(14)	0.9500

C(14)-C(1)-C(2)	124.3(8)
C(14)-C(1)-Br(1)	118.9(6)
C(2)-C(1)-Br(1)	116.7(6)
C(1)-C(2)-C(3)	116.7(7)
C(1)-C(2)-H(2)	121.7
C(3)-C(2)-H(2)	121.7
C(4)-C(3)-C(2)	120.5(7)
C(4)-C(3)-H(3)	119.8
C(2)-C(3)-H(3)	119.8
C(3)-C(4)-C(13)	120.4(7)
C(3)-C(4)-S(1)	126.1(6)
C(13)-C(4)-S(1)	113.4(6)
O(1)-C(5)-C(12)	106.3(6)
O(1)-C(5)-S(1)	112.3(5)
C(12)-C(5)-S(1)	108.8(5)
O(1)-C(5)-H(5)	109.8
C(12)-C(5)-H(5)	109.8
S(1)-C(5)-H(5)	109.8
O(1)-C(6)-C(11)	114.9(8)
O(1)-C(6)-C(7)	124.5(6)
C(11)-C(6)-C(7)	120.5(9)
C(8)-C(7)-C(6)	118.5(7)
C(8)-C(7)-H(7)	120.8
C(6)-C(7)-H(7)	120.8
C(7)-C(8)-C(9)	121.3(8)
C(7)-C(8)-H(8)	119.3
C(9)-C(8)-H(8)	119.3
C(10)-C(9)-C(8)	119.2(8)
C(10)-C(9)-H(9)	120.4
C(8)-C(9)-H(9)	120.4
C(11)-C(10)-C(9)	119.3(7)
C(11)-C(10)-H(10)	120.3

C(9)-C(10)-H(10)	120.3
C(10)-C(11)-C(6)	121.1(8)
C(10)-C(11)-C(12)	131.6(6)
C(6)-C(11)-C(12)	107.2(7)
C(11)-C(12)-C(13)	113.6(7)
C(11)-C(12)-C(5)	102.0(6)
C(13)-C(12)-C(5)	107.6(6)
C(11)-C(12)-H(12)	111.1
C(13)-C(12)-H(12)	111.1
C(5)-C(12)-H(12)	111.1
C(14)-C(13)-C(4)	120.0(7)
C(14)-C(13)-C(12)	125.6(7)
C(4)-C(13)-C(12)	114.4(7)
C(1)-C(14)-C(13)	117.9(7)
C(1)-C(14)-H(14)	121.0
C(13)-C(14)-H(14)	121.0
C(6)-O(1)-C(5)	107.2(6)
C(4)-S(1)-C(5)	93.1(4)

Symmetry transformations used to generate equivalent atoms:

Supplementary Table 4.

Table 4. Anisotropic displacement parameters (A^2 x 10^3) for s4511ma.
The anisotropic displacement factor exponent takes the form:
-2 pi^2 [h^2 a*^2 U11 + ... + 2 h k a* b* U12]

	U11	U22	U33	U23	U13	U12
Br(1)	25(1)	26(1)	30(1)	1(1)	5(1)	-4(1)
C(1)	21(4)	27(4)	26(3)	-1(3)	5(3)	4(3)
C(2)	23(4)	24(4)	30(4)	-1(3)	1(3)	5(3)
C(3)	21(3)	25(4)	32(3)	-4(4)	1(3)	-3(4)
C(4)	11(3)	24(4)	36(4)	-8(3)	5(3)	-2(3)
C(5)	22(4)	26(4)	29(3)	1(3)	9(3)	2(3)
C(6)	22(3)	19(4)	34(3)	7(4)	5(3)	3(4)
C(7)	25(4)	29(5)	27(3)	0(3)	6(3)	1(3)
C(8)	25(4)	38(5)	29(4)	-1(4)	-3(3)	5(4)
C(9)	19(4)	29(5)	35(4)	2(3)	-3(3)	0(3)
C(10)	15(4)	34(5)	31(4)	4(3)	-1(3)	2(3)
C(11)	17(3)	27(4)	27(3)	8(4)	0(2)	0(4)
C(12)	15(3)	26(4)	30(4)	0(3)	2(3)	2(3)
C(13)	14(3)	22(5)	29(3)	-5(3)	4(3)	-2(2)
C(14)	12(3)	20(4)	33(4)	-8(3)	6(3)	-3(3)
O(1)	20(3)	30(3)	31(3)	-2(2)	5(2)	-4(2)
S(1)	18(1)	29(1)	35(1)	1(1)	4(1)	-5(1)

Supplementary Table 5.

Table 5. Hydrogen coordinates (x 10^4) and isotropic displacement parameters (A^2 x 10^3) for s4511ma.

	x	y	z	U(eq)
H(2)	-252	10070	416	31
H(3)	-2318	6717	1007	31
H(5)	1078	2132	3214	31
H(7)	349	8905	4753	33
H(8)	3347	11836	4951	37
H(9)	6316	11982	4162	34
H(10)	6331	9000	3185	32
H(12)	3979	4155	2692	29
H(14)	4720	9296	1917	26

Supplementary Table 6.

Table 6. Torsion angles [deg] for s4511ma.

C(14)-C(1)-C(2)-C(3)	-2.8(13)
Br(1)-C(1)-C(2)-C(3)	179.8(6)
C(1)-C(2)-C(3)-C(4)	3.5(13)
C(2)-C(3)-C(4)-C(13)	-3.9(13)
C(2)-C(3)-C(4)-S(1)	179.5(7)
O(1)-C(6)-C(7)-C(8)	-177.4(8)
C(11)-C(6)-C(7)-C(8)	-0.2(13)
C(6)-C(7)-C(8)-C(9)	0.0(13)
C(7)-C(8)-C(9)-C(10)	-1.1(13)
C(8)-C(9)-C(10)-C(11)	2.5(12)
C(9)-C(10)-C(11)-C(6)	-2.7(13)
C(9)-C(10)-C(11)-C(12)	174.3(9)
O(1)-C(6)-C(11)-C(10)	179.1(8)
C(7)-C(6)-C(11)-C(10)	1.6(14)
O(1)-C(6)-C(11)-C(12)	1.4(11)
C(7)-C(6)-C(11)-C(12)	-176.1(8)
C(10)-C(11)-C(12)-C(13)	-71.8(12)
C(6)-C(11)-C(12)-C(13)	105.5(8)
C(10)-C(11)-C(12)-C(5)	172.7(10)
C(6)-C(11)-C(12)-C(5)	-10.0(9)
O(1)-C(5)-C(12)-C(11)	14.7(8)
S(1)-C(5)-C(12)-C(11)	135.9(6)
O(1)-C(5)-C(12)-C(13)	-105.1(7)
S(1)-C(5)-C(12)-C(13)	16.1(8)
C(3)-C(4)-C(13)-C(14)	3.4(12)
S(1)-C(4)-C(13)-C(14)	-179.7(6)
C(3)-C(4)-C(13)-C(12)	-178.6(8)
S(1)-C(4)-C(13)-C(12)	-1.7(8)
C(11)-C(12)-C(13)-C(14)	56.1(9)
C(5)-C(12)-C(13)-C(14)	168.3(7)
C(11)-C(12)-C(13)-C(4)	-121.7(7)
C(5)-C(12)-C(13)-C(4)	-9.6(9)
C(2)-C(1)-C(14)-C(13)	2.3(12)
Br(1)-C(1)-C(14)-C(13)	179.7(6)
C(4)-C(13)-C(14)-C(1)	-2.5(11)

C(12)-C(13)-C(14)-C(1)	179.8(7)
C(11)-C(6)-O(1)-C(5)	8.6(10)
C(7)-C(6)-O(1)-C(5)	-174.0(8)
C(12)-C(5)-O(1)-C(6)	-14.6(8)
S(1)-C(5)-O(1)-C(6)	-133.5(6)
C(3)-C(4)-S(1)-C(5)	-173.1(8)
C(13)-C(4)-S(1)-C(5)	10.1(6)
O(1)-C(5)-S(1)-C(4)	102.3(6)
C(12)-C(5)-S(1)-C(4)	-15.1(6)

Symmetry transformations used to generate equivalent atoms:

Supplementary Table 7.

Table 7. Hydrogen bonds for s4511ma [A and deg.].

D-H...A	d(D-H)	d(H...A)	d(D...A)	<(DHA)
C(12)-H(12)...S(1)#1	1.00	2.86	3.719(8)	143.8
C(3)-H(3)...Br(1)#2	0.95	3.07	3.948(9)	153.6

Symmetry transformations used to generate equivalent atoms:
#1 x+1,y,z #2 x-1,y-1,z

Supplementary References

1] Pouzet, P., Erdelmeier, I., Dansette, P. M. & Mansuy, D. Synthesis of (4-chlorophenyl)-(1-oxo-1λ^4-benzo[b]thien-2-yl)methanone and study of its reactivity towards sulfur- and oxygen-containing nucleophiles. *Tetrahedron*, **54**, 14811-14824 (1998).

2] Iaroshenko, V. O., Ali, S., Mkrtchyan, S., Gevorgyan, A., Babar, T. M., Semeniuchenko, V., Hassan, Z., Villinger, A. & Langer, P. Design and synthesis of condensed thienocoumarins by Suzuki–Miyaura reaction/lactonization tandem protocol. *Tetrahedron Letters*, **53**, 7135-7139 (2012).

3] Blanco, B., Sedes, A., Peón, A., Lamb, H., Hawkins, A. R. Castedo, L. & González-Bello, C. Synthesis of 3-alkyl enol mimics inhibitors of type II dehydroquinase: factors influencing their inhibition potency. *Org. Biomol. Chem.* **10**, 3662-3676 (2012).
